# Tertiary Amine Coupling by Oxidation for Selective Labeling of Dimethyl Lysine Post-Translational Modifications

**DOI:** 10.1021/jacs.4c00253

**Published:** 2024-04-08

**Authors:** Benjamin Emenike, Patrick Czabala, Jonathan Farhi, Jagannath Swaminathan, Eric V. Anslyn, Jennifer Spangle, Monika Raj

**Affiliations:** † Department of Chemistry, Emory University, Atlanta, Georgia 30322, United States; ‡ Department of Radiation Oncology, Emory University School of Medicine, Atlanta, Georgia 30322, United States; § Department of Chemistry, University of Texas at Austin, Austin, Texas 78712, United States

## Abstract

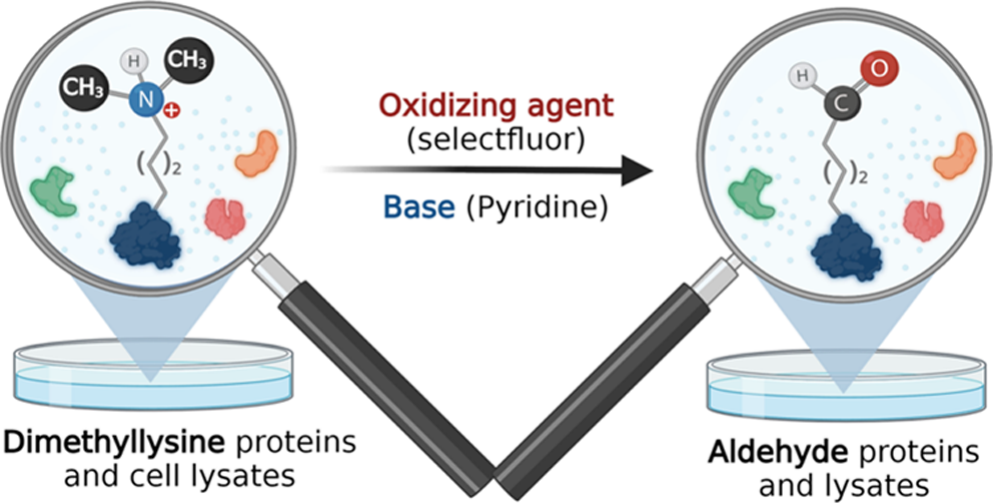

Lysine dimethylation (Kme_2_) is a crucial post-translational modification (PTM) that regulates biological processes and is implicated in diseases. There is significant interest in globally identifying these methylation marks. Unfortunately, this remains challenging due to the lack of robust technologies for selectively labeling Kme_2_. To address this, we present a chemical method named tertiary amine coupling by oxidation (TACO). This method selectively modifies Kme_2_ to aldehydes using Selectfluor and a base. The resulting aldehydes from Kme_2_ were then functionalized using reductive amination, thiolamine, and oxime chemistry. We successfully demonstrated the versatility of TACO in selectively labeling Kme_2_ peptides and proteins in complex cell lysate mixtures with varying payloads, including affinity tags and fluorophores. We further showed the application of TACO chemistry for the identification of Kme_2_ sites at a single-molecule level by fluorosequencing. We discovered novel 30 Kme_2_ sites, in addition to previously known 5 Kme_2_ sites, by proteomics analysis of TACO-modified nuclear extracts. Our work establishes a unique strategy for covalently modifying Kme_2_, facilitating the global identification of low-abundance Kme_2_-PTMs and their sites within complex cell lysate mixtures.

## Introduction

**Figure 1:**
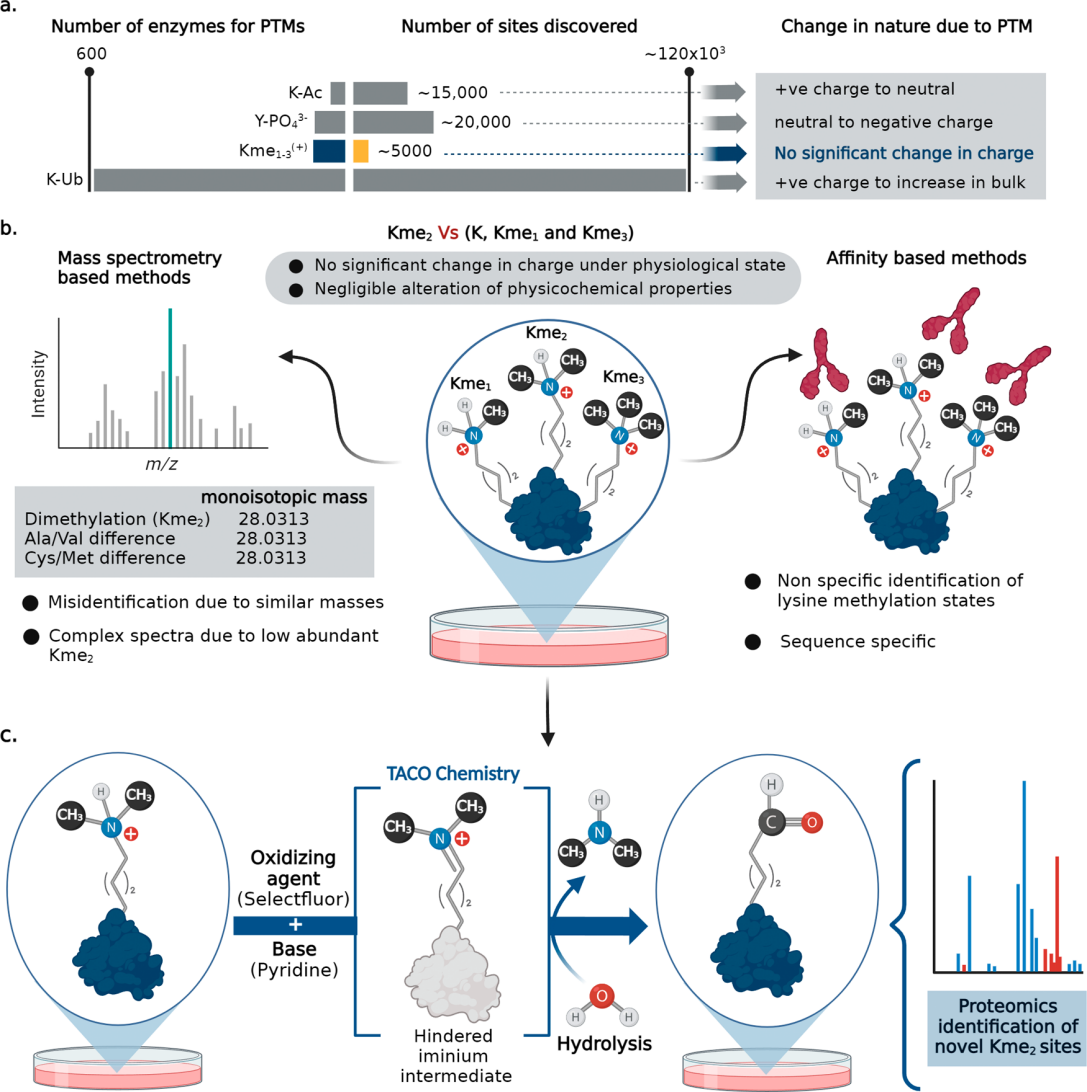
Different lysine methylation states and methods to profile dimethyl lysine. (a) Number of lysine methylation states discovered so far are much lower than enzymes responsible for such PTMs. The major challenge in identifying lysine methylation is due to the negligible change in the charge and physicochemical properties. (b) Limitations of the affinity-based method and MS in identifying lysine methylation. (c) This work: Development of the oxidative coupling chemical reaction for the selective modification of Kme_2_ by hydrolysis of iminium ions generating clickable aldehyde sites for identification by proteomics and fluorosequencing.

Lysine dimethylation (Kme_2_) dynamics constitute a crucial epigenetic event heavily involved in gene expression regulation and various biological processes.^1−3^ Despite increasing interest in this post-translational modification (PTM) and its pivotal role in diverse biological events and diseases beyond chromatin organization, its global identification remains elusive (Figure 1a). This is largely due to the lack of pan-specific chemical tools for labeling dimethyllysine (Kme_2_). Attempts to identify and characterize lysine dimethylation Kme_2_ have relied heavily on the use of affinity reagents such as antibodies and methyl-binding domains, MBD.^4−7^ However, these reagents fail to globally detect Kme_2_ sites due to their sequence-specific nature and limited specificity for different lysine methylation states. Antibodies are further constrained by the unequal abundance of lysine methylation states, reducing the fidelity in measuring methylation levels (Figure 1b). These challenges can be partially attributed to no significant change in the charge and negligible change in the bulk, hydrophobicity, and physicochemical properties of lysine upon the addition of methyl groups (Figure 1b).^4,8−10^ Furthermore, affinity-based methods are unable to detect trypsin-digested Kme_2_ fragments because they require flanking amino acids on either side of Kme_2_ for efficient recognition.^11,12^ Alternatively, mass spectrometry (MS)-based proteomics for detecting these low-abundant Kme_2_ PTMs require additional modification to reduce sample complexity and minimize false positive due to misassignment resulting from amino acid combinations with similar masses to two methyl groups (e.g., Ala vs Val and Cys vs Met) (Figure 1b).^10,13,14^ Collectively, none of the existing methods can selectively label Kme_2_ in a specific manner, underscoring the necessity for robust chemical methods for this purpose. Herein, we introduce a pioneering chemical strategy, termed tertiary amine coupling by oxidation (TACO), designed for the selective labeling of tertiary amines (Kme_2_) to aldehydes regardless of the surrounding amino acid sequence (Figure 1c).^15−18^ We demonstrated the broad applicability of TACO chemistry by selectively labeling Kme_2_ on peptides, proteins, nucleosomes, and whole cell lysates using various payloads like affinity tags and fluorophores, independent of the sequence, adjacent PTMs, or multiple Kme_2_ modifications.

We demonstrated the potential of TACO chemistry in identifying Kme_2_ sites at the single molecule level through fluorosequencing. Additionally, we applied TACO chemistry to label and enrich Kme_2_ proteins, subsequently conducting a proteomic analysis on the labeled samples. This led to the discovery of 30 novel Kme_2_ sites. Notably, there are no chemical methods available in the literature for the site-selective labeling of Kme_2_. The capability to selectively label Kme_2_ sites using various tags in a complex mixture renders this technology exceptionally valuable for identifying and quantifying Kme_2_ biomarkers across diverse biological samples.

## Results and Discussion

### Development of TACO

**Figure 2:**
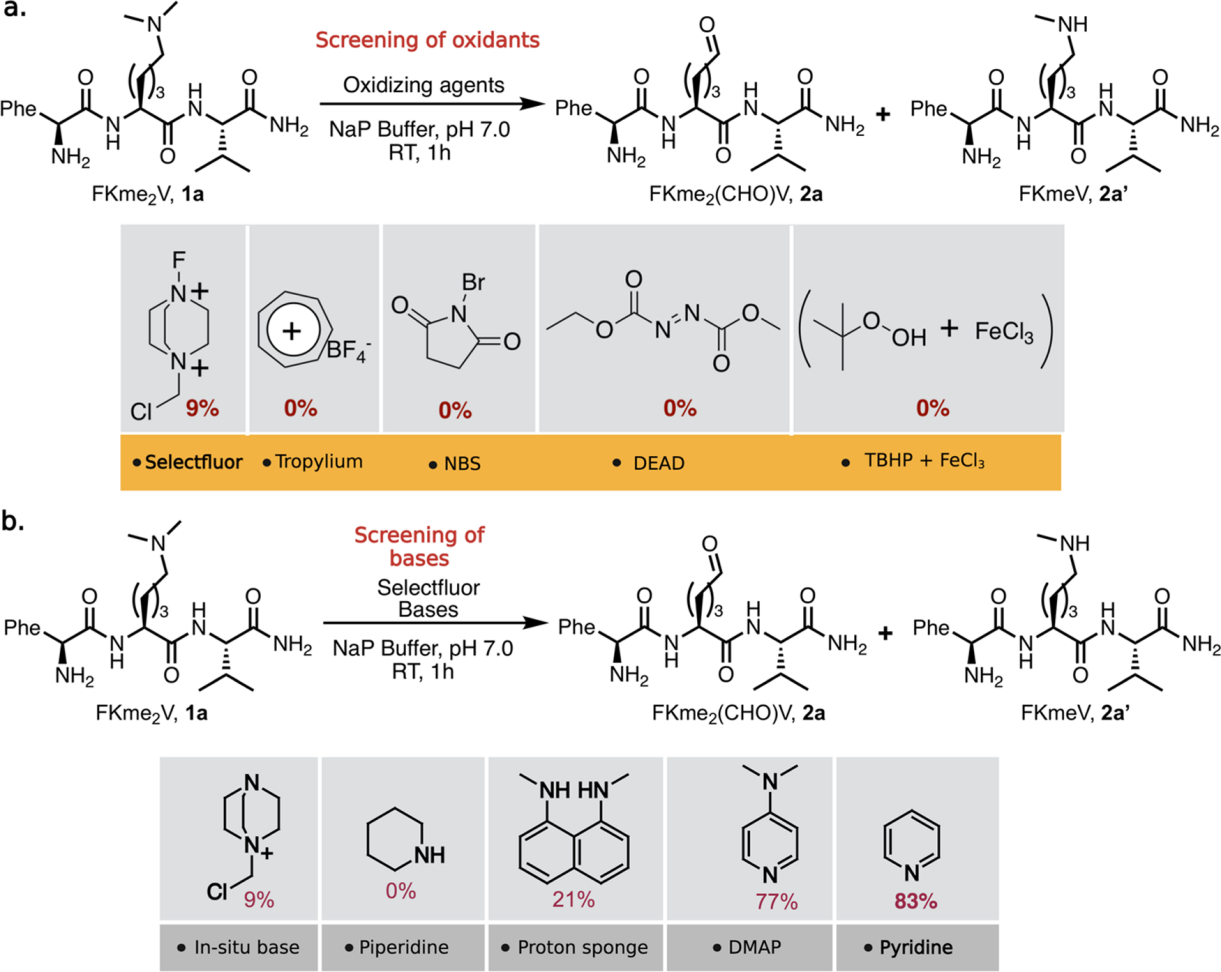
Optimization of the TACO chemistry. (a) Development of the TACO reaction for modification of dimethyllysine Kme_2_ in peptide FKme_2_V **1a** by exploring varying oxidizing reagents. (b) Evaluation of bases and optimization to the peptide aldehyde FKme_2_(CHO)V **2a**. Reaction conditions: To 6–10 mM of dimethyllysine peptide in 300 μL of 10 mM sodium phosphate buffer (NaP, pH 7.0) were added a base (5 equiv) and oxidizing reagent (2 equiv), and the reaction mixture was stirred for 1 h at room temperature.

The tertiary amine coupling by oxidation (TACO) reaction capitalizes on the tendency of tertiary amines to generate highly electrophilic iminium ions under oxidative conditions. These ions undergo rapid hydrolysis, leading to the robust conversion of tertiary amines to aldehydes under physiological conditions (Figure 1c).^15,19,^ We explored a variety of oxidizing reagents such as Selectfluor, tropylium tetrafluoroborate, N-bromo succinimide (NBS), diethyl azodicarboxylate (DEAD), and *t*BuOOH (TBHP) with FeCl_3_ under hydrolytic conditions on a model peptide FKme_2_V **1a** (Figures 2a and Supporting Figure 1). Of all the reactions, Selectfluor (2 equiv) resulted in the formation of an aldehyde product, FKme_2_(CHO)V **2a**, with 9% conversion in 1 h in sodium phosphate buffer (10 mM, pH 7) at room temperature. However, this reaction also generated small amounts of monomethyllysine FKme_1_V **2a’**, which coeluted with the starting peptide FKme_2_V **1a** as determined by LCMS (Supporting Figure 1). We postulated that the formation of aldehyde **2a** and monomethyllysine **2a’** resulted from the nucleophilic attack of water on the more hindered and less hindered iminium ion, respectively (Supporting Figure 2). We did not observe the formation of any aldehyde product in the presence of other oxidants such as tropylium, NBS, DEAD, and (TBHP + FeCl_3_), due to their poor water compatibility and instability in aqueous conditions. To enhance the conversion of the dimethyl lysine peptide to the peptide aldehyde, we introduced bases stronger than those generated in situ by Selectfluor. Screening various bases such as piperidine, proton sponge, DMAP, and pyridine, we observed high conversion to the peptide aldehyde product FKme_2_(CHO)V **2a** with DMAP (77%) and pyridine (83%) (Figures 2b and Supporting Figure 3). We attribute the high conversion to the planarity of pyridine and DMAP that enables the abstraction of the hindered methylene proton of dimethyl lysine, resulting in the formation of iminium ions followed by hydrolysis to generate an aldehyde. Other bases such as piperidine, DABCO-like base from Selectfluor, and proton sponge are too bulky to remove the proton from the hindered carbon of dimethyl lysine. Next, we generated high amounts of peptide aldehyde (OAc)FKme_2_(CHO)V **2b** from peptide (OAc)FKme_2_V **1b** under optimized reaction conditions and characterized it by NMR (Supporting Figure 4).

### Chemoselectivity Studies for Peptide Aldehyde

**Figure 3:**
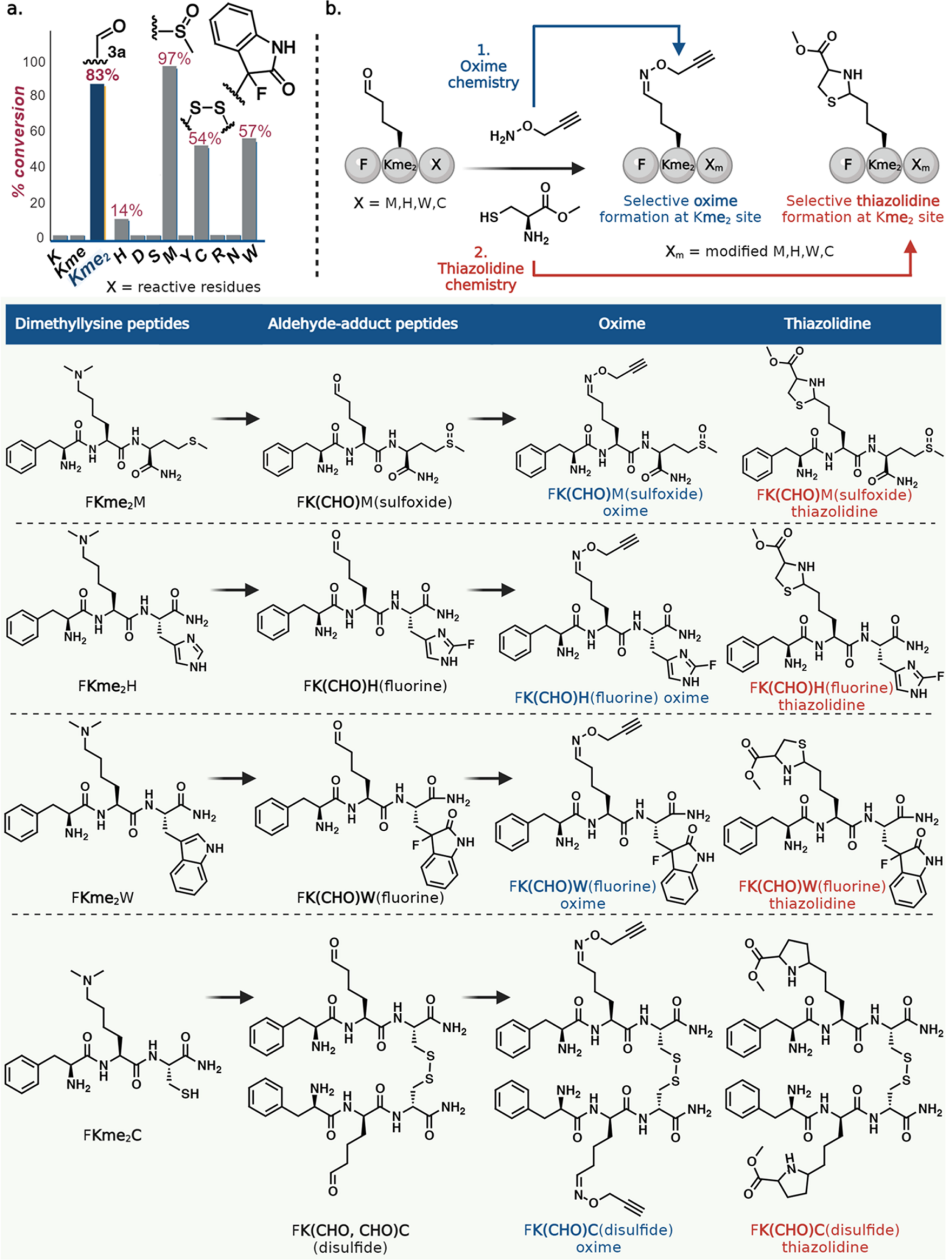
Evaluation of chemoselectivity of the TACO reaction. (a) Chemoselectivity studies of the TACO reaction with varying tripeptides FXV, where X = K, Kme, Kme_2_, H, D, S, M, Y, C, R, N, and W. Only the Kme_2_ residue modified to an aldehyde under the reaction conditions. Side adducts were observed with histidine (fluorination), methionine (sulfoxide), tryptophan (fluorination and oxygenation), and cysteine (disulfide). (b) Selective derivatization of Kme_2_-generated aldehydes on tripeptides FKme_2_X, where X = M, H, W, and C, in the presence of side adducts on histidine, methionine, cysteine, and tryptophan using oxime and thiazolidine chemistry in NaP buffer. The presence of TACO-generated adducts did not interfere with subsequent modification of dimethyl lysine sites, thus indicating the negligible effect of the adducts on downstream application of TACO for profiling of lysine dimethylome.

Chemoselectivity studies for peptide aldehyde revealed the specificity of the TACO reaction for generating aldehyde products with Kme_2_ only, as demonstrated by varying peptides FXV with different lysine methylation states (where X = K, Kme, and Kme_2_) and reactive amino acids (where X = H, D, S, M, Y, C, R, N, W) (Figures 3a and Supporting Figure 5). Our observations indicated the oxidation of Met and Cys, fluorination of Trp and His, and no formation of quinone and the ipso-fluorinated product with tyrosine under the reaction conditions.^21−24^ To further evaluate the effect of these side adducts on the enrichment of Kme_2_-generated aldehyde sites, we synthesized four dimethyllysine tripeptides with methionine (FKme_2_M), histidine (FKme_2_H), tryptophan (FKme_2_W), and cysteine (FKme_2_C) and subjected them to the optimized TACO reaction conditions. The reactions generated the corresponding aldehyde adducts, FK(CHO)M(sulfoxide), FK(CHO)H(fluorine), FK(CHO)W(fluorine), and FK(CHO)C(disulfide) in high conversions (Figures 3b and Supporting Figure 6). Further, treatment of aldehyde adducts with hydroxylamine and amino-thiol (cysteine methyl ester) generated near-quantitative conversions to the oxime and thiazolidine products. These data strongly validate the negligible effects of TACO-generated adducts on downstream chemoproteomics application on Kme_2_ in complex mixtures (Figures 3b and Supporting Figure 6).

### Application of TACO for Diversification of Dimethyllysine on Histone Peptides

**Figure 4:**
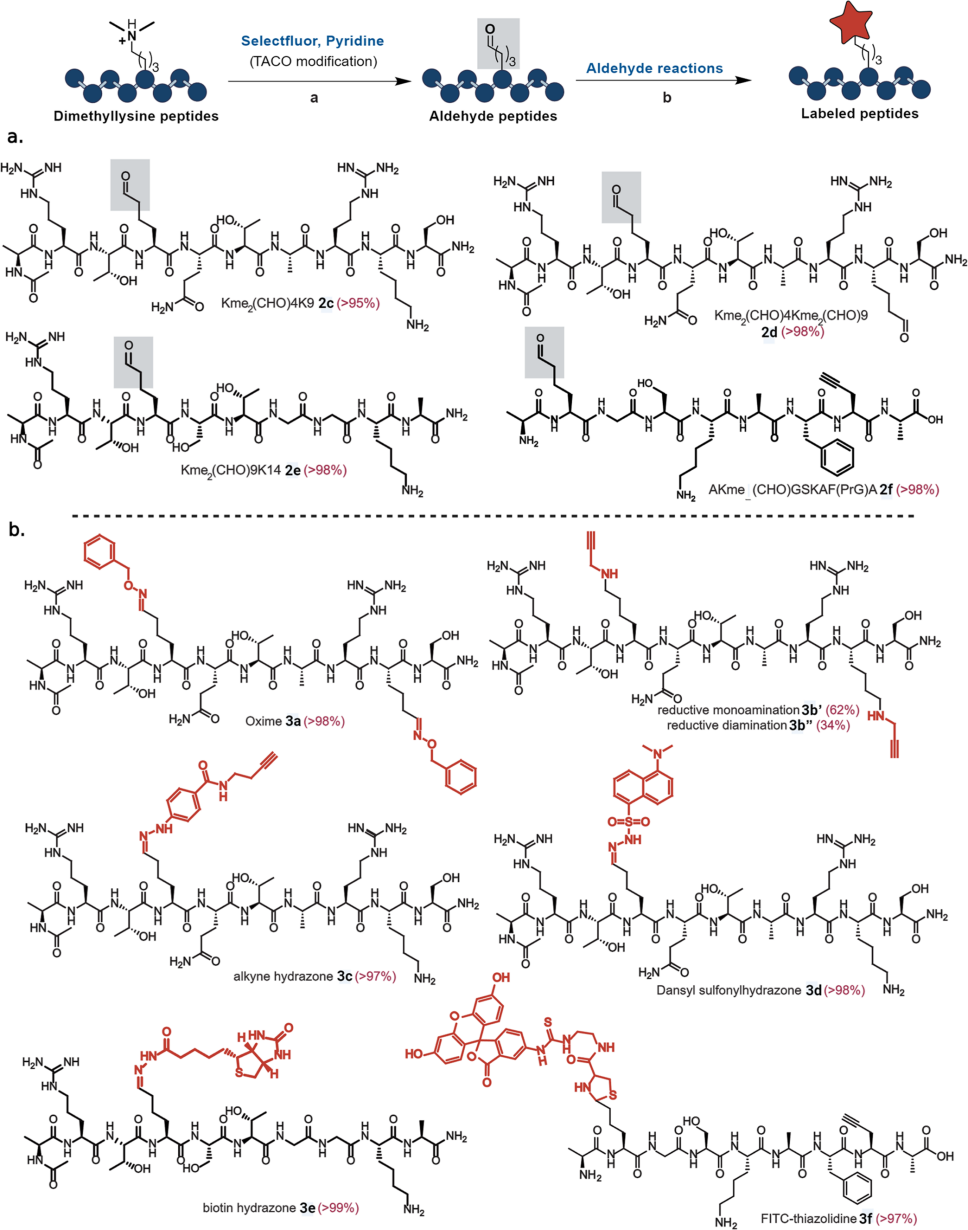
Application of the TACO reaction for Kme_2_ and further derivitization with varying payloads. (a) TACO-mediated modification of histone H3.3 peptide (1c–1f) fragments with Kme_2_ to aldehydes (2c–2f). Reaction conditions: To 6–10 mM of dimethyllysine peptide in 300 μL of 10 mM sodium phosphate buffer (NaP, pH 7.0) were added pyridine (5 equiv) and Selectfluor (2 equiv) and the reaction mixture was stirred for 1 h at room temperature. (b) Late-stage diversification of peptide aldehydes with varying chemistries and payloads.

To demonstrate the application of TACO for diversification of histone peptides, we conducted reactions on various peptides of different sizes and amino acid compositions, including histone H3.3 peptide fragments frequently methylated at K4, K9, K27, and K36, known to regulate biological processes and implicated in disease states.^25−27^ Using solid-phase peptide synthesis, we synthesized the H3.3 peptide fragments Kme_2_4K9 (ARTKme_2_QTARKS) **1c**, Kme_2_4Kme_2_9 (ARTKme_2_QTARKme_2_S) **1d**, Kme_2_9K14 (ARTKme_2_STGGKA) **1e**, propargyl amine containing peptide AKme_2_(CHO)GSKAF(PrG)A (where PrG = propargyl glycine) **1f**, and a negative control peptide K4K9 (ARTKSTGGKA) **1g**. Under optimized conditions, all Kme_2_-containing peptides underwent modification to aldehyde peptide products (Figures 4a and Supporting Figure 7). Intriguingly, the H3.3 peptide fragment Kme_2_4Kme_2_9 (ARTKme_2_QTARKme_2_S) **1d**, containing two Kme_2_, produced the double aldehyde product ARTKme_2_(CHO)QTARKme_2_(CHO)S **2d** with >98% conversion (Supporting Figure 7). Conversely, the negative control peptide K4K9, ARTKSTGGKA **1g** without Kme_2_, remained unmodified (Figures 4a and Supporting Figure 7). These results unequivocally confirmed the application of TACO toward dimethyllysine Kme_2_, marking the first chemical method developed for Kme_2_ modification.

We further diversified peptide aldehydes using aldehyde-specific reactions, such as oxime, hydrazone, thiazolidine chemistry, and reductive amination (Figure 4b). For instance, the histone peptide ARTKme_2_(CHO)QTARKme_2_(CHO)S **2d** with double aldehyde groups underwent modifications with benzylhydroxylamine, generating the oxime product **3a** in >98% yield (Figures 4b and Supporting Figure 8). Additionally, this double-aldehyde peptide was treated with propragylamine and sodium cyanoborohydride, resulting in reductive amination products, single modification **3b’** (62%) and double modification **3b″** (34%) in high conversions (96%) (Figures 4b and Supporting Figure 9).

Next, we functionalized Kme_2_4K9-aldehyde (ARTKme_2_(CHO)QTARKS) **2c** with alkyne- and dansyl-tagged hydrazines to generate hydrazone products **3c** and **3d** in >97% and >98% conversions, respectively (Figure 4b and Supporting Figures 10 and 11). Biotin hydrazine treatment of the peptide aldehyde (ARTKme_2_(CHO)STGGKA) **2e** resulted in 99% conversion to biotin peptide hydrazone **3e** (Figures 4b and Supporting Figure 12). Concurrently, introducing both an affinity handle and a fluorescent dye into a peptide (AKme_2_(CHO)GSKAF(PrG)A, where PrG = propargyl glycine) **2f** via derivatization with FITC-cysteine yielded the thiazolidine conjugation product **3f** in >97% conversion (Figures 4b and Supporting Figure 13). Moreover, treatment of peptide aldehyde FKme_2_(CHO)V **2a** with cysteine resulted in thiazolidine conjugation with high conversion (>99%, Supporting Figure 13). These outcomes underscore the broad scope and efficiency of TACO chemistry in selectively modifying and diversifying Kme_2_-containing peptides with various payloads.

### Selective Labeling of Kme_2_ Peptides by TACO in a Complex Cell Lysate Mixture

**Figure 5:**
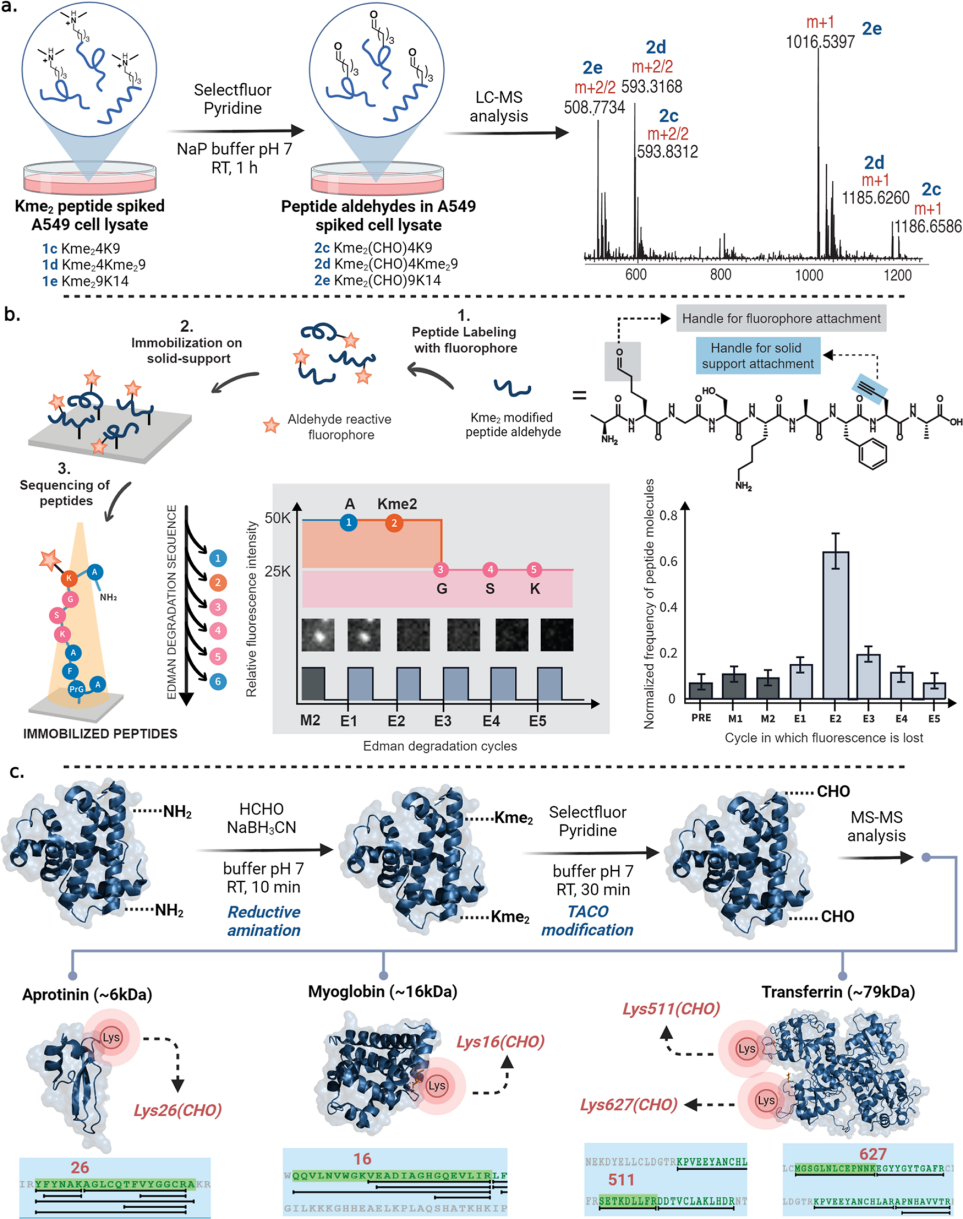
Labeling and identification of Kme_2_ peptides and proteins in a complex mixture. (a) Selective modification of Kme_2_ peptides in a complex mixture of A549 lung cancer cell lysate to peptide aldehydes in a quantitative manner. Reaction conditions: To 100 μg of A549 whole cell lysate in 200 μL of NaP buffer pH 7.0 was added 0.1 mg of each histone peptide followed by addition of pyridine (30 equiv with respect to peptides) and selectfluor (10 equiv with respect to peptides). The reaction mixture was stirred for 1 h. (b) Application of TACO in the identification of Kme_2_ sites on a peptide by single molecule fluorosequencing. The left panel provides an exemplary view of the single fluorescent peptide molecule through cycles of Edman degradation. The right panel indicates the normalized counts of the labeled Kme_2_ peptide where its fluorescence intensity was lost. (c) Selective modification of chemically generated Kme_2_ proteins by TACO chemistry followed by the identification of Kme_2_ sites on proteins by LC-MS/MS analysis.

To evaluate the robustness of our TACO technique in labeling Kme_2_ peptides within a complex mixture, we spiked cell lysates from epithelial carcinoma A549 and H1792 lung cancer cell lines (100 μg) with three different histone H3.3 peptides (0.1 mg each): Kme_2_4K9 (ARTKme_2_QTARKS) **1c**, Kme_2_4Kme_2_9 (ARTKme_2_QTARKme_2_S) **1d**, and Kme_2_9K14 (ARTKme_2_STGGKA) **1e** and **1c**, **1d**, and **1g**, respectively. Both complex mixtures were incubated with Selectfluor and pyridine for 1 h, followed by LC-MS analysis (Figure 5a, A549 Supporting Figures 14 and 15). All peptides containing Kme_2_ (**1c**–**1e**) were successfully converted to the corresponding peptide aldehydes (**2c**–**2e**) without any detectable traces of the unmodified starting peptides in the complex cell lysate mixtures (Figure 5a, A549 Supporting Figures 14 and 15). Notably, peptide **1g**, K4K9 (ARTKSTGGKA), lacking Kme_2_, showed no modification in the H1792 cell lysate (Supporting Figure 15).

### Identification of Kme_2_ Sites by TACO at a Single-Molecule Level Using Fluorosequencing

To determine the site of Kme_2_ in a peptide at a single-molecule level, we modified a model peptide, AKme_2_GSKAF(PrG)A (where PrG = propargyl glycine), by TACO chemistry to peptide aldehyde, AKme_2_(CHO)GSKAF(PrG)A. Next, we functionalized the Kme_2_-aldehyde group with an Atto647N fluorophore by using dithiolane chemistry under acidic conditions and subjected it to a fluorosequencing workflow including immobilization on the azide-functionalized microscopic slide using PrG on a peptide, AKme_2_^Atto647N^GSKAF(PrG)A, by click chemistry (Figures 5b and Supporting Figure 16). In the case of biologically derived peptides, the C-terminus will be used for immobilization using a photoredox catalyst that can differentiate oxidation potential of the C-terminus from the side chains of Asp and Glu, leading to C-terminal immobilization exclusively.^28^ Next, the fluorophore-labeled immobilized peptide was subjected to several rounds of Edman’s degradation including one prerun and two rounds of mock Edman’s degradation (M1-M2) with all the reagents except phenylisothiocyanate followed by analysis using a total internal reflection fluorescence (TIRF) microscope (Figure 5b).^29^ No decrease in the fluorescence was observed under the mock conditions, suggesting the high stability of the Kme_2_-modified fluorophore under Edman’s degradation conditions requiring TFA and pyridine. Next, Edman’s degradation was performed on a peptide with phenylisothiocyanate followed by the analysis of fluorescence by TIRF after each round. A significant decrease in the fluorescence was observed after the second round of Edman’s degradation, confirming the site of Kme_2_ at a single-molecule level. Subsequent rounds of Edman’s degradation did not lead to a change in the fluorescence. There are no other chemical methods for the identification of sites of Kme_2_ PTM by any other single-molecule protein sequencing SMPS techniques.^30^ Future studies will be focused on discovering proteins containing Kme_2_ and the sites of Kme_2_ on a proteome-wide scale by fluorosequencing. The work under this direction is currently underway in our laboratory.

### Modification of Proteins and Whole Cell Lysate by TACO

**Figure 6:**
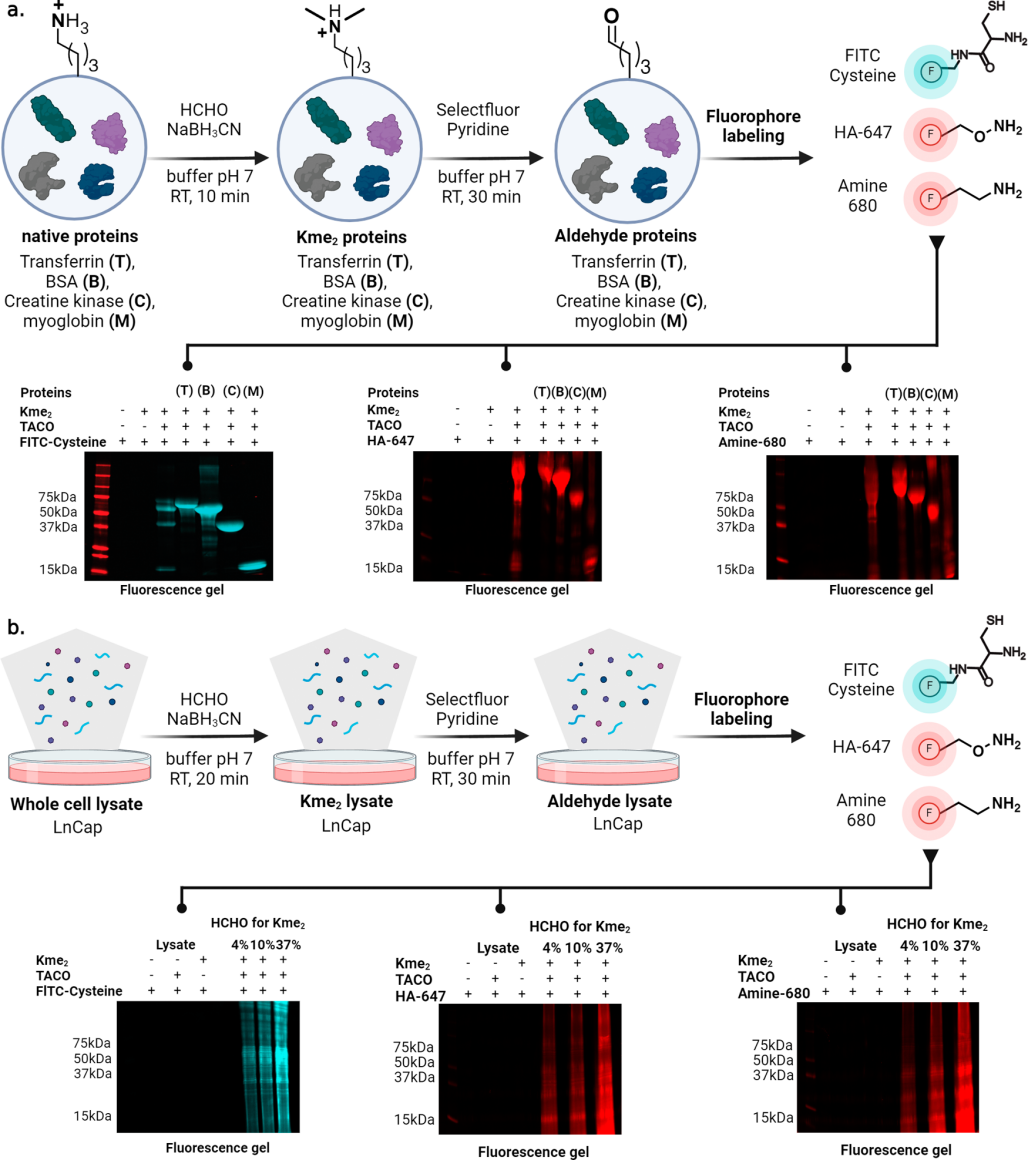
Selective labeling of Kme_2_ proteins by TACO chemistry using varying fluorophores. (a) Selective modification of chemically generated Kme_2_ proteins to aldehyde proteins by TACO chemistry followed by labeling with varying fluorophores using different aldehyde chemistries as analyzed by running SDS PAGE. No fluorophore labeling was observed without the TACO reaction. (b) Selective modification of chemically generated Kme_2_ proteins in cell lysate to aldehyde proteins by TACO followed by fluorophore labeling and in-gel analysis. High fluorescence was observed in cell lysate in a dose-dependent manner, indicating high amounts of Kme_2_-proteins. No fluorophore labeling was observed on cell lysate without the TACO reaction or without Kme_2_-modified cell lysate.

To demonstrate the compatibility of TACO chemistry with proteins, we chemically modified lysines (K) to dimethyl lysine (Kme_2_) on proteins of varying molecular weights, aprotinin (A, 5 kDa), myoglobin (M, 16 kDa), and transferrin (T, 79 kDa) using reductive amination. Proteins were treated with a solution of formaldehyde and sodium cyanoborohydride in citrate buffer for 10 min. Subsequently, Kme_2_ proteins were subjected to TACO chemistry to generate aldehyde proteins. The MS/MS analysis of digested aldehyde proteins identified K26 (aprotinin), K16 (myoglobin), K511 (transferrin), and K627 (transferrin) as Kme_2_ modification sites (Figures 5c and Supporting Figure 17). Moreover, four proteins of varying molecular weights and 3 D structures, transferrin (T), BSA (B), creatine kinase (C), and myoglobin (M), were subjected to reductive amination followed by TACO chemistry to produce aldehyde proteins. These aldehyde proteins were incubated with various fluorophores containing diverse reactive aldehyde functional groups (FITC-cysteine, hydroxylamine (HA)-647 and amine-680) and analyzed via in-gel fluorescence (Figure 6a). The results showed the selective labeling with aldehyde proteins only (lanes 3–7, lane 3 = mixture of all four proteins, Figures 6a and Supporting Figure 18). No fluorophore labeling was observed with native proteins and Kme_2_ proteins (lanes 1, 2, Figures 6a and Supporting Figure 18). This result demonstrated the ability of TACO chemistry to selectively modify Kme_2_ sites on proteins to aldehydes, which can then be further modified with varying payloads.

To further demonstrate the robustness of TACO chemistry to modify proteins within cell lysates, we treated prostate cancer cell lysate (LnCap) with reductive amination reagents (formaldehyde and sodium cyanoborohydride) in a dose-dependent manner (4, 10, and 37%), followed by TACO chemistry and subsequent labeling with FITC-cysteine, hydroxylamine (HA)-647, and amine-680 fluorophores (Figures 6b and Supporting Figure 19). Analysis and quantification of in-gel fluorescence results clearly showed selective fluorophore labeling of aldehyde-modified cell lysate and a dose-dependent increase in the fluorescence intensity from 4 to 37% of reductive amination reagents (with Cys-FITC, 37% lane = 1.0, 10% lane = 0.582, 4% lane = 0.420; with HA-647, 37% lane = 1.0, 10% lane = 0.513, 4% lane = 0.459; with amine-680, 37% lane = 1.0, 10% lane = 0.672, 4% lane = 0.373) (lanes 4–6, Figures 6b and Supporting Figure 19). No fluorescence was observed with untreated LnCap cell lysate, Kme_2_-modified cell lysate, and TACO-treated cell lysate without Kme_2_ modification (lanes 1–3, Figure 6b and Supporting Figure 19), confirming the high selectivity of TACO chemistry for Kme_2_.

### Selective Enrichment of Kme_2_ Proteins from Nucleosomes by TACO

To explore the capability of TACO chemistry in the selective modification and enrichment of naturally occurring Kme_2_ on histones, we exposed nucleosomes from prostate cancer cell lysate (LnCap) to Selectfluor and pyridine for 1 h, followed by the enrichment of aldehyde proteins via oxime chemistry using hydroxylamine-functionalized resin in pull-down experiments. The resin was thoroughly washed with solvents to remove noncovalently bound proteins (filtrate). Subsequently, enriched proteins were released from the resin under acidic conditions (95% TFA in water).

**Figure 7:**
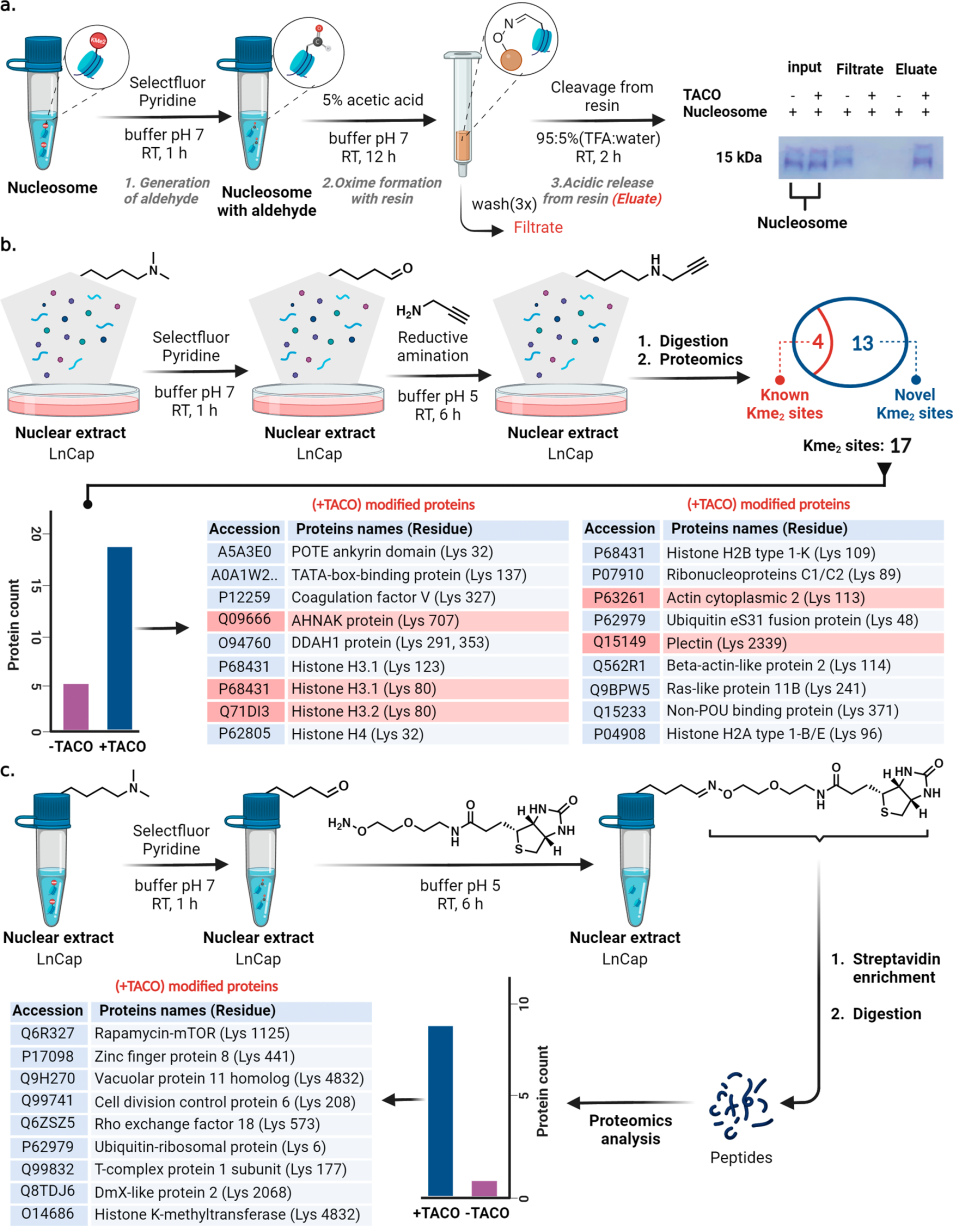
Discovery of naturally occurring Kme_2_ proteins and Kme_2_-sites from a complex cell lysate was carried out by TACO chemistry. (a) Selective modification of naturally occurring dimethyl lysine in nucleosomes using TACO chemistry followed by the enrichment of Kme_2_-generated aldehyde proteins using hydroxylamine resin and release of trapped proteins under acidic conditions. The filtrate contains proteins remaining after the enrichment process with and without TACO chemistry on nucleosomes, while the eluate comprises proteins enriched from the nucleosomes with and without TACO chemistry using a solid support. The filtrate and eluate were analyzed by SDS PAGE analysis. The gels showed the enrichment of Kme_2_ proteins from nucleosomes under TACO reaction conditions. (b) Selective modification of Kme_2_ proteins by TACO chemistry followed by trapping of Kme_2_-generated aldehyde proteins by reductive amination and proteomic analysis. Twelve novel Kme_2_ proteins and thirteen new Kme_2_ sites were identified (novel = blue, known = red). In the negative control, five proteins were identified, which vary from the TACO-treated samples due to the presence of natural occurring allysine on proteins. (c) Enrichment of TACO-modified Kme_2_ proteins followed by proteomic analysis. Nine low-abundance Kme_2_ proteins were discovered with nine new Kme_2_ sites.

SDS-PAGE analysis of the cell lysate (filtrate) postenrichment and protein release from the solid support (eluate) distinctly demonstrated the efficient capture and release of Kme_2_-modified protein aldehydes (Figure 7a, lanes 4 and 6, Supporting Figures 20 and 21). Repetition of this reaction in duplicate showed consistent gel profiles across all replicates, confirming the reproducibility, selectivity, and robustness of the TACO approach in tagging and enriching naturally occurring Kme_2_ proteins from a complex nucleosome mixture. Enrichment of nucleosomes by hydroxylamine resin without the TACO reaction failed to capture proteins (lane 3 (filtrate) and lane 5 (eluate), Figure 7a). Lanes 1 and 2 represent total nucleosomes with and without TACO reagents, respectively (Figure 7a).

### Identification and Discovery of Kme_2_ Proteins and Kme_2_ Sites by TACO from a Complex Cell Lysate

To demonstrate an exciting application of TACO chemistry for identifying and discovering naturally occurring Kme_2_ proteins, we treated nuclear extracts obtained from LnCap cells with TACO chemistry followed by labeling of Kme_2_-generated aldehyde proteins with propargylamine using reductive amination. We performed this reaction to increase the stability of the adducts under proteomics conditions and to add a tag for identification. By digesting and analyzing the cellular mixture via proteomics, we discovered 13 Kme_2_ sites that had not been previously identified, along with four Kme_2_-sites previously reported by other methods^31^ (Figures 7b and Supporting Figure 22). The treatment of nuclear extract of MCF-10A, a preadenocarcinoma cell, with TACO chemistry led to the identification of nine Kme_2_ sites with one known Kme_2_ site, K240 of actin, α cardiac muscle 1 (Supporting Figure 22). Notably, none of the identified Kme_2_ proteins in MCF-10A cells match with Kme_2_ proteins observed in LnCap cancer cells, thus suggesting the potential of this approach in discovering dimethyllysine biomarkers. Moreover, proteomics analysis of nuclear extracts without TACO modification (negative control) identified five proteins and sites, differing from those observed in the TACO-treated sample. We attribute this modification to the presence of allysine, an endogenous aerobic oxidation aldehyde product of lysine.^32^

Furthermore, we developed an enrichment protocol for trapping Kme_2_-generated aldehyde proteins obtained through TACO chemistry to identify low-abundant Kme_2_ proteins from a complex nuclear extract mixture of LnCap cells. Employing biotin-derivatized alkoxyamine probes to label aldehyde proteins, followed by streptavidin enrichment and digestion of enriched proteins, led to the discovery of nine novel low-abundant Kme_2_ proteins and identification of nine new Kme_2_ sites (Figures 7c and Supporting Figure 23). Taken together, these results demonstrate the potential of TACO chemistry to serve as a chemical platform for the identification and discovery of the lysine dimethylome.

## Conclusions

In summary, we have developed TACO, a versatile chemical method for modifying dimethyllysine Kme_2_ to an aldehyde group within peptides, proteins, and cell lysates under physiological conditions. The TACO chemistry involves the oxidation of dimethyl lysine/Kme_2_ by Selectfluor, followed by hydrolysis of the resulting iminium ions to generate aldehydes. Our study demonstrates the robustness of TACO chemistry for selectively labeling of Kme_2_-containing peptides, proteins, and cell lysates with diverse payloads, including affinity tags such as alkyne, biotin, and various fluorophores, by diverse aldehyde chemistries in high conversions (>90%). Moreover, we showcased the compatibility of TACO chemistry with fluorosequencing, thus demonstrating the potential of identifying Kme_2_ sites at a single-molecule level. Applying TACO chemistry to the nuclear extract of cancer and preadenocarcinoma cells, we discovered 30 novel Kme_2_ sites, in addition to 5 previously reported sites using MS/MS analysis. This demonstrates the capability of TACO chemistry to enable the identification of Kme_2_ proteins and their sites on a proteome-wide scale, through either MS-based proteomics or single molecule fluorosequencing. We compared proteome profiling of diseased cells to healthy controls, and none of the identified Kme_2_ proteins in MCF-10A cells match with Kme_2_ proteins observed in LnCap cancer cells, thus suggesting the potential of this approach in discovering Kme_2_ proteins and Kme_2_ sites as disease biomarkers. The work in these directions is currently underway in our laboratory. We believe that our chemical approach will complement existing detection methods, significantly expanding the chemical toolbox available for epigenetics research.

## Supporting Information Available

List of proteins (TACO) (XLSX)
Optimization of the TACO reaction with varying oxidizing reagents and nucleophiles; reaction procedures; procedure of TACO reaction with peptides; proteins; cell lysates; nucleosomes; fluorosequencing; enrichment protocol; proteomic analysis and product characterization by NMR; HPLC; HRMS and computational data; and raw images for fluorosequencing uploaded to Zenodo (PDF)

## Notes

B.E. and M.R. are the inventors of a United States patent application (PCT/US2022/076782) of Emory University covering the methodology of the TACO reaction for modification of Kme_2_ and its application in global profiling and identification of Kme_2_ sites from a complex biological system. J.S. and E.V.A. are cofounders and shareholders of Erisyon, Inc. E.V.A. serves on the scientific advisory board of Erisyon.

## Abbreviations

Kme_2_: lysine dimethylation
PTM: post-translational modification
TACO: tertiary amine coupling by oxidation
Kme_1_: monomethyl lysine
MS: mass spectrometry
TIRF: total internal reflection fluorescence
MBD: methyl-binding domains
PrG: propargyl glycine
DEAD: diethyl azodicarboxylate
