# Supplementary material for: Tertiary Amine Coupling by Oxidation for Selective Labeling of Dimethyl Lysine Post-Translational Modifications
Source: J Am Chem Soc. 2024 Apr 8;146(15):10621–31. doi: 10.1021/jacs.4c00253 (PMC11027136; doi:10.1021/jacs.4c00253)
Supplement: Supplementary file 2 — ja4c00253_si_002.pdf [file ja4c00253_si_002.pdf]

Supporting Information

Tertiary Amine Coupling by Oxidation for Selective Labeling of Dimethyl Lysine Posttranslational Modifications

Benjamin Emenike[1], Patrick Czabala[1], Jonathan Farhi[2], Jagannath Swaminathan[3], Eric V Anslyn[3], Jennifer Spangle[2], Monika Raj[1*]

Table of content **Pages**

**I.**	General	2
**II.**	Materials	2
**III.**	Purification	2
**IV.**	Analytical Methods	2-3
**V.**	Fmoc Solid-Phase Peptide Synthesis	3
**VI.**	General procedure 1 for oxidative transformation of Kme_2 peptides	3
**VII.**	Figure 1. Evaluation of oxidizing agents	4-6
**VIII.**	Figure 2. Proposed mechanism for TACO	6
**IX.**	Figure 3. Evaluation of bases	7-12
**X.**	Figure 4. Characterization of aldehyde products	13-15
**XI.**	Figure 5. Chemoselectivity studies	15-27
**XII.**	Figure 6. Effect of adducts on enrichment of Kme_2-generated aldehyde	28-42
**XIII.**	Figure 7. Pan-specificity evaluation of TACO	42-50
**XIV.**	Figure 8. Oxime chemistry on histone aldehyde peptides	51-52
**XV.**	Figure 9. Reductive amination of histone aldehyde peptides	52-54
**XVI.**	Figure 10. Hydrazone chemistry on histone aldehyde peptides	54-55
**XVII.**	Figure 11. Dansyl hydrazone with peptide aldehydes	56-57
**XVIII.**	Figure 12. Biotin hydrazone with peptide aldehydes	57-58
**XIX.**	Figure 13. Thiazolidine formation using histone aldehyde peptides	58-61
**XX.**	Figure 14. Modification of histone peptide spiked cancer cell lysates A549	62-63
**XXI.**	Figure 15. Modification of histone peptide spiked cancer cell lysates H1792	63-64
**XXII.**	Figure 16. Single molecule protein sequencing	64-67
**XXIII.**	Figure 17. Modification of Kme_2 proteins using TACO	67-75
**XXIV.**	Figure 18. Modification of Kme_2 proteins with fluorophore	75-82
**XXV.**	Figure 19. Modification of Kme_2 proteins in cell lysate	82-84
**XXVI.**	Figure 20. Solid-support enrichment of peptide aldehydes	84-85
**XXVII.**	Figure 21. Solid-support enrichment of nucleosomes from prostate cancer cells	85-86
**XXVIII.**	Figure 22. TACO modification of nuclear extract and proteomics analysis	86-104
**XXIX.**	Figure 23. Biotin enrichment of TACO modified Kme_2 sites	105-111
**XXX.**	References	111

I. General. All commercial materials (Sigma-Aldrich, Fluka and Novabiochem) were used without further purification. All solvents were reagent or HPLC (Fisher) grade. All reactions were performed under air in glass vials. Yields refer to chromatographically pure compounds; % yields were obtained by comparing HPLC peak areas of products and starting materials. HPLC and MS were used to monitor reaction progress, and product elucidation was done using MS and NMR.

II. Materials. Fmoc-amino acids, Rink amide resin, 3-[bis(dimethylamino)methyliumyl]-3H-benzotriazol-1-oxide hexafluorophosphate (HBTU), 1-hydroxy-7-azabenzotriazole (HOAt), N,N′-iisopropylcarbodiimide(DIC), and N,N-diisopropylethylamine (DIEA) were obtained from CreoSalus (Louisville, Kentucky). Piperidine, trifluoroacetic acid (TFA), were obtained from Alfa Aesar (Ward Hill, Massachusetts). N,Ndimethylformamide (DMF), dichloromethane (DCM), methanol (MeOH), acetonitrile (ACN), were obtained from VWR (100 Matsonford Road Radnor, Pennsylvania). Selectfluor (1-Chloromethyl-4-fluoro-1,4-diazoniabicyclo[2.2.2]octane bis(tetrafluoroborate)), 4-Dimethylaminopyridine and pyridine were obtained from Sigma.

III. Purification. HPLC: Purification of peptide starting materials was performed using high performance liquid chromatography (HPLC) on an Agilent 1100 series HPLC equipped with a C-18 reverse phase column with a particle size of 5 μm. All separations involved a mobile phase of water (solvent A) and acetonitrile (solvent B). The HPLC method used a linear gradient of 0-80% solvent B over 30 minutes at ambient temperature with a flow rate of 1 mL min^{-1}. The eluent was monitored by absorbance at 220 nm.

IV. Instrumentation and sample analysis. NMR. ^1H and ^{13}C spectra were acquired at 25 °C in DMSO-d_6, $CDCl_3$ using an Agilent DD2 (600 MHz) spectrometer with a 3-mm He triple resonance (HCN) cryoprobe. All ^1H NMR chemical shifts (δ) were referenced relative to the residual DMSO-d_6 peak at 2.50 ppm, $CDCl_3$ peak at 7.26 ppm or internal tetramethylsilane (TMS) at 0.00 ppm. ^{13}C NMR chemical shifts were referenced to DMSO-d_6 at 39.52 ppm and $CDCl_3$ at 77.2 ppm. ^{13}C NMR spectra were proton decoupled. NMR spectral data are reported as chemical shift (multiplicity, coupling constants (*J*), integration). Multiplicity is reported as follows: singlet (s), broad singlet (br s), doublet (d), doublet of doublets (dd), doublet of triplets (td), triplet (t) and multiplet (m). Coupling constant (J) in hertz (Hz).

Analytical HPLC. Analytical HPLC chromatography (HPLC) was performed on an Agilent 1200 series HPLC equipped with a 5 mm C-18 reversed-phase column. The reaction was monitored by analytical reverse phase HPLC using a gradient of water versus acetonitrile. All separations involved mobile phase with 0.1 % formic acid in water (solvent A) and 0.1 % formic acid in acetonitrile (solvent B). Analytical HPLC method used for purification of peptides a linear gradient of 0-80% solvent B over 30 min at room temperature with a flow rate of 1.0 mL min^{-1}. The oxidative tertiary amine reactions were analyzed by HPLC, and MS. HPLC was carried out with 0.1% formic acid: water (solvent A): acetonitrile (solvent B) at detection wavelength 220 nm. HPLC **METHOD A:** Gradient: 0 to 80 % **B** (0.1% formic acid in ACN) in 30 min; 80-100 % B in 31-35 min at a flow rate of 1 mL/min.
HPLC METHOD B: Gradient: 0 to 50 % **B** (0.1% formic acid in ACN) in 30 min; 50-100 % B in 31-35 min at a flow rate of 0.5 mL/min.

LC/MS. High resolution LC-MS conditions for all purified peptides: Analyses were performed on an ultraperformance LC system (ACQUITY, Waters Corp., USA) coupled with a quadrupole

time-of-flight mass spectrometer (Q-ToF Premier, Waters) with electrospray ionization (ESI) in positive mode using Mass lynx software (V4.1) or high-performance LC system (Agilent, 1100 series) coupled with triple quadrupole.

LC-MS (Agilent technologies 6460) with electrospray ionization (ESI) in positive mode using Agilent mass hunter (10.0). Unless otherwise mentioned a sample was injected either onto a C4 column (Phenomenex Aeris™ 3.6 µm WIDEPORE C4 200 Å, LC Column 50 x 2.1 mm) with a 400 µL/min flow rate of mobile phase of solution A (90 % H_2O, 10 % acetonitrile and 0.1 % formic acid (FA)) and solution B (95 % acetonitrile, 5 % H_2O, and 0.1 % formic acid) beginning gradient- Time- 0 min 10 % B; 5 min 28 % B; 20 min 38 % B; 22 min 90 % B; C18 column (ACQUITY UPLC BEH 1.7 µm 1x 50 mm) with a 200 µL/min flow rate of mobile phase of solution A (90 % H_2O, 10 % acetonitrile and 0.1 % formic acid) and solution B (90 % acetonitrile, 10 % H_2O, and 0.1 % formic acid) beginning gradient- Time- 1 min 0% B; 1-10 min 100% B for chromatography analysis (or) directly injected with mobile phase 90 % H_2O: 10 % ACN, 0.1% formic acid at 400 µL/min flow rate in ESI positive mode. HPLC and MS of the cyclized products were performed without the addition of formic acid to the eluting solvent because the triazene compound is acid labile.

HRMS. High resolution MS data were acquired on Thermo Exactive Plus using a heated electrospray source. The solution was infused at a rate of 10-25 µL/min/electrospray using 3.3 KV. The typical settings were Capillary temp 320 ^oC. S-lens RF level was between 30-80 with an AGC setting of 1 E6. The maximum injection time was set to 50 ms. Spectra were taken at 140,000 resolutions at m/z 200 using Tune software and analyze with Thermo's Freestyle software.

V. Fmoc Solid-Phase Peptide Synthesis (Fmoc-SPPS).[1] Peptides were synthesized manually on a 0.25 mm scale using Rink amide resin. Resin was swollen with DCM for 1 h at room temperature. Fmoc was deprotected using 20% piperidine–DMF for 5 min to obtain a deprotected peptide-resin. First, Fmoc protected amino acid (1.25 mm/5 equiv.) was coupled using HOAt (1.25 mm/5 equiv.) and DIC (1.25 mm/5 equiv.) in DMF for 15 min at room temperature. Fmoc-protected amino acids (0.75 mm/3 equiv.) were sequentially coupled on the resin using HBTU (0.75 mm/3 equiv.) and DIEA (1.5 mm/6 equiv.) in DMF for 5 min at room temperature. Peptides were synthesized using standard protocols. Peptides were cleaved from the resin using a cocktail of 95:5, trifluoroacetic acid: water for 2 h. The resin was removed by filtration and the resulting solution was concentrated. The residue was diluted with ACN/water mixture. The resulting solution was purified by HPLC.

VI. General procedure 1: Tertiary amine coupling by Oxidation (TACO) of dimethyllysine peptides to generate aldehyde-peptide products.

To 1 mg (6-10mM) of unprotected dimethyllysine peptide dissolved in 300 µL of 10 mM sodium phosphate buffer (NaP, pH 7.0), was added pyridine (5 eq) and selectfluor (2 eq). The reaction mixture was stirred for 1 h and subsequently injected into the HPLC for determining the % conversion of dimethyllysine peptides to the aldehyde peptide product and the mass confirmed with LC-MS. HPLC was carried out with 0.1% formic acid: water (solvent A): acetonitrile (solvent B) at detection wavelength 220 nm.

HPLC METHOD A: HPLC METHOD A: Gradient: 0 to 80 % **B** (0.1% formic acid in ACN) in 30 min; 80-100 % B in 31-35 min at a flow rate of 1 mL/min.
HPLC METHOD B: Gradient: 0 to 50 % **B** (0.1% formic acid in ACN) in 30 min; 50-100 % B in 31-35 min at a flow rate of 0.5 mL/min.
VII. Supplementary Figure 1: Evaluation of oxidizing reagents for conversion of Kme_2 to aldehyde

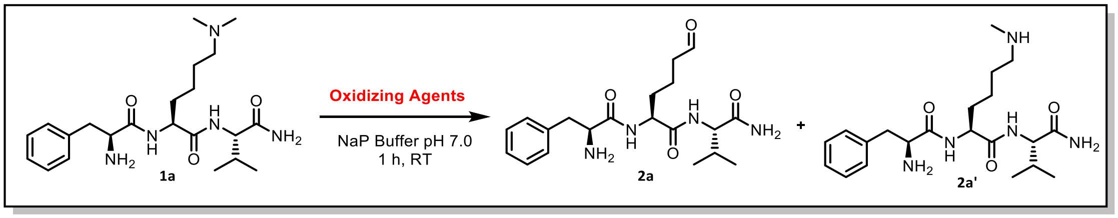

To 1.0 mg (8mM) of $FKme_2V$ peptide **1a** in 300 μL of 10 mM sodium phosphate buffer (NaP, pH 7.0) was added different oxidizing reagents such selectfluor, tropylium, NBS, DEAD, and $FeCl_3$/t-BuOOH (2 eq). The reaction mixture was stirred for 1 h. Samples were taken from the reaction mixture, injected into LC-MS to monitor the generation of aldehyde product $FKme_2$(CHO)V **2a**. The reaction mixture was analyzed by HPLC using method A and the % conversion to aldehyde-peptide product **2a** was determined and compiled in the table below.

$FKme_2V$ linear peptide 1a. LCMS: *m/z* 420.29315 (calcd [M+H]+ = 420.2896), (HPLC analysis at 220 nm). Retention time in HPLC: 4.824

$FKme_2$(CHO)V peptide aldehyde product 2a. LCMS: *m/z* 391.23049 (calcd [M+H]+ = 391.2340), (HPLC analysis at 220 nm). Retention time in HPLC: 7.017

HPLC Trace of aldehyde generation reaction from 1a using selectfluor

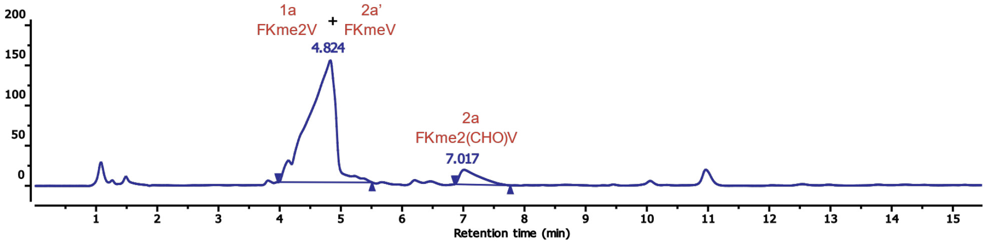

MS-Trace of peak 4.824

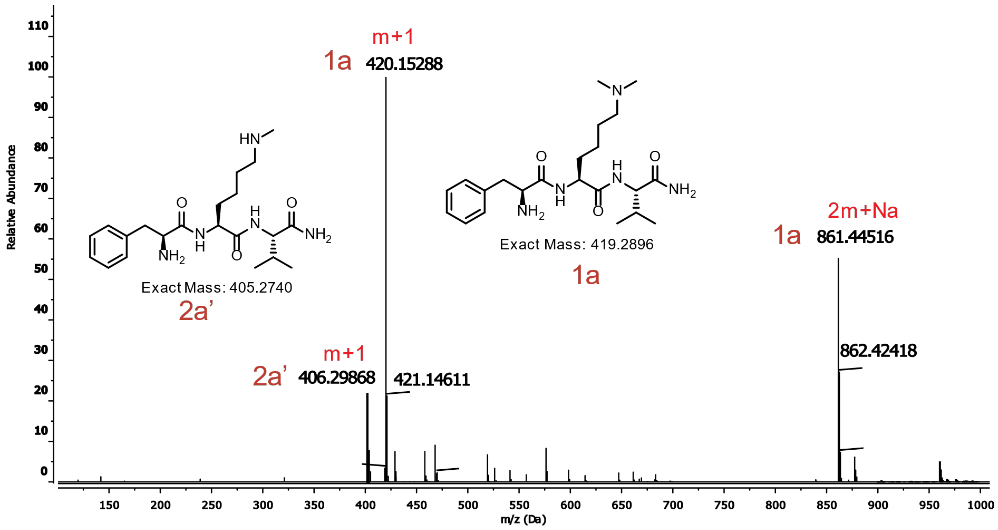

MS-Trace of peak 7.017

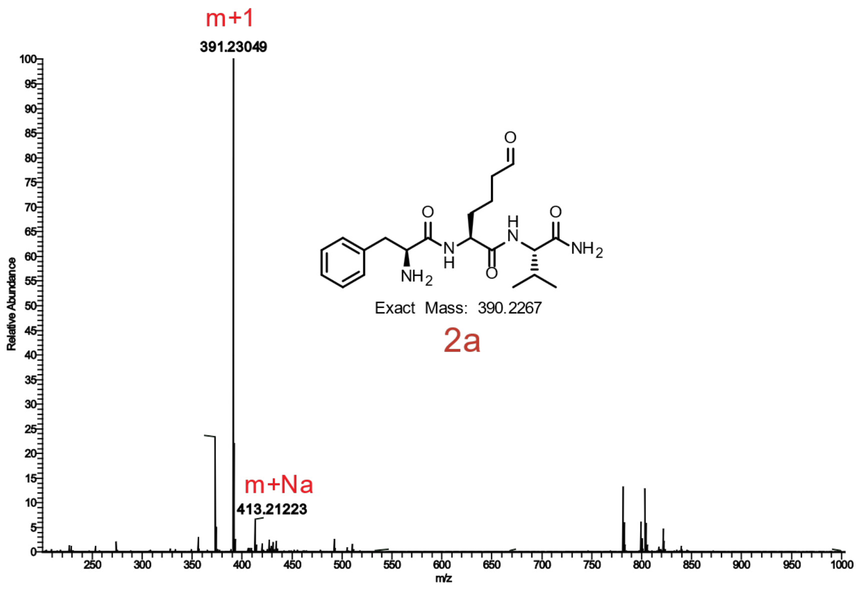

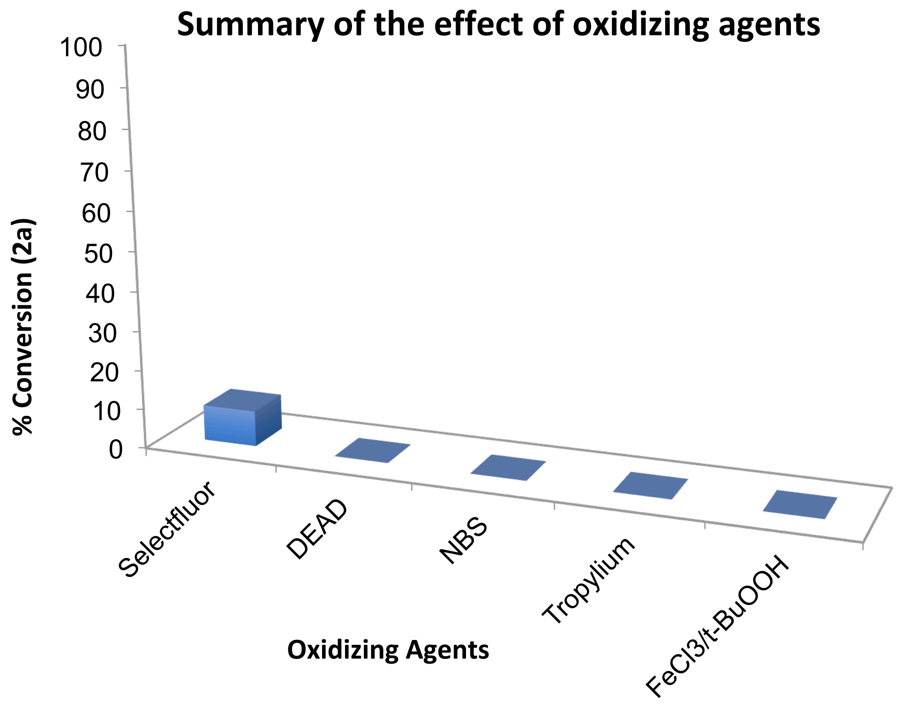

Oxidizing agents	Selectfluor	DEAD	NBS	Tropylium	$FeCl_3$/t-BuOOH
% Conversion (2a)	9	0	0	0	0

VIII. Supplementary Figure 2: Plausible mechanism for the formation of peptide aldehyde and monomethyllysine peptides.

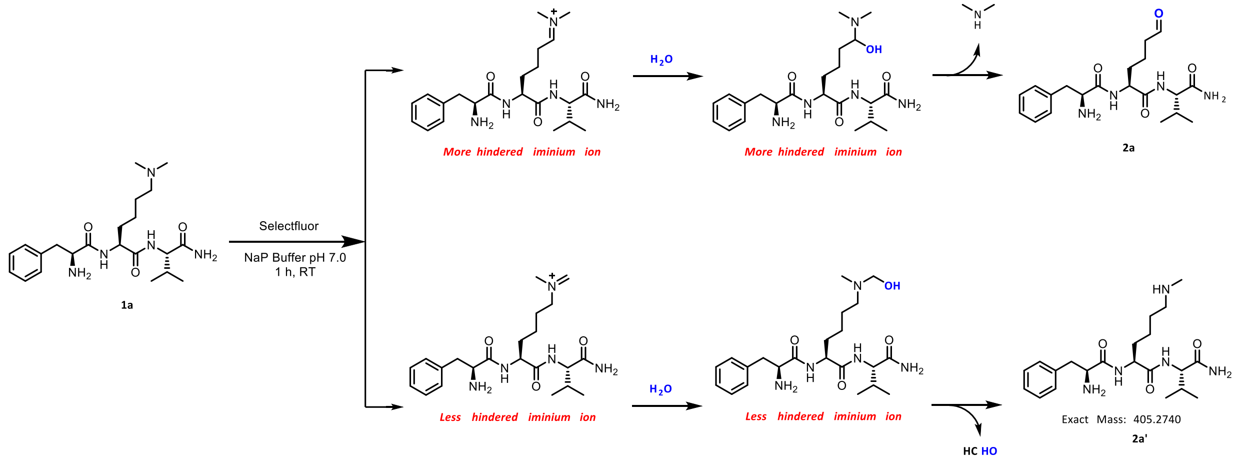

IX. Supplementary Figure 3: Evaluation of various bases for the oxidative tertiary amine transformation of dimethyllysine peptide **1a** to peptide aldehyde **2a**.

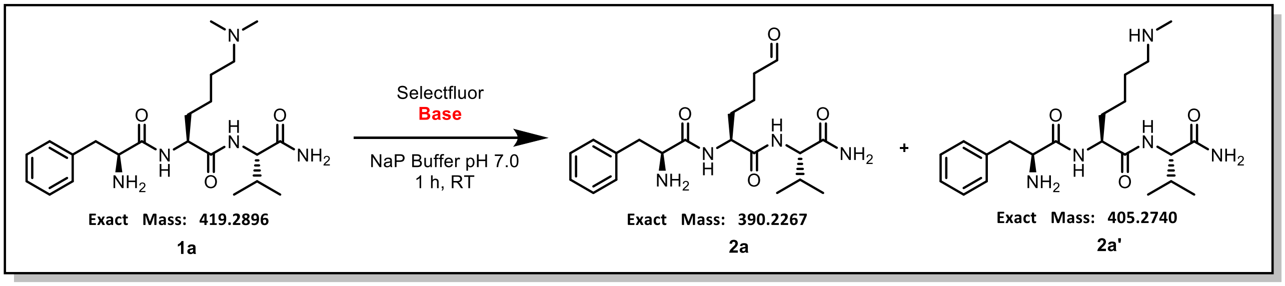

To 1.0 mg of FKme$_2$V **1a** dissolved in 300 μL of 10 mM sodium phosphate buffer (NaP, pH 7.0), was added varying **base** such as pyridine, piperidine, DMAP and proton-sponge (1-5 eq) and selectfluor (2 eq). The reaction mixtures were stirred for 1 h. Samples were taken from the reaction mixture, injected into LC-MS to monitor the generation of peptide aldehyde **2a**. The reaction mixtures were analyzed by HPLC using method A to determine the % conversion to peptide aldehyde **2a** and monomethyllysine peptide **2a'**.

FKme$_2$(CHO)V peptide-aldehyde product 2a. LCMS: *m/z* 391.23049 (calcd $[M+H]^+$ = 391.2340), m/z 413.21223 (calcd [M+Na]+ =413.21), (HPLC analysis at 220 nm). Retention time in HPLC: 6.905

FKmeV monomethyllysine peptide product 2a'. LCMS: *m/z* 406.18372 (calcd $[M+H]^+$ = 391.23049), m/z 811.59185 (calcd [2M+1]+ = 811.15), (HPLC analysis at 220 nm). Retention time in HPLC: 5.259

Evaluation of pyridine:

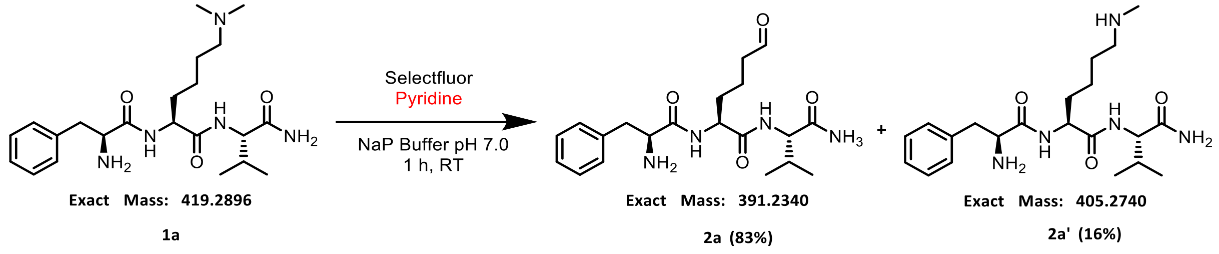

HPLC Trace of aldehyde generation reaction from 1a using pyridine

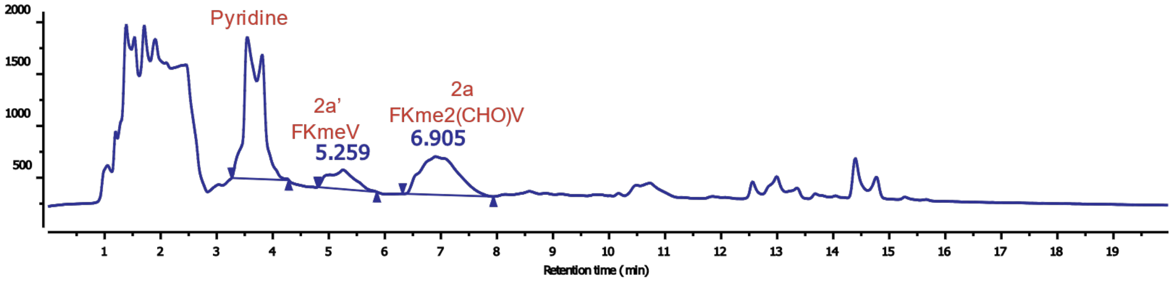

MS-Trace of 2a (Peak 6.905)

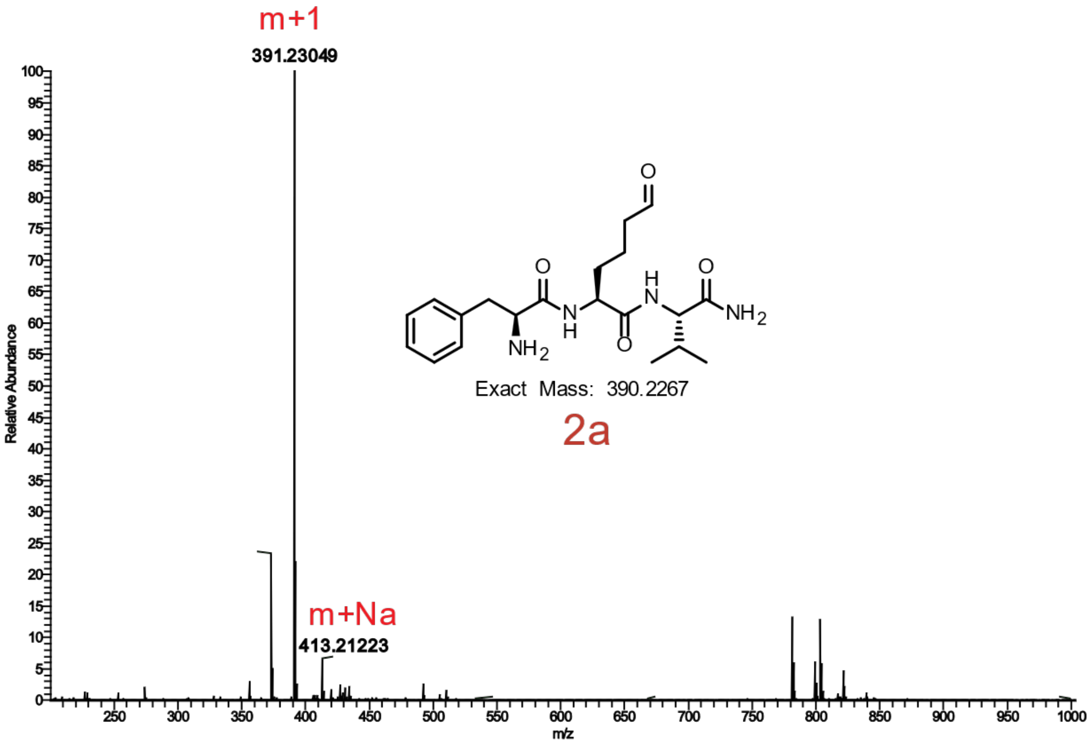

MS-Trace of 2a' (peak 5.259)

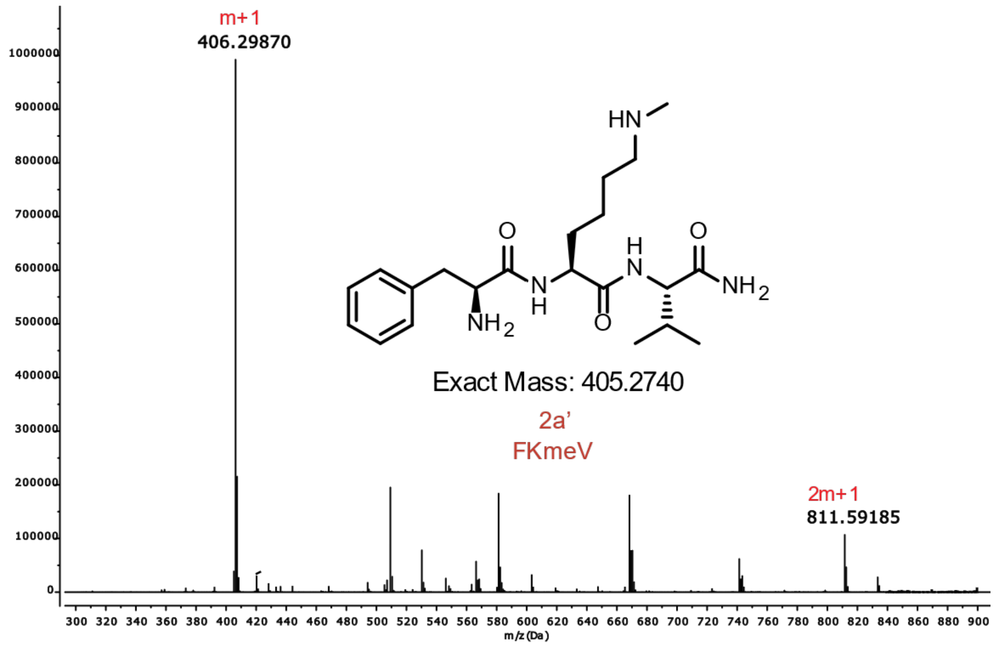

Evaluation of in-situ base generated from selectfluor without any external base.

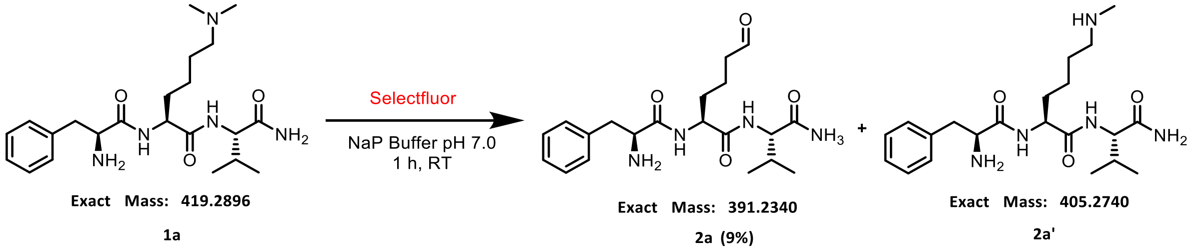

HPLC Trace of aldehyde generation reaction from 1a using Dabco-like base

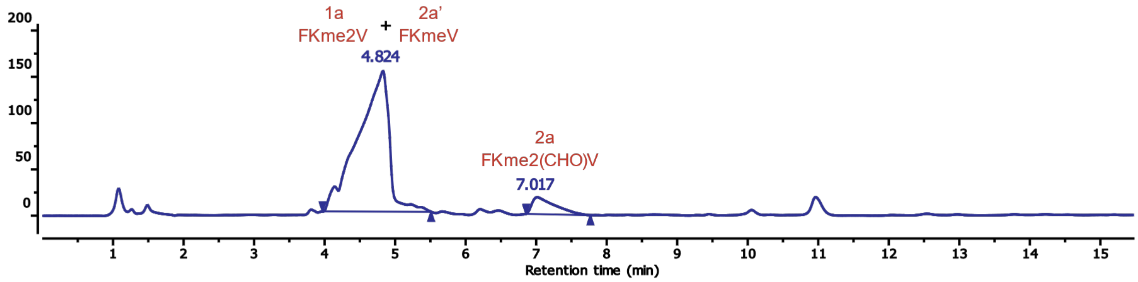

MS-Trace of peak 4.824

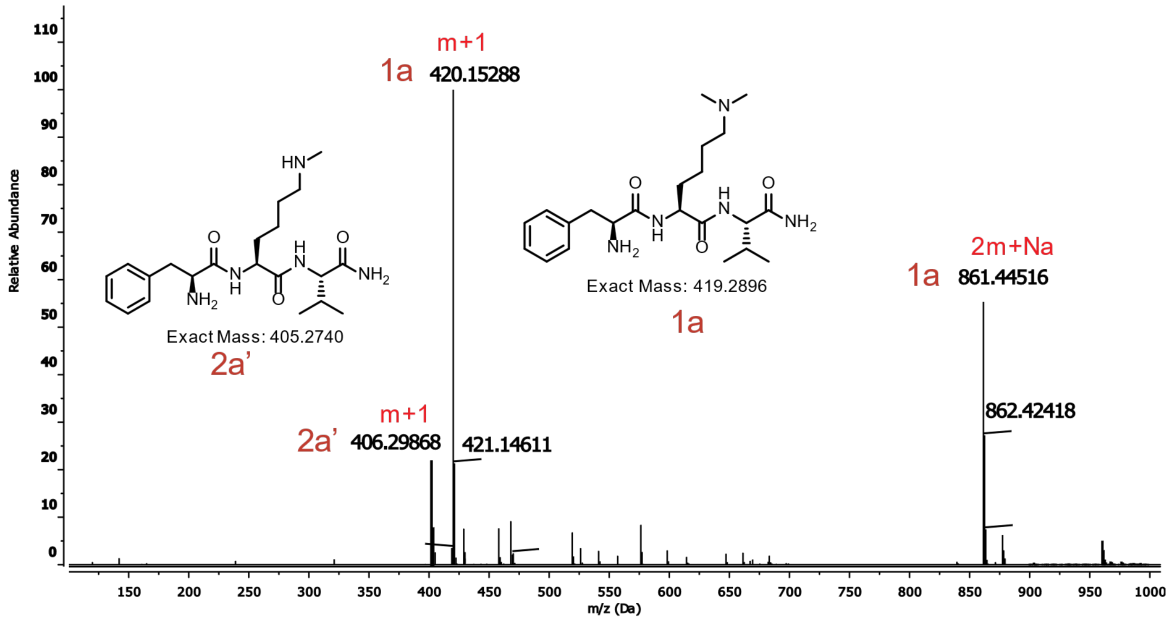

Evaluation of piperidine:

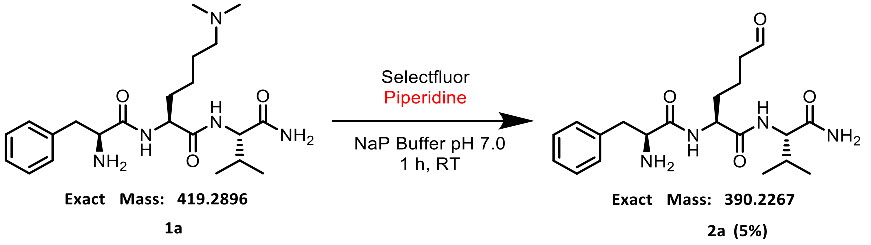

HPLC Trace of aldehyde generation reaction from 1a using piperidine

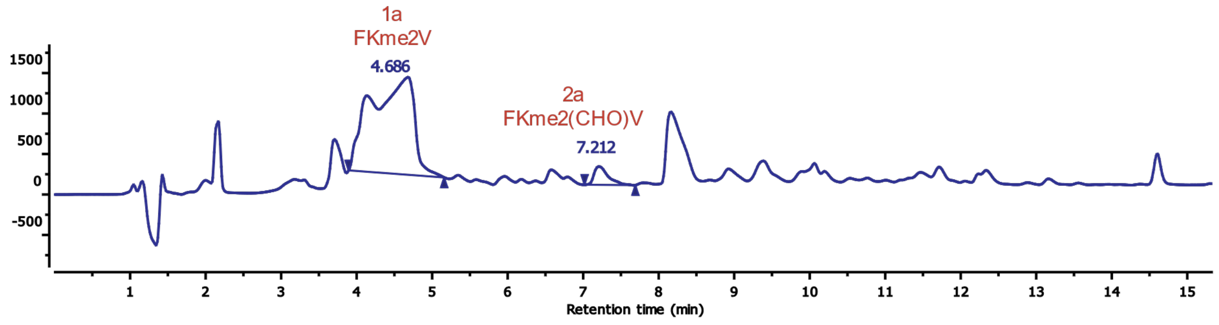

Evaluation of DMAP:

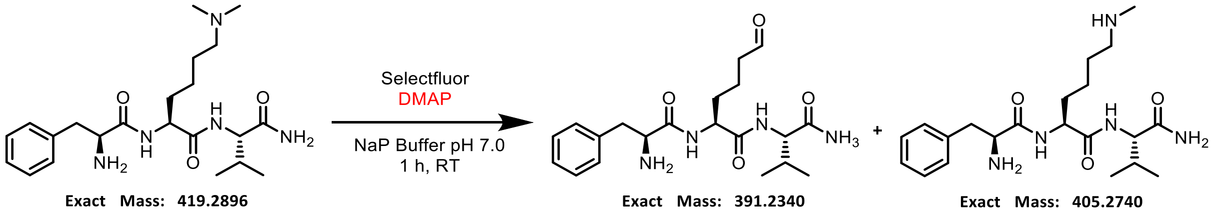

1a 2a (77%) 2a' (23%)

HPLC Trace of aldehyde generation reaction from 1a using DMAP

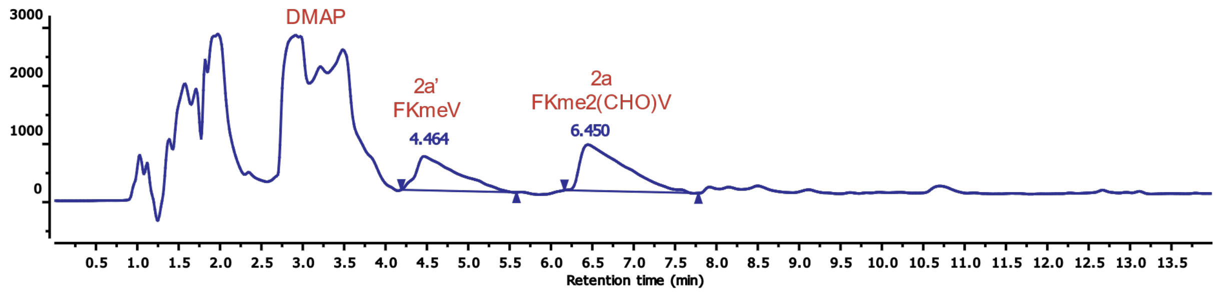

Evaluation of proton-sponge:

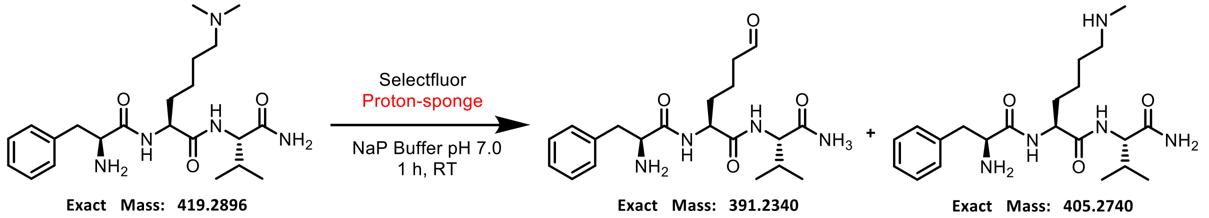

1a 2a (24%) 2a'

HPLC Trace of aldehyde generation reaction from 1a using proton-sponge

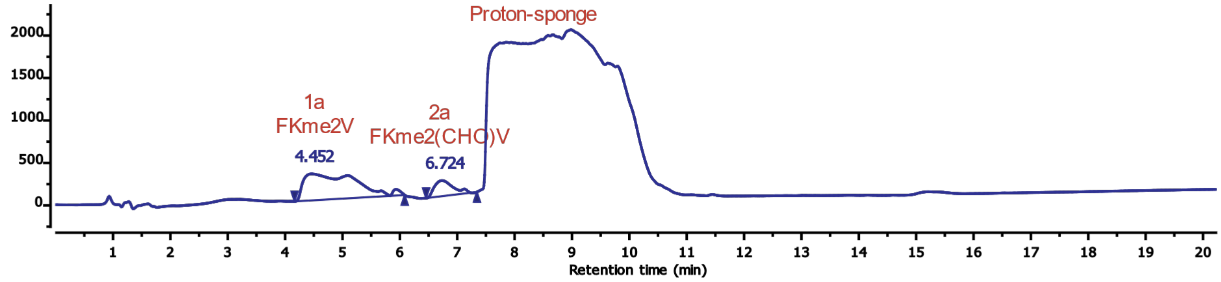

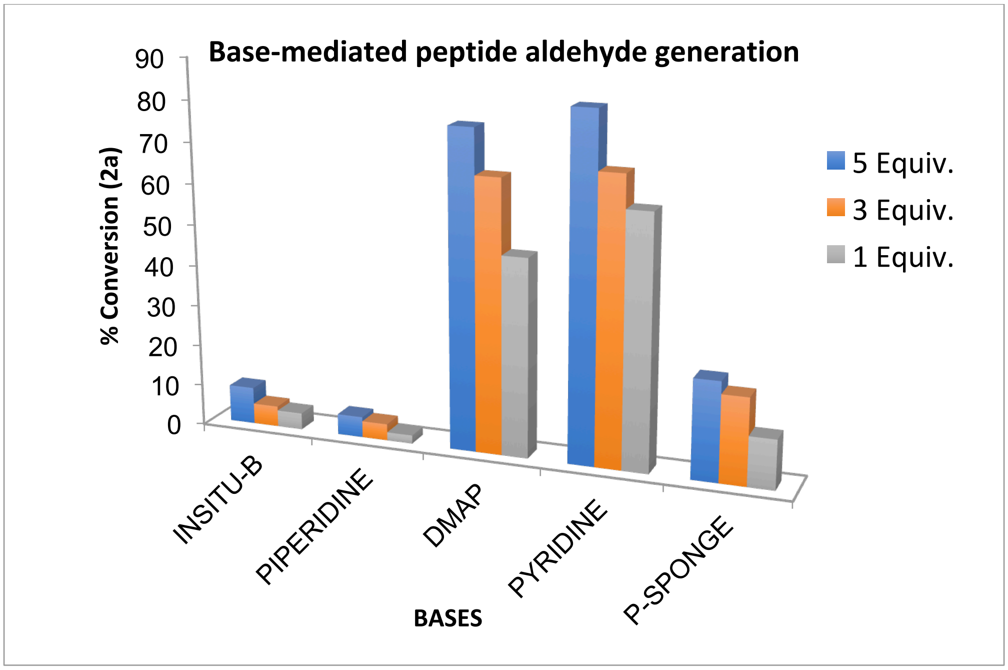

Bases	Equivalence	% Conversion (2a)
**No base (Insitu-Base)**	1	4
	3	5
	5	9
**Piperidine**	1	5
	3	4
	5	2
**DMAP**	1	48
	3	66
	5	**77**
**Pyridine**	1	61
	3	69
	5	**83**
**Proton-sponge**	1	12
	3	21
	5	24

X. Supplementary Figure 4: Characterization of peptide-aldehyde product **2b** and monomethyllysine peptide **2b'**.

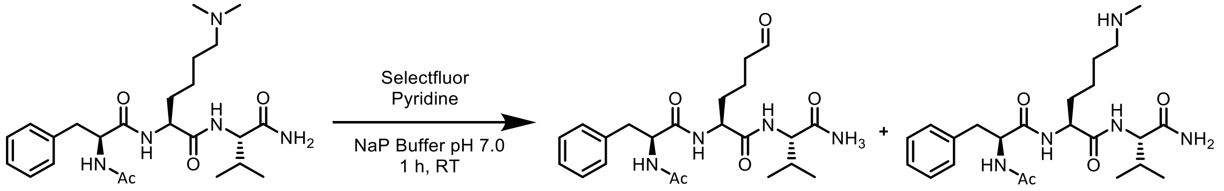

1b **2b** **2b'**

(OAc)FKme$_2$(CHO)V 2b: ^{1}**H NMR** (600 MHz, DMSO-d$_6$) δ 9.61 (t, *J* = 1.6 Hz, 1H), 7.94 (d, *J* = 7.8 Hz, 1H), 7.89 (d, *J* = 8.4 Hz, 1H), 7.83 (d, *J* = 8.3 Hz, 1H), 7.23 (d, *J* = 7.1 Hz, 3H), 7.21 – 7.18 (m, 3H), 4.40 (td, *J* = 8.6, 5.1 Hz, 1H), 4.18 (td, *J* = 8.0, 5.3 Hz, 1H), 4.10 (dd, *J* = 8.4, 6.7 Hz, 1H), 2.98 (dd, *J* = 13.8, 5.1 Hz, 1H), 2.82 (dd, *J* = 13.9, 8.8 Hz, 1H), 2.40 – 2.34 (m, 2H), 1.92 (q, *J* = 6.8 Hz, 1H), 1.87 (s, 3H), 1.57 – 1.40 (m, 5H), 0.80 (dd, *J* = 6.8, 2.4 Hz, 6H). ^{13}**C NMR** (151 MHz, DMSO) δ 203.68, 173.07, 171.69, 171.46, 169.99, 138.22, 129.58, 128.47, 126.67, 58.42, 54.10, 52.93, 43.04, 37.93, 31.62, 30.64, 22.96, 19.72, 18.65.

(OAc)FKmeV 2b': ^{1}**H NMR** (600 MHz, DMSO-d$_6$) δ 8.02 (d, *J* = 7.7 Hz, 1H), 7.99 (d, *J* = 8.3 Hz, 1H), 7.86 (d, *J* = 8.2 Hz, 1H), 7.26 – 7.22 (m, 2H), 7.22 – 7.17 (m, 3H), 4.40 (td, *J* = 8.6, 5.0 Hz, 1H), 4.16 (td, *J* = 8.3, 5.3 Hz, 1H), 4.09 (dd, *J* = 8.3, 6.7 Hz, 1H), 3.00 (dd, *J* = 13.9, 5.0 Hz, 1H), 2.86 – 2.82 (m, 1H), 2.61 (d, *J* = 1.9 Hz, 2H), 2.61 (t, *J* = 7.4 Hz, 2H), 2.40 (s, 3H), 1.93 (h, *J* = 6.7 Hz, 2H), 1.88 (s, 3H), 1.59 – 1.48 (m, 2H), 1.47 – 1.41 (m, 2H), 1.25 – 1.16 (m, 2H), 0.81 (dd, *J* = 6.9, 1.2 Hz, 6H). ^{13}**C NMR** (151 MHz, DMSO) δ 173.19, 171.76, 171.63, 170.09, 138.27, 129.59, 128.47, 126.67, 58.58, 54.16, 53.14, 49.75, 37.90, 34.39, 31.72, 30.60, 22.94 (d, *J* = 7.5 Hz), 19.74, 18.70.

^{1}H NMR (600 MHz, DMSO-d$_6$) of aldehyde-peptide 2b

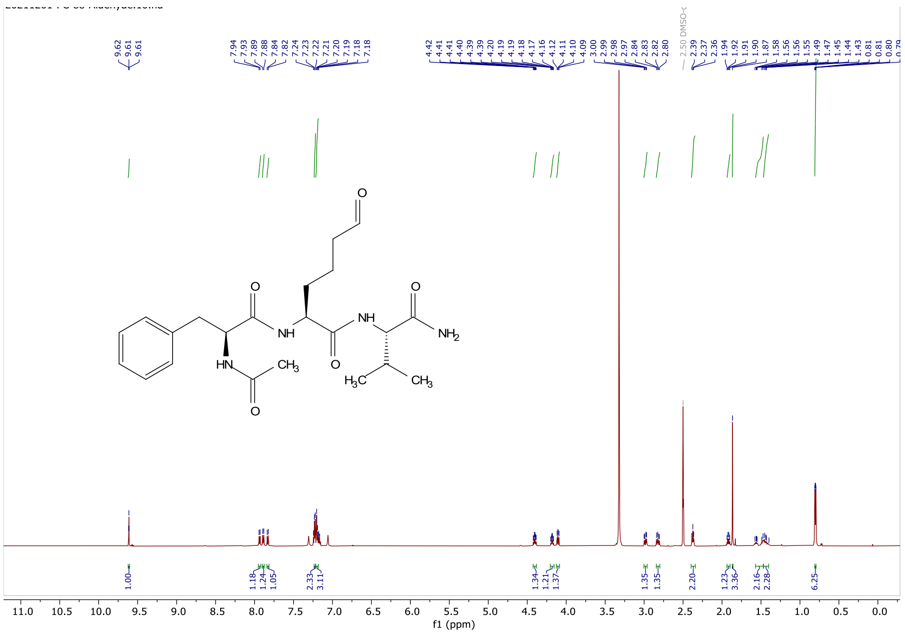

^{13}C NMR (151 MHz, DMSO-d6) of aldehyde-peptide 2b

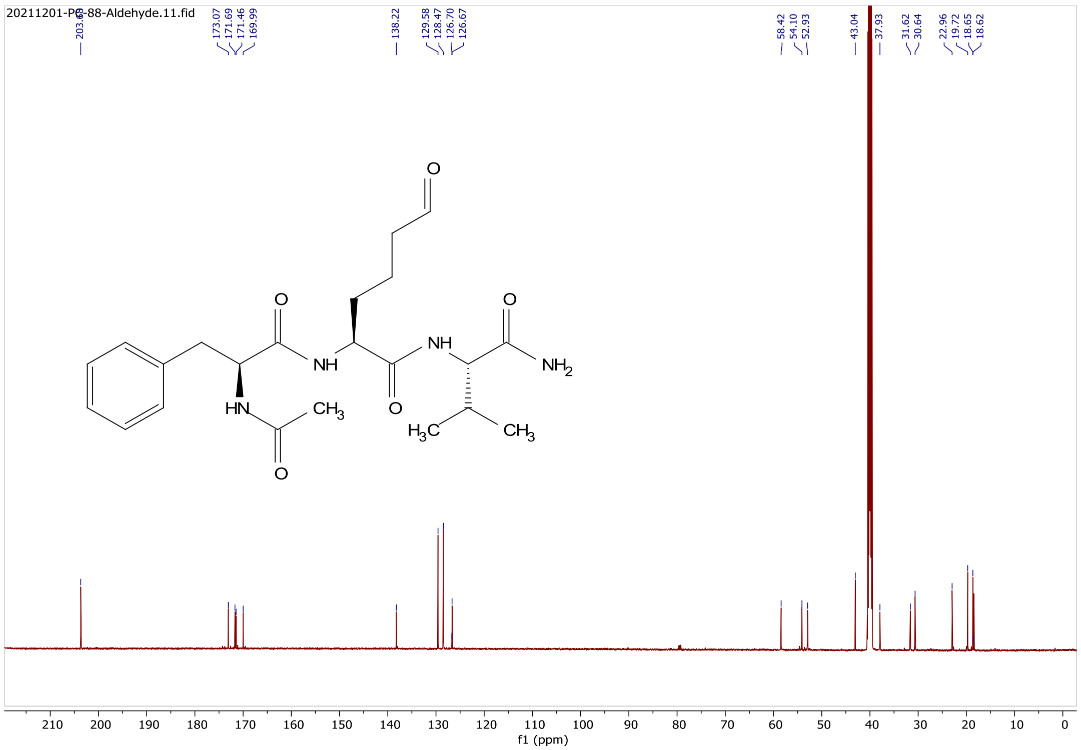

^{1}H NMR (600 MHz, DMSO-d_6) of monomethyl lysine peptide (OAc)FKmeV 2b'

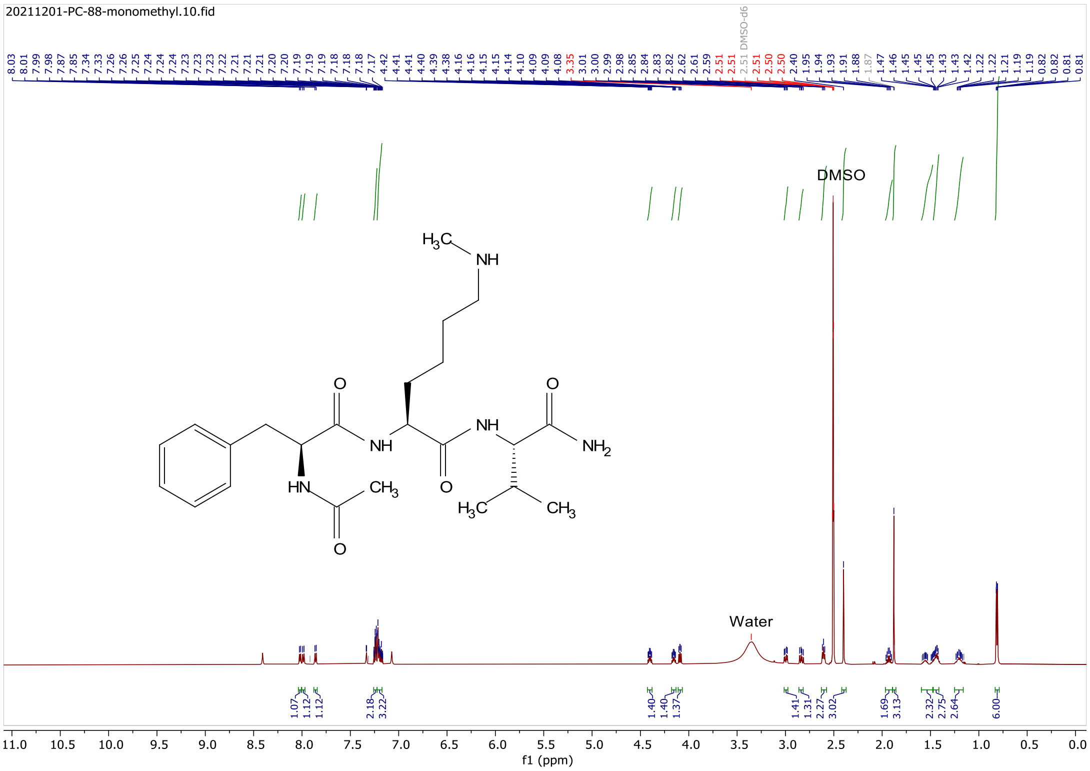

^{13}C NMR (151 MHz, DMSO-d_6) of of monomethyl lysine peptide (OAc)FKmeV 2b’

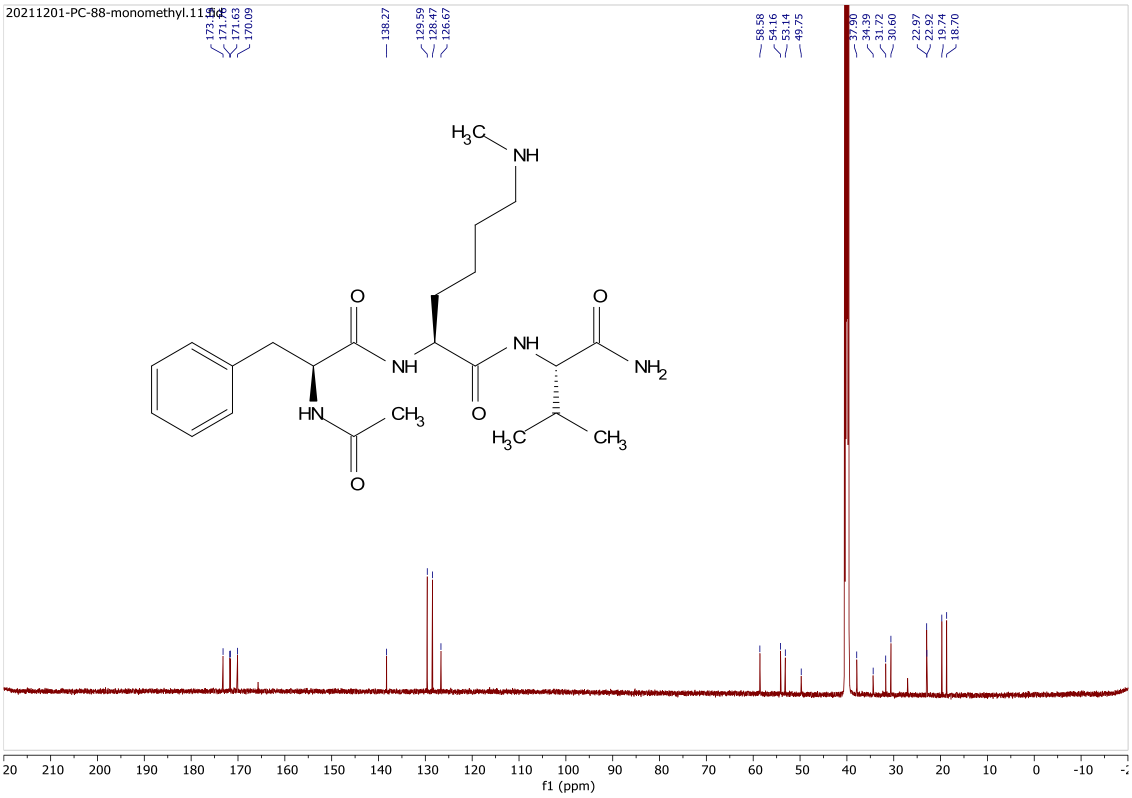

XI. Supplementary Figure 5: Chemoselectivity studies for aldehyde-peptide formation.

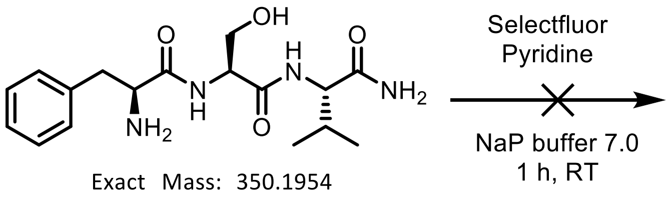

1.0 mg of serine containing tripeptide FSV was modified using **general procedure 1.** The reaction mixture was analyzed by HPLC using method A to determine the % conversion. No modification of the starting peptide was observed.

FSV linear peptide. LCMS: *m/z* 351.15584 (calcd [M+H]+ = 351.2027), *m/z* 373.16921 (calcd [M+Na] = 373.16), *m/z* 389.13582 (calcd [M+K] = 389.1591), *m/z* 701.44533 (calcd [2M+H]+ = 701.3981), *m/z* 723.43486 (calcd [2M+Na] = 723.2800), (HPLC analysis at 220 nm). Retention time in HPLC: 6.029

HPLC Trace of chemoselectivity evaluation of serine

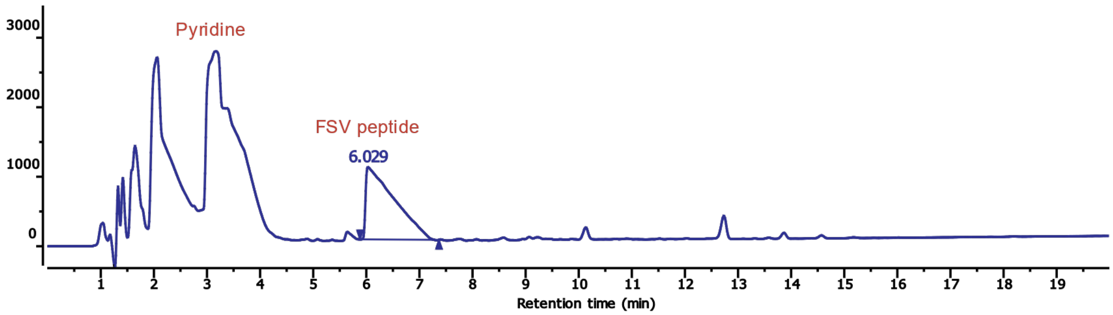

MS-Trace of peak 6.029

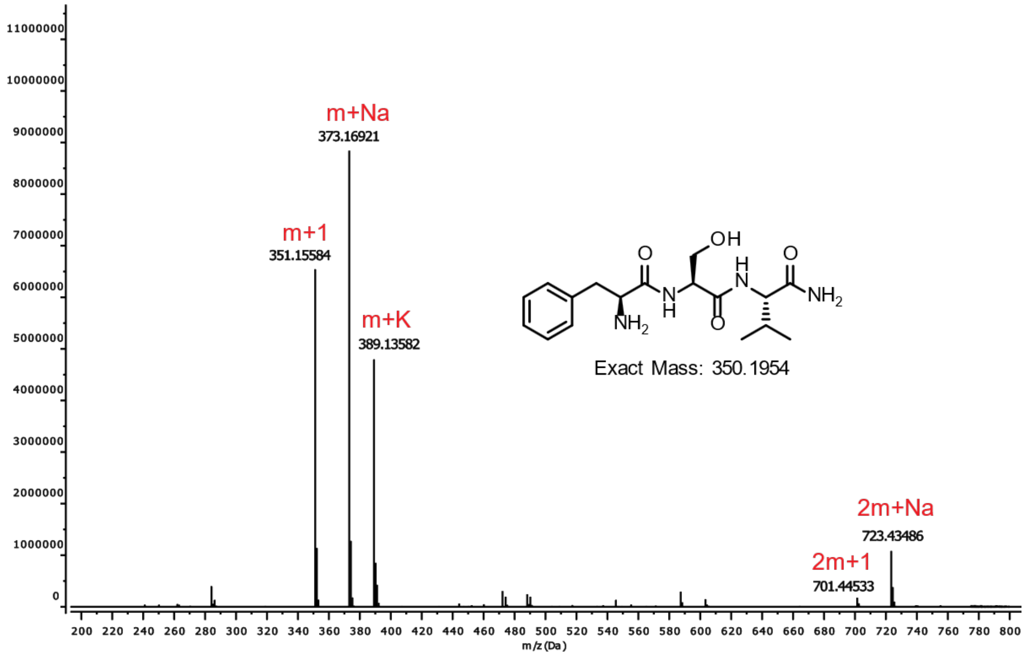

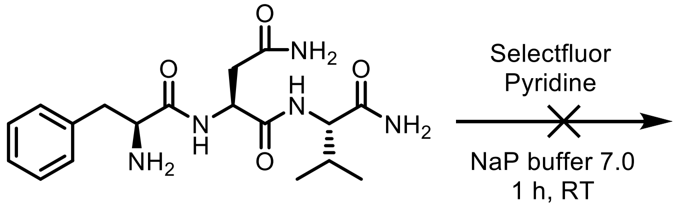

Exact Mass: 377.2063

1.0 mg of asparagine containing tripeptide FNV was modified using **general procedure 1.** The reaction mixture was analyzed by HPLC using method A to determine the % conversion. No modification of the starting peptide was observed.

FNV linear peptide. LCMS: *m/z* 378.19993 (calcd [M+H]+ = 378.2136), *m/z* 400.20011 (calcd [M+Na]+ = 400.1955), *m/z* 416.14965 (calcd [M+K]+ = 416.1695), *m/z* 755.46067 (calcd [2M+H]+ = 755.4199), *m/z* 777.44211 (calcd [2M+Na]+ = 777.4018), (HPLC analysis at 220 nm). Retention time in HPLC: 5.664

HPLC Trace of chemoselectivity evaluation of asparagine

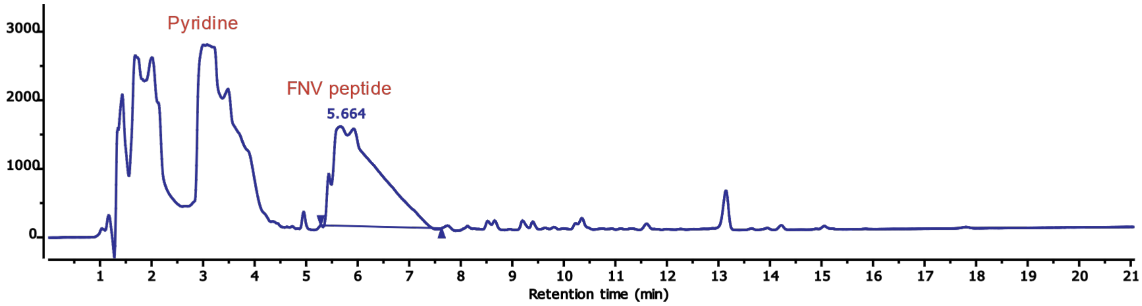

MS-Trace of peak 5.664

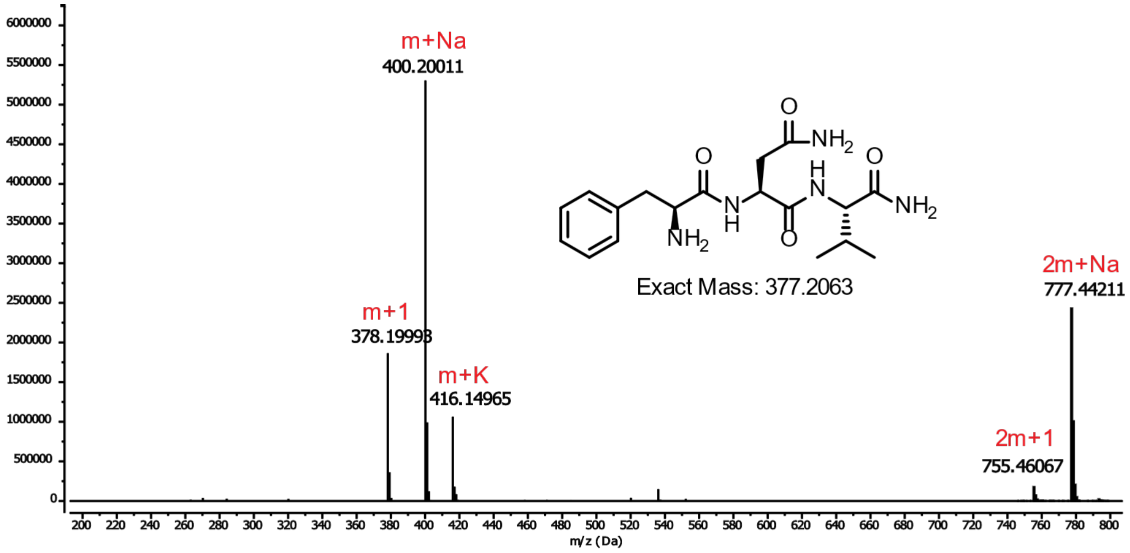

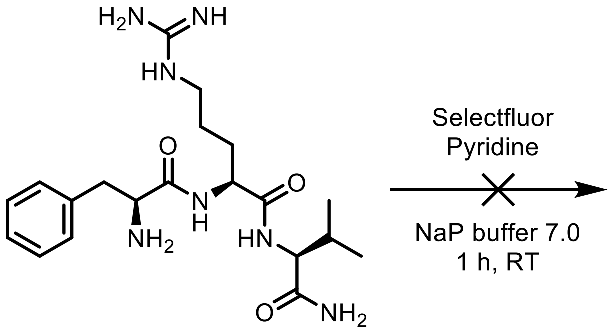

Exact Mass: 419.2645

1.0 mg of arginine containing peptide FRV was modified using **general procedure 1.** The reaction mixture was analyzed by HPLC using method A to determine the % conversion. No modification of the starting peptide was observed.

FRV linear peptide. LCMS: *m/z* 420.12929 (calcd $[M+H]^+$ = 420.2718), *m/z* 210.56289 (calcd $[M+2/2]^+$ = 210.5659), *m/z* 839.39032 (calcd $[2M+H]^+$ = 839.5363), (HPLC analysis at 220 nm). Retention time in HPLC: 4.574

HPLC Trace of chemoselectivity evaluation of arginine

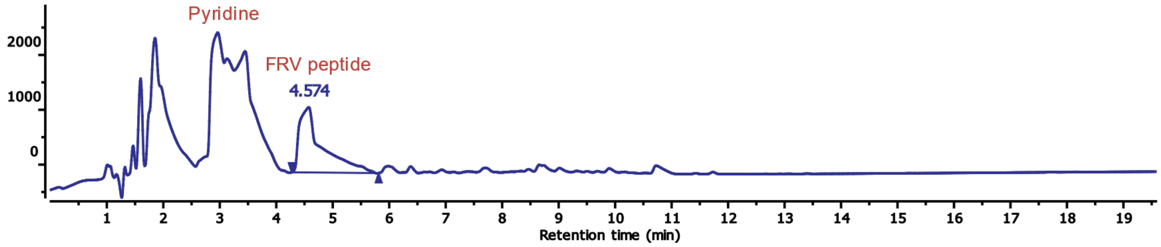

MS-Trace of peak 4.574

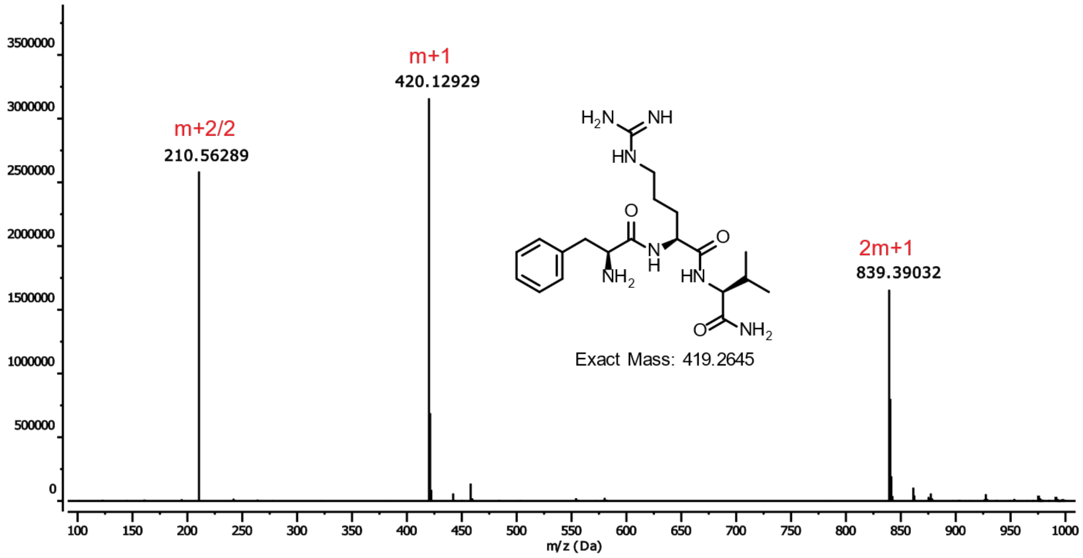

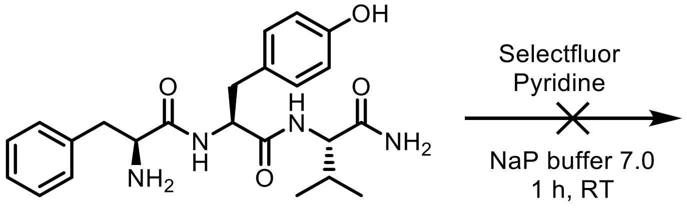

Exact Mass: 426.2267

1.0 mg of tyrosine containing tripeptide FYV was modified using **general procedure 1.** The reaction mixture was analyzed by HPLC using method A to determine the % conversion. No modification of the starting peptide was observed.

FYV linear peptide. LCMS: *m/z* 427.22984 (calcd [M+H]+ =427.2340), *m/z* 449.18843 (calcd [M+Na]+ = 449.2159), *m/z* 465.23626 (calcd [M+K]+ = 465.1899), (HPLC analysis at 220 nm). Retention time in HPLC: 5.614

HPLC Trace of chemoselectivity evaluation of tyrosine

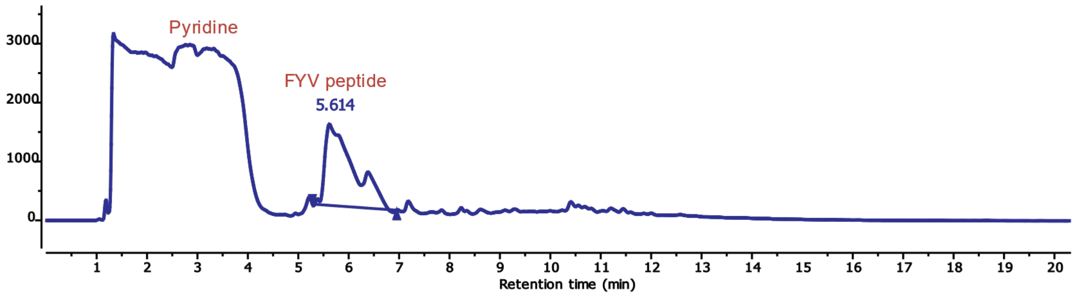

MS-Trace of peak 5.614

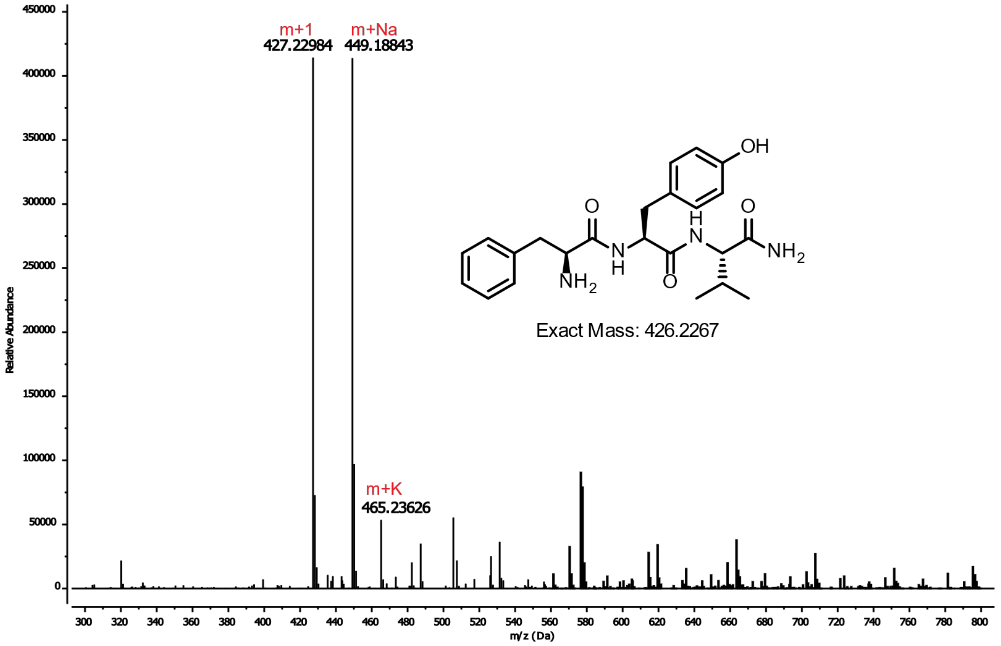

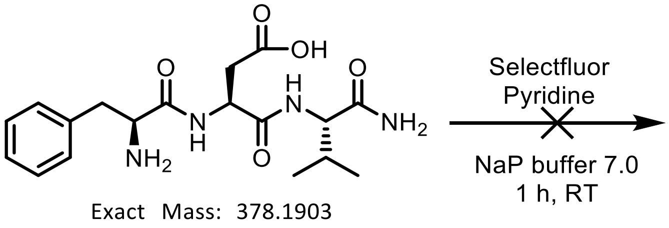

1.0 mg of aspartic acid containing tripeptide FDV was modified using **general procedure 1.** The reaction mixture was analyzed by HPLC using method A to determine the % conversion. No modification of the starting peptide was observed

FDV linear peptide. LCMS: m/z 379.09165 (calcd [M+H]+ = 379.1976), m/z 401.02190 (calcd [M+Na]+ = 401.1795), m/z 417.03049 (calcd [M+K]+ = 417.1535), (HPLC analysis at 220 nm). Retention time in HPLC: 6.446

HPLC Trace of chemoselectivity evaluation of aspartic acid

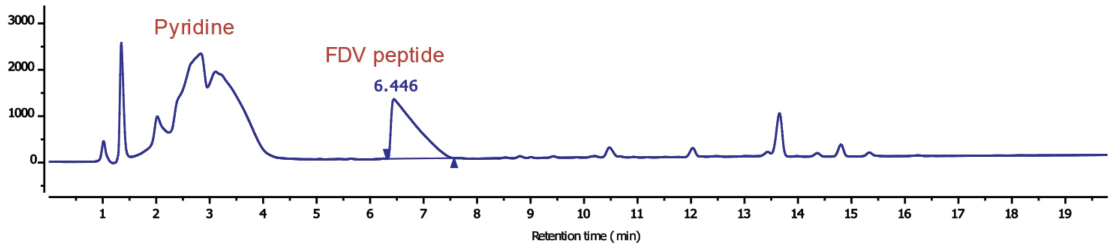

MS-Trace of peak 6.446

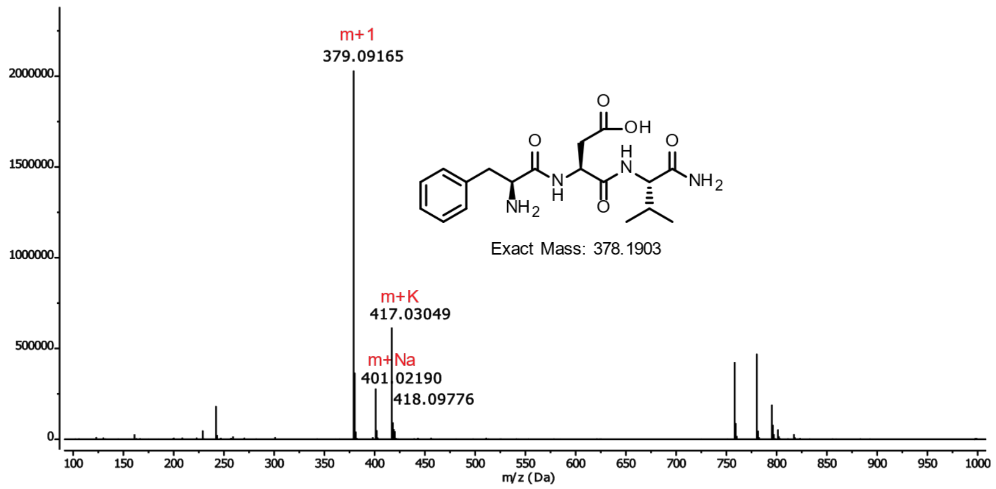

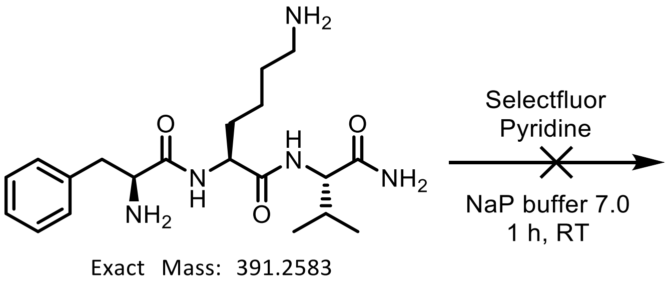

1.0 mg of lysine containing tripeptide FKV was modified using **general procedure 1.** The reaction mixture was analyzed by HPLC using method A to determine the % conversion. No modification of the starting peptide was observed.

FKV linear peptide. LCMS: m/z 392.15067 (calcd [M+H]+ = 392.2656), m/z 392.15067 (calcd [M+2/2]+ = 196.6291), m/z 783.37022 (calcd [2M+H]+ = 783.5240), (HPLC analysis at 220 nm). Retention time in HPLC: 4.960

HPLC Trace of chemoselectivity evaluation of lysine

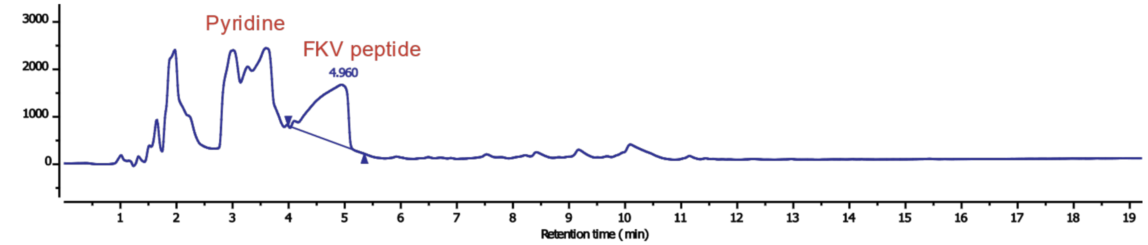

MS-Trace of peak 4.960

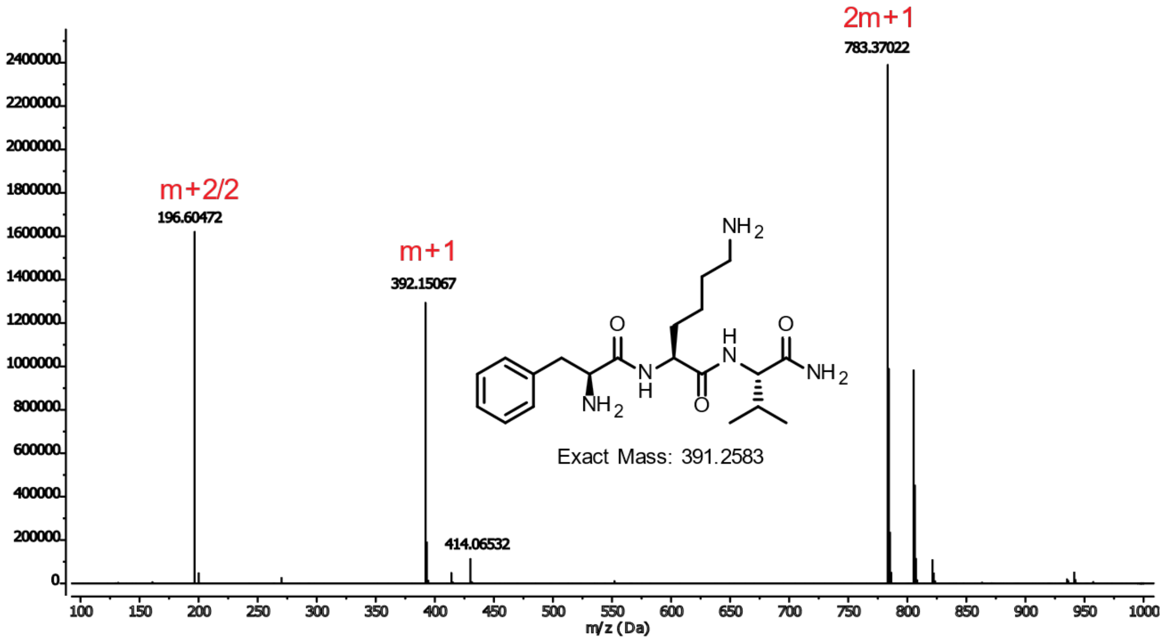

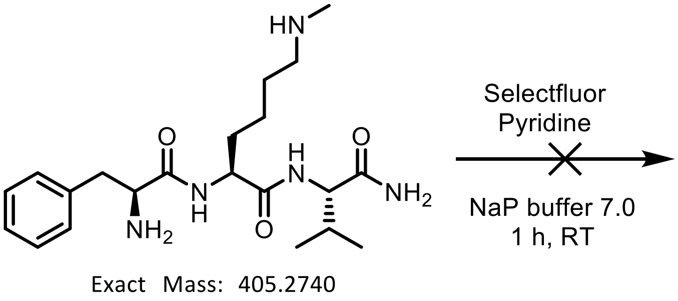

1.0 mg of monomethyllysine containing tripeptide FKmeV was modified using **general procedure 1.** The reaction mixture was analyzed by HPLC using method A to determine the % conversion. No modification of the starting peptide was observed.

FKmeV linear peptide **2b**. LCMS: m/z 406.29870 (calcd [M+H]+ = 406.54), m/z 811.59185 (calcd [2M+H]+ = 811.59), (HPLC analysis at 220 nm). Retention time in HPLC: 4.872

HPLC Trace of chemoselectivity evaluation of monomethyllysine

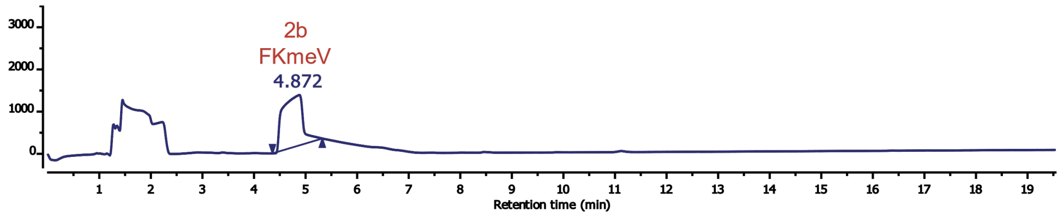

MS-Trace of peak 4.872

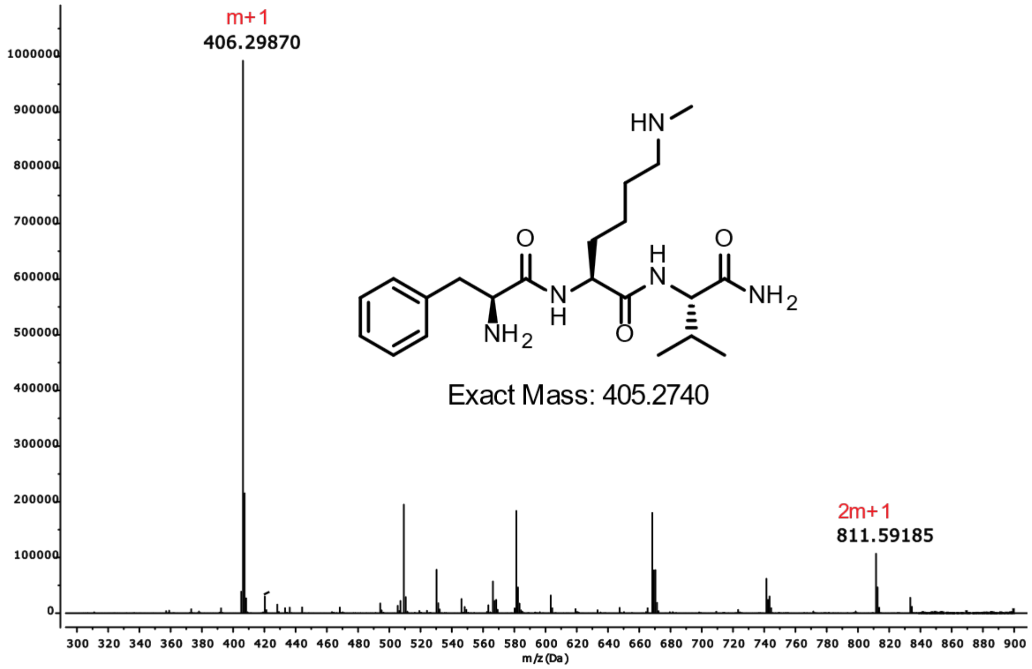

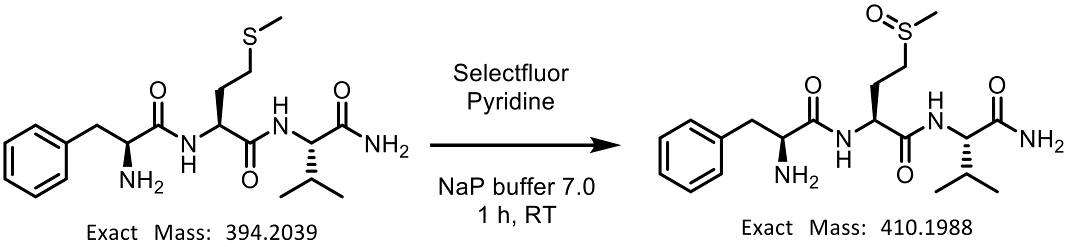

1.0 mg of methionine containing tripeptide FMV was modified using **general procedure 1.** The reaction mixture was analyzed by HPLC using method A to determine the % conversion. The conversion of the sulfoxide product was determined to be (97%).

FMV linear peptide. LCMS: *m/z* 395.06721 (calcd [M+H]+ = 395.2111), (HPLC analysis at 220 nm). Retention time in HPLC: 6.839

FMV sulfoxide peptide product. LCMS: *m/z* 411.06721 (calcd [M+H]+ = 411.2061), *m/z* 821.22852 (calcd [2M+H]+ = 821.4048), *m/z* 843.19960 (calcd [2M+Na]+ = 843.3868), (HPLC analysis at 220 nm). Retention time in HPLC: 6.001

HPLC Trace of chemoselectivity evaluation of methionine

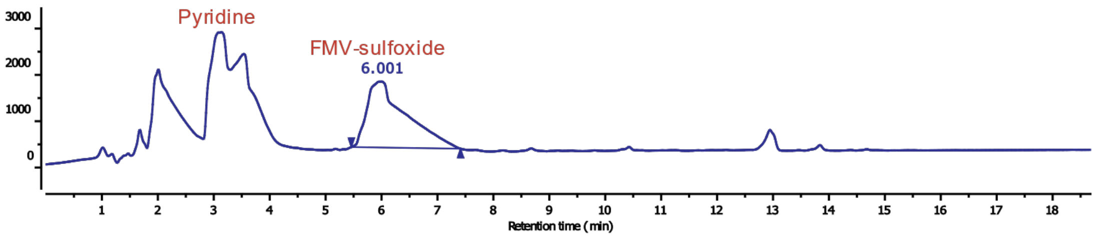

MS-Trace of peak 6.001

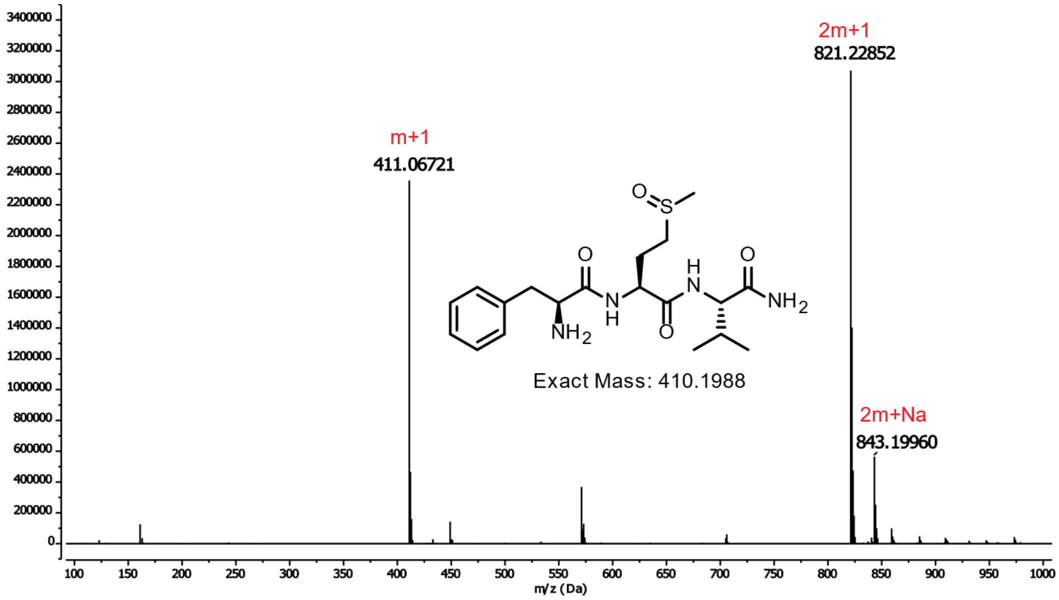

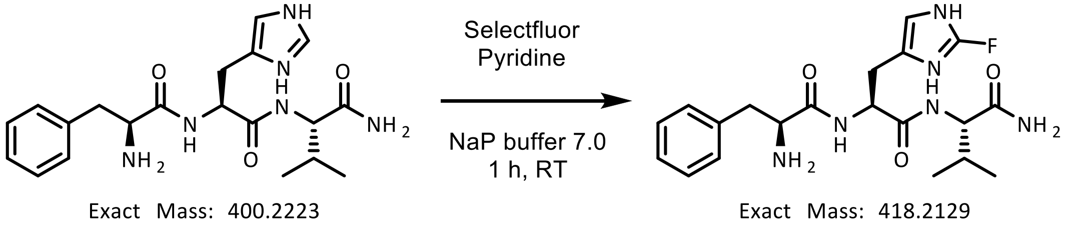

1 mg of histidine containing tripeptide FHV was modified using **general procedure 1.** The reaction mixture was analyzed by HPLC using method A to determine the % conversion. The conversion of the fluorinated product was determined to be (14%).

FHV linear peptide. LCMS: *m/z* 401.06929 (calcd [M+H]+ = 401.2296), *m/z* 201.03720 (calcd [M+2/2]+ = 201.1148), *m/z* 801.27315 (calcd [2M+H]+ = 801.4519), *m/z* 823.24819 (calcd [2M+Na]+ = 823.4338), (HPLC analysis at 220 nm). Retention time in HPLC: 4.356

FHV flourination peptide product. LCMS: *m/z* 419.06721 (calcd [M+H]+ = 419.2201), *m/z* 210.03720 (calcd [M+2/2]+ = 210.1100), (HPLC analysis at 220 nm). Retention time in HPLC: 7.295

HPLC Trace of chemoselectivity evaluation of histidine

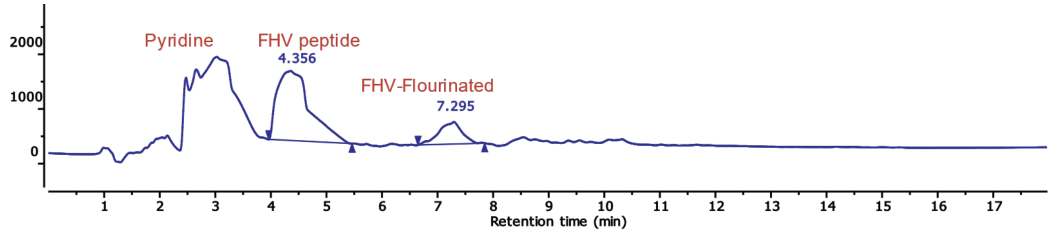

MS-Trace of peak 4.356

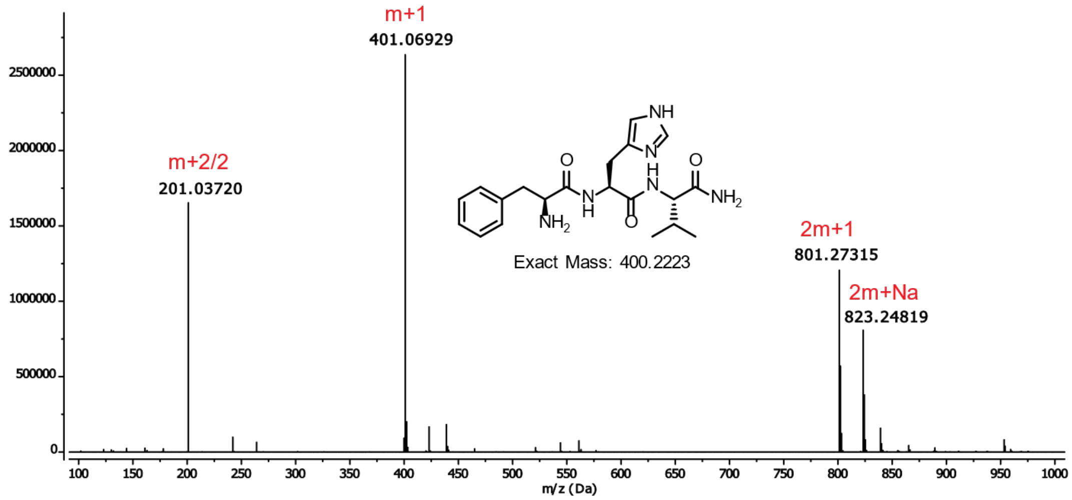

MS-Trace of peak 7.295

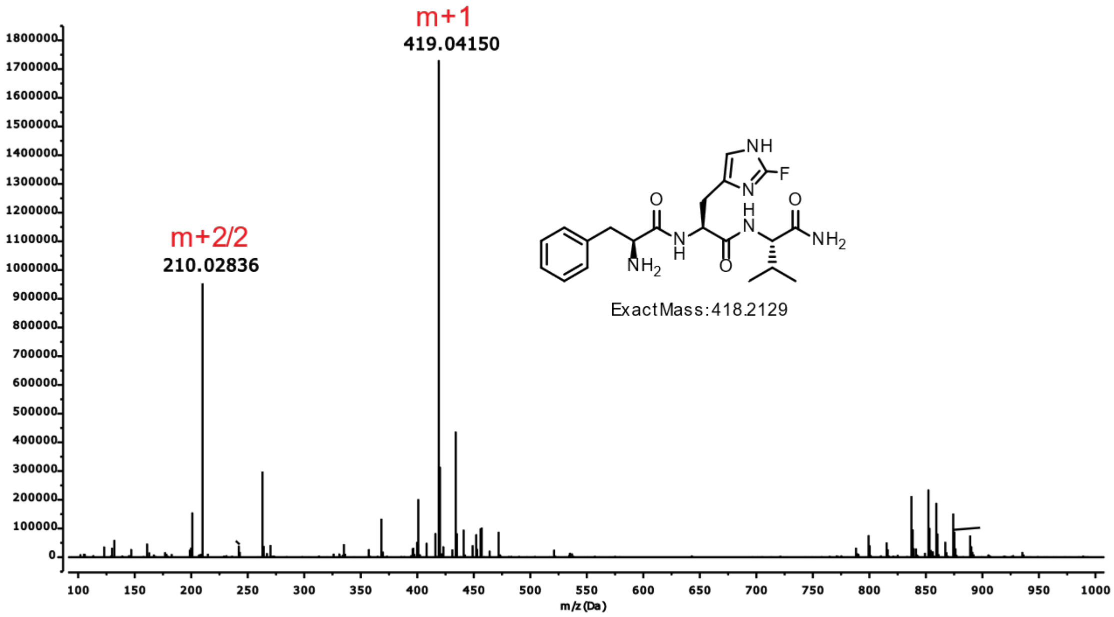

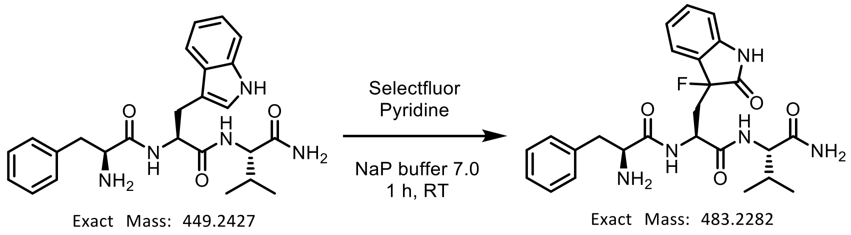

1.0 mg of tryptophan containing tripeptide FWV was modified using **general procedure 1.** The reaction mixture was analyzed by HPLC using method A to determine the % conversion. The conversion of the fluorinated product was determined to be (57%).

FWV linear peptide. LCMS: m/z 450.26093 (calcd [M+H]+ = 450.2500), LCMS: m/z 472.21833 (calcd [M+Na]+ = 472.2319), (HPLC analysis at 220 nm). Retention time in HPLC: 9.763

FWV flourinated peptide product. LCMS: m/z 484.10143 (calcd [M+H]+ = 484.2355), (HPLC analysis at 220 nm). Retention time in HPLC: 10.463

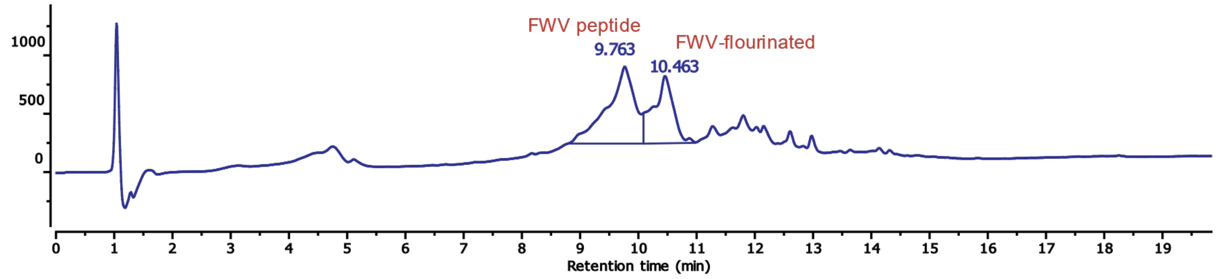

MS-Trace of peak 9.763

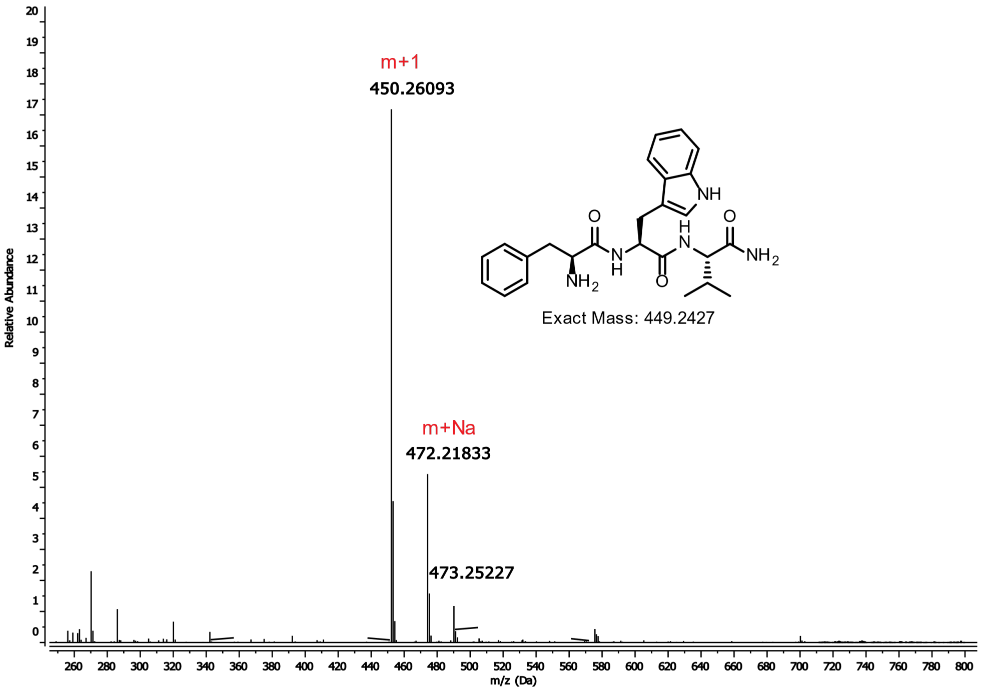

MS-Trace of peak 10.463

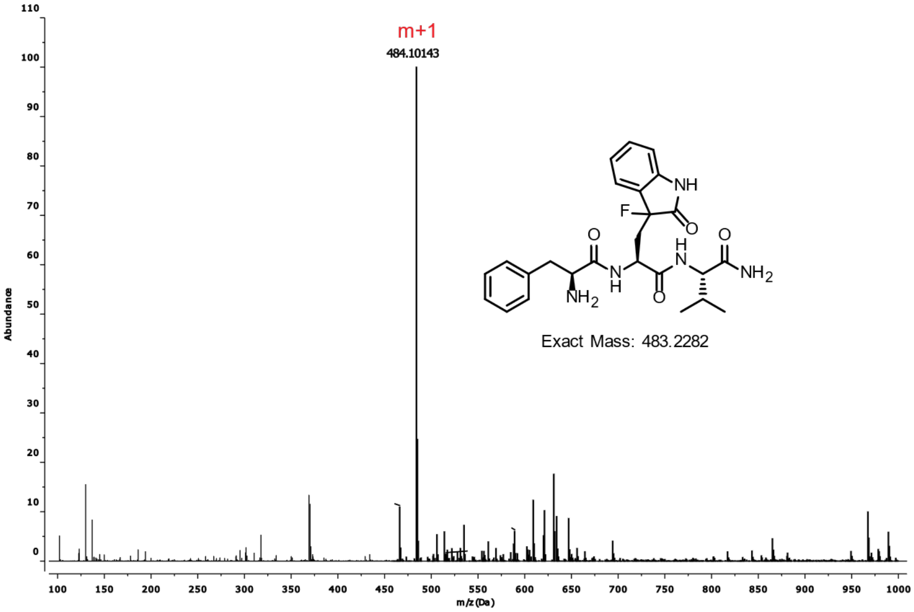

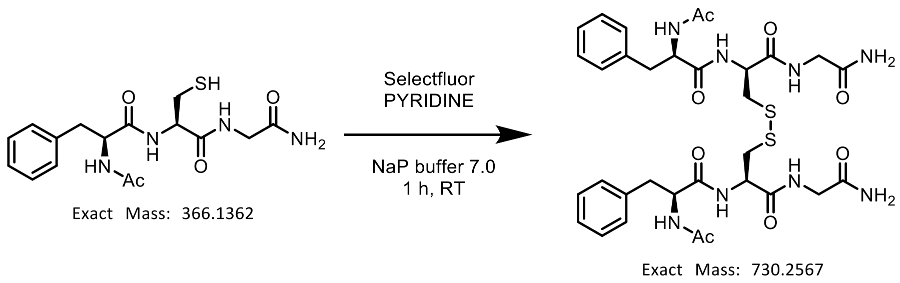

1.0 mg of cysteine containing tripeptide FCV was modified using **general procedure 1.** The reaction mixture was analyzed by HPLC using method A to determine the % conversion. The disulfide product was observed to be (54%).

FCV linear peptide. LCMS: m/z 367.12273 (calcd [M+H]+ = 367.1435), m/z 389.11641 (calcd [M+H]+ = 389.1254), m/z 405.11966 (calcd [M+H]+ = 405.0993), (HPLC analysis at 220 nm). Retention time in HPLC: 8.473

FCV disulfide peptide product. LCMS: *m/z* 731.46219 (calcd [M+H]+ = 731.2640), m/z 366.36320 (calcd [M+2/2]+ = 366.1327), *m/z* 753.44483 (calcd [M+Na]+ = 753.2459), (HPLC analysis at 220 nm). Retention time in HPLC: 10.932

HPLC Trace of chemoselectivity evaluation of cysteine

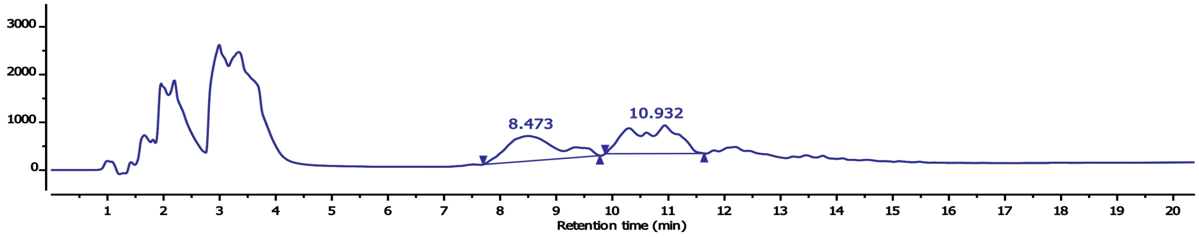

MS-Trace of peak 8.473

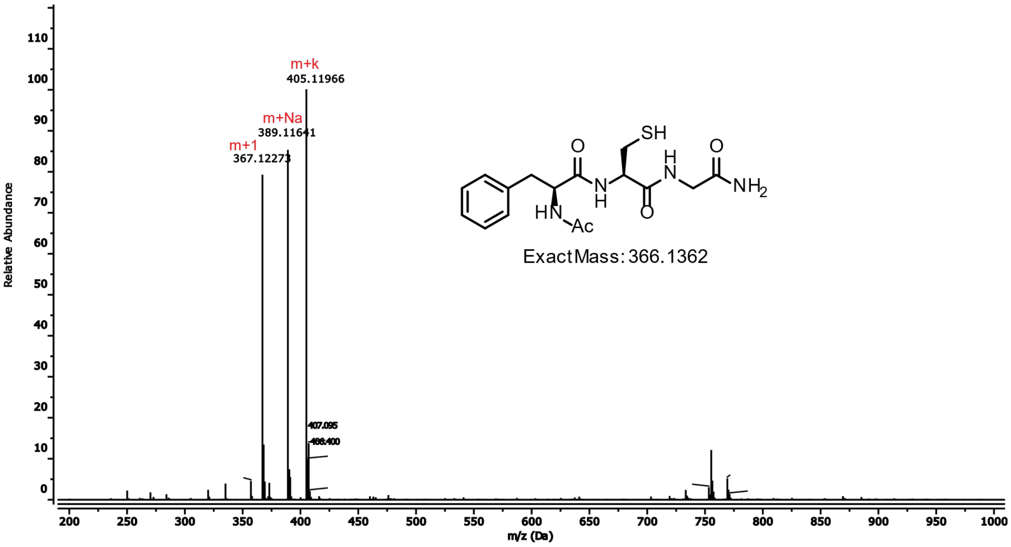

MS-Trace of peak 10.932

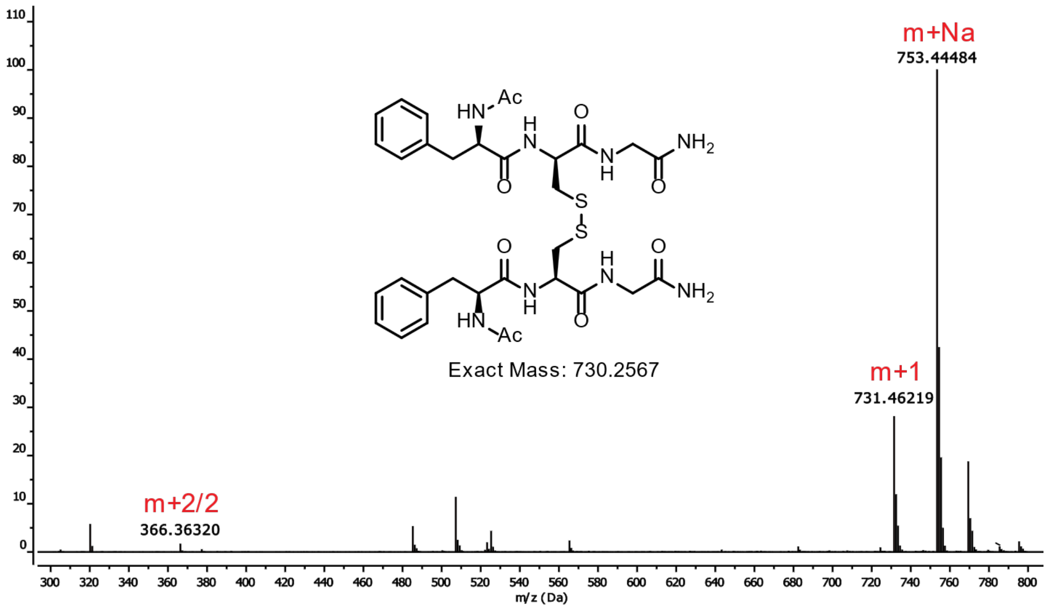

XII. Supplementary Figure 6. Evaluation of effect of adducts on enrichment of Kme_2-generated aldehyde.

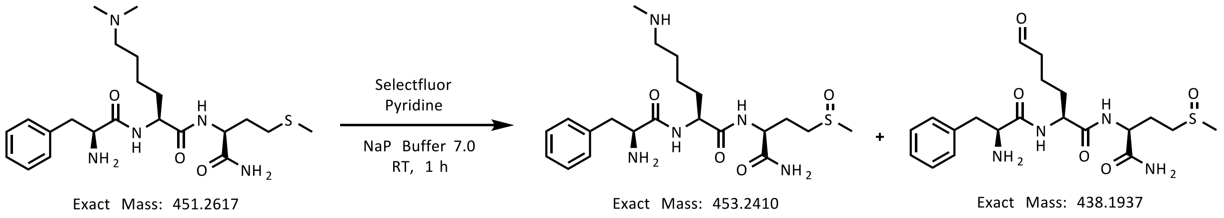

To 1.0 mg of $FKme_2M$ peptide dissolved in 300 μL of 10 mM sodium phosphate buffer (NaP, pH 7.0), was added pyridine (5 eq) and selectfluor (2 eq). The reaction mixture was stirred for 1 h. Samples were taken from the reaction mixture, injected into LC-MS to monitor the generation of peptide aldehyde and sulfoxide product. The reaction mixture was analyzed by HPLC using method A and product peak purified by preparative HPLC.

FKmeM(sulfoxide). LCMS: *m/z* 454.36388 (calcd [M+H]+ = 454.2483), *m/z* 476.34103 (calcd [M+Na]+ = 476.2302), (HPLC analysis at 220 nm, **method A**). Retention time in HPLC: 3.982

FK(CHO)M(sulfoxide). LCMS: *m/z* 439.05783 (calcd [M+H]+ = 439.2010), *m/z* 877.11566 (calcd [2M+1]+ = 877.3947), (HPLC analysis at 220 nm, **method A**). Retention time in HPLC: 4.451

HPLC Trace of reaction

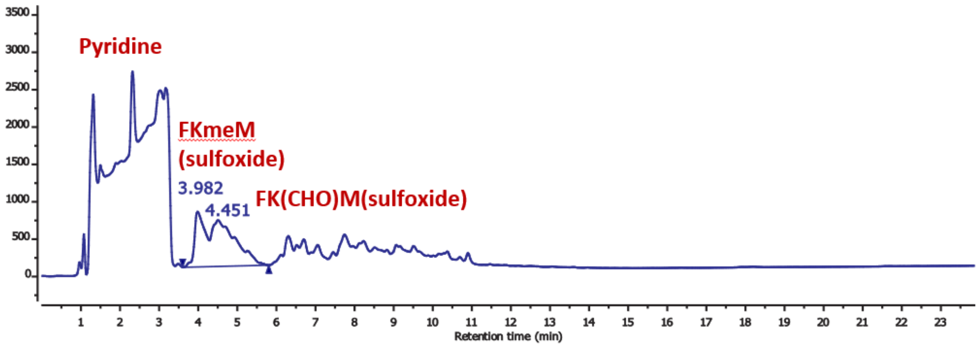

MS-Trace of peak 3.982 (FKmeM(sulfoxide))

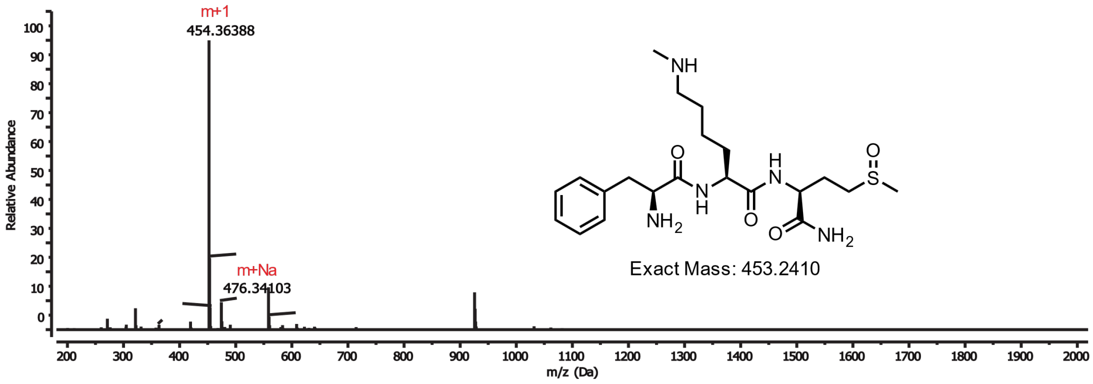

MS-Trace of peak 4.451 (FK(CHO)M(sulfoxide))

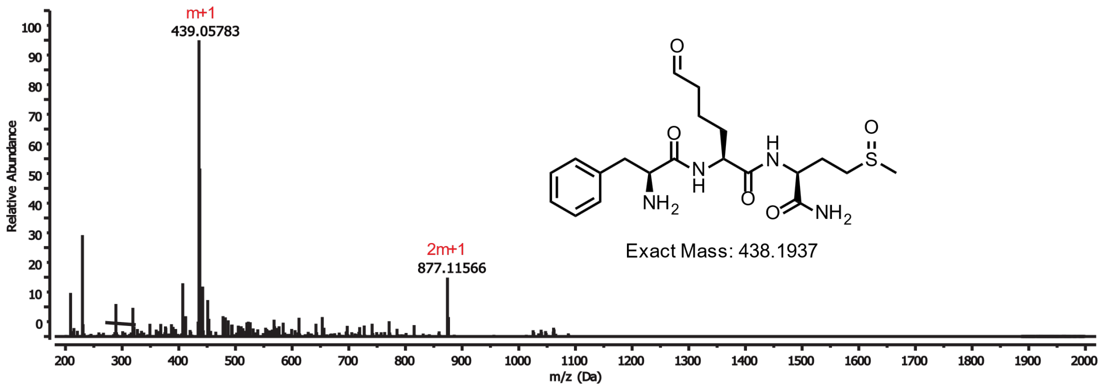

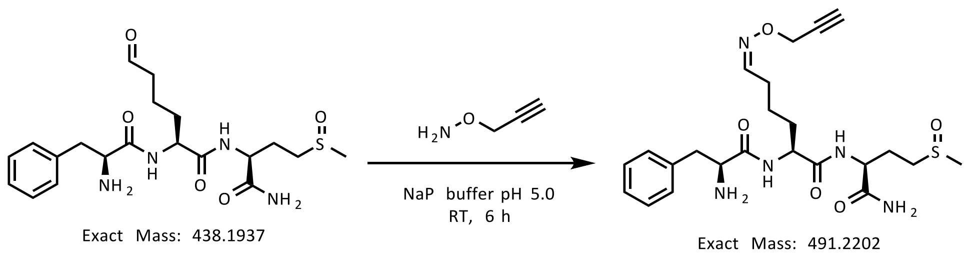

To 1 mg of aldehyde-sulfoxide peptide **FK(CHO)M(sulfoxide)** in 300 µL of NaP buffer was added 4 equivalences of O-2-Propynylhydroxylamine hydrochloride. The reaction was stirred for 6 h. Samples were taken from the reaction mixture, injected into LC-MS to monitor the modification of the peptide **FK(CHO)M(sulfoxide)** to the oxime propargyl peptide product. The reaction mixture was analyzed by HPLC using method B and the % conversion to the oxime propargyl peptide product (>98%).

FK(CHO)M(sulfoxide) propargyl oxime. LCMS: *m/z* 492.27461 (calcd [M+H]+ = 492.2275), (HPLC analysis at 220 nm, **method A**). Retention time in HPLC: 5.384

HPLC Trace of reaction

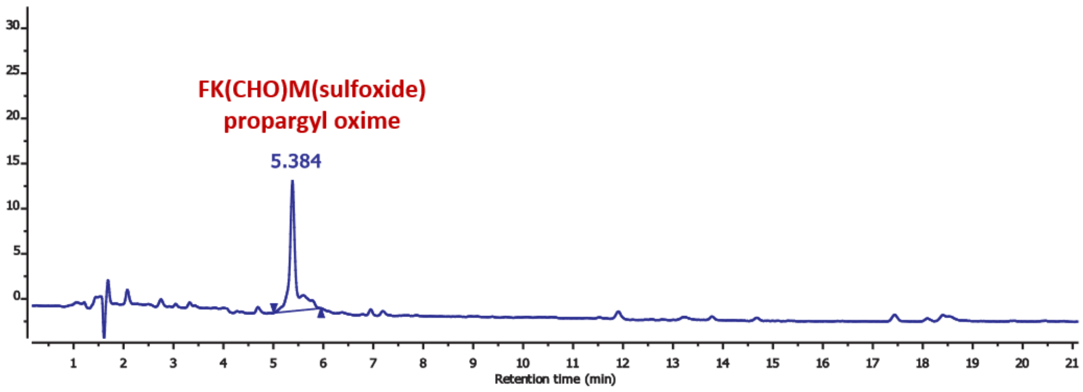

MS-Trace of peak 5.384 (FK(CHO)M(sulfoxide) propargyl oxime)

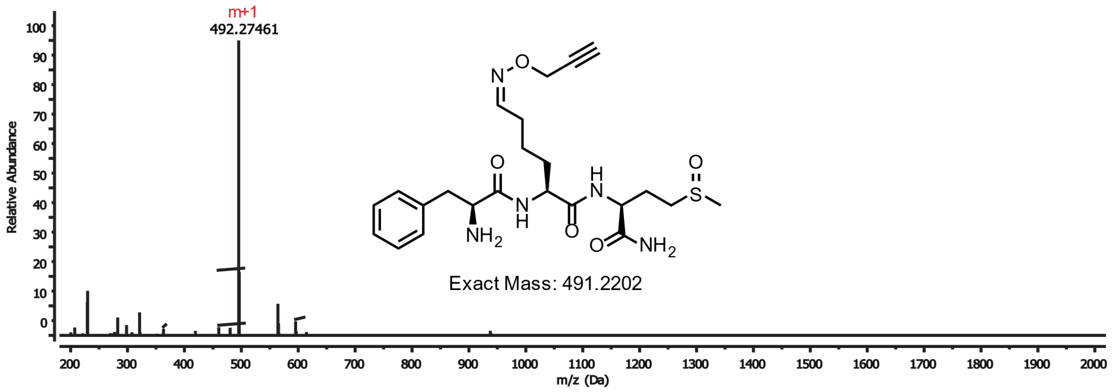

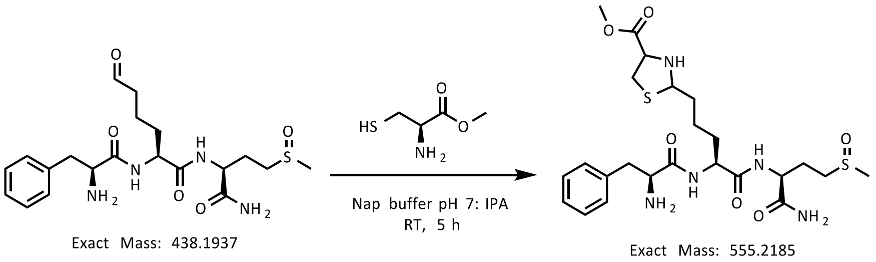

To 1 mg of aldehyde-sulfoxide peptide **FK(CHO)M(sulfoxide)** in 300 µL of NaP buffer and isopropyl alcohol (IPA) was added 5 equivalences of cysteine methylester. Reaction was stirred at room temperature for 5 h. Samples were taken from the reaction mixture, injected into LC-MS to monitor the formation of thiazolidine. The reaction mixture was analyzed by HPLC using method A and % conversion to thiazolidine product was >99%.

FK(CHO)M(sulfoxide) thiazolidine. LCMS: *m/z* 556.22810 (calcd [M+H]+ = 556.2258), *m/z* 578.28747 (calcd [M+Na]+ = 578.2077), (HPLC analysis at 220 nm, **method A**). Retention time in HPLC: 5.985

HPLC Trace of reaction

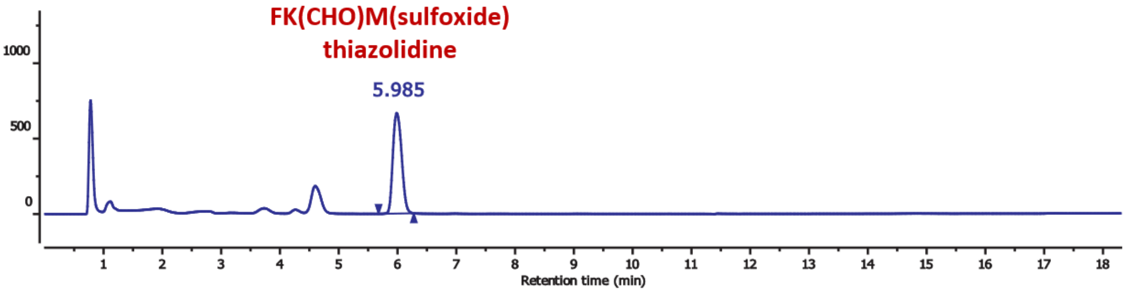

MS-Trace of peak 5.985 (FK(CHO)M(sulfoxide) thiazolidine)

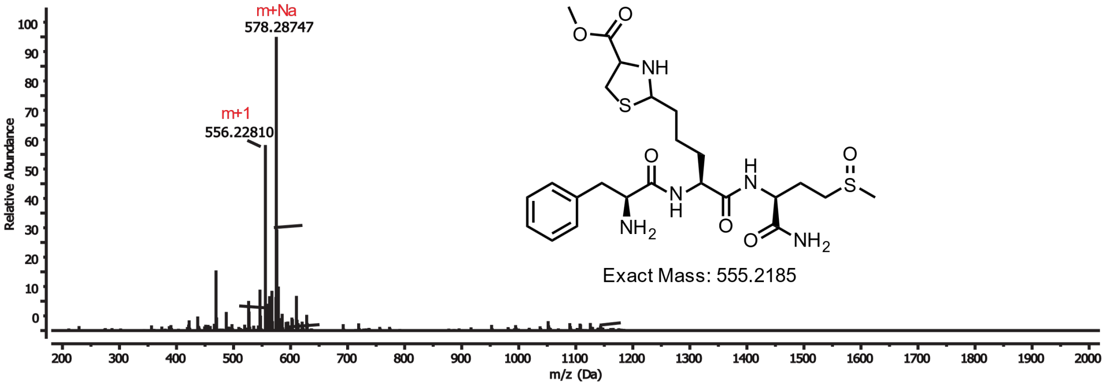

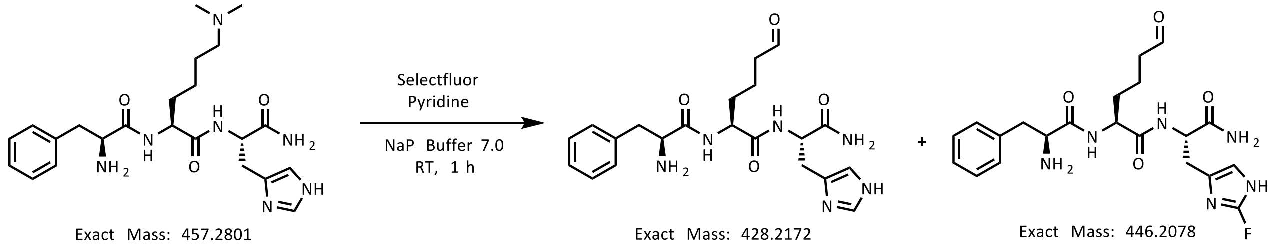

To 1.0 mg of FKme$_2$H peptide dissolved in 300 µL of 10 mM sodium phosphate buffer (NaP, pH 7.0), was added pyridine (5 eq) and selectfluor (2 eq). The reaction mixture was stirred for 1 h. Samples were taken from the reaction mixture, injected into LC-MS to monitor the generation of fluorinated peptide aldehyde product. The reaction mixture was analyzed by HPLC using method A and product peak purified by preparative HPLC.

FK(CHO)H. LCMS: *m/z* 429.20121 (calcd [M+H]+ = 429.2245), (HPLC analysis at 220 nm, **method A**). Retention time in HPLC: 6.346

FK(CHO)H(flourine). LCMS: *m/z* 447.26419 (calcd [M+H]+ = 447.2151), (HPLC analysis at 220 nm, **method A**). Retention time in HPLC: 7.686

HPLC Trace of reaction

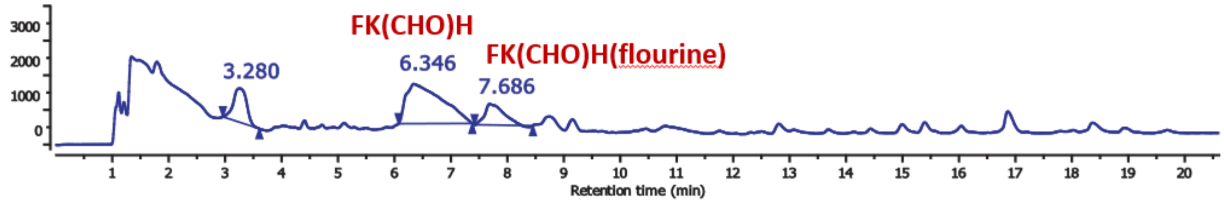

MS-Trace of peak 6.346 (FK(CHO)H)

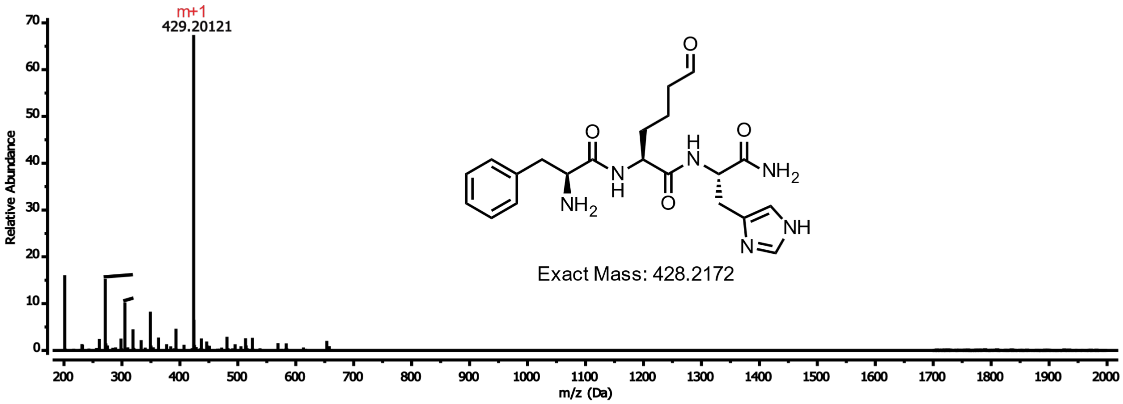

MS-Trace of peak 7.686 (FK(CHO)H(flourine))

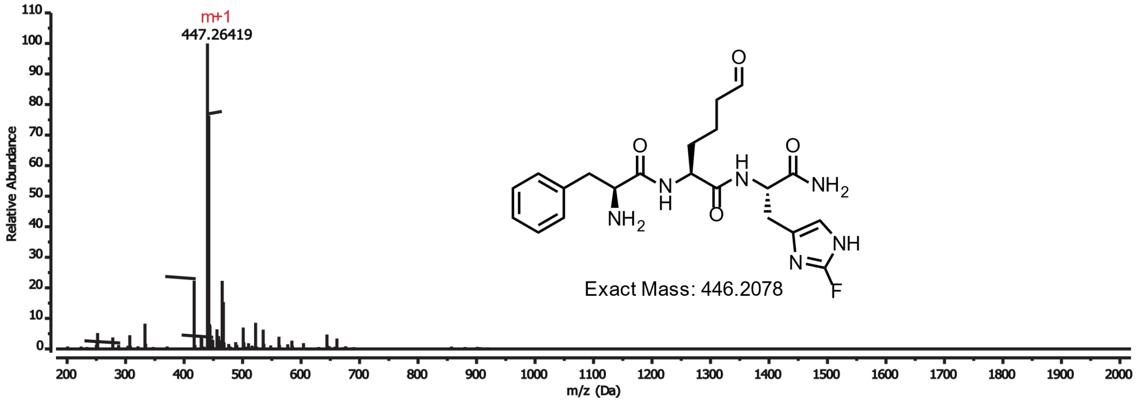

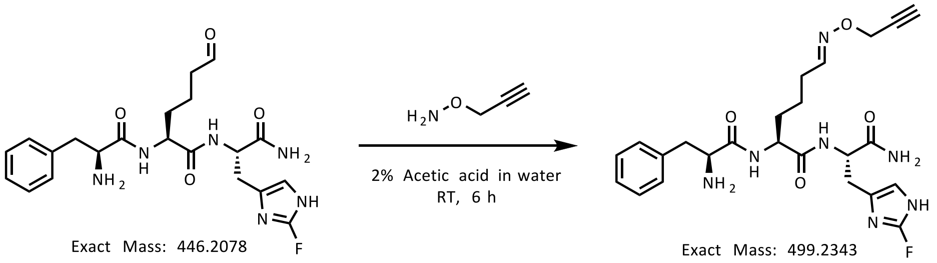

To 1 mg of aldehyde-flourinated peptide **FK(CHO)H(flourine)** in 300 µL of NaP buffer was added 4 equivalences of O-2-Propynylhydroxylamine hydrochloride. The reaction was stirred for 6 h. Samples were taken from the reaction mixture, injected into LC-MS to monitor the modification of the peptide **FK(CHO)H(flourine)** to the oxime propargyl peptide product. The reaction mixture was analyzed by HPLC using method B and the % conversion to the oxime propargyl peptide product (>98%).

FK(CHO)H(flourine) propargyl oxime. LCMS: *m/z* 500.27292 (calcd [M+H]+ = 500.2416), (HPLC analysis at 220 nm, **method A**). Retention time in HPLC: 6.549

HPLC Trace of reaction

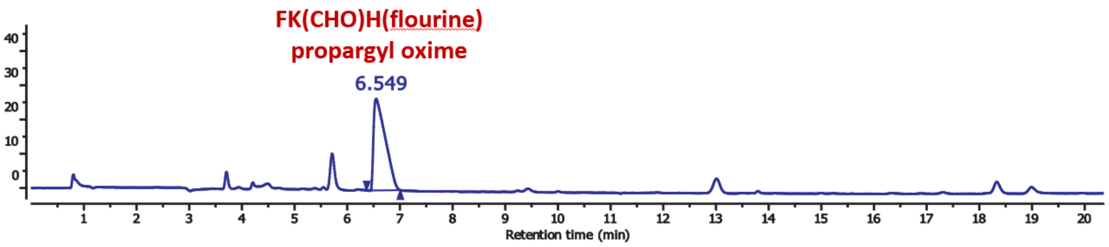

MS-Trace of peak 6.549 (FK(CHO)H(flourine) propargyl oxime)

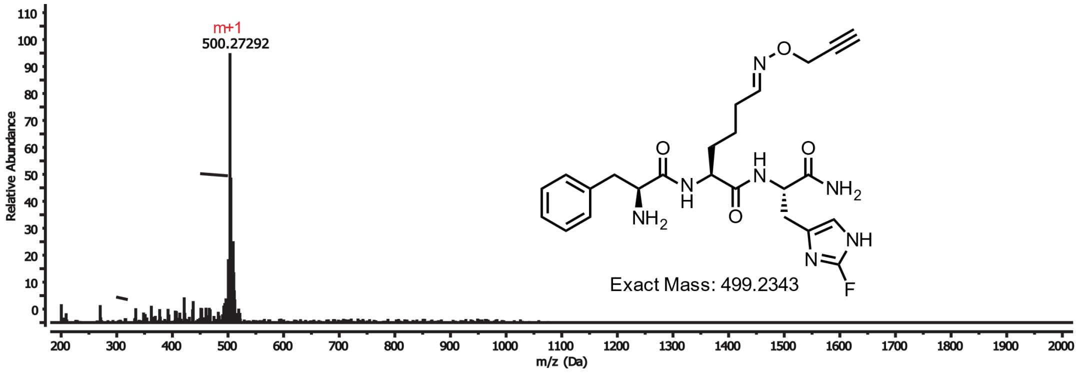

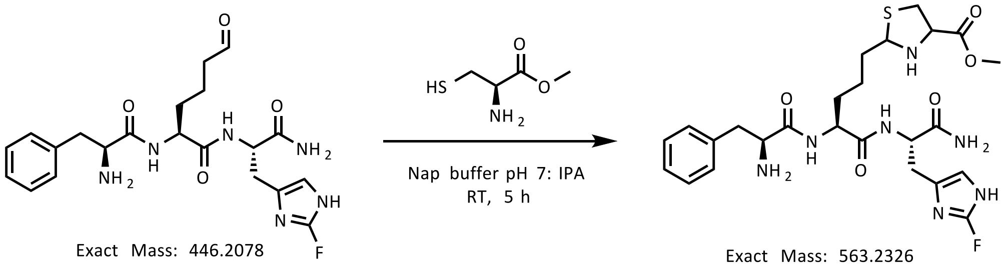

To 1 mg of aldehyde-flourinated peptide **FK(CHO)H(flourine)** in 300 µL of NaP buffer and isopropyl alcohol (IPA) was added 5 equivalences of cysteine methylester. Reaction was stirred at room temperature for 5 h. Samples were taken from the reaction mixture, injected into LC-MS to monitor the formation of thiazolidine. The reaction mixture was analyzed by HPLC using method A and % conversion to thiazolidine product was >99%.

FK(CHO)H(flourine) thiazolidine. LCMS: *m/z* 564.25421 (calcd [M+H]+ = 564.2399), *m/z* 1149.71489 (calcd [2M+H]+ = 1149.4545), (HPLC analysis at 220 nm, **method A**). Retention time in HPLC: 9.646

HPLC Trace of reaction

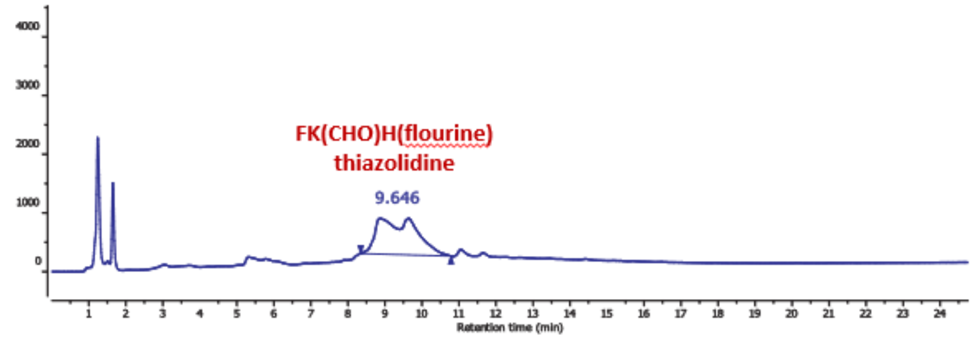

MS-Trace of peak 9.646 (FK(CHO)H(flourine) thiazolidine)

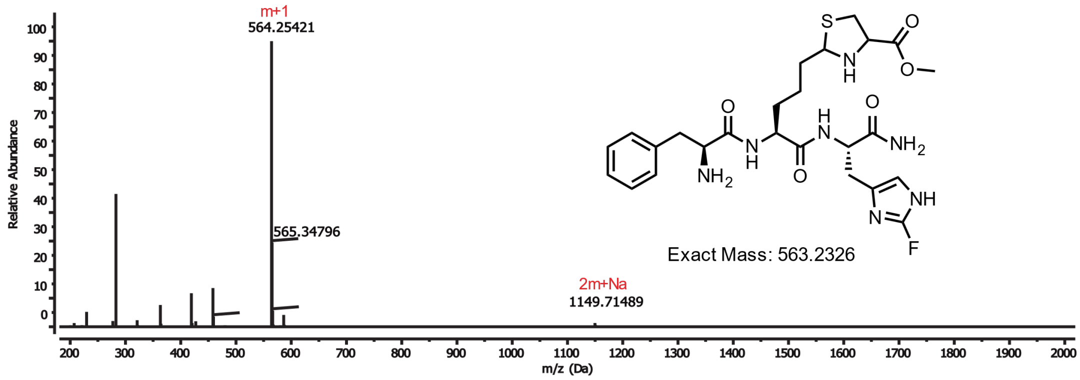

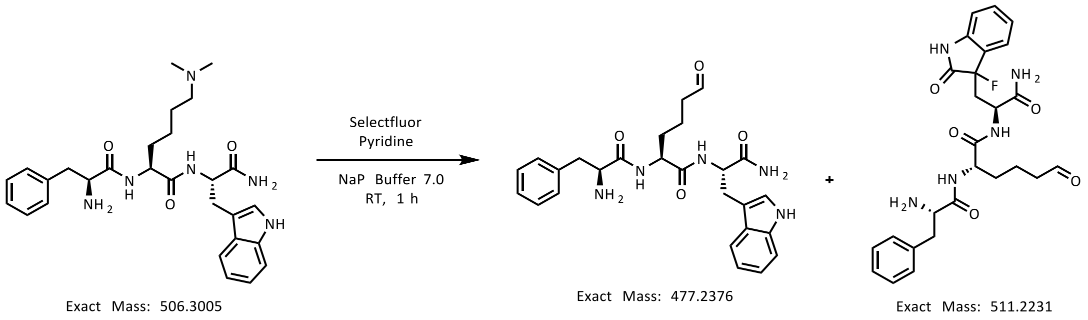

To 1.0 mg of FKme$_2$W peptide dissolved in 300 µL of 10 mM sodium phosphate buffer (NaP, pH 7.0), was added pyridine (5 eq) and selectfluor (2 eq). The reaction mixture was stirred for 1 h. Samples were taken from the reaction mixture, injected into LC-MS to monitor the generation of fluorinated peptide aldehyde product. The reaction mixture was analyzed by HPLC using method A and product peak purified by preparative HPLC.

FK(CHO)W. LCMS: *m/z* 478.35721 (calcd [M+H]+ = 478.2449), *m/z* 955.67990 (calcd [2M+H]+ = 955.4825), (HPLC analysis at 220 nm, **method A**). Retention time in HPLC: 4.300

FK(CHO)W(fluorine). LCMS: *m/z* 512.22616 (calcd [M+H]+ = 512.2304), *m/z* 256.53616 (calcd [M+2/2]+ = 256.6115), (HPLC analysis at 220 nm, **method A**). Retention time in HPLC: 5.773

HPLC Trace of reaction

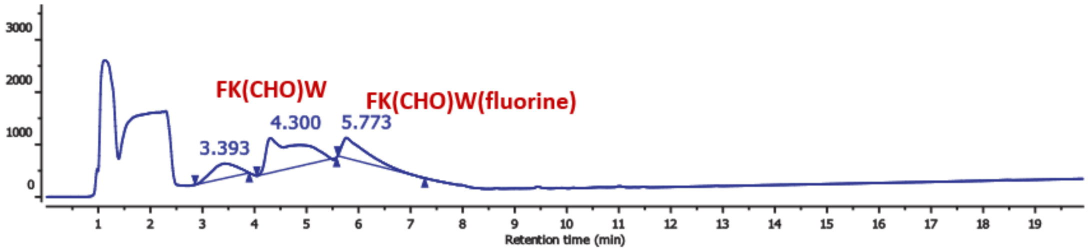

MS-Trace of peak 4.300 (FK(CHO)W)

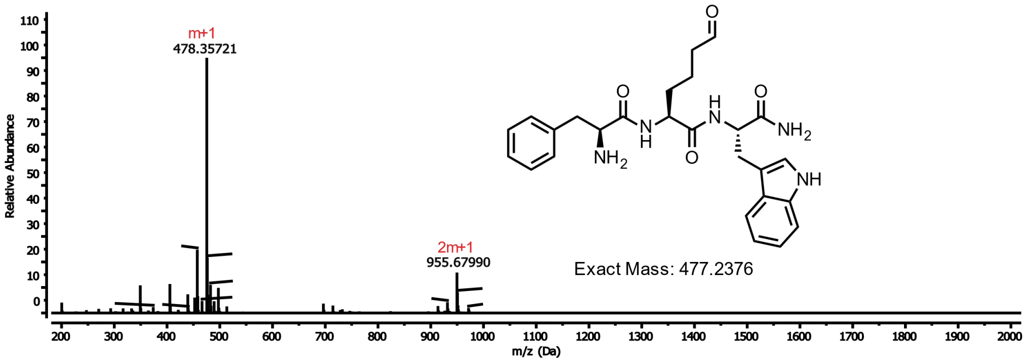

MS-Trace of peak 5.773 (FK(CHO)W(fluorine))

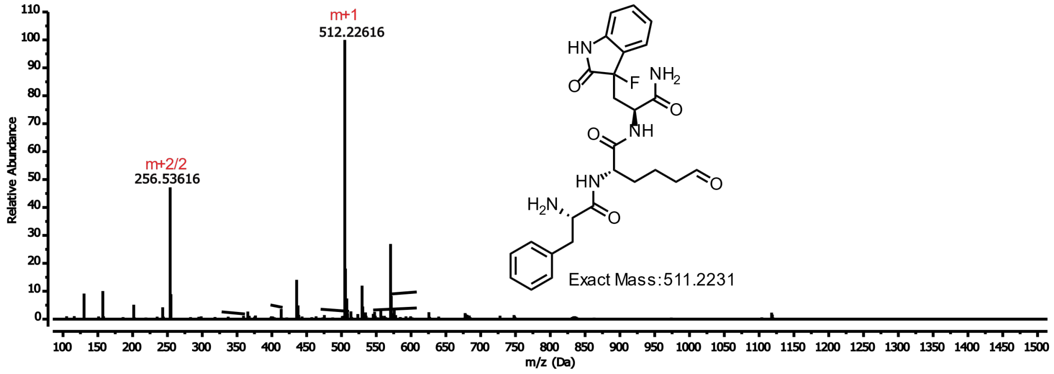

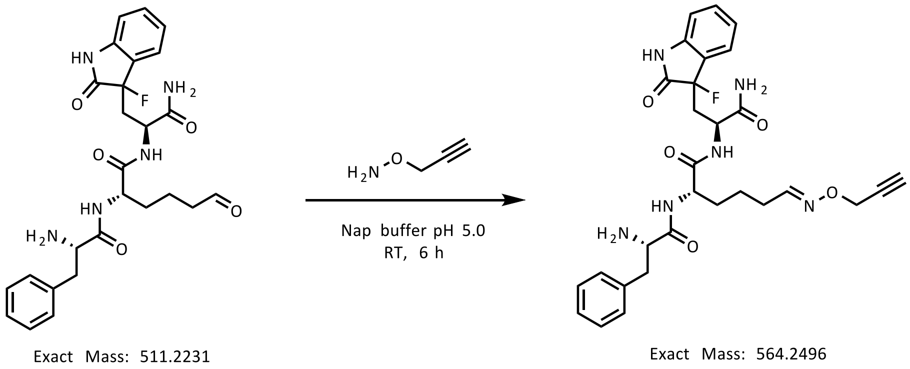

To 1 mg of aldehyde-flourinated peptide **FK(CHO)W(flourine)** in 300 µL of NaP buffer was added 4 equivalences of O-2-Propynylhydroxylamine hydrochloride. The reaction was stirred for 6 h. Samples were taken from the reaction mixture, injected into LC-MS to monitor the modification of the peptide **FK(CHO)W(flourine)** to the oxime propargyl peptide product. The reaction mixture was analyzed by HPLC using method B and the % conversion to the oxime propargyl peptide product (>98%).

FK(CHO)W(flourine) propargyl oxime. LCMS: *m/z* 565.42033 (calcd [M+H]+ = 565.2569), (HPLC analysis at 220 nm, **method A**). Retention time in HPLC: 9.216

HPLC Trace of reaction

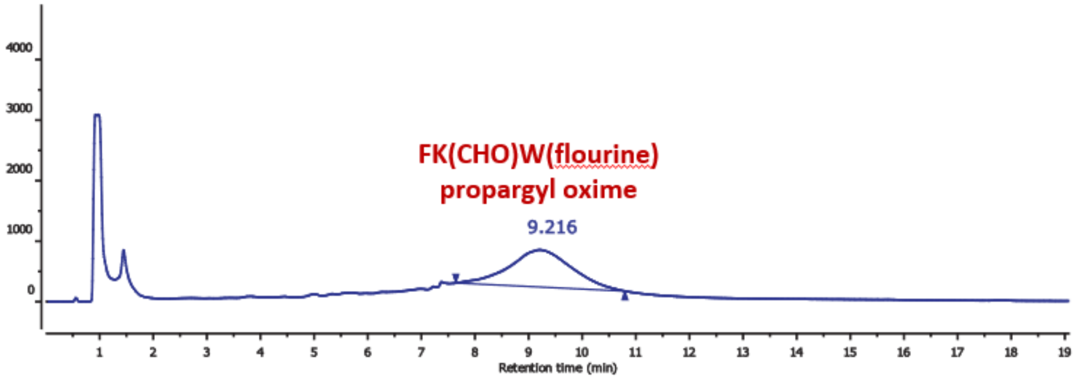

MS-Trace of peak 9.216 (FK(CHO)W(flourine) propargyl oxime)

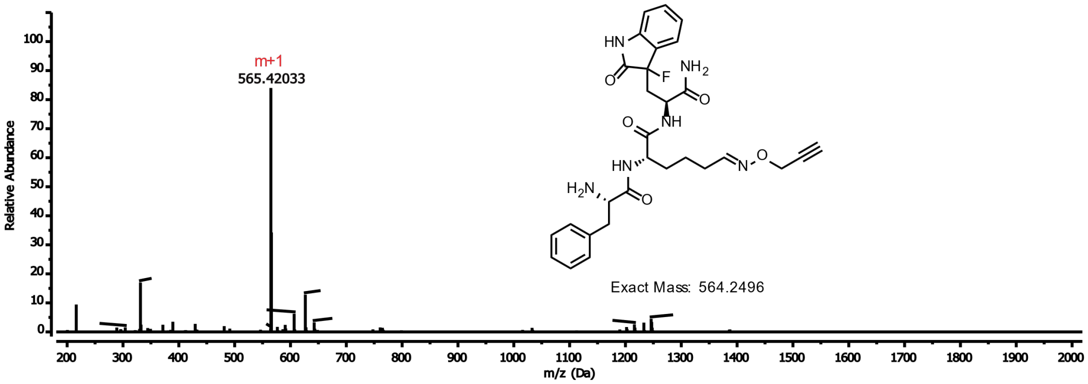

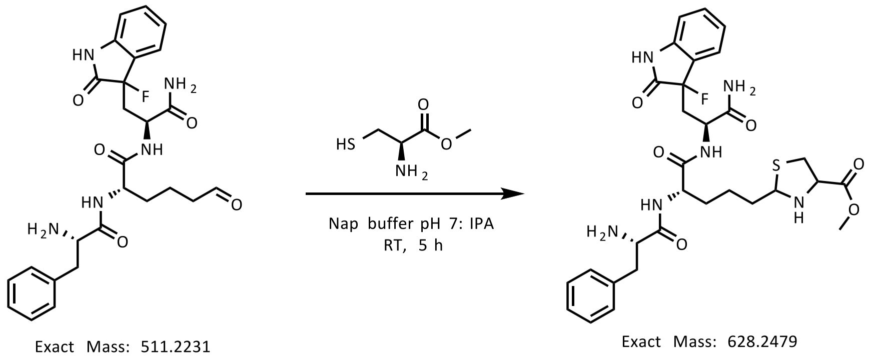

To 1 mg of aldehyde-flourinated peptide **FK(CHO)W(flourine)** in 300 µL of NaP buffer and isopropyl alcohol (IPA) was added 5 equivalences of cysteine methylester. Reaction was stirred at room temperature for 5 h. Samples were taken from the reaction mixture, injected into LC-MS to monitor the formation of thiazolidine. The reaction mixture was analyzed by HPLC using method A and % conversion to thiazolidine product was >99%.

FK(CHO)W(flourine) thiazolidine. LCMS: *m/z* 629.30498 (calcd [M+H]+ = 629.2552), *m/z* 1257.04556 (calcd [2M+H]+ = 1257.5031), *m/z* 1149.71489 (calcd [2M+H]+ = 1149.4545), (HPLC analysis at 220 nm, **method A**). Retention time in HPLC: 12.083

HPLC Trace of reaction

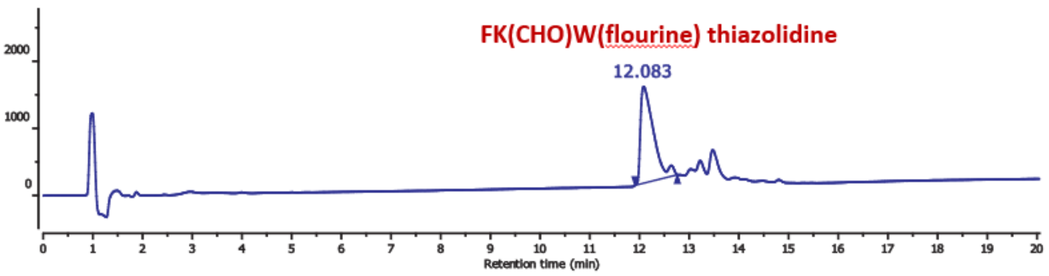

MS-Trace of peak 12.083 (FK(CHO)W(flourine) thiazolidine)

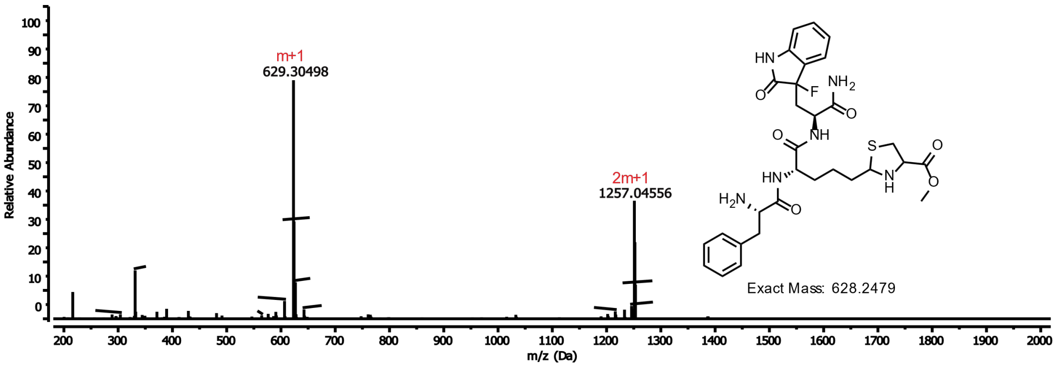

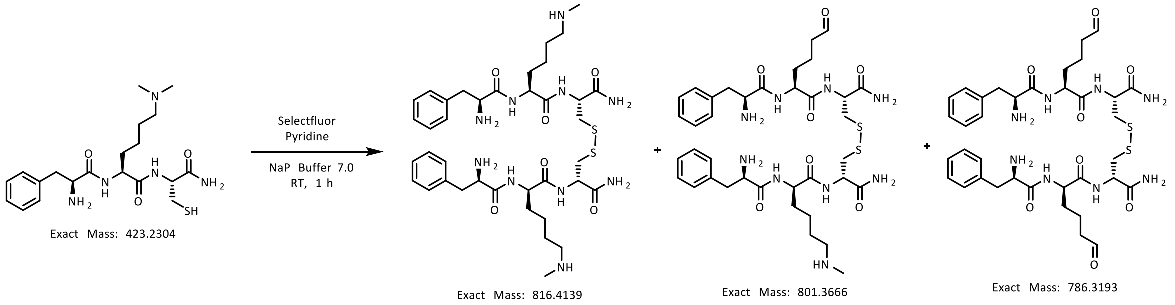

To 1.0 mg of FKme$_2$C peptide dissolved in 300 µL of 10 mM sodium phosphate buffer (NaP, pH 7.0), was added pyridine (5 eq) and selectfluor (2 eq). The reaction mixture was stirred for 1 h. Samples were taken from the reaction mixture, injected into LC-MS to monitor the generation of peptide aldehyde disulfide product. The reaction mixture was analyzed by HPLC using method A and product peak purified by preparative HPLC.

FK(Kme)(Kme)C(disulfide). LCMS: *m/z* 817.39377 (calcd [M+H]+ = 817.4211), *m/z* 839.96313 (calcd [M+Na]+ = 839.4031), (HPLC analysis at 220 nm, **method A**). Retention time in HPLC: 5.491

FK(CHO)(Kme)C(disulfide). LCMS: *m/z* 802.15983 (calcd [M+H]+ = 802.3739), *m/z* 1063.22263 (calcd [2M+H]+ = 1603.3404), (HPLC analysis at 220 nm, **method A**). Retention time in HPLC: 6.038

FK(CHO)(CHO)C(disulfide). LCMS: *m/z* 787.34160 (calcd [M+H]+ = 787.3266), *m/z* 809.30939 (calcd [M+Na]+ = 809.3085), *m/z* 394.10974 (calcd [M+2/2]+ = 394.1596), (HPLC analysis at 220 nm, **method A**). Retention time in HPLC: 7.604

HPLC Trace of reaction

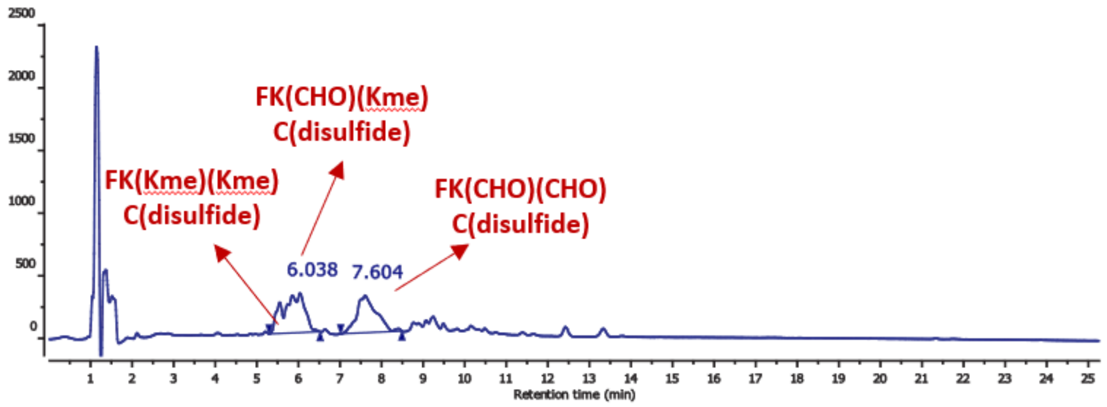

MS-Trace of peak 5.491

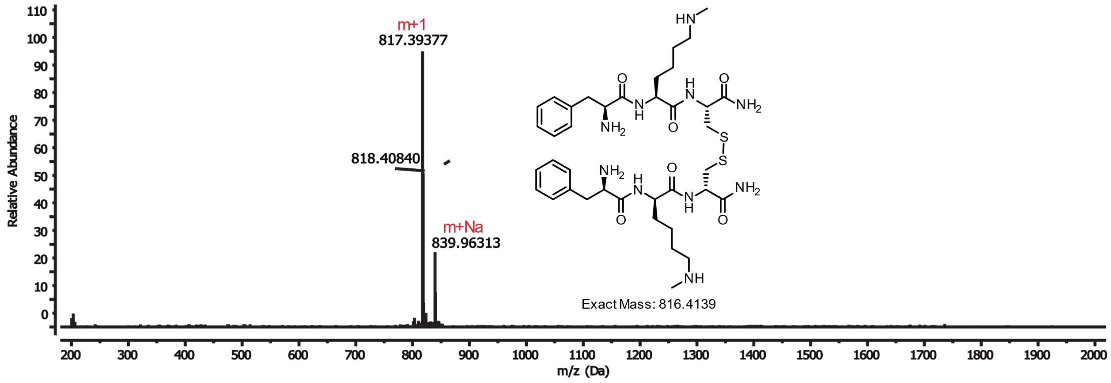

MS-Trace of peak 6.038

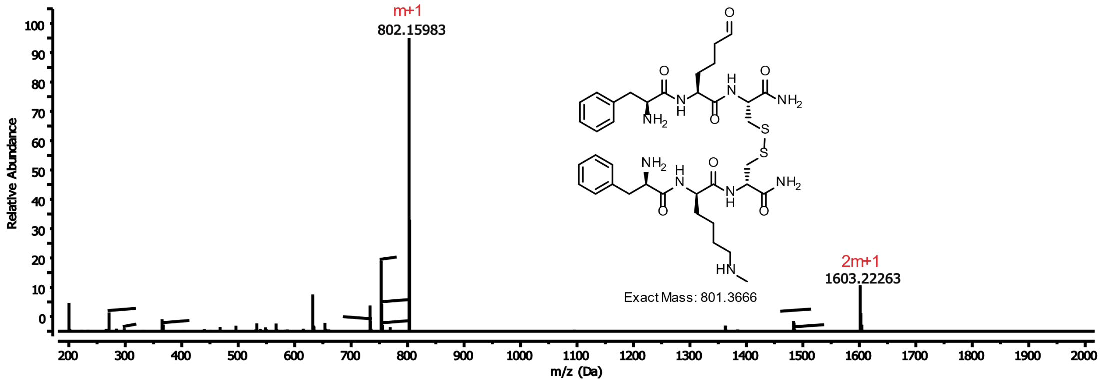

MS-Trace of peak 7.604

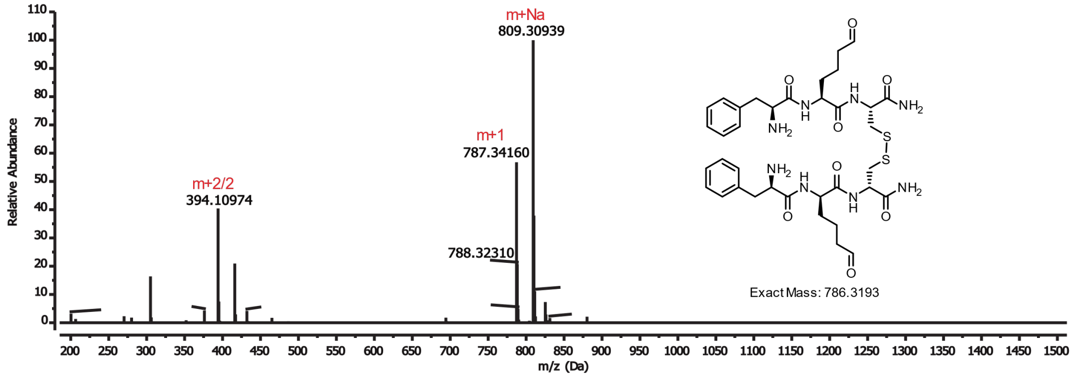

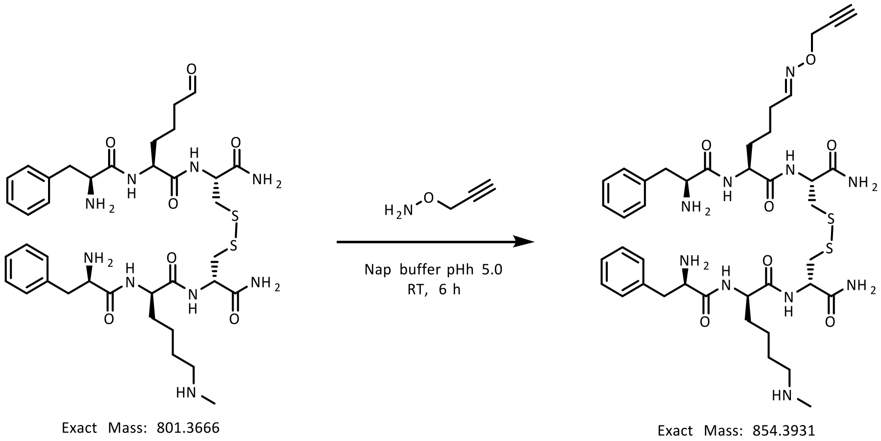

To 1 mg of aldehyde-disulfide peptide **FK(CHO)(Kme)C(disulfide)** in 300 µL of NaP buffer was added 4 equivalences of O-2-Propynylhydroxylamine hydrochloride. The reaction was stirred for 6 h. Samples were taken from the reaction mixture, injected into LC-MS to monitor the modification of the peptide **FK(CHO)(Kme)C(disulfide)** to the oxime propargyl peptide product. The reaction mixture was analyzed by HPLC using method B and the % conversion to the oxime propargyl peptide product (>98%).

FK(CHO)(Kme)C(disulfide) propargyl oxime. LCMS: *m/z* 855.37139 (calcd [M+H]+ = 855.4004), (HPLC analysis at 220 nm, **method A**). Retention time in HPLC: 10.525

HPLC Trace of reaction

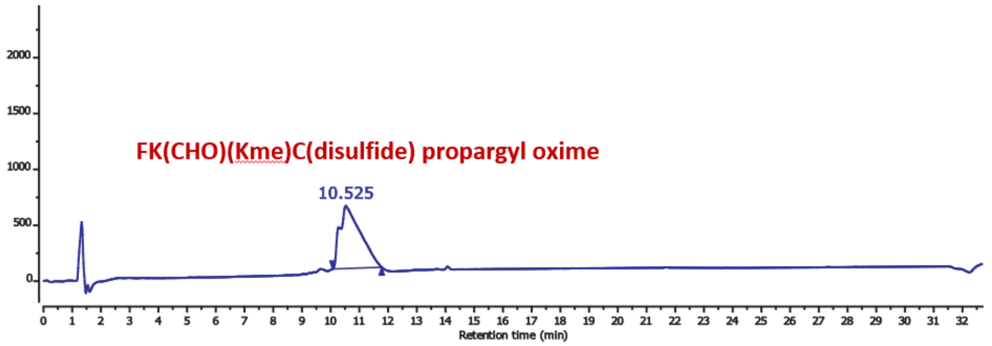

MS-Trace of peak 10.525

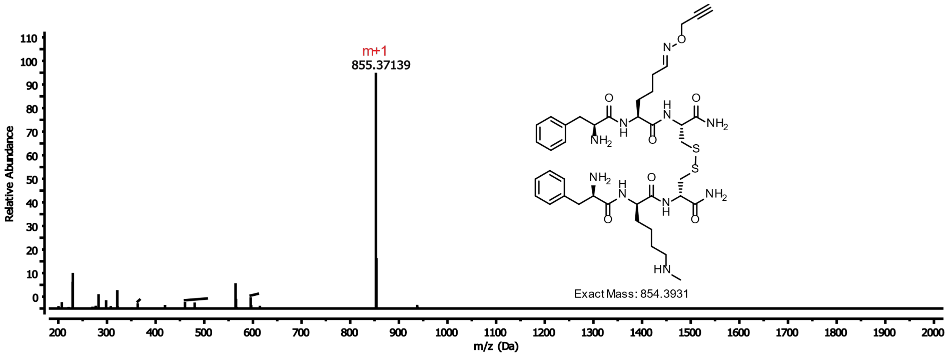

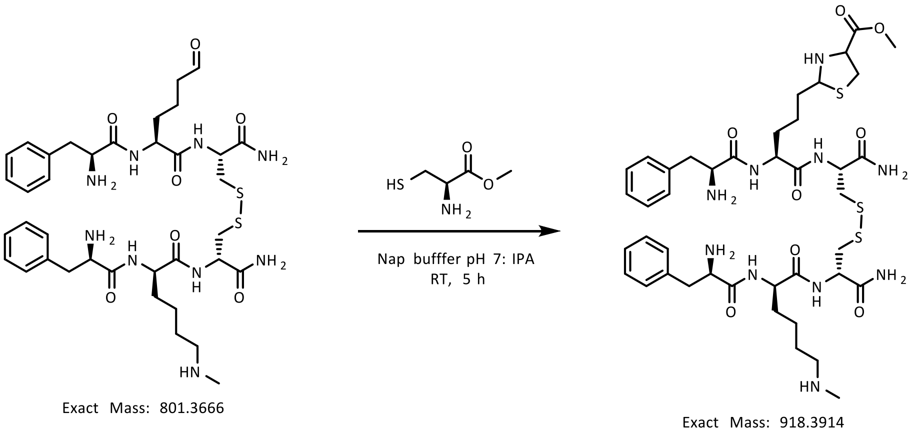

To 1 mg of aldehyde-disulfide peptide **FK(CHO)(Kme)C(disulfide)** in 300 µL of NaP buffer and isopropyl alcohol (IPA) was added 5 equivalences of cysteine methylester. Reaction was stirred at room temperature for 5 h. Samples were taken from the reaction mixture, injected into LC-MS to monitor the formation of thiazolidine. The reaction mixture was analyzed by HPLC using method A and % conversion to thiazolidine product was >99%.

FK(CHO)(Kme)C(disulfide) thiazolidine. LCMS: *m/z* 919.40159 (calcd [M+H]+ = 919.3987), *m/z* 941.45094 (calcd [M+Na]+ = 941.3804), *m/z* 460.15611 (calcd [M+2/2]+ = 460.2030), (HPLC analysis at 220 nm, **method A**). Retention time in HPLC: 13.308

HPLC Trace of reaction

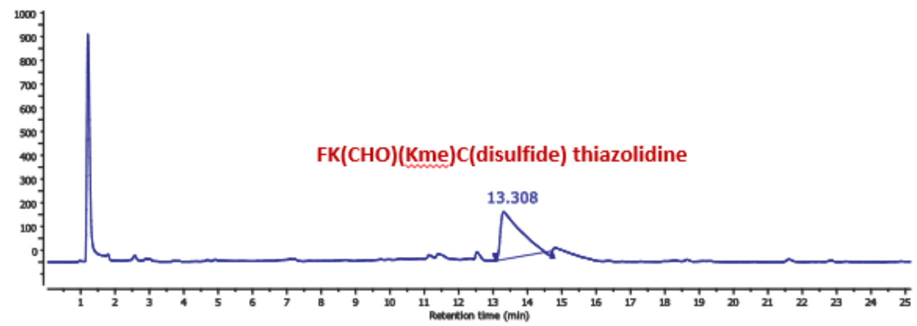

MS-Trace of peak 13.308

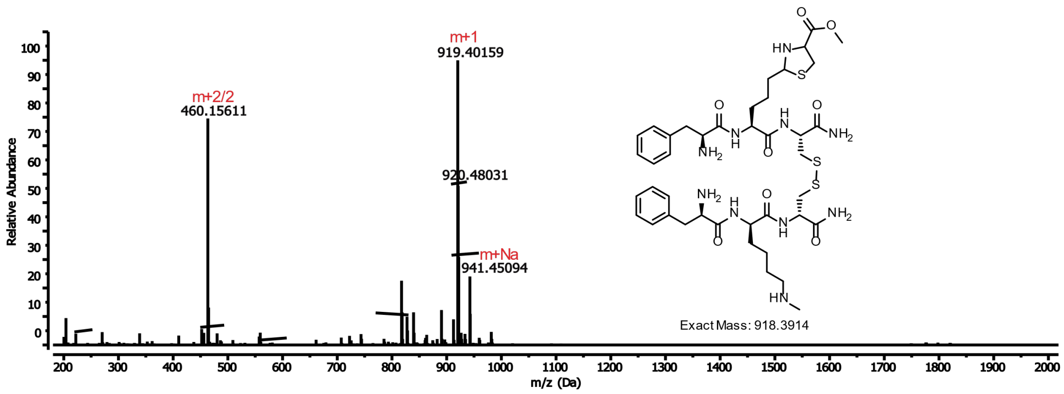

XIII. Supplementary Figure 7: Pan-specificity evaluation of dimethyllysine containing histone peptide fragments to generate histone peptide aldehydes.

$Kme_2$4K9 (ARTKme_2QTARKS) 1c:

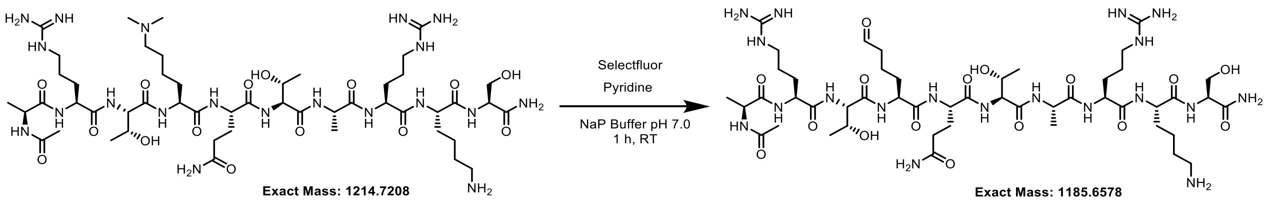

1c

2c (>95%)

To 1.0 mg of histone peptide fragment $Kme_2$4K9 (ARTKme_2QTARKS) **1c** dissolved in 300 µL of 10 mM sodium phosphate buffer (NaP, pH 7.0), was added pyridine (5 eq) and selectfluor (2 eq). The reaction mixture was stirred for 1 h. Samples were taken from the reaction mixture, injected into LC-MS to monitor the generation of histone peptide aldehyde product **2c**. The reaction mixture was analyzed by HPLC using method B and % conversion to peptide aldehyde **2c** was >95%.

$Kme_2$4K9 (ARTKme_2QTARKS) 1c. LCMS: *m/z* 1215.72037 (calcd [M+H]+ = 1215.7280), *m/z* 608.36361 (calcd [M+2/2]+ = 608.3641), *m/z* 405.91159 (calcd [M+3/3]+ = 405.9093), *m/z* 304.68563 (calcd [M+4/4]+ = 304.6826).

Kme_2(CHO)4K9 (ARTKme_2(CHO)QTARKS) 2c. LCMS: *m/z* 1186.65866 (calcd [M+H]+ = 1186.6651), *m/z* 593.83341 (calcd [M+2/2]+ = 593.8326), *m/z* 396.21955 (calcd [M+3/3]+ = 396.2217), (HPLC analysis at 220 nm, **method B**). Retention time in HPLC: 6.421

HPLC Trace of reaction

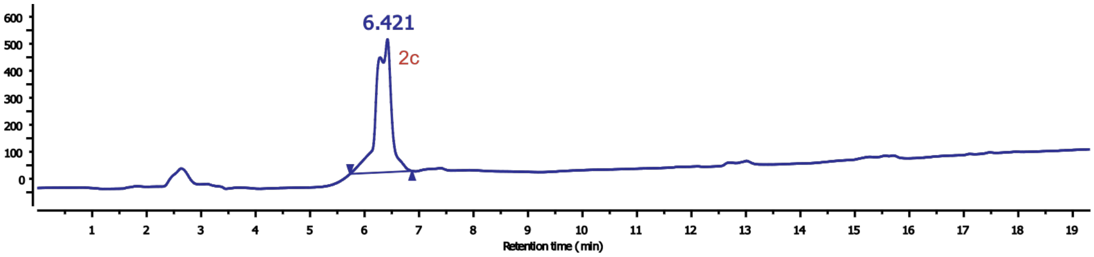

MS-Trace of starting histone peptide fragment 1c

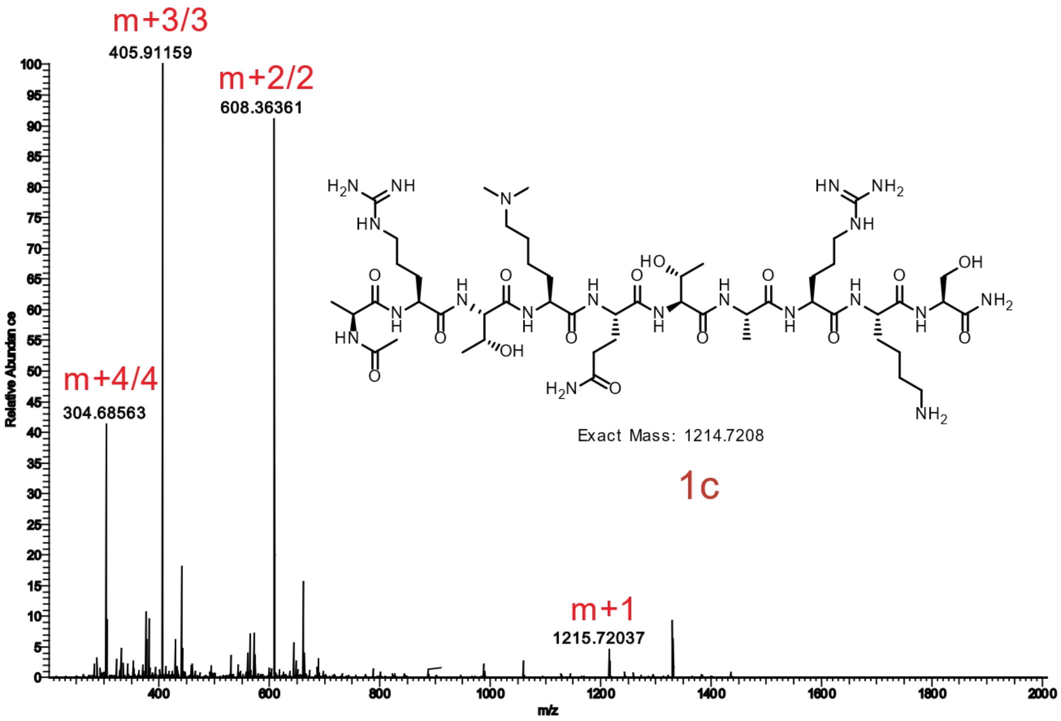

MS-Trace of histone aldehyde peptide product 2c

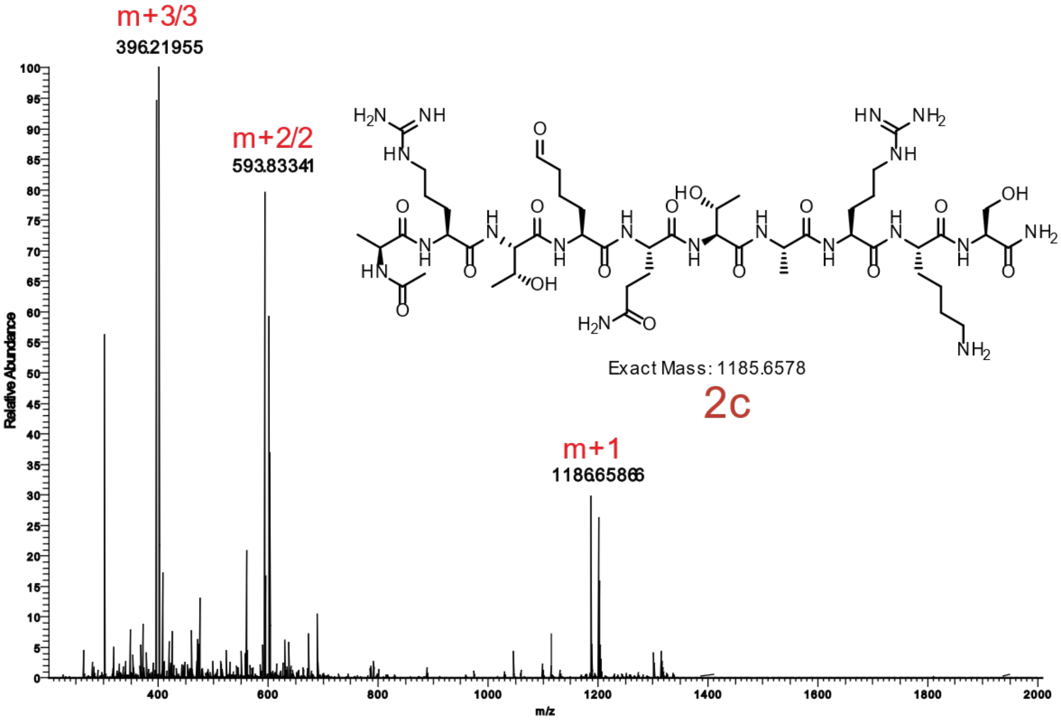

Modification of histone peptide fragment 1d:

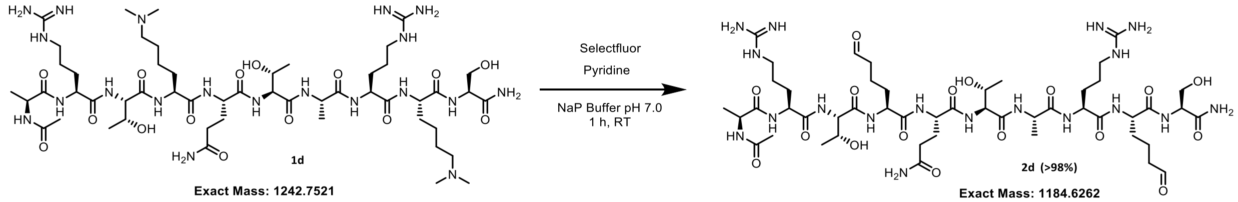

To 1.0 mg of histone peptide fragment $Kme_2$4$Kme_2$9 (ARTKme_2QTARKme_2S) **1d** dissolved in 300 μL of 10 mM sodium phosphate buffer (NaP, pH 7.0), was added pyridine (5 eq) and selectfluor (2 eq). The reaction mixture was stirred for 1 h. Samples were taken from the reaction mixture, injected into LC-MS to monitor the generation of histone peptide aldehyde product **2d**. The reaction mixture was analyzed by HPLC using method B and % conversion to peptide aldehyde **2d** was >98%.

$Kme_2$4$Kme_2$9 (ARTKme_2QTARKme_2S) 1d. LCMS: *m/z* 1243.75401 (calcd [M+H]+ = 1243.7593), *m/z* 622.37994 (calcd [M+2/2]+ = 622.3796), *m/z* 415.25601 (calcd [M+3/3]+ = 415.2531), *m/z* 311.69388 (calcd [M+4/4]+ = 311.6898).

Kme_2(CHO)4Kme_2(CHO)9 (ARTKme_2(CHO)QTARKme_2(CHO)S) 2d. LCMS: *m/z* 1185.62600 (calcd [M+H]+ = 1185.6335), *m/z* 593.31680 (calcd [M+2/2]+ = 593.3167), (HPLC analysis at 220 nm, **method B**). Retention time in HPLC: 8.768

HPLC Trace of reaction

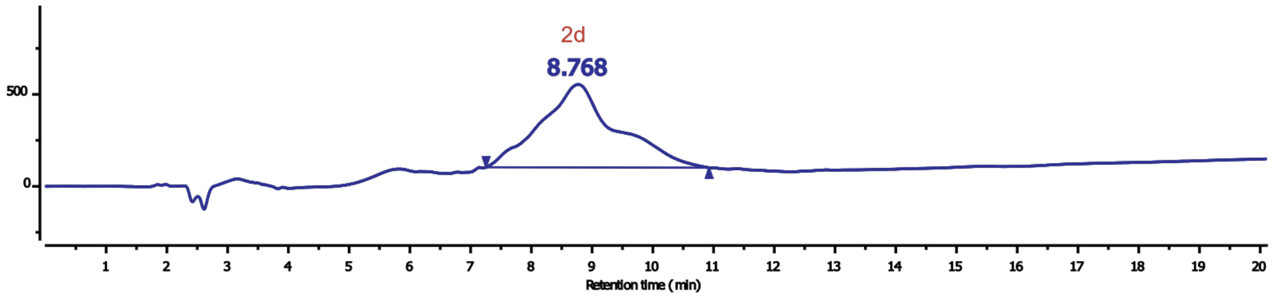

MS-Trace of 1d

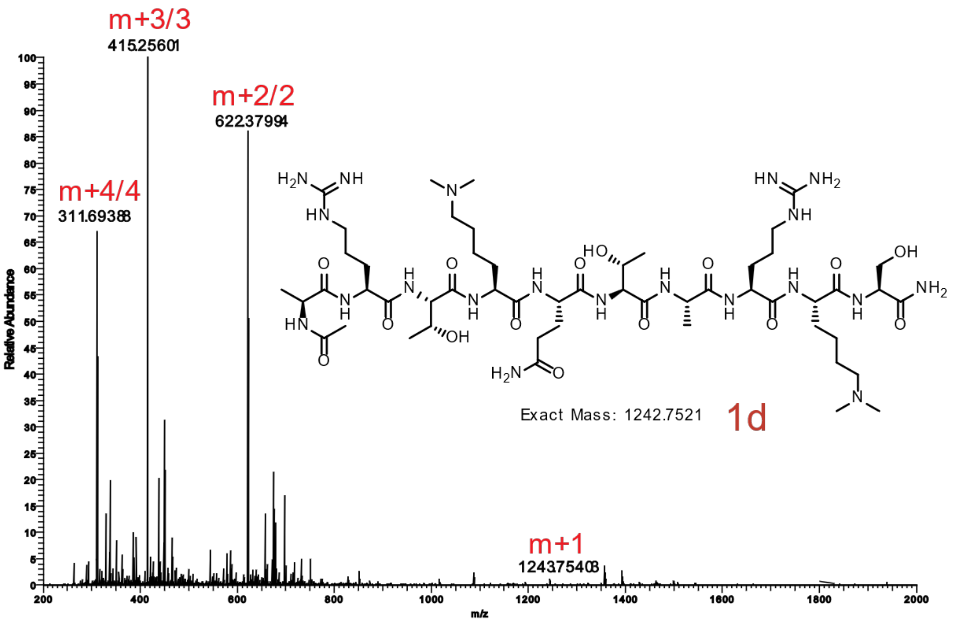

MS-Trace of product 2d

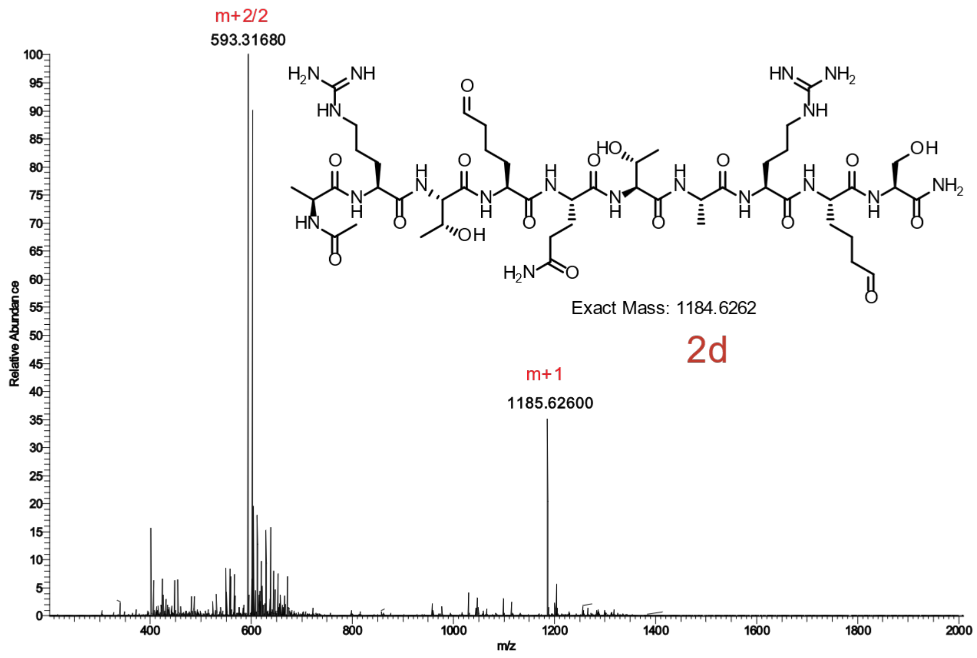

Modification of histone peptide fragment 1e:

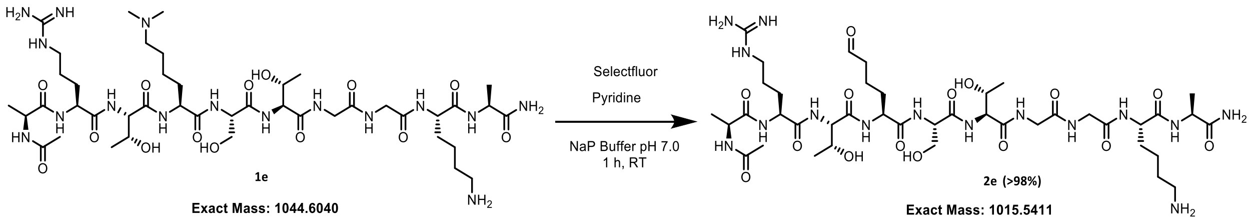

To 1.0 mg of $Kme_2$9K14 (ARTKme_2STGGKA) **1e** dissolved in 300 µL of 10 mM sodium phosphate buffer (NaP, pH 7.0), was added pyridine (5 eq) and selectfluor (2 eq). The reaction mixture was stirred for 1 h. Samples were taken from the reaction mixture, injected into LC-MS to monitor the generation of histone peptide aldehyde product Kme_2(CHO)9K14 (ARTKme_2(CHO)STGGKA) **2e**. The reaction mixture was analyzed by HPLC using method B and % conversion to peptide aldehyde **2e** was >98%.

Linear peptide of histone peptide fragment 1e. LCMS: *m/z* 1045.60359 (calcd [M+H]+ = 1045.6113), *m/z* 523.30550 (calcd [M+2/2]+ = 523.30565), *m/z* 349.20630 (calcd [M+3/3]+ = 349.2037).

Histone aldehyde peptide product 2e. LCMS: *m/z* 1016.54144 (calcd [M+H]+ = 1016.5483), *m/z* 508.77432 (calcd [M+2/2]+ = 508.7741), (HPLC analysis at 220 nm, **method B**). Retention time in HPLC: 7.239

HPLC Trace of reaction

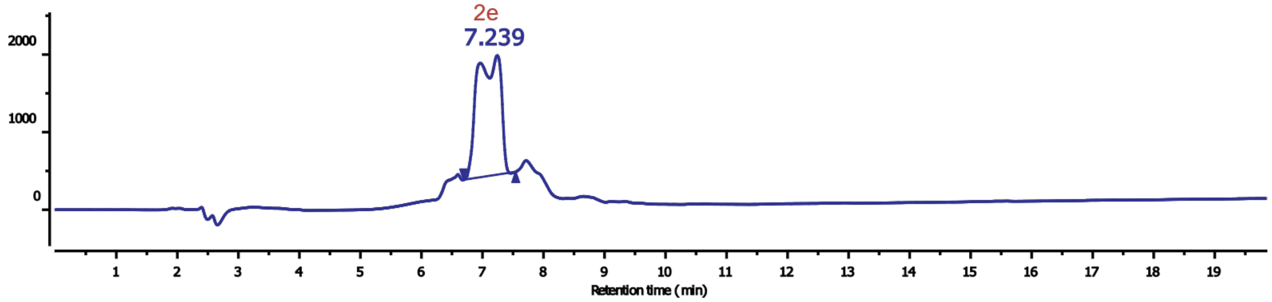

MS-Trace of 1e

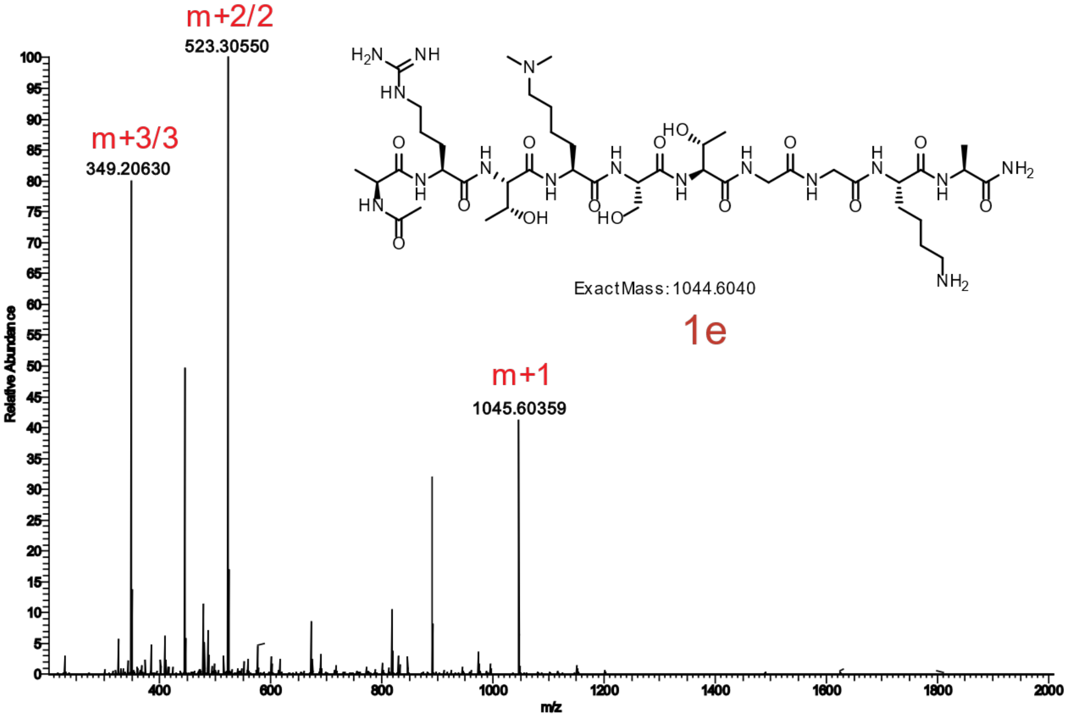

MS-Trace of product 2e

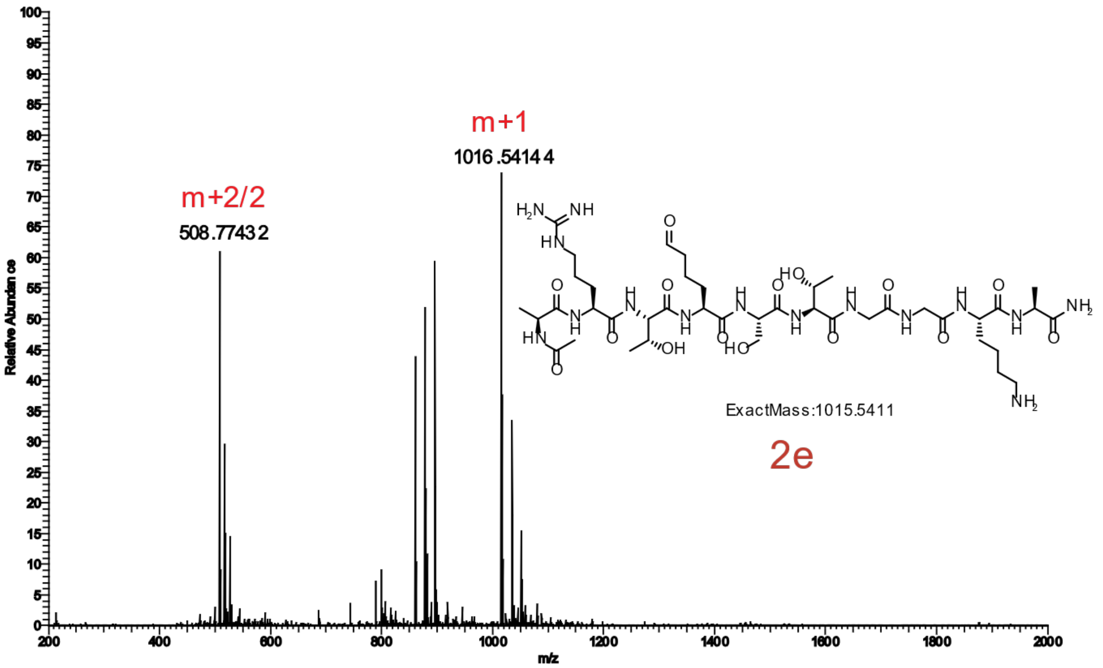

Modification of peptide fragment 1f:

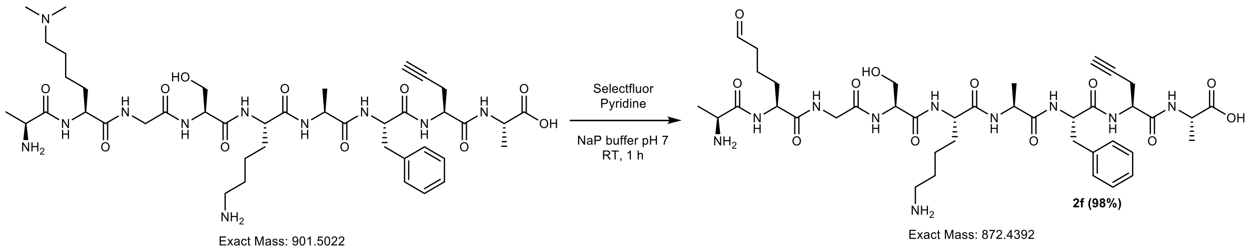

Linear peptide 1f: To 1.0 mg of a peptide fragment AKme$_2$GSKAF(PrG)A (where PrG = propargyl glycine) dissolved in 300 μL of 10 mM sodium phosphate buffer (NaP, pH 7.0), was added pyridine (5 eq) and selectfluor (2 eq). The reaction mixture was stirred for 1 h. The reaction mixture was analyzed by HPLC using method A and % conversion to aldehyde product **2f** (>98%).

Peptide aldehyde product 2f. LCMS: *m/z* 873.43885 (calcd [M+H]+ = 873.4392), *m/z* 895.59729 (calcd [M+Na]+ = 895.4284), (HPLC analysis at 220 nm). Retention time in HPLC: 9.170

HPLC Trace of peptide aldehyde product 2f

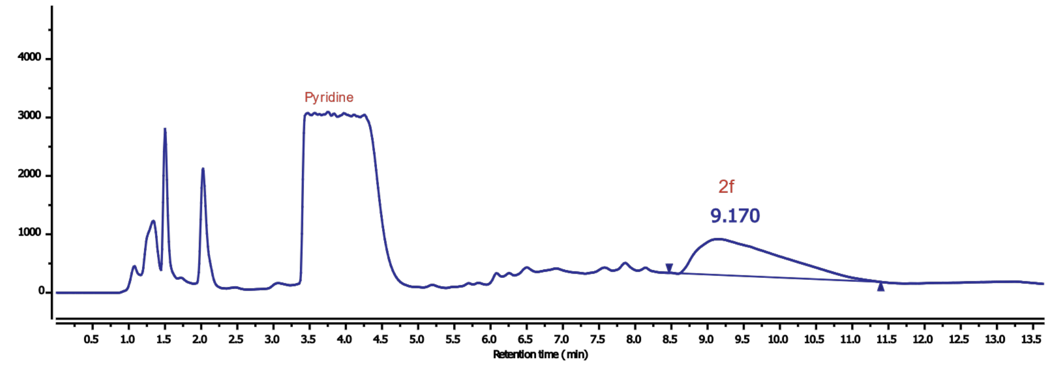

MS-Trace of peak 9.170

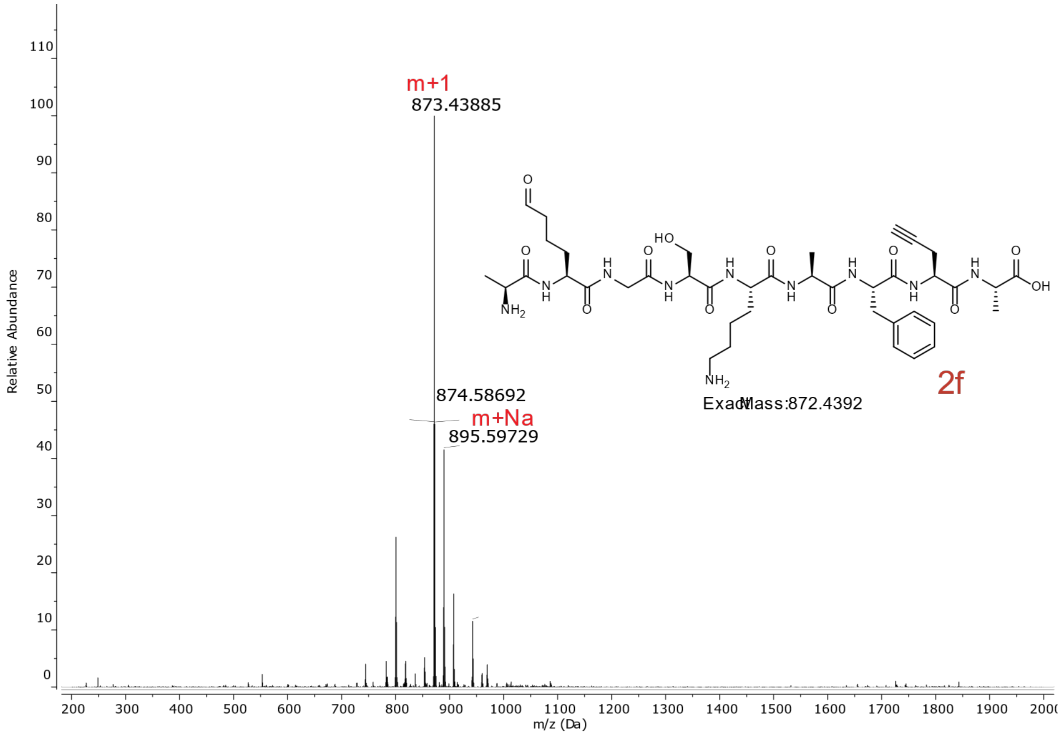

Modification of histone negative control peptide fragment 1g without Kme_2:

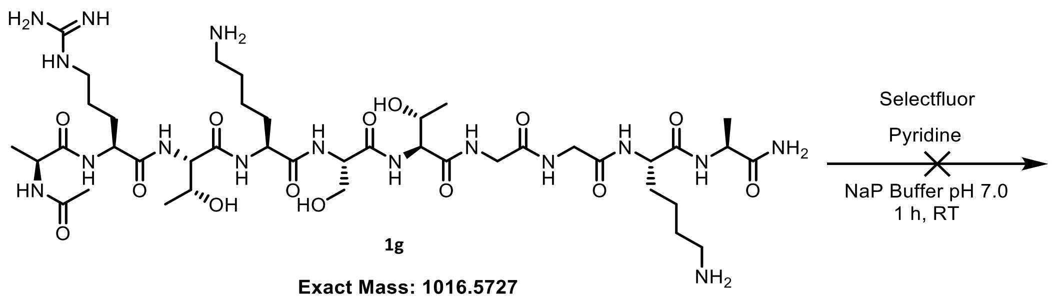

1g

Exact Mass: 1016.5727

To 1.0 mg of histone peptide fragment, ARTKQTARKS **1g** dissolved in 300 µL of 10 mM sodium phosphate buffer (NaP, pH 7.0), was added pyridine (5 eq) and selectfluor (2 eq). The reaction mixture was stirred for 1 h. The reaction mixture was analyzed by HPLC using method B. No modification of peptide **1g** was observed.

K4K9 (ARTKQTARKS) 1g. LCMS: *m/z* 1017.57249 (calcd [M+H]+ = 1017.5800), *m/z* 509.28979 (calcd [M+2/2]+ = 509.2900), *m/z* 339.86256 (calcd [M+3/3]+ = 339.8600), (HPLC analysis at 220 nm, **method B**). Retention time in HPLC: 6.253

HPLC Trace of reaction

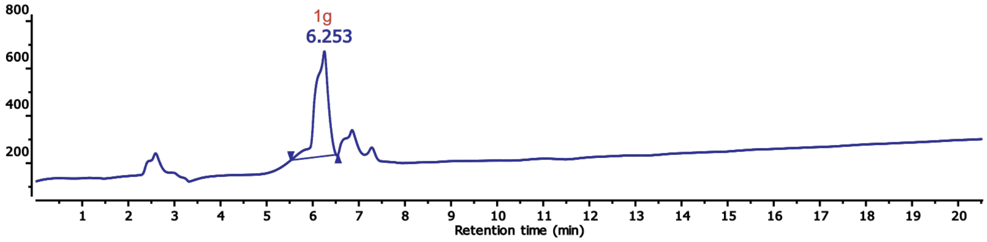

MS-Trace of peak 6.253: unreacted control peptide 1g

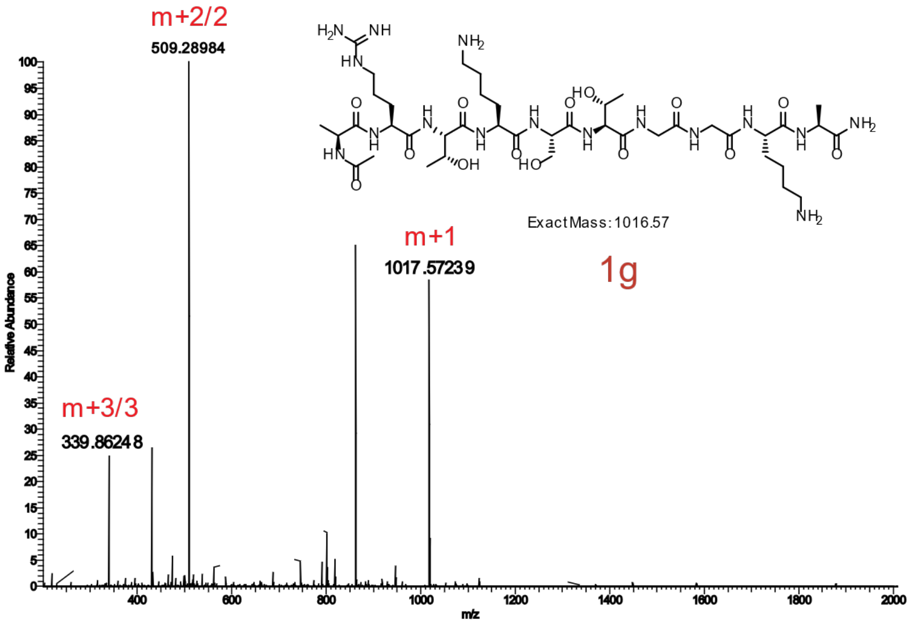

XIV. Supplementary Figure 8: Derivatization of di-aldehyde ARTKme$_2$(CHO)QTARKme$_2$(CHO)S **2d** with benzyl hydroxylamine to generate oxime adducts.

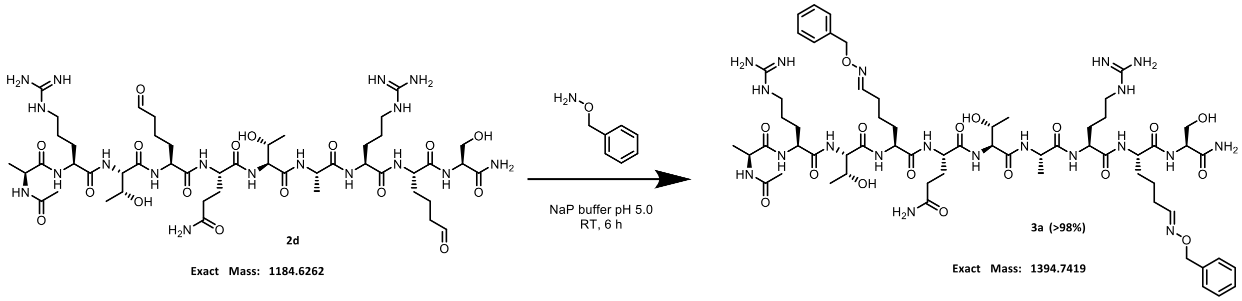

To 1 mg of aldehyde peptide **2d** in 300 µL of NaP buffer was added 4 equivalences of o-benzylhydroxylamine. The reaction was stirred for 6 h. The samples were taken from the reaction mixture, injected into LC-MS to monitor the modification of the peptide **2d** to the oxime peptide product **3a**. The reaction mixture was analyzed by HPLC using method B and the % conversion to the double oxime peptide product **3a** (>98%).

Kme$_2$(CHO)4Kme$_2$(CHO)9 (ARTKme$_2$(CHO)QTARKme$_2$(CHO)S) 2d. LCMS: *m/z* 1185.62600 (calcd [M+H]+ = 1185.6335), *m/z* 593.31680 (calcd [M+2/2]+ = 593.3167), (HPLC analysis at 220 nm). Retention time in HPLC: 8.768

Doubly modified oxime histone peptide product 3a. LCMS: *m/z* 1395.73547 (calcd [M+H]+ = 1395.7592), *m/z* 698.37195 (calcd [M+2/2]+ = 698.3796), (HPLC analysis at 220 nm). Retention time in HPLC: 11.339

Benzylhydroxylamine. (HPLC analysis at 220 nm). Retention time in HPLC: 4.754

HPLC Trace of oxime formation reaction

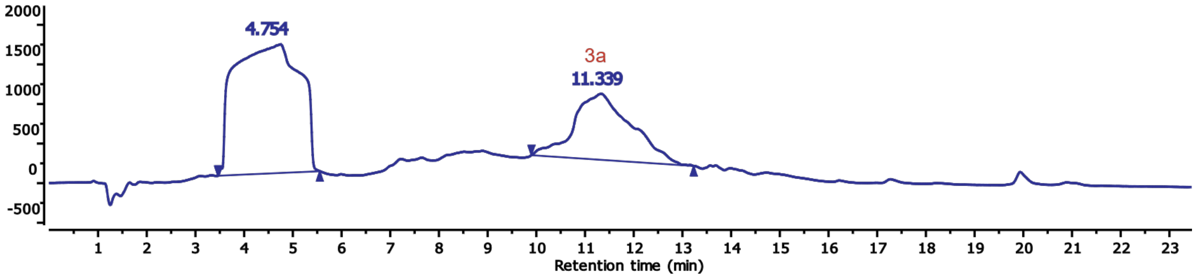

MS-Trace of peak 11.339

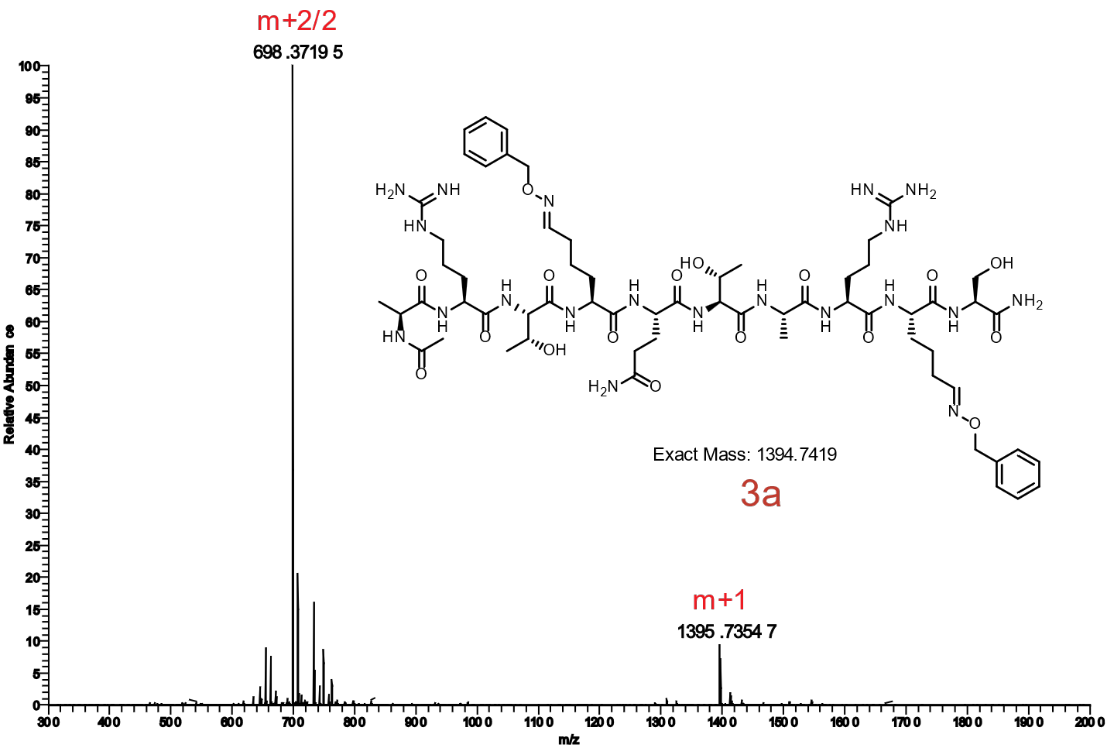

XV. Supplementary Figure 9: Reductive amination of dialdehyde peptide ARTKme$_2$(CHO)QTARKme$_2$(CHO)S **2d** with propargylamine and sodium cyanoborohydride.

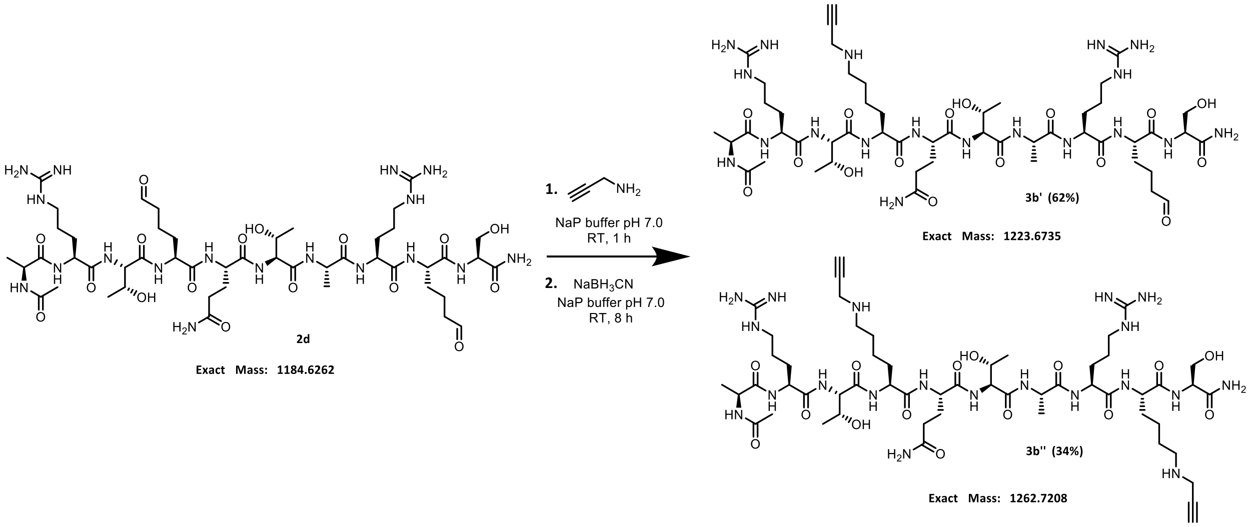

To 1 mg of aldehyde peptide **2d** in 300 µL of NaP buffer was added 5 equivalences of propargylamine. The reaction was stirred for 1 h, followed by the addition of 10 equivalence of sodium cyanoborohydride. Reaction was stirred for 8 h. Samples were taken from the reaction mixture, injected into LC-MS to monitor the modification of dialdehyde peptide ARTKme$_2$(CHO)QTARKme$_2$(CHO)S **2d**. The reaction mixture was analyzed by HPLC using

method B and % conversion to single alkynylated peptide product **3b’** was 62% and double alkynylated **3b’’** was 34%.

Kme$_2$(CHO)4Kme$_2$(CHO)9 (ARTKme$_2$(CHO)QTARKme$_2$(CHO)S) 2d. LCMS: *m/z* 1185.62600 (calcd [M+H]+ = 1185.6335), *m/z* 593.31680 (calcd [M+2/2]+ = 593.3167), (HPLC analysis at 220 nm). Retention time in HPLC: 8.768

Single-modified propargylamine histone peptide 3b’. LCMS: *m/z* 1224.67163 (calcd [M+H]+ = 1224.6807), (HPLC analysis at 220 nm). Retention time in HPLC: 8.060

Double-modified propargylamine histone peptide fragment 3b’’. LCMS: *m/z* 1263.71790 (calcd [M+H]+ = 1263.7280), *m/z* 632.36218 (calcd [M+2/2]+ = 632.3640), *m/z* 421.91077 (calcd [M+3/3]+ = 421.9093), (HPLC analysis at 220 nm). Retention time in HPLC: 7.000

HPLC Trace of reductive amination reaction of ARTKme$_2$(CHO)QTARKme$_2$(CHO)S **2d**

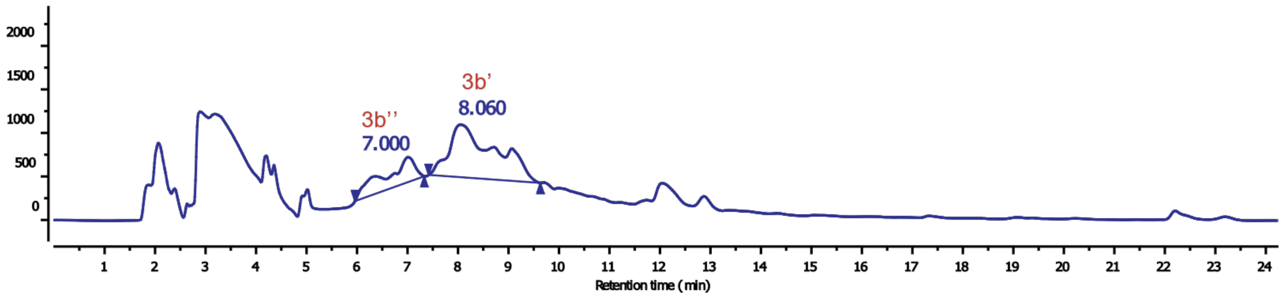

MS-Trace of peak 7.000

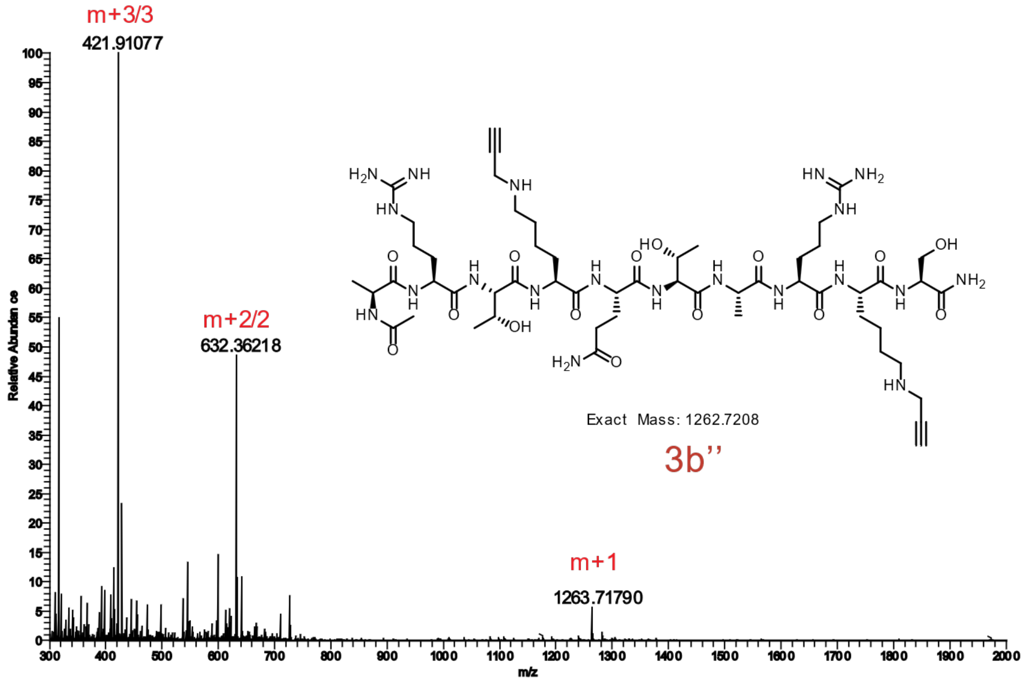

MS-Trace of peak 8.060

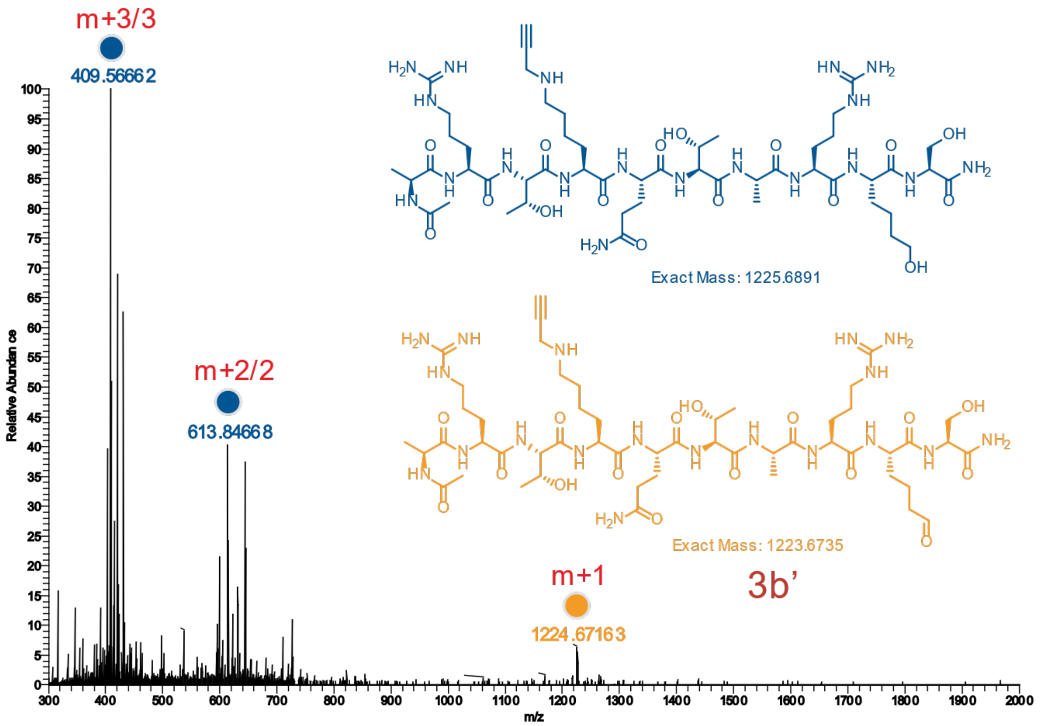

XVI. Supplementary Figure 10. Derivatization of Kme$_2$4K9-aldehyde (ARTKme$_2$(CHO)QTARKS) **2c** peptide aldehyde with hydrazone.

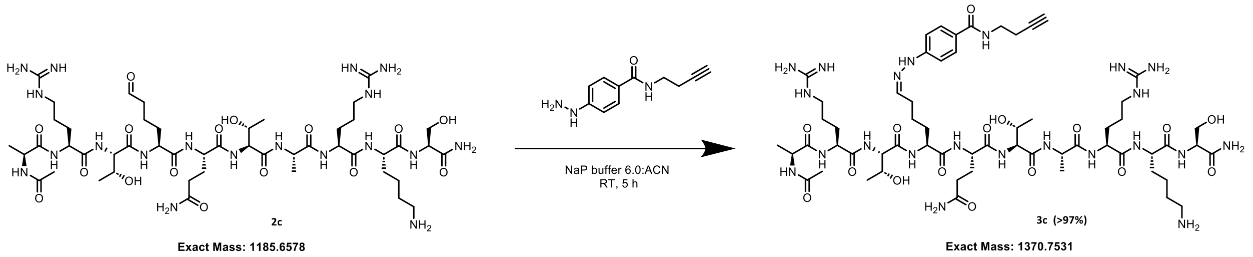

To 1 mg of aldehyde peptide **2c** in 300 μL of NaP buffer and acetonitrile was added 5 equivalences of N-3-butyn-1-yl-4-hydrazinyl-, hydrochloride. The reaction was stirred at room temperature for 5 h. Samples were taken from the reaction mixture, injected into LC-MS to monitor the modification of aldehyde peptide Kme$_2$4K9-aldehyde (ARTKme$_2$(CHO)QTARKS) **2c**. The reaction mixture was analyzed by HPLC using method B and % conversion to alkynylated peptide product **3c** was >97%.

Kme$_2$(CHO)4K9 (ARTKme$_2$(CHO)QTARKS) 2c. LCMS: *m/z* 1186.65866 (calcd [M+H]+ = 1186.6651), *m/z* 593.83341 (calcd [M+2/2]+ = 593.8225), *m/z* 396.21955 (calcd [M+3/3]+ = 396.2217), (HPLC analysis at 220 nm). Retention time in HPLC: 6.421

Alkyne-modified hydrazone peptide 3c. LCMS: *m/z* 686.37823 (calcd [M+2/2]+ = 686.3802), *m/z* 457.91166 (calcd [M+3/3]+ = 457.9201), (HPLC analysis at 220 nm). Retention time in HPLC: 12.319

N-3-butyn-1-yl-4-hydrazinyl-hydrochloride. (HPLC analysis at 220 nm). Retention time in HPLC: 7.121.

HPLC Trace of reaction of hydrzone modification of 2c with alkyne hydrazine

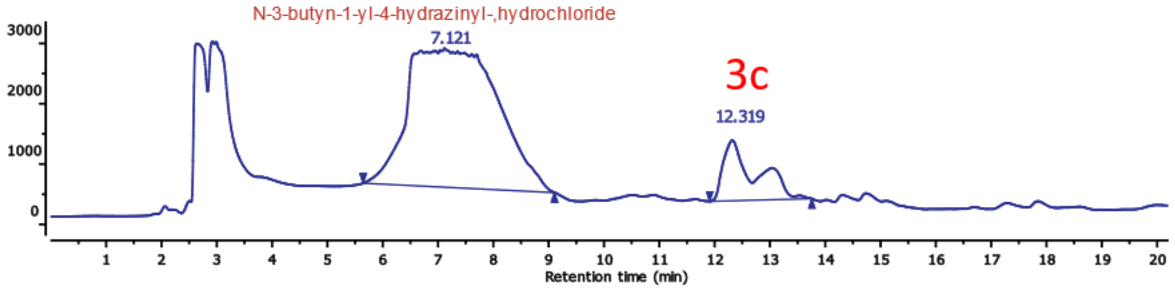

MS-Trace of peak 12.319

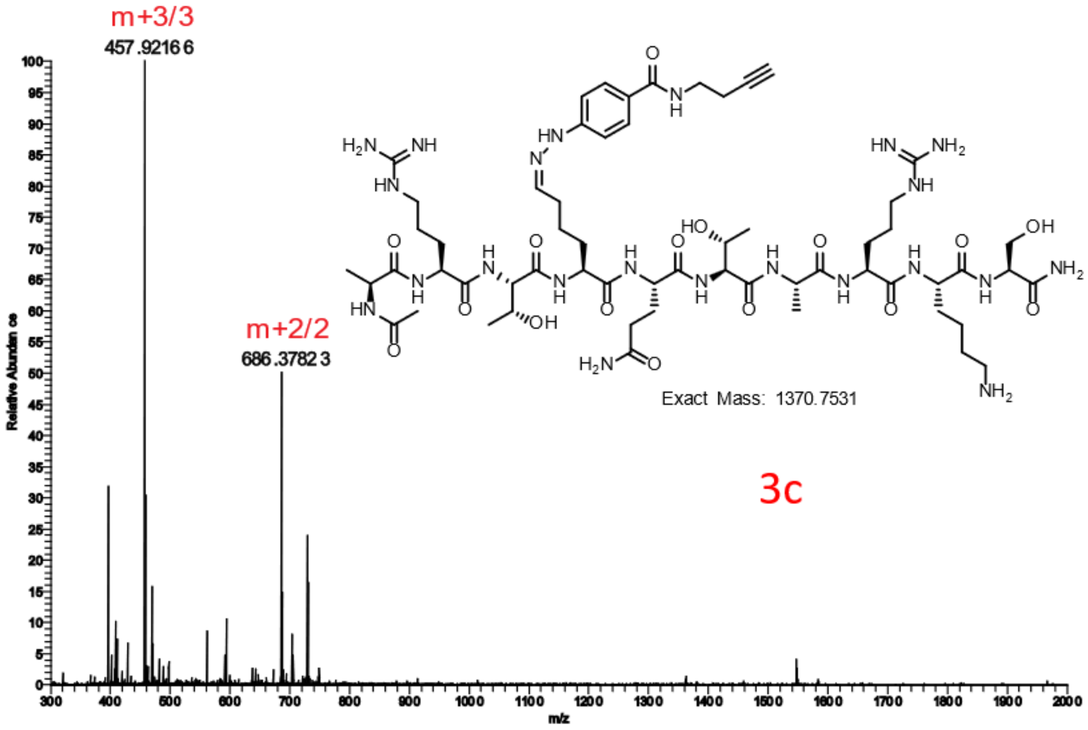

XVII. Supplementary Figure 11: Derivatization of $Kme_2$4K9-aldehyde ($ARTKme_2$(CHO)QTARKS) **2c** with dansyl sulfonylhydrazine.

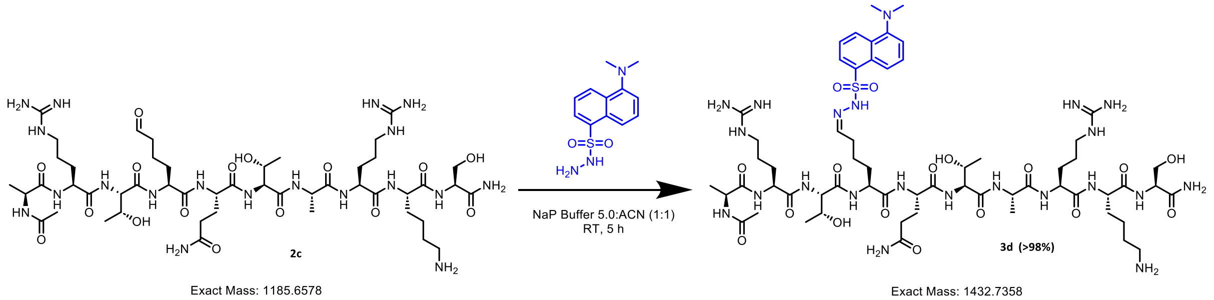

To 1 mg of $Kme_2$4K9-aldehyde ($ARTKme_2$(CHO)QTARKS) aldehyde **2c** in 150 µL of NaP buffer and acetonitrile was added 5 equivalences of dansyl sulfonylhydrazine. Reaction was stirred at room temperature for 5 h. Samples were taken from the reaction mixture, injected into LC-MS to monitor the modification of the histone peptide aldehyde **2c.** The reaction mixture was analyzed by HPLC using method B and % conversion to dansyl hydrazone peptide product **3d** was >98%.

Kme_2(CHO)4K9 ($ARTKme_2$(CHO)QTARKS) 2c. LCMS: *m/z* 1186.65866 (calcd [M+H]+ = 1186.6651), *m/z* 593.83341 (calcd [M+2/2]+ = 593.8325), *m/z* 396.21955 (calcd [M+3/3]+ = 396.2217), (HPLC analysis at 220 nm). Retention time in HPLC: 6.421

Dansyl sulfonylhydrazine-modified histone peptide 3d. LCMS: *m/z* 717.36963 (calcd [M+2/2]+ =717.3715), *m/z* 478.58221 (calcd [M+3/3]+ =478.5810), (HPLC analysis at 220 nm). Retention time in HPLC: 12.206

HPLC Trace of reaction

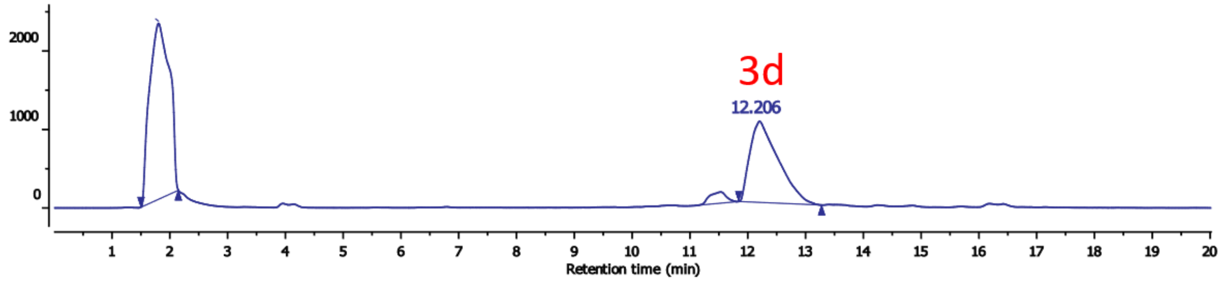

MS-Trace of peak 12.206

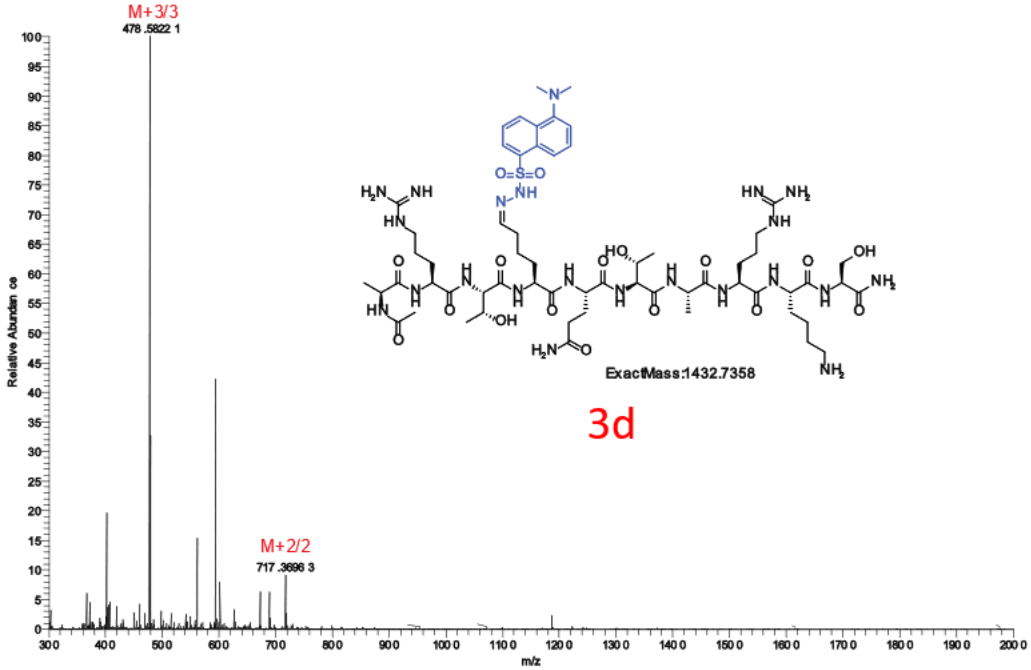

XVIII. Supplementary Figure 12: Derivatization of (ARTKme$_2$(CHO)STGGKA) **2e** with biotin-hydrazide.

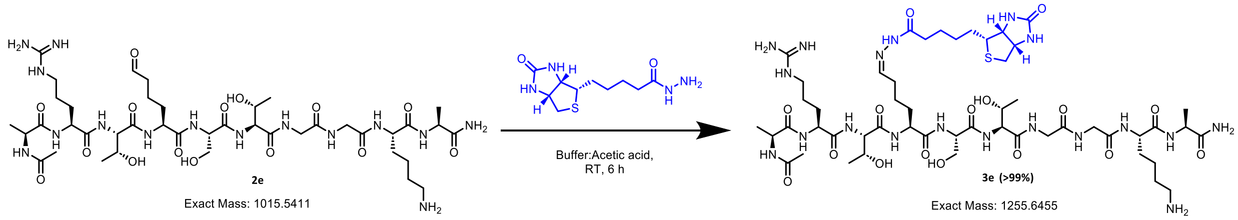

To 1 mg of aldehyde peptide (ARTKme$_2$(CHO)STGGKA) **2e** in 100 µL of NaP buffer and 200 µL acetic acid was added 10 equivalences of biotinhydrazine. The reaction was stirred at room temperature for 6 h. Samples were taken from the reaction mixture, injected into LC-MS to monitor the modification of the histone peptide aldehyde ARTKme$_2$(CHO)STGGKA) **2e.** The reaction mixture was analyzed by HPLC using method B and % conversion to biotin-hydrazone peptide product **3e** was >99%.

Histone aldehyde peptide product 2e. LCMS: *m/z* 1016.54144 (calcd [M+H]+ = 1016.5483), *m/z* 508.77432 (calcd [M+2/2]+ = 508.7741), (HPLC analysis at 220 nm). Retention time in HPLC: 7.239

Biotin-hydrazide modified histone peptide product 3e. LCMS: *m/z* 1256.87647 (calcd [M+H]+ = 1256.6528), *m/z* 1278.98010 (calcd [M+Na]+ = 1278.6348), *m/z* 628.46888 (calcd [M+2/2]+ = 628.8227), (HPLC analysis at 220 nm). Retention time in HPLC: 9.151

HPLC Trace of reaction

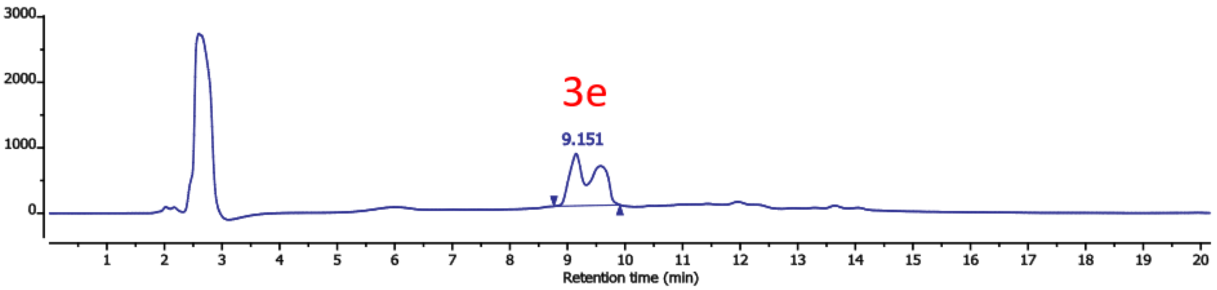

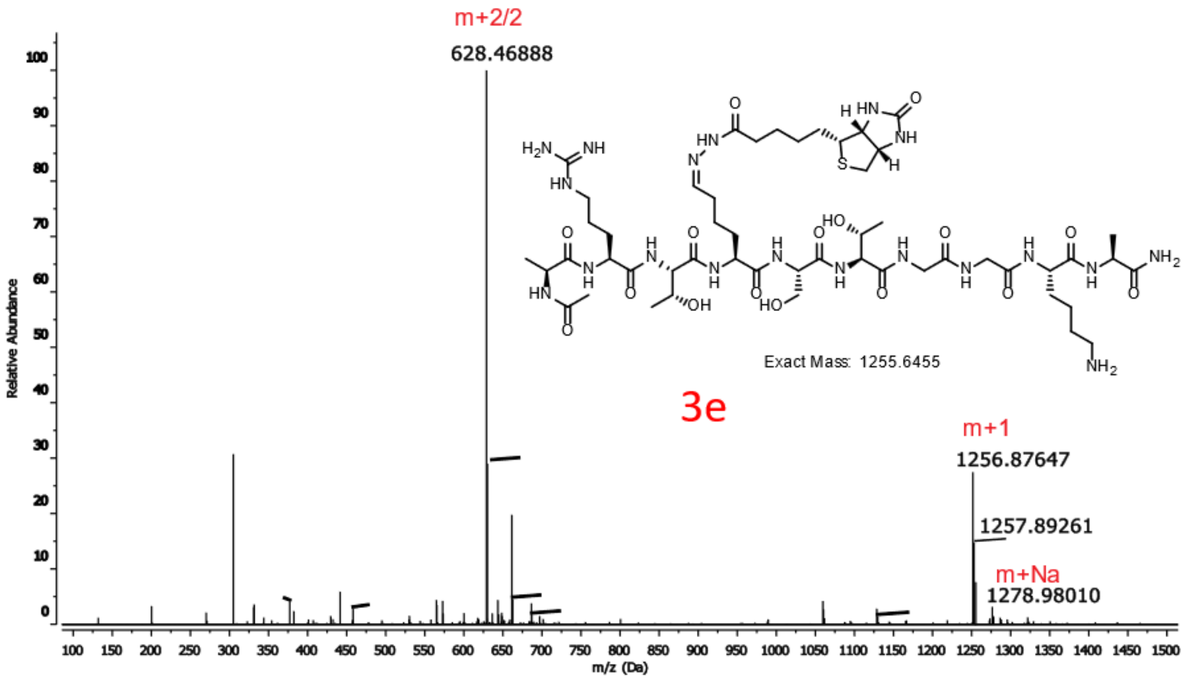

XIX. Supplementary Figure 13: Modification of peptide aldehydes with cysteine-derivatives to thiazolidine products.

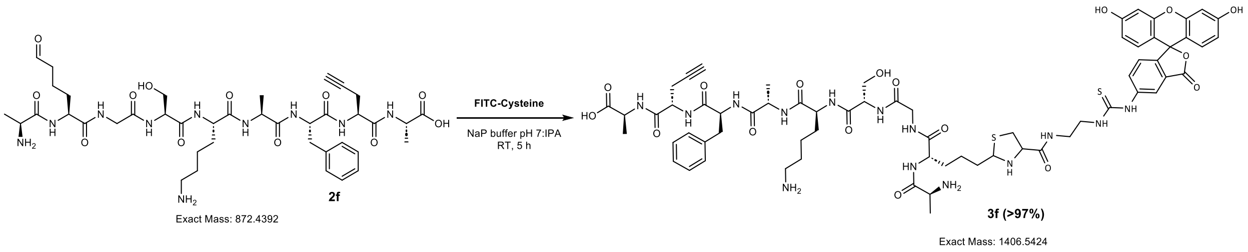

Labeling of peptide aldehyde 2f by FITC-cysteine: To 1 mg of peptide aldehyde AKme$_2$(CHO)GSKAF(PrG)A (where PrG = propargyl glycine) **2f** in 300 μL of NaP buffer and isopropyl alcohol (IPA) was added 3 equivalences of FITC-Cysteine. Reaction was stirred at room temperature for 5 h. Samples were taken from the reaction mixture, injected into LC-MS to monitor the formation of thiazolidine **3f**. The reaction mixture was analyzed by HPLC using method A and % conversion to thiazolidine product **3f** was >97%.

Peptide aldehyde product 2f. LCMS: *m/z* 873.43885 (calcd [M+H]+ = 873.4284), *m/z* 895.59729 (calcd [M+Na]+ = 895.4284), (HPLC analysis at 220 nm). Retention time in HPLC: 9.170

FITC-Thiazolidine peptide product 3f. LCMS: *m/z* 1407.54264 (calcd [M+H]+ = 1407.5497), *m/z* 704.31022 (calcd [M+2/2]+ = 704.7748), (HPLC analysis at 220 nm). Retention time in HPLC: 12.451

HPLC Trace of thiazolidine product 3f

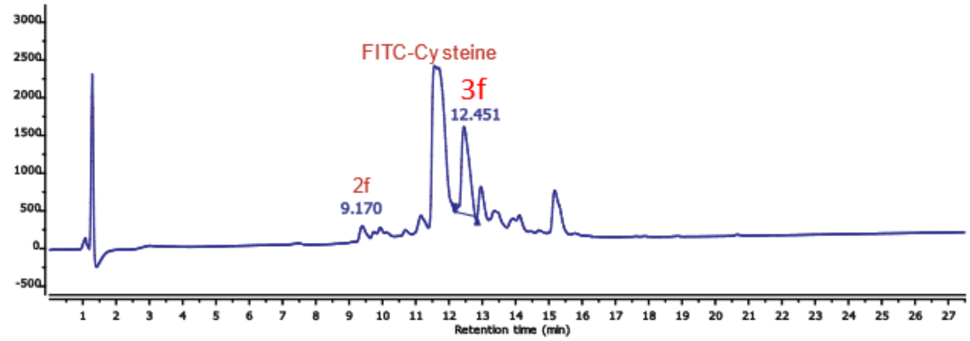

MS-Trace of peak 9.170

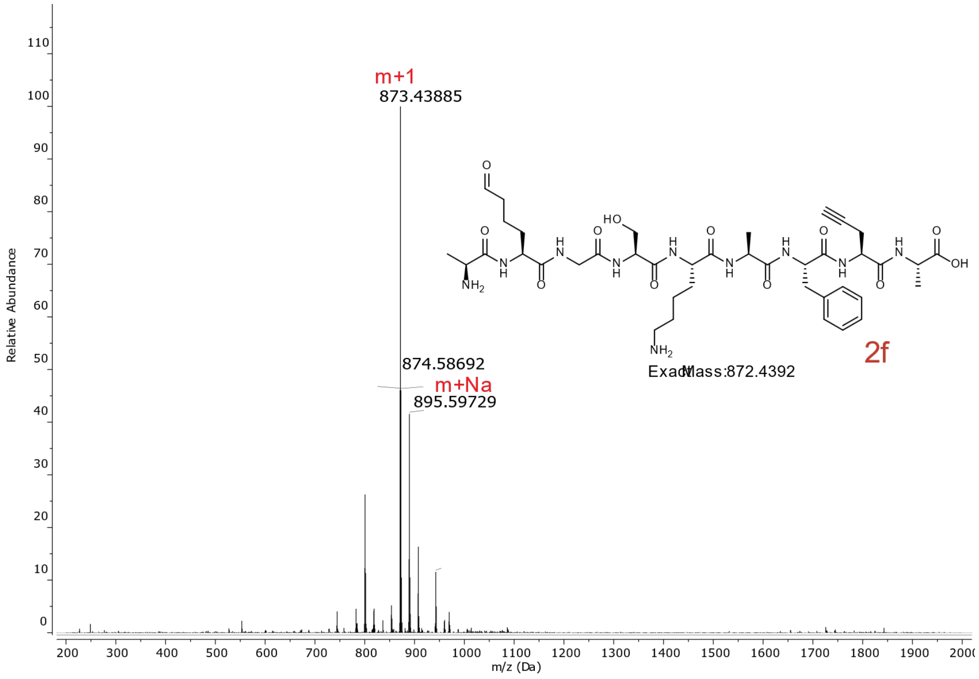

MS-Trace of peak 12.451

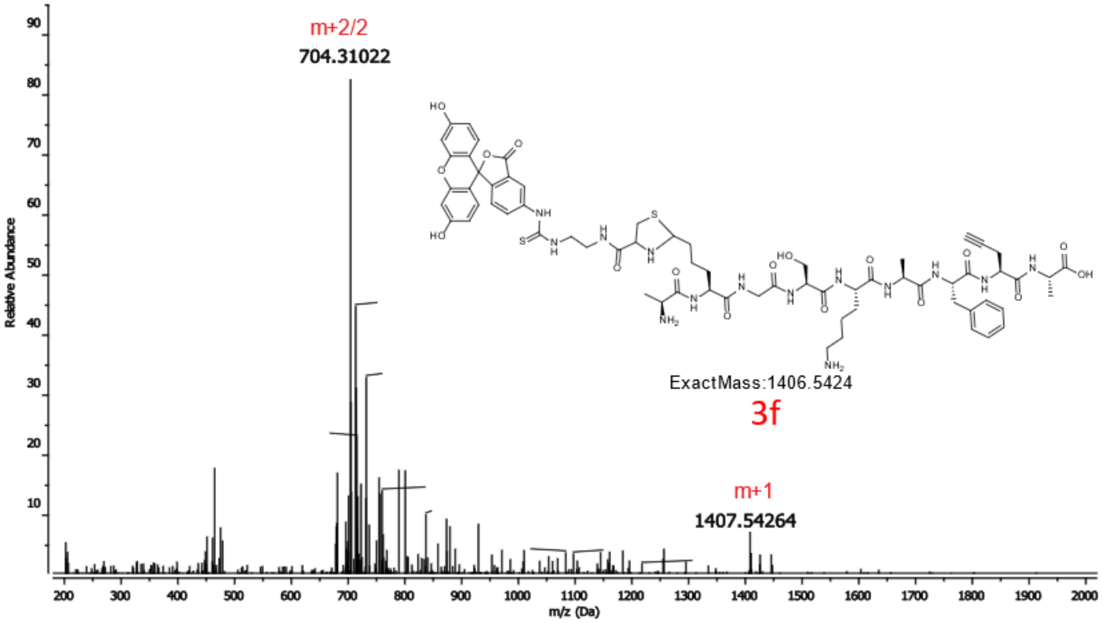

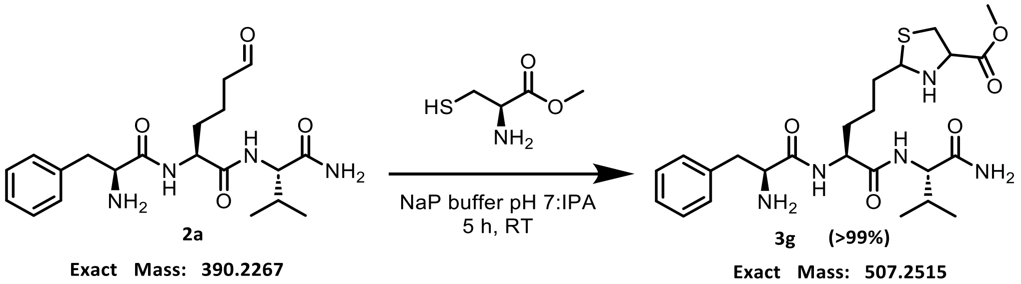

To 1 mg of peptide aldehyde Fkme$_2$(CHO)V **2a** in 300 µL of NaP buffer and isopropyl alcohol (IPA) was added 5 equivalences of cysteine methylester. Reaction was stirred at room temperature for 5 h. Samples were taken from the reaction mixture, injected into LC-MS to monitor the formation of thiazolidine. The reaction mixture was analyzed by HPLC using method A and % conversion to thiazolidine product was >99%.

FKme$_2$(CHO)V 2a. LCMS: *m/z* 391.23049 (calcd [M+H]+ = 391.2340), (HPLC analysis at 220 nm). Retention time in HPLC: 6.681

Thiazolidine peptide product of aldehyde 3g. LCMS: *m/z* 508.25449 (calcd [M+H]+ = 508.2588), *m/z* 254.63107 (calcd [M+2/2]+ = 254.6294), *m/z* 530.23617 (calcd [M+Na]+ = 530.2408), (HPLC analysis at 220 nm). Retention time in HPLC: 7.661

HPLC Trace of $FKme_2(CHO)V$ 2a

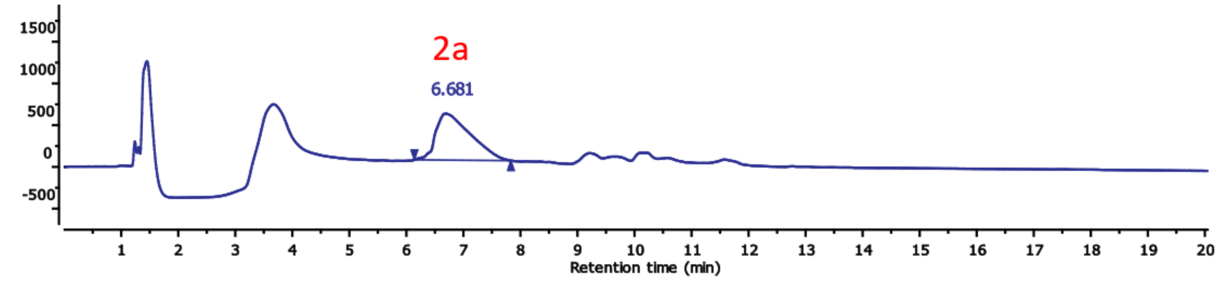

HPLC Trace of thiazolidine product

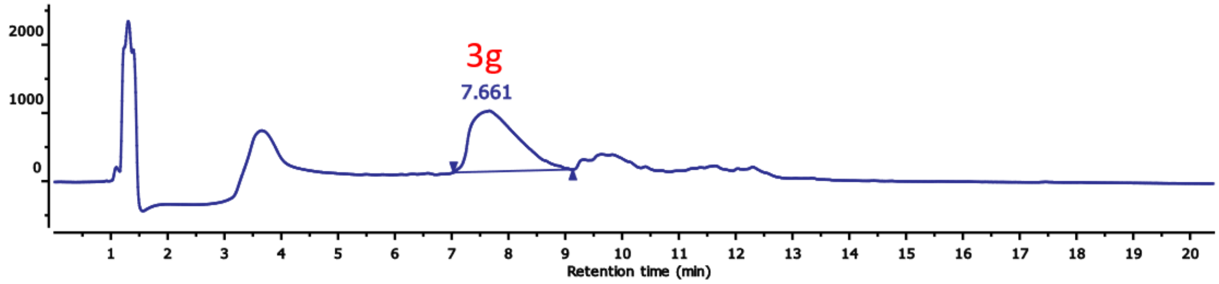

MS-Trace of peak 7.661

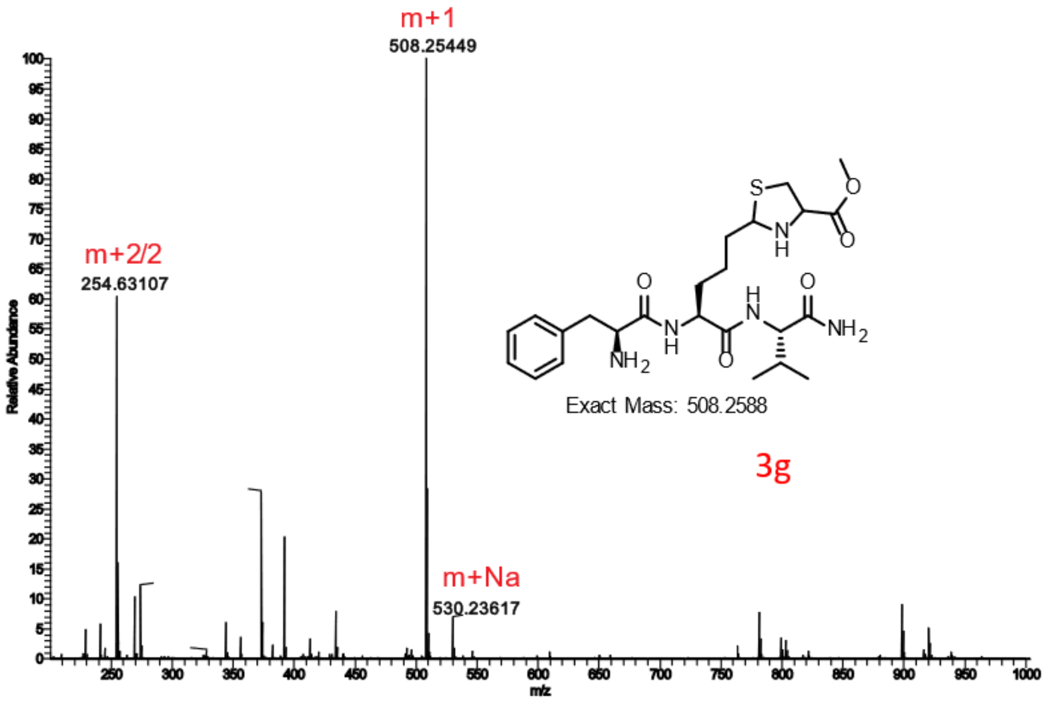

XX. Supplementary Figure 14: Oxidative tertiary amine transformation of dimethyllysine containing histone peptides fragments (**1c-1e**) spiked into a complex mixture of epithelial carcinoma A549 whole cell lysate.

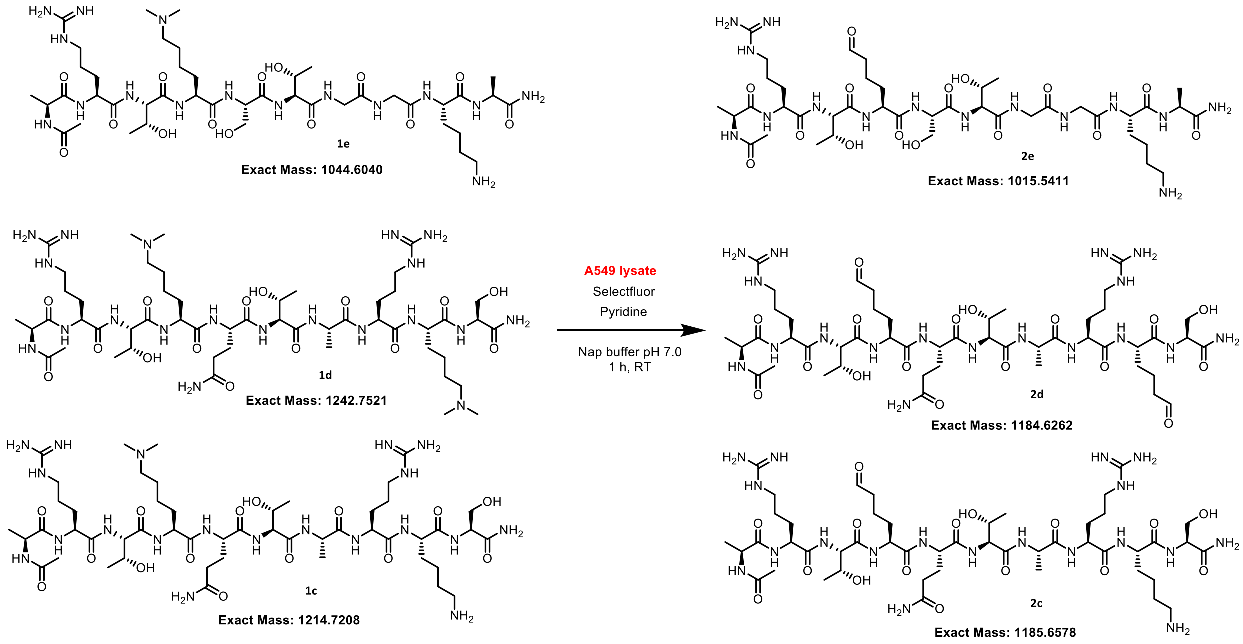

To 100 µg of A549 whole cell lysate in 200 µL of NaP buffer pH 7.0 was added 0.1 mg of histone peptide ($ARTKme_2QTARKS$) **1c**, 0.1 mg of histone peptide Kme_24Kme_29 ($ARTKme_2QTARKme_2S$) **1d**, and 0.1 mg of histone peptide Kme_29K14 ($ARTKme_2STGGKA$**)** **1e**. To this mixture of lysate and histone peptides was added pyridine (30 eq with respect to peptides) and selectfluor (10 eq. with respect to peptides). The reaction mixture was stirred for 1 h. Samples were taken from the reaction mixture, injected into LC-MS to monitor the modification of dimethyllysine containing histone peptides into aldehyde products. The reaction mixture was analyzed by HPLC using method B.

MS-Trace of the Cell lysate after the reaction

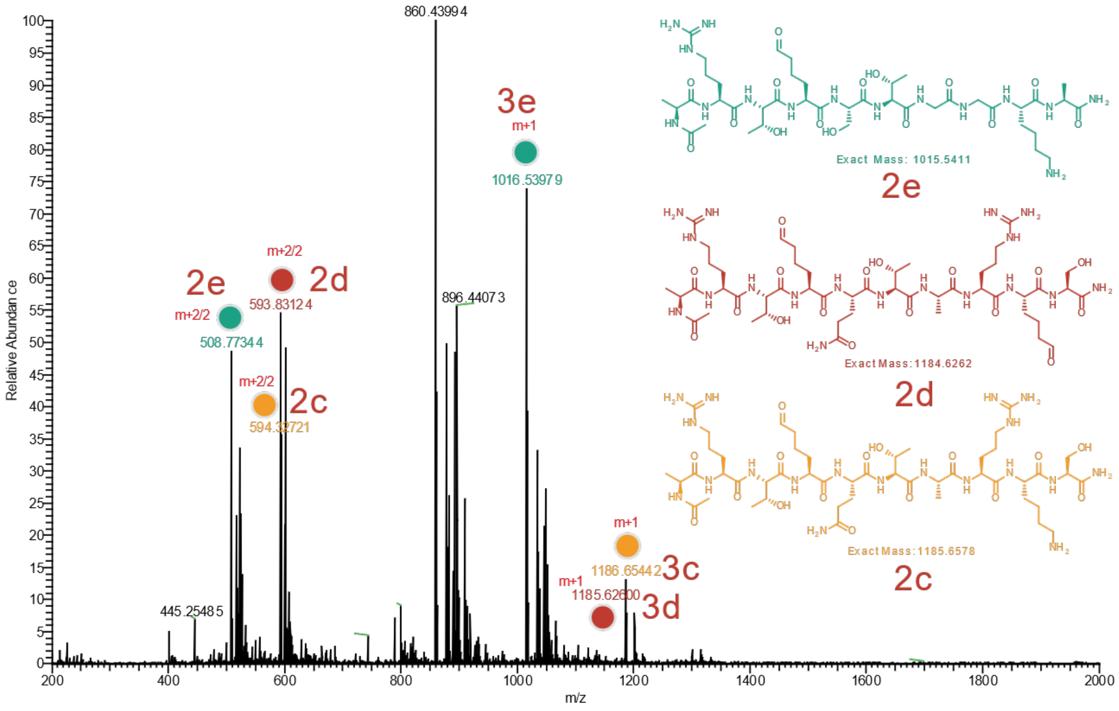

XXI. Supplementary Fig. 15: Oxidative tertiary amine transformation of dimethyllysine containing histone peptides fragments (**1c, 1d and 1g**) spiked into a complex mixture of H1792-lung cancer cell lysate.

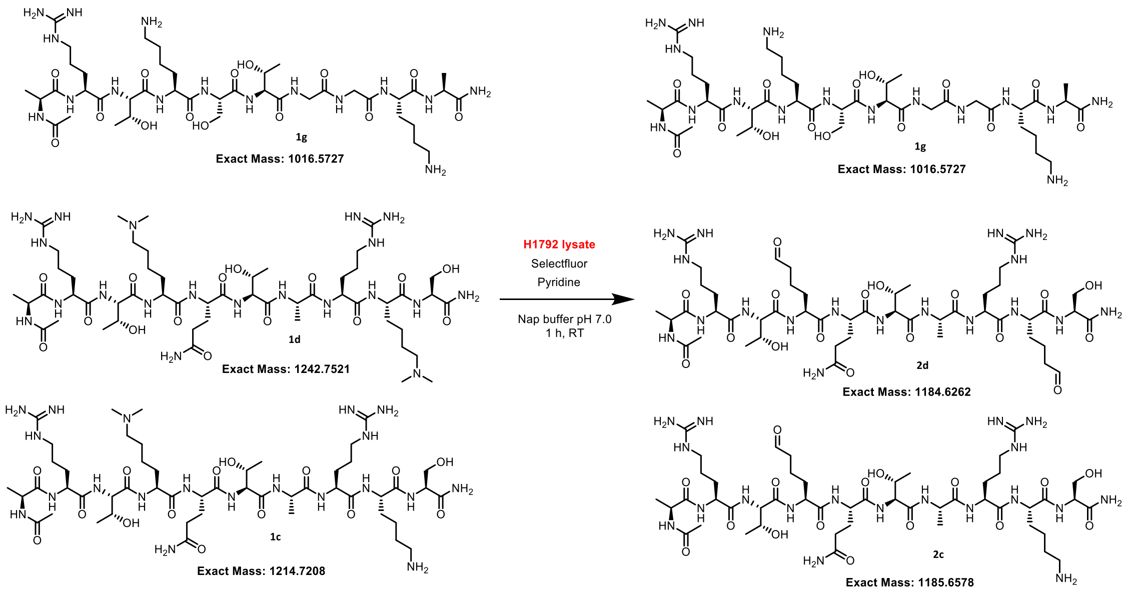

To 100 µg of H1792 whole cell lysate in 200 µL of NaP buffer pH 7.0 was added 0.1 mg of histone peptide (ARTKme$_2$QTARKS) **1c**, 0.1 mg of histone peptide Kme$_2$4Kme$_2$9

(ARTKme$_2$QTARKme$_2$S) **1d**, and 0.1 mg of histone peptide Kme$_2$9K14 (ARTKme$_2$STGGKA) **1g**. To this mixture of lysate and histone peptides was added pyridine (30 eq with respect to peptides) and selectfluor (10 eq. with respect to peptides). The reaction mixture was stirred for 1 h. Samples were taken from the reaction mixture, injected into LC-MS to monitor the modification of dimethyllysine containing histone peptides into aldehyde products. The reaction mixture was analyzed by HPLC using method B.

MS-Trace of the Cell lysate after the reaction

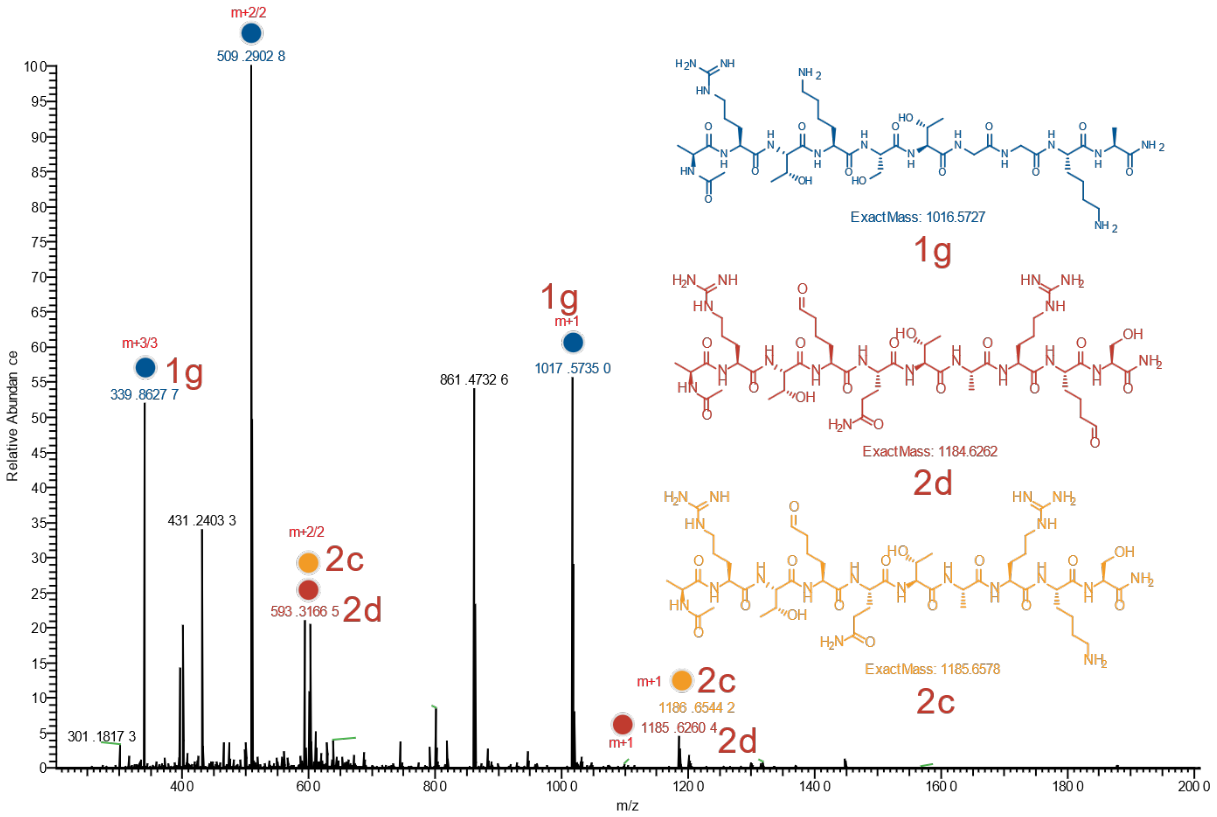

XXII. Supplementary Figure 16. Single-molecule sequencing for identification of Kme$_2$ sites by TACO

Aldehyde generation: To 1.0 mg of fluorosequencing peptide fragment AKme$_2$GSKAF(PrG)A (where PrG = propargyl glycine) dissolved in 300 µL of 10 mM sodium phosphate buffer (NaP, pH 7.0), was added pyridine (5 eq) and selectfluor (2 eq). The reaction mixture was stirred for 1 h. Samples were taken from the reaction mixture, injected into LC-MS to monitor the generation of peptide aldehyde product AKme$_2$(CHO)GSKAF(PrG)A **2f** (>98%).

AKme$_2$(CHO)GSKAF(PrG)A **2f.** LCMS: *m/z* 873.43885 (calcd [M+H]+ = 508.4465), *m/z* 895.59729 (calcd [M+Na]+ = 895.4284), (HPLC analysis at 220 nm). Retention time in HPLC: 9.17

HPLC trace of reaction

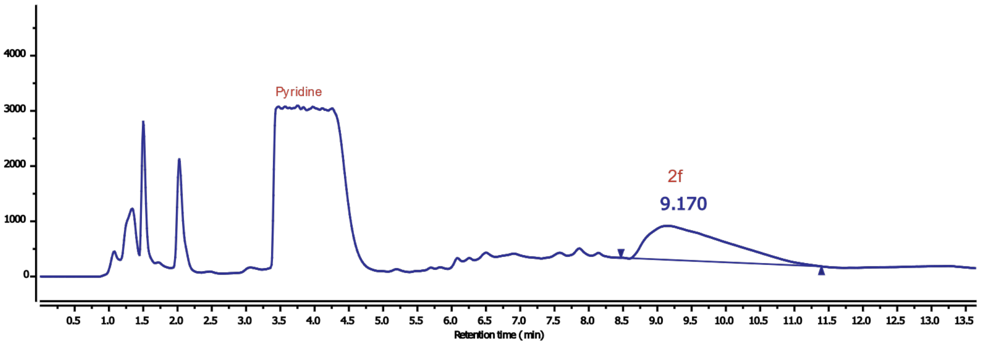

MS-Trace of peak 9.170

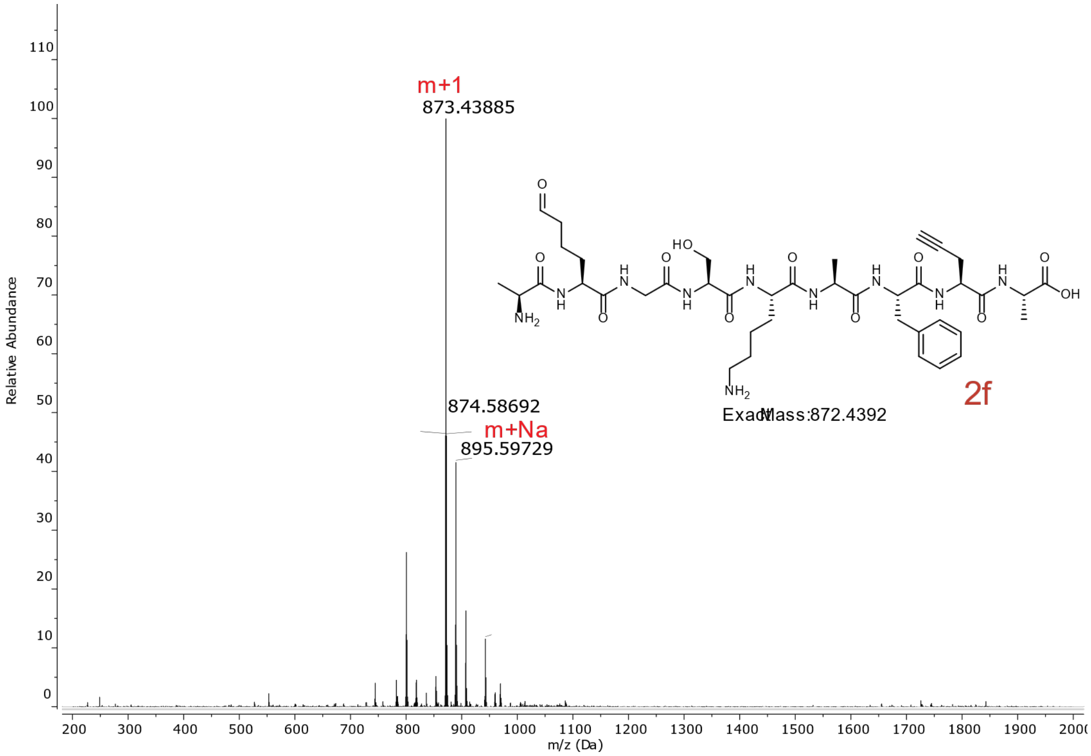

Fluorophore labeling of Kme$_2$ peptide: Atto647N-LA (Lipoic acid) was custom synthesized by Atto-tec (Germany), by reacting 1 mg of Atto647N-Amine (Cat# AD 647N-91) with 2 eq. lipoic acid (Cas # 1077-28-7; Sigma (Cat # T5625-1G)) and 2 eq. of triethylamine in dimethylformamide for 2 h at RT. Standard HPLC purification was performed to isolate the Atto647N-LA compound and lyophilized. Atto647N-LA was coupled to modified Kme$_2$ peptide (AKme$_2$(CHO)GSKAF(PrG)A) **2f** by reacting with 1.5 eq of Atto647N-LA, 2 eq. TCEP (tris(2-carboxyethyl)phosphine) in solution of 5% TFA in methanol/water (1:1 vv) solution for 16 h. The labeled peptide was purified using standard HPLC method and lyophilized.

Atto647N-peptide product. LCMS: *m/z* 876.0 (calcd [M+2/2]+ = 867.4679), *m/z* 578.5 (calcd [M+3/3]+ =578.6452), (HPLC analysis at 220 nm). Retention time in HPLC: 6.0

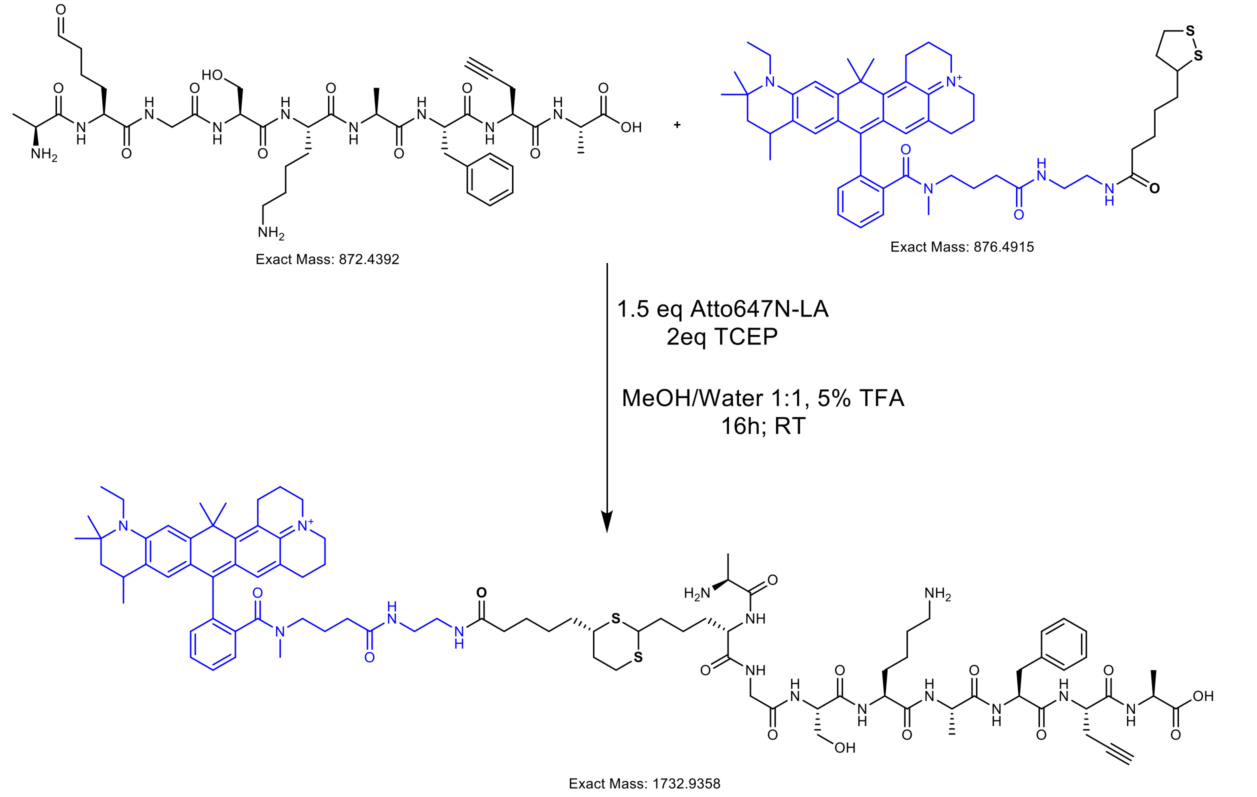

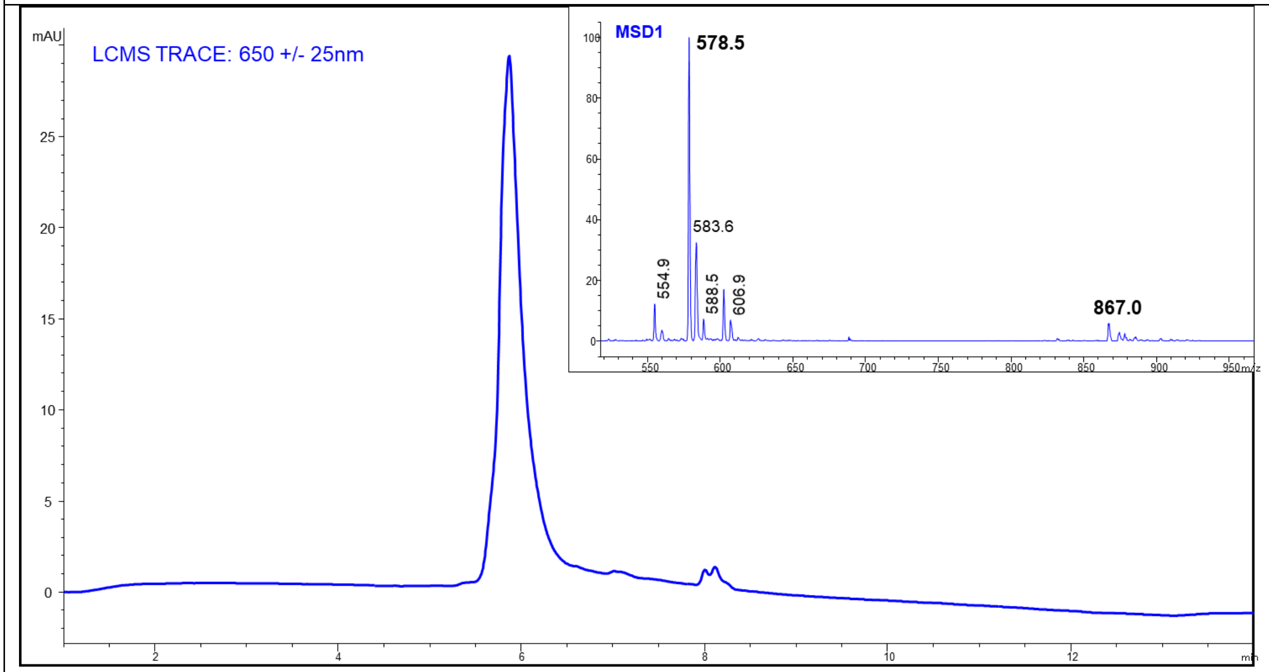

Surface immobilization of peptide. For single molecule peptide sequencing, a 40 mm German Desag 263 borosilicate glass coverslip (Bioptechs) surface was first cleaned by UV/ozone (Jelight Company) and then functionalized by chemical vapor deposition of 3-azidopropyltriethoxysilane (Gelest, Cat # SIA0770.0) under vacuum at 70 ^{o}C for 1 h. Atto647N labeled Kme_2 peptides were covalently coupled to the coverslip surface *via* copper catalyzed click chemistry between the propargyl amino acid on peptide and the azido silane. A fresh solution of of 2 mM copper sulfate, 1 mM Tris(3-hydroxypropyltriazolylmethyl)amine (Sigma, Cat # 762342), 100 mM HEPES (pH 8.5), 5mM sodium ascorbate with Atto647N labeled Kme_2 peptides was incubated for 10 minutes at room temperature on the azido silane functionalized coverslip, washed with water to remove unbound peptides, and dried under a nitrogen gas stream.

Fluorosequencing.[2] Single molecule sequencing was performed as described with minor modifications in the solvents use, incubation times and use of a different camera (Orca Fusion BT, Hamamatsu). The wash solvent ethyl acetate was substituted with acetonitrile and the phenyl isothiocyanate solution is now 20% vv in pyridine. Incubation time for cleavage step (use of trifluoroacetic acid) is reduced to 5mins. Fluorosequencing datasets were analyzed using the *SigProc* software tool, available as part of the Plaster package at https://github.com/erisyon/plaster_v1. Further details of the experimental methods and analysis are available on request.

XXIII. Supplementary Figure 17. Modification of Kme_2 proteins using TACO

General procedure for dimethylation of proteins: To 2 mg of protein in 1 mL of NaP buffer (pH 7), was added 500 µL of 10 % formaldehyde solution in water. The reaction was vortexed for 2 min, followed by the addition of 500 µL of 600 mM $NaBH_3CN$ solution in water, and vortexed for additional 2 min. The reaction was incubated at room temperature for 6 min, filtered through a molecular weight cut off (3 kDa) to remove small molecules and the resulting purified protein was analyzed by MS.

Mass spectrum of unmodified aprotinin protein

Molecular weight: 6511 (4 lysines, 1 N-terminus)

Sequence:

RPDFCLEPPYTGPCKARIIRYFYNAKAGLCQTFVYGGCRAKRNNFKSAEDCMRTCGGA

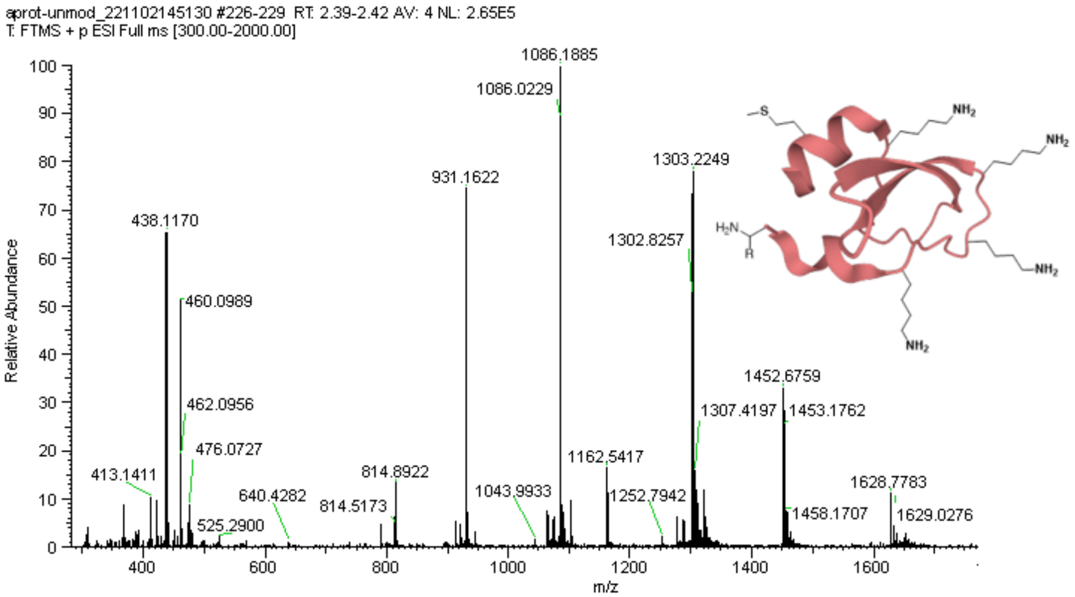

Mass spectrum of dimethylated lysine aprotinin protein

Molecular weight: 6511 (4 lysines, 1 N-terminus)

Sequence:

(me2)RPDFCLEPPYTGPCKme$_2$ARIIRYFYNAKme$_2$AGLCQTFVYGGCRAKme$_2$RNNFKme$_2$SAE DCMRTCGGA

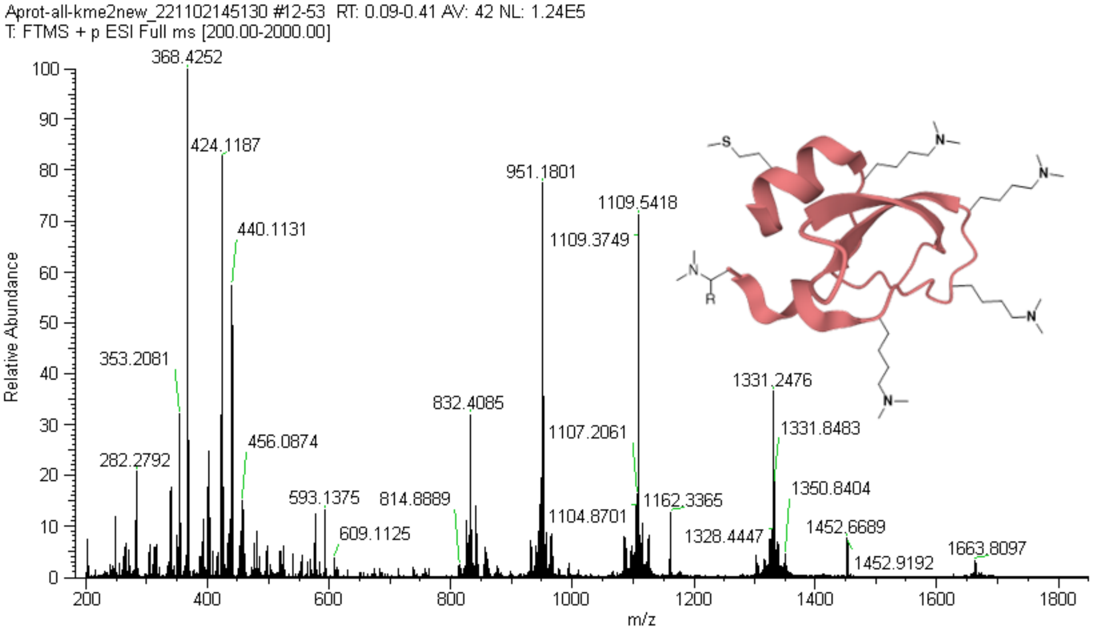

TACO modification of Kme$_2$ containing proteins: To 2 mg Kme$_2$ proteins in 1 mL of NaP buffer pH 7, was added 10 mg of selectfluor and 50 µL of pyridine. The reaction was stirred at room temperature for 30 min and filtered through a molecular weight cutoff to remove chemical

reagents at the end of reaction. Protein samples were digested using the thermofischer scientific lysine/Lys C protein digestion kit. Digested samples were analyzed using LC-MS/MS.

APROTININ

Fragment 1 mass spectrum data for aprotinin

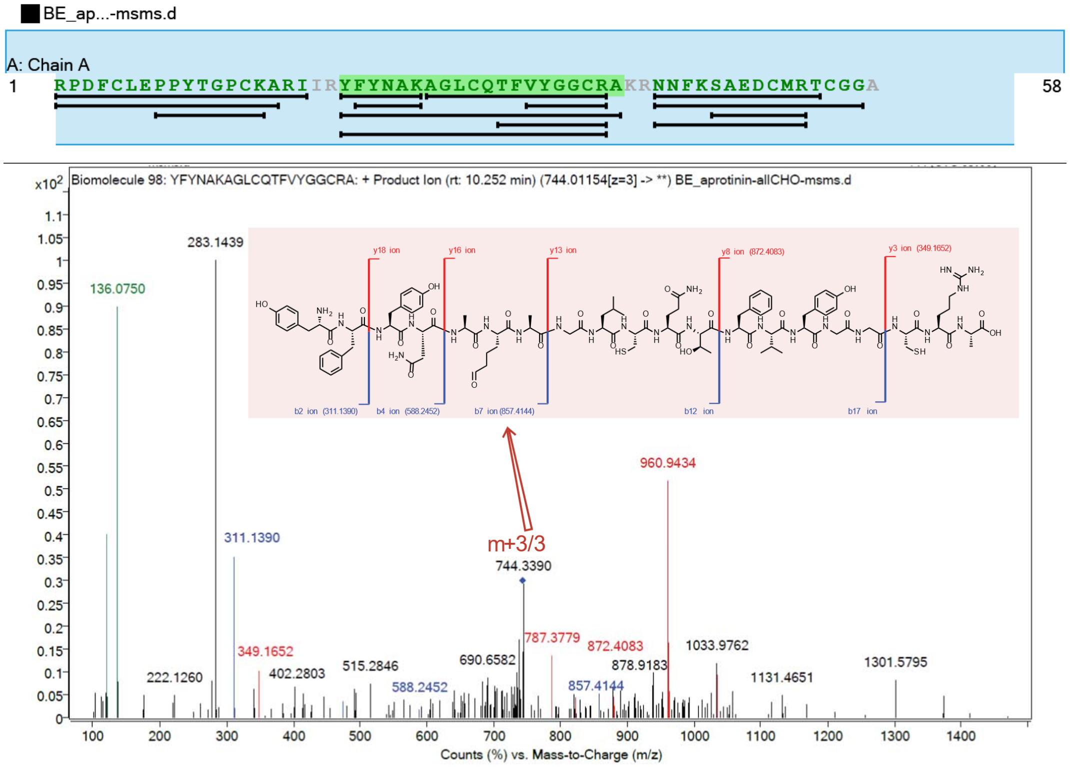

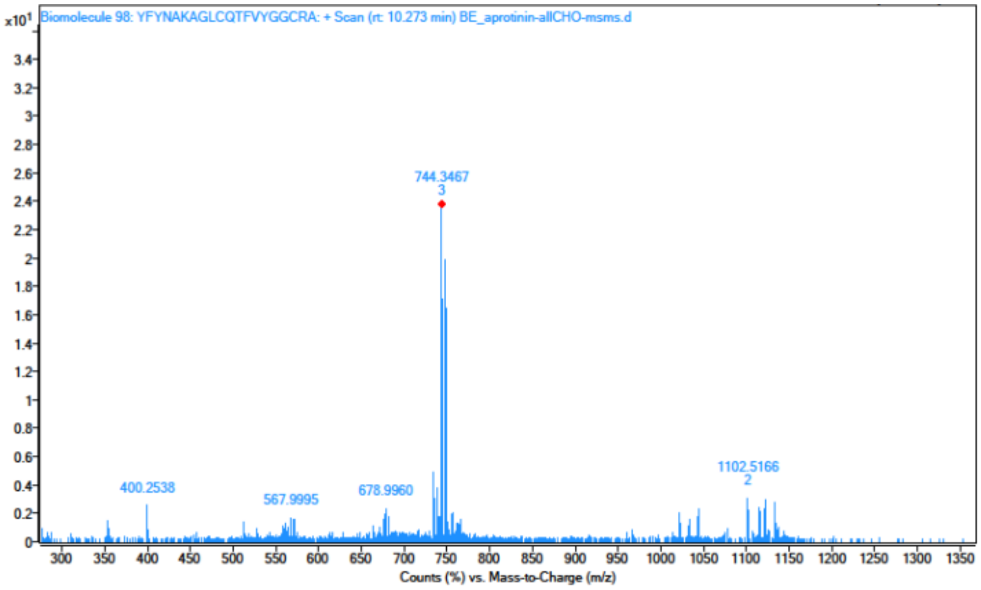

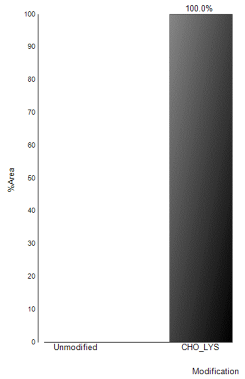

Fragment 2 mass spectrum data for aprotinin

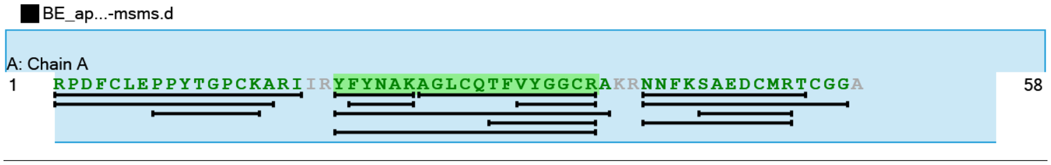

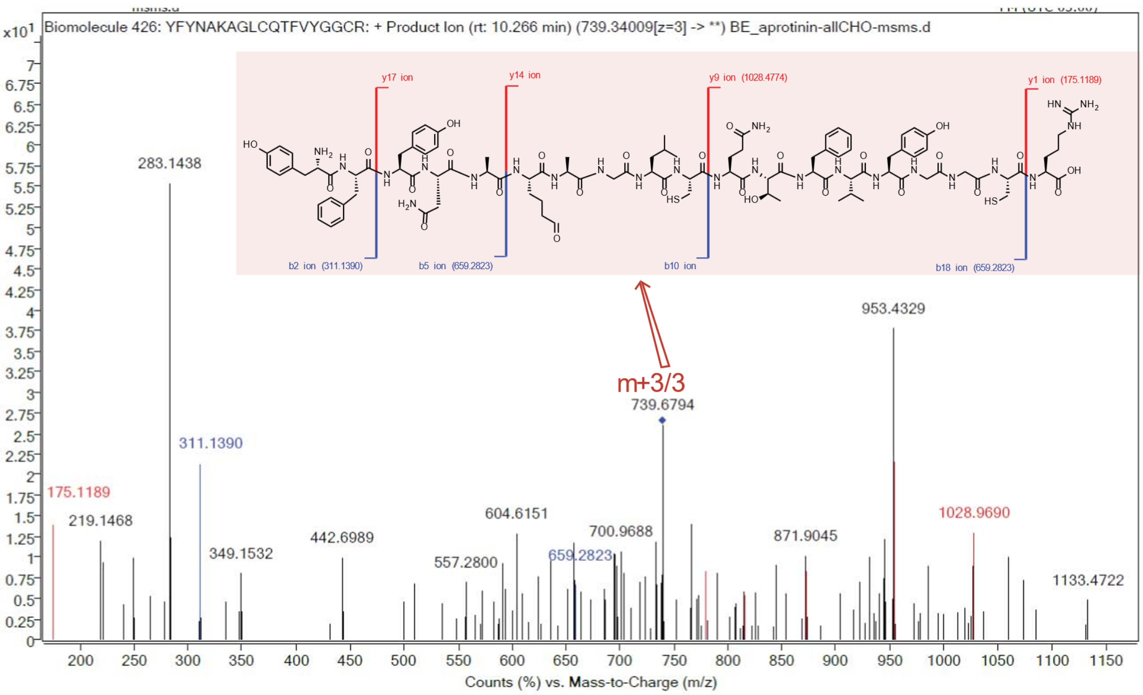

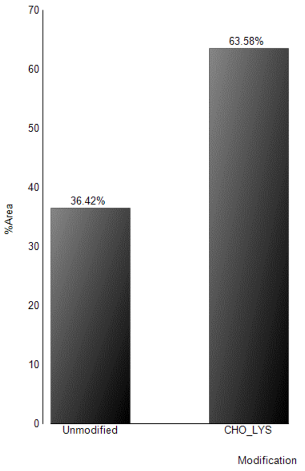

MYOGLOBIN

Fragment mass spectrum data for myoglobin

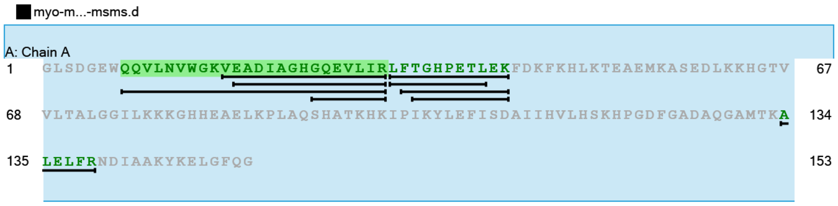

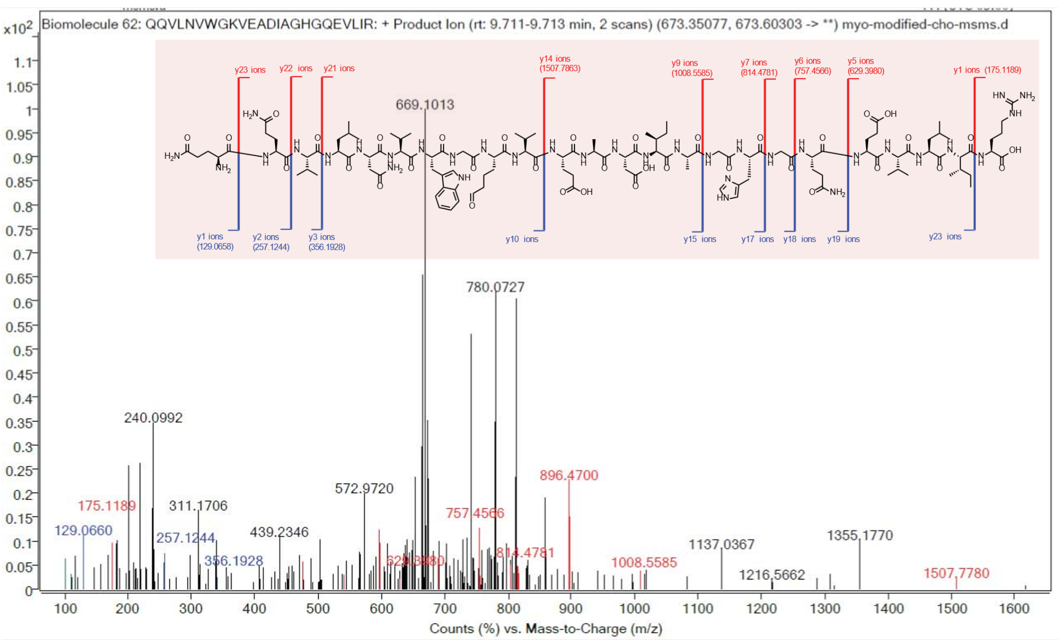

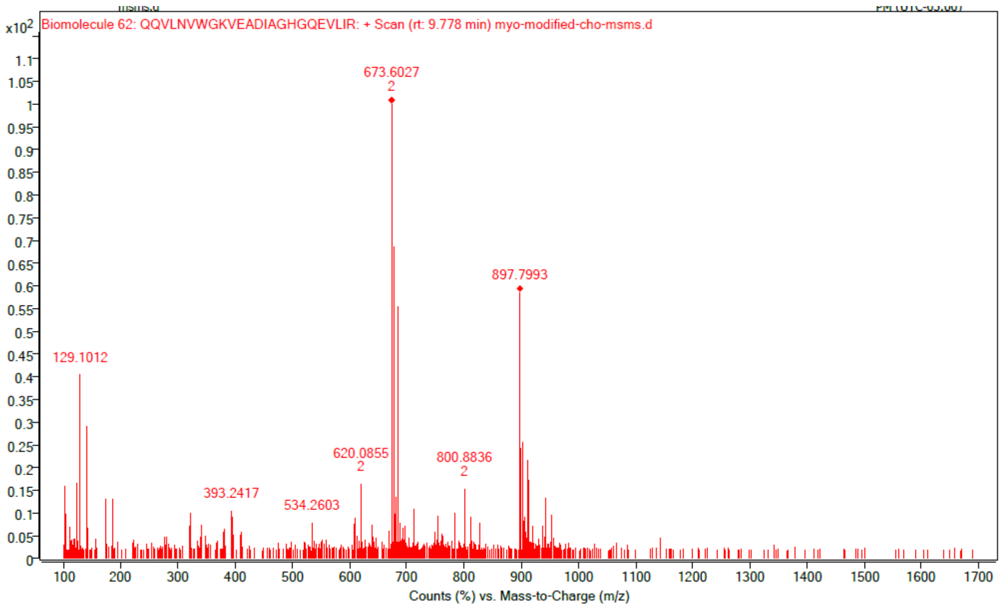

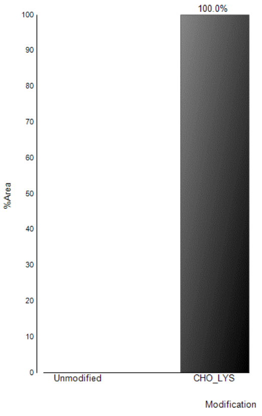

TRANSFERRIN

Fragment 1 mass spectrum data for transferrin

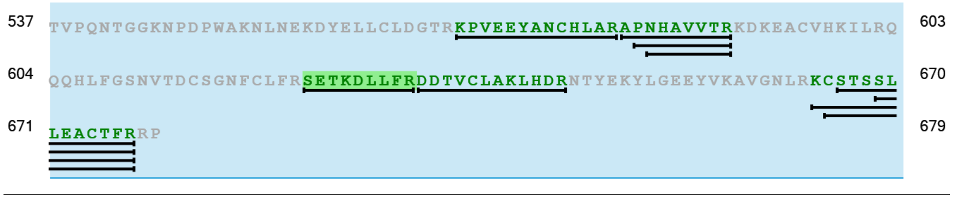

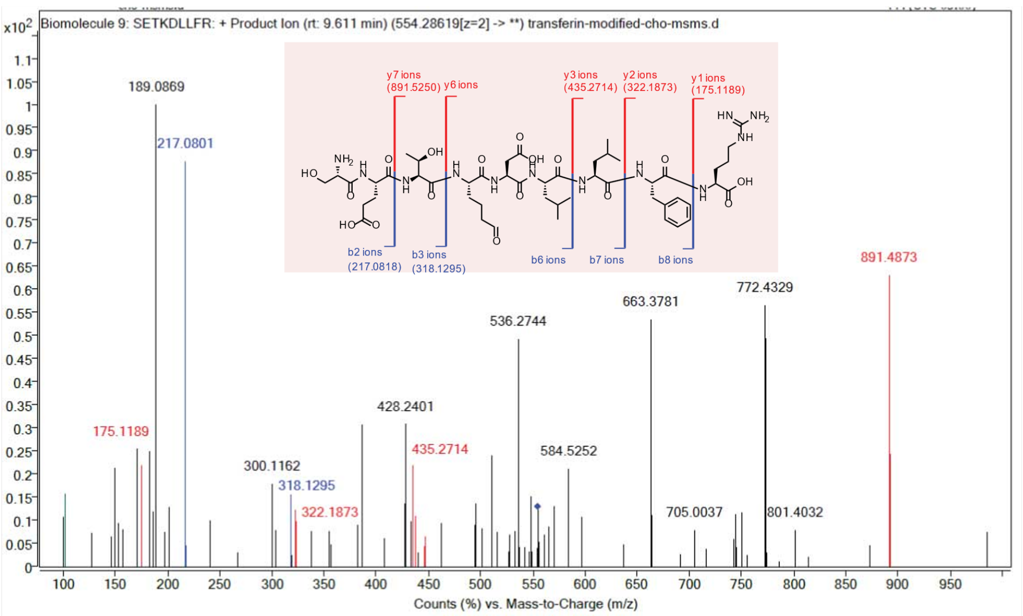

Biomolecule 9: SETKDLLFR: + Product Ion (rt: 9.611 min) (554.28619[z=2] -> **) transferin-modified-cho-msms.d
y7 ions (891.5250)
y6 ions
y3 ions (435.2714)
y2 ions (322.1873)
y1 ions (175.1189)
b2 ions (217.0818)
b3 ions (318.1295)
b6 ions
b7 ions
b8 ions
189.0869
217.0801
175.1189
300.1162
318.1295
322.1873
428.2401
435.2714
536.2744
584.5252
663.3781
705.0037
772.4329
801.4032
891.4873
Counts (%) vs. Mass-to-Charge (m/z)

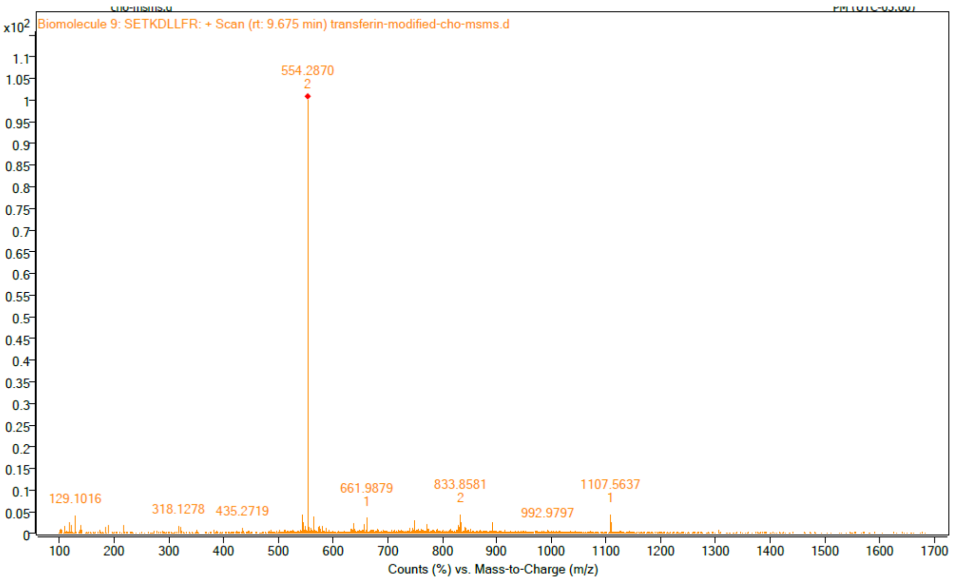

Biomolecule 9: SETKDLLFR: + Scan (rt: 9.675 min) transferin-modified-cho-msms.d
554.2870
129.1016
318.1278
435.2719
661.9879
833.8581
992.9797
1107.5637
Counts (%) vs. Mass-to-Charge (m/z)

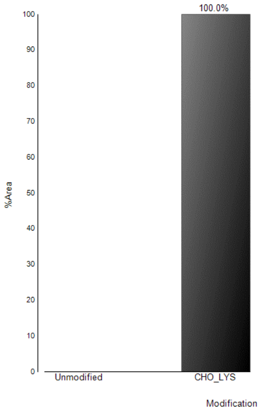

100.0%
%Area
Unmodified
CHO_LYS
Modification

Fragment 2 mass spectrum data for transferrin

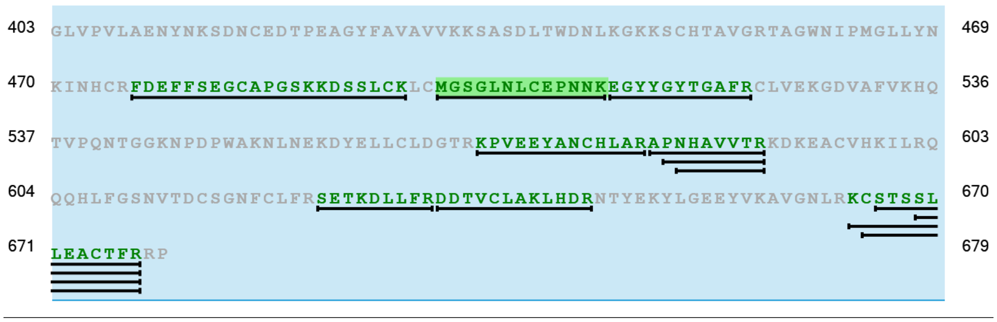

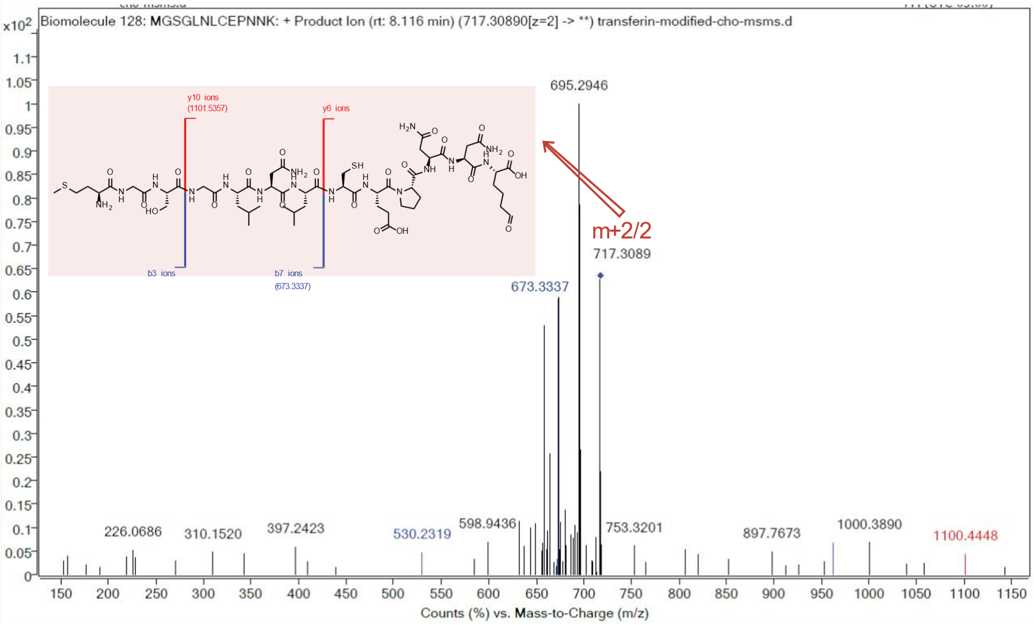

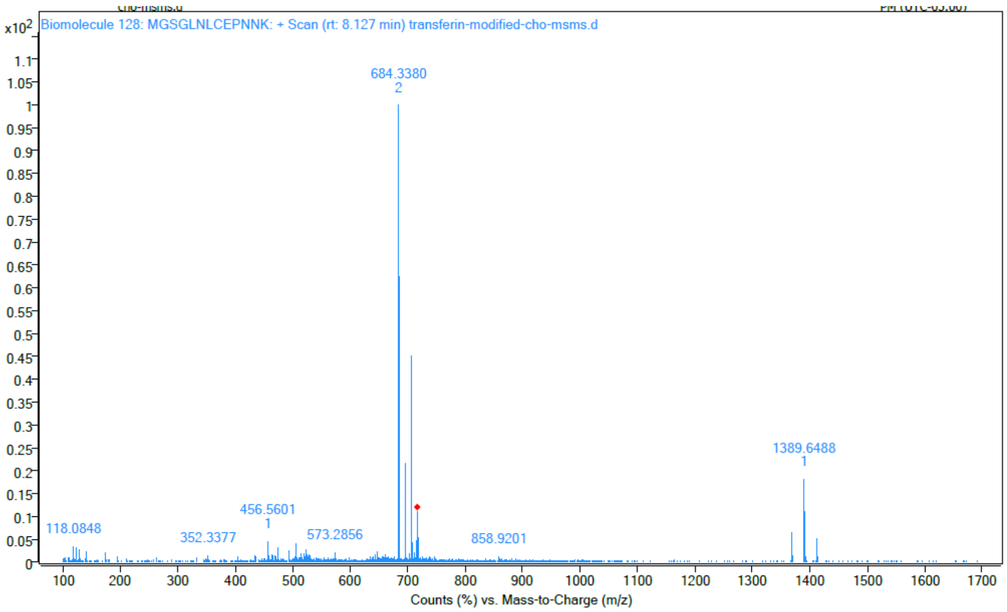

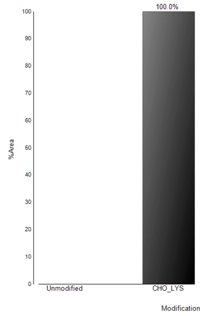

XXIV. Supplementary Figure 18. Modification of Kme$_2$ proteins with FITC-cysteine fluorophore.

Synthetic scheme for FITC-Cysteine probe synthesis.

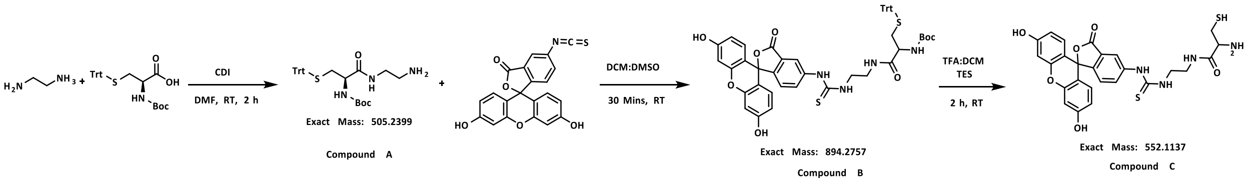

Synthesis of tert-butyl (R)-(1-((2-aminoethyl)amino)-1-oxo-3-(tritylthio)propan-2-yl)carbamate (A). To 1 g of N-Boc-S-trityl-L-cysteine in 10 mL of DMF was added 420 mg (2.6 mmol) of carbonyl diimidazole and reaction stirred at room temperature for 30 min. After 30 min, ethylenediamine (1.4 mL, 21.6 mmol) was added in a single portion to the reaction mixture and stirred for an additional 2 h at room temperature. Upon completion, DMF was removed in vacuo, and residue was dissolved in 50 mL of dichloromethane and washed with water (3 x 50 mL), and saturated brine (50 mL). The resulting extracted solution was dried with anhydrous magnesium sulfate, and solvent was reduced to 1 mL in vacuo and solid was collected by filtration and dried in vacuo.

^{1}**H NMR** (600 MHz, CD_3OD) δ 7.48 – 7.31 (m, 15H), 4.01 (t, 1H), 3.43 (d, *J* = 2.7 Hz, 2H), 3.40

(d, J = 22.7 Hz, 2H), 2.78 (q, J = 5.3, 4.6 Hz, 2H), 2.62 – 2.51 (m, 2H), 1.51 (s, 9H).
^{13}C NMR (151 MHz, CD_3OD) δ 172.15, 156.02, 144.55, 129.35, 127.72, 126.63, 79.62, 66.63, 54.01, 40.78, 40.33, 33.86, 27.41.

Proton NMR of tert-butyl (R)-(1-((2-aminoethyl)amino)-1-oxo-3-(tritylthio)propan-2-yl)carbamate (A) (compound A)

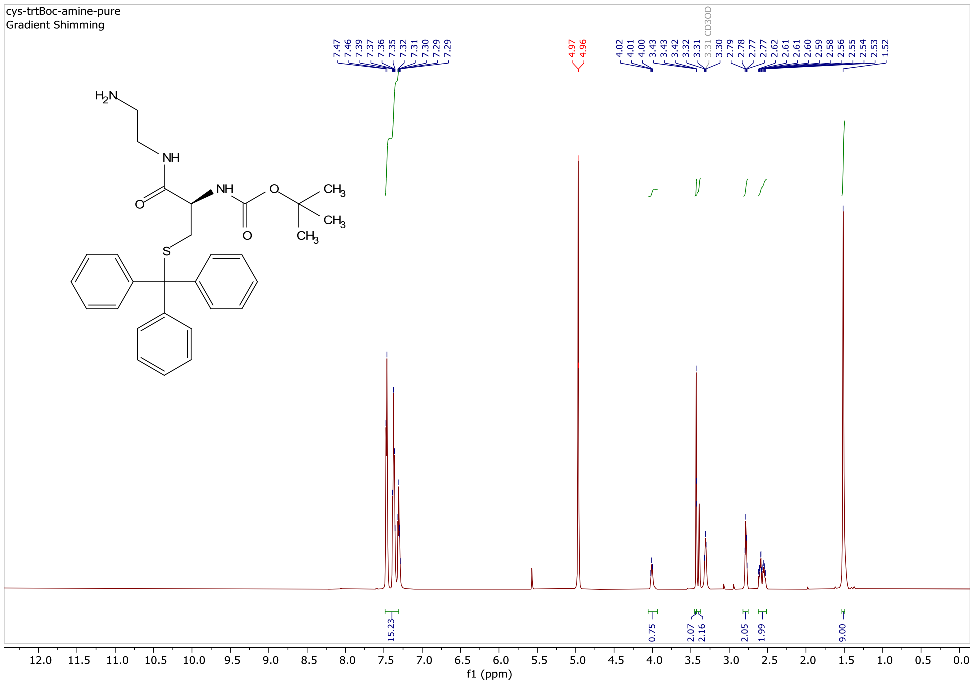

Carbon NMR of tert-butyl (R)-(1-((2-aminoethyl)amino)-1-oxo-3-(tritylthio)propan-2-yl)carbamate (A) (compound A)

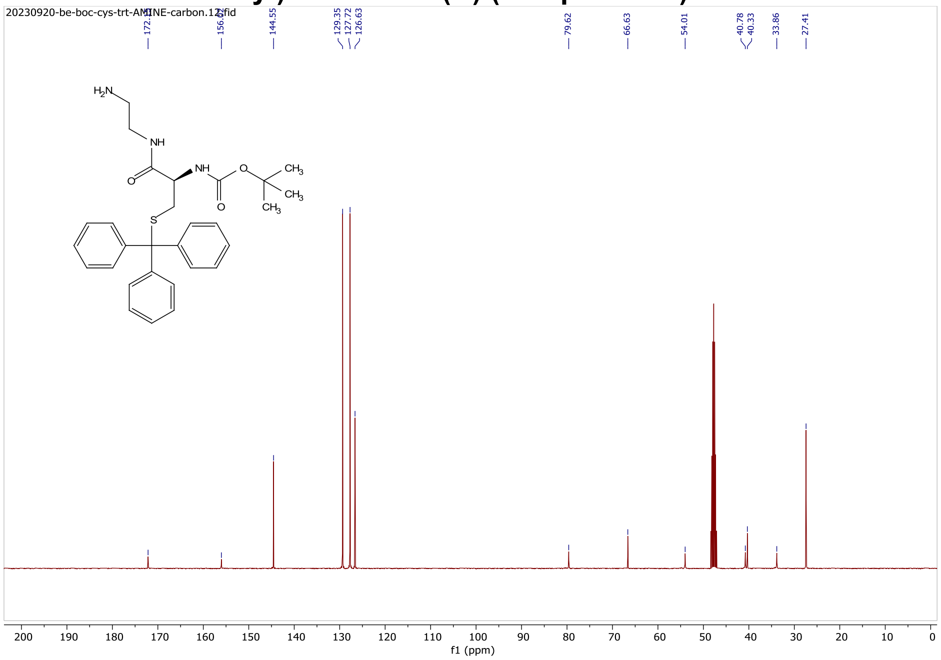

MS-Trace of tert-butyl (R)-(1-((2-aminoethyl)amino)-1-oxo-3-(tritylthio)propan-2-yl)carbamate (A) (compound A)

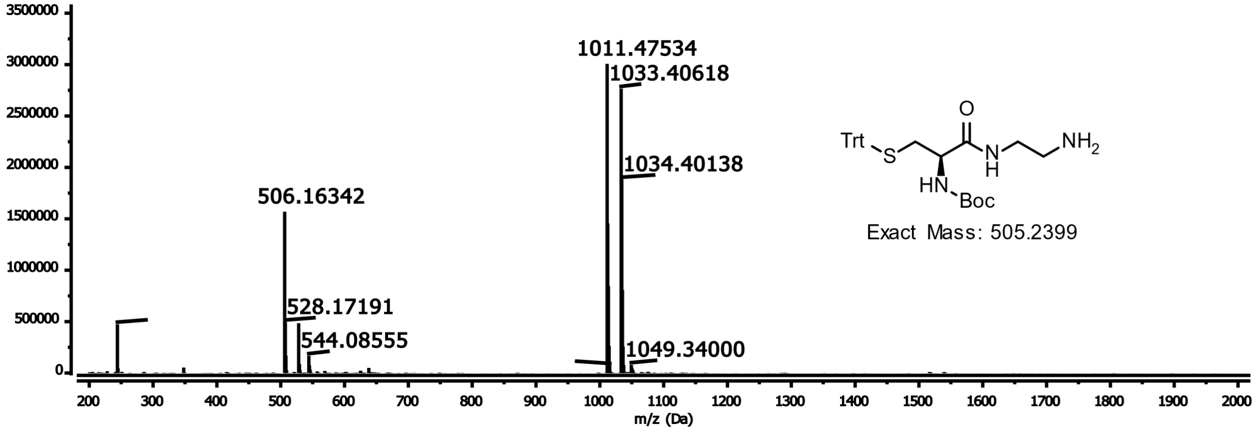

Synthesis of tert-butyl (1-((2-(3-(3',6'-dihydroxy-3-oxo-3H-spiro[isobenzofuran-1,9'-xanthen]-5-yl)thioureido)ethyl)amino)-1-oxo-3-(tritylthio)propan-2-yl)carbamate (B). To 100 mg of tert-butyl (R)-(1-((2-aminoethyl)amino)-1-oxo-3-(tritylthio)propan-2-yl)carbamate in 10 mL of dichloromethane was added 77 mg (1.98 mmol) of fluorescein isothiocyanate dissolved in 1 mL of DMSO. Reaction was stirred at room temperature for 30 min. After 30 min, dichloromethane was removed in vacuo and acetonitrile was added to the residue and purified by preparative HPLC.

^{1}H NMR (400 MHz, DMSO-d_6) δ 10.05 (d, *J* = 32.7 Hz, 2H), 8.18 (d, *J* = 2.0 Hz, 1H), 8.03 (s, 1H), 7.96 (d, *J* = 5.4 Hz, 1H), 7.73 (d, *J* = 8.4 Hz, 1H), 7.36 – 7.21 (m, 15H), 7.15 (d, *J* = 8.3 Hz, 1H), 6.81 (s, 1H), 6.68 (d, *J* = 2.2 Hz, 2H), 6.59 (d, *J* = 8.6 Hz, 2H), 6.56 (dd, *J* = 8.6, 2.2 Hz, 2H), 3.95 (d, *J* = 7.6 Hz, 1H), 3.56 (d, 2H), 2.45 – 2.31 (m, 2H), 1.36 (s, 9H).

^{1}H NMR of tert-butyl (1-((2-(3-(3',6'-dihydroxy-3-oxo-3H-spiro[isobenzofuran-1,9'-xanthen]-5-yl)thioureido)ethyl)amino)-1-oxo-3-(tritylthio)propan-2-yl)carbamate (compound B)

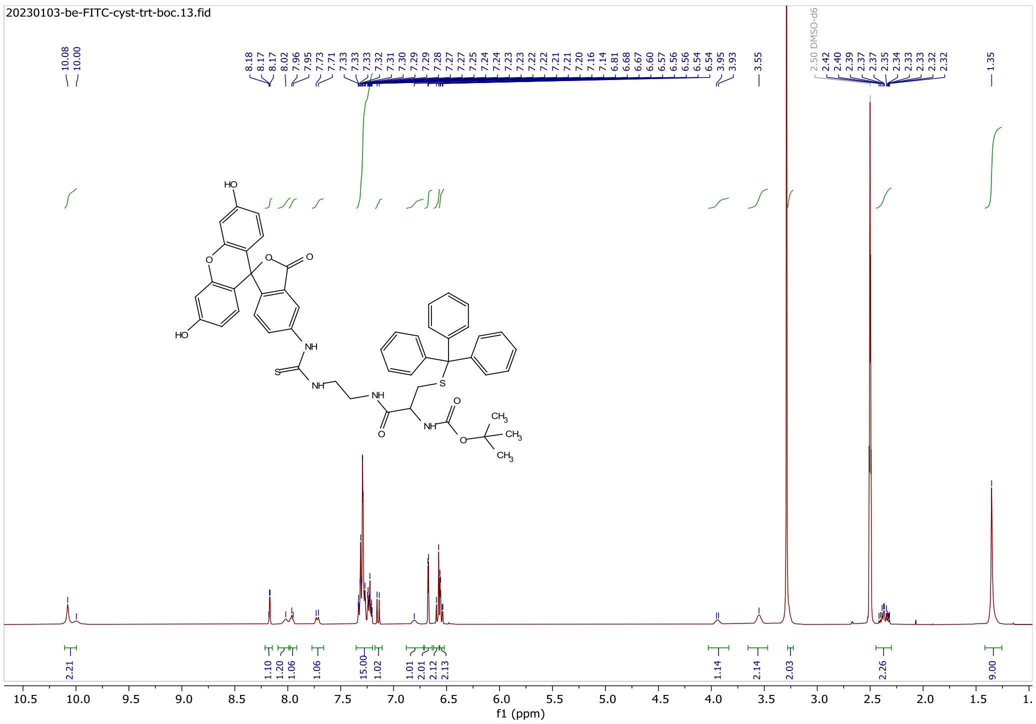

MS-Trace of tert-butyl (1-((2-(3-(3',6'-dihydroxy-3-oxo-3H-spiro[isobenzofuran-1,9'-xanthen]-5-yl)thioureido)ethyl)amino)-1-oxo-3-(tritylthio)propan-2-yl)carbamate (compound B)

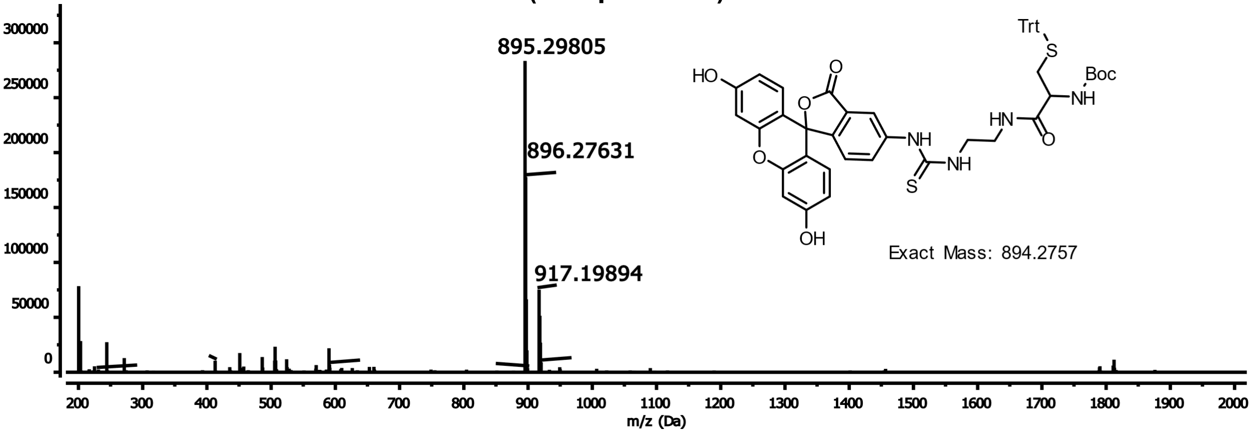

Synthesis of 2-amino-N-(2-(3-(3',6'-dihydroxy-3-oxo-3H-spiro[isobenzofuran-1,9'-xanthen]-5-yl)thioureido)ethyl)-3-mercaptopropanamide (compound C) (FITC-Cysteine). To 100 mg of tert-butyl (1-((2-(3-(3',6'-dihydroxy-3-oxo-3H-spiro[isobenzofuran-1,9'-xanthen]-5-yl)thioureido)ethyl)amino)-1-oxo-3-(tritylthio)propan-2-yl)carbamate in 5 mL of dichloromethane was added 5 mL of trifluoroacetic acid, 300µL of triethyl silane, and 100 µL of water. Reaction was stirred at room temperature for 2 h. After 2 h, the reaction was dried in vacuo and residue

washed with hexane (3x). Solid was dried in vacuo to obtain the title compound C, **FITC-Cysteine** as yellow oil.

^{1}H NMR (400 MHz, DMSO-d_6) δ 10.13 (s, 2H), 8.58 (t, *J* = 5.7 Hz, 1H), 8.21 (d, 1H), 8.19 (t, *J* = 2.1 Hz, 1H), 7.74 (d, *J* = 8.4 Hz, 1H), 7.33 – 7.20 (m, 1H), 7.24 – 7.07 (m, 2H), 6.68 (d, *J* = 2.2 Hz, 2H), 6.63 – 6.57 (m, 2H), 6.57 – 6.53 (m, 1H), 3.95 (t, *J* = 12.7 Hz, 1H), 3.67 (t, *J* = 6.4 Hz, 2H), 3.53 – 3.30 (m, 2H), 3.03 – 2.88 (m, 2H), 2.56 (t, 1H).

^{13}C NMR (151 MHz, DMSO-d_6) δ 181.14, 168.64, 167.64, 167.43, 161.71, 153.33, 142.11, 130.11, 127.68, 125.56, 120.58, 117.43, 114.31, 111.15, 102.69, 54.68, 51.55, 43.05, 38.49, 25.41.

^{1}H of 2-amino-N-(2-(3-(3',6'-dihydroxy-3-oxo-3H-spiro[isobenzofuran-1,9'-xanthen]-5-yl)thioureido)ethyl)-3-mercaptopropanamide (compound C, FITC-Cysteine)

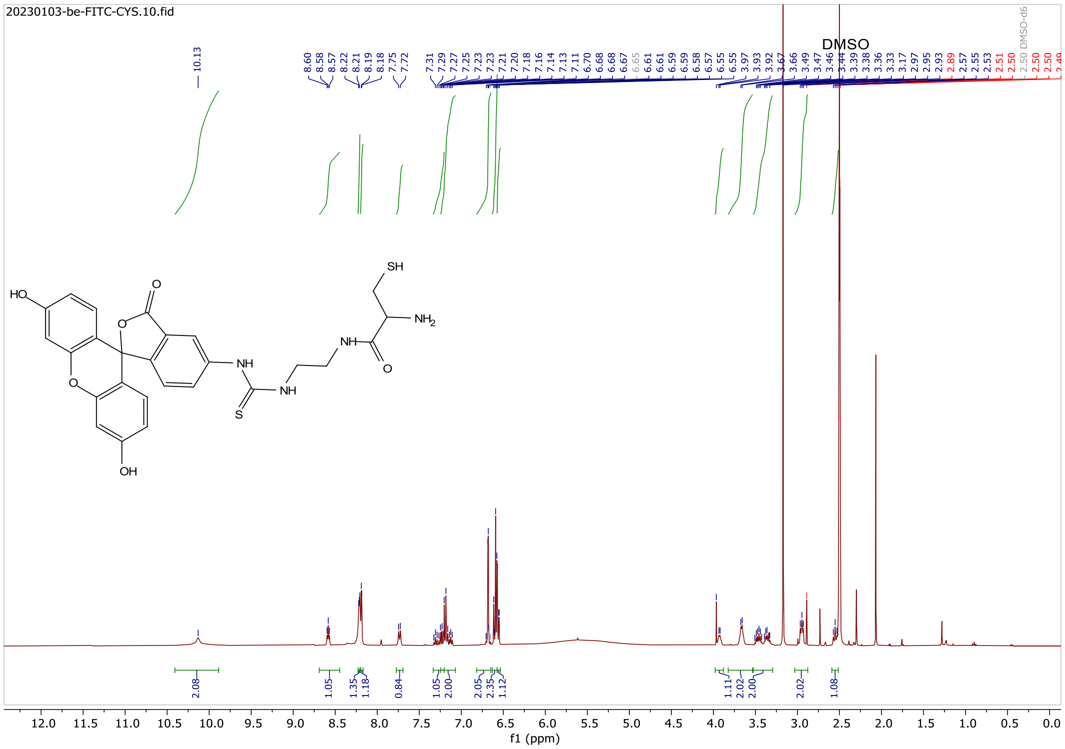

^{13}C of 2-amino-N-(2-(3-(3',6'-dihydroxy-3-oxo-3H-spiro[isobenzofuran-1,9'-xanthen]-5-yl)thioureido)ethyl)-3-mercaptopropanamide (compound C, FITC-Cysteine)

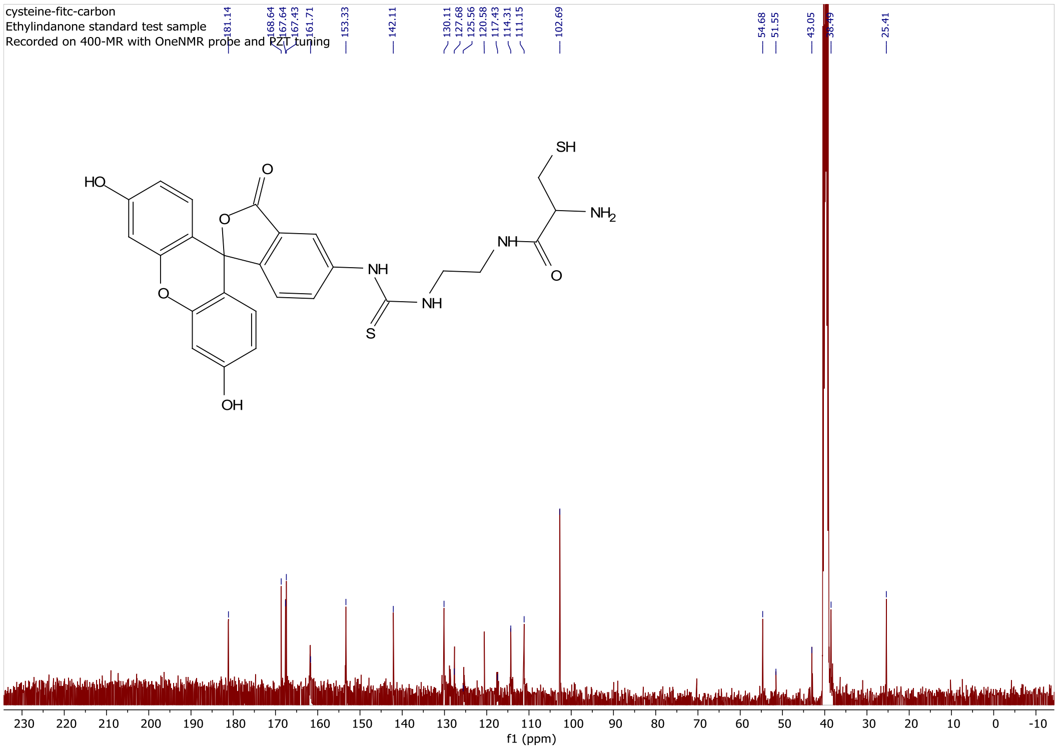

Mass spectrum of 2-amino-N-(2-(3-(3',6'-dihydroxy-3-oxo-3H-spiro[isobenzofuran-1,9'-xanthen]-5-yl)thioureido)ethyl)-3-mercaptopropanamide (compound C, FITC-Cysteine)

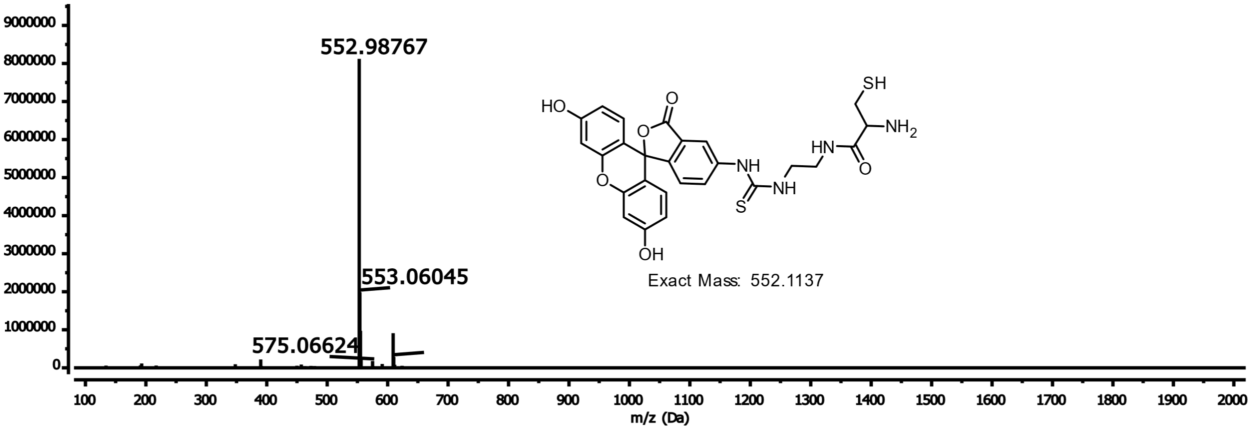

TACO modification of Kme_2 proteins and conjugation with FITC-cysteine: To 2 mg Kme_2 proteins transferrin (T), BSA (B), creatine kinase (C) and myoglobin (M) in 1 mL of NaP buffer pH 7, was added 10 mg of selectfluor and 50 µL of pyridine. The reaction was stirred at room temperature for 30 min and filtered through a molecular weight cutoff to remove small molecules to obtain pure protein. Reaction was filtered with a 3 kDa molecular weight cutoff, and proteins resuspended in 300 µL of PBS buffer. To the sample was added 200µL of 100 µM solution of

FITC-cysteine and incubated for 2 h. Excess fluorophores were washed off through the molecular weight cuttoff. Labelled proteins were then analyzed through in gel fluorescence imaging and coomassie blue staining.

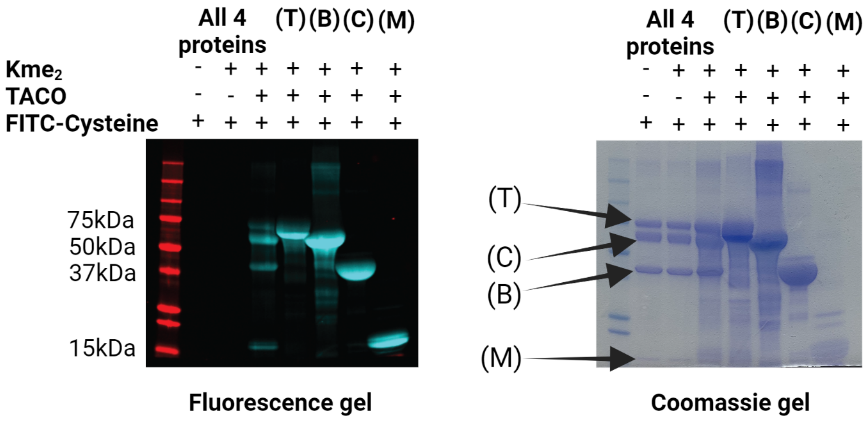

TACO modification of Kme$_2$ proteins and conjugation with Hydroxylamine-647 dye: To 2 mg Kme$_2$ proteins transferrin (T), BSA (B), creatine kinase (C) and myoglobin (M) in 1 mL of NaP buffer pH 7, was added 10 mg of selectfluor and 50 µL of pyridine. The reaction was stirred at room temperature for 30 min and filtered through a molecular weight cutoff to remove small molecules to obtain pure protein. Reaction was filtered with a 3 kDa molecular weight cutoff, and proteins resuspended in 300 µL of PBS buffer. To the sample was added 200µL of 100 µM solution of hydroxylamine-647 and incubated for 2 h. Excess fluorophores were washed off through the molecular weight cuttoff. Labelled proteins were then analyzed through in gel fluorescence imaging and coomassie blue staining.

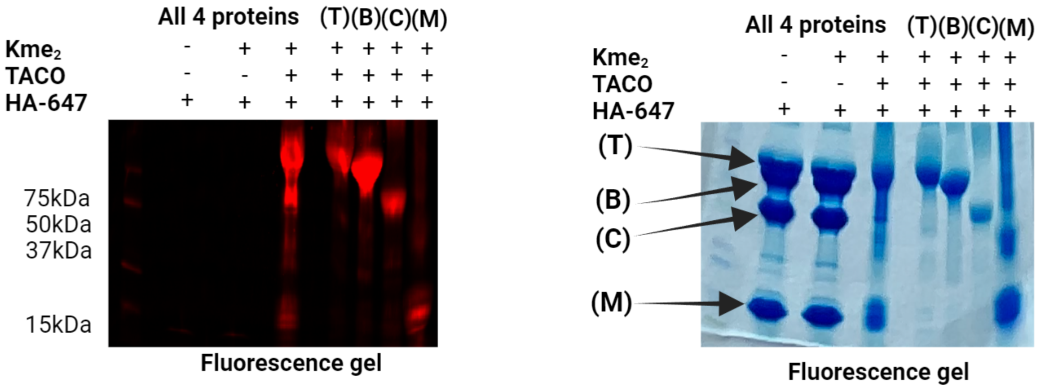

TACO modification of Kme$_2$ proteins and conjugation with Amine-680 dye: To 2 mg Kme$_2$ proteins transferrin (T), BSA (B), creatine kinase (C) and myoglobin (M) in 1 mL of NaP buffer pH 7, was added 10 mg of selectfluor and 50 µL of pyridine. The reaction was stirred at room temperature for 30 min and filtered through a molecular weight cutoff to remove small molecules to obtain pure protein. Reaction was filtered with a 3 kDa molecular weight cutoff, and proteins resuspended in 300 µL of PBS buffer. To the sample was added 200µL of 100 µM solution of Amine-680 dye and incubated for 2 h. Excess fluorophores were washed off through the

molecular weight cuttoff. Labelled proteins were then analyzed through in gel fluorescence imaging and coomassie blue staining.

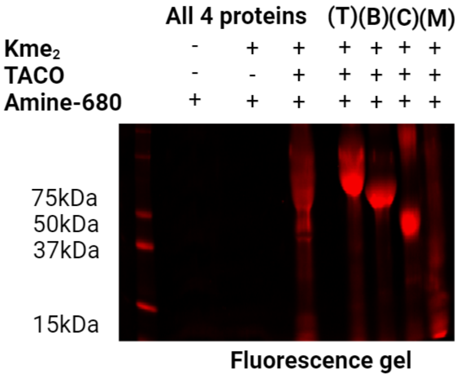

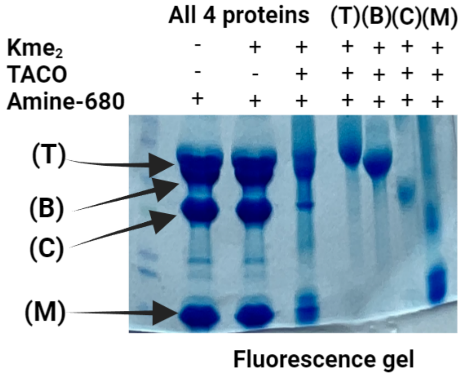

XXV. Supplementary Figure 19. TACO modification of Kme_2 proteins in cell lysates

Dose Dependent labeling of cell lysate with Kme_2 (4%): To 300 µg Lncap cell lysate in 200 µL of NaP buffer (pH 7), was added 50 µL of 4% formaldehyde solution in water. The reaction was vortexed for 2 min, followed by the addition of 50 µL of 600 mM $NaBH_3CN$ solution in water, and vortexed for additional 2 min. The reaction was incubated at room temperature for 16 min, and subjected to acetone precipitated to obtain Kme_2 containing proteins. Lysates were resuspended in 500 µL NaP buffer pH 7.

Dose Dependent labeling of cell lysate with Kme_2 (10%): To 300 µg Lncap cell lysate in 200 µL of NaP buffer (pH 7), was added 50 µL of 10% formaldehyde solution in water. The reaction was vortexed for 2 min, followed by the addition of 50 µL of 600 mM $NaBH_3CN$ solution in water, and vortexed for additional 2 min. The reaction was incubated at room temperature for 16 min, and subjected to acetone precipitated to obtain Kme_2 containing proteins. Lysates were resuspended in 500 µL NaP buffer pH 7.

Dose Dependent labeling of cell lysate with Kme_2 (37%): To 300 µg Lncap cell lysate in 200 µL of NaP buffer (pH 7), was added 50 µL of 37% formaldehyde solution in water. The reaction was vortexed for 2 min, followed by the addition of 50 µL of 600 mM $NaBH_3CN$ solution in water, and vortexed for additional 2 min. The reaction was incubated at room temperature for 16 min, and subjected to acetone precipitated to obtain Kme_2 containing proteins. Lysates were resuspended in 500 µL NaP buffer pH 7.

TACO modification of Kme_2 containing lysates and conjugation of FITC-cysteine: To 300 µg of Kme_2-lysate in 500 µL of NaP buffer was added 2 mg of selectfluor and 10 µL of pyridine. The reaction was stirred at room temperature for 30 min. Samples were subjected to acetone precipitation to remove small molecules and obtain pure proteins, dissolved in 200 µL of PBS buffer pH 7.5 and incubated with 200 µL of 15 mM of iodoacetamide in the dark for 30 min. Proteins in cell lysate were acetone precipitated, resuspended in 100 µL PBS buffer. 200 µL of 100 µM solution of FITC-cysteine (compound C) was added and incubated for 2 h. Excess fluorophores were removed by another round of acetone precipitation, followed by analysis of proteins through in gel fluorescence imaging and Coomassie blue staining. Quantification of gel lanes using Invitrogen ibright FL-1500 image processing software clearly showed a dose-

dependent response (37% lane = 1.0, 10% lane = 0.582, 4% lane = 0.420).

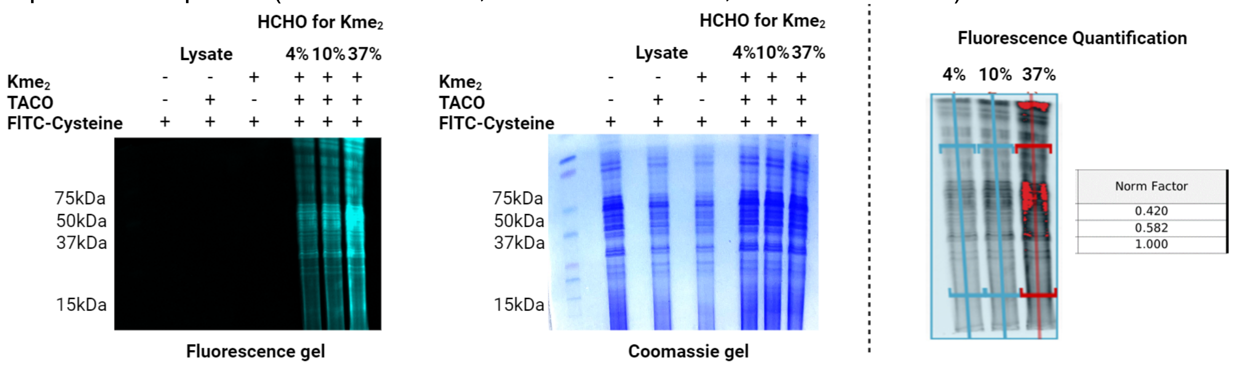

TACO modification of Kme_2 containing lysates and conjugation of Hydroxylamine-647 dye: To 300 µg of Kme_2-lysate in 500 µL of NaP buffer was added 2 mg of selectfluor and 10 µL of pyridine. The reaction was stirred at room temperature for 30 min. Samples were subjected to acetone precipitation to remove small molecules and obtain pure proteins, dissolved in 200 µL of PBS buffer pH 7.5 and incubated with 200 µL of 15 mM of iodoacetamide in the dark for 30 min. Proteins in cell lysate were acetone precipitated, resuspended in 100 µL PBS buffer. 200 µL of 100 µM solution of hydroxylamine-647 dye was added and incubated for 2 h. Excess fluorophores were removed by another round of acetone precipitation, followed by analysis of proteins through in gel fluorescence imaging and Coomassie blue staining. Quantification of gel lanes using Invitrogen ibright FL-1500 image processing software clearly showed a dose-dependent response (37% lane = 1.0, 10% lane = 0.513, 4% lane = 0.459).

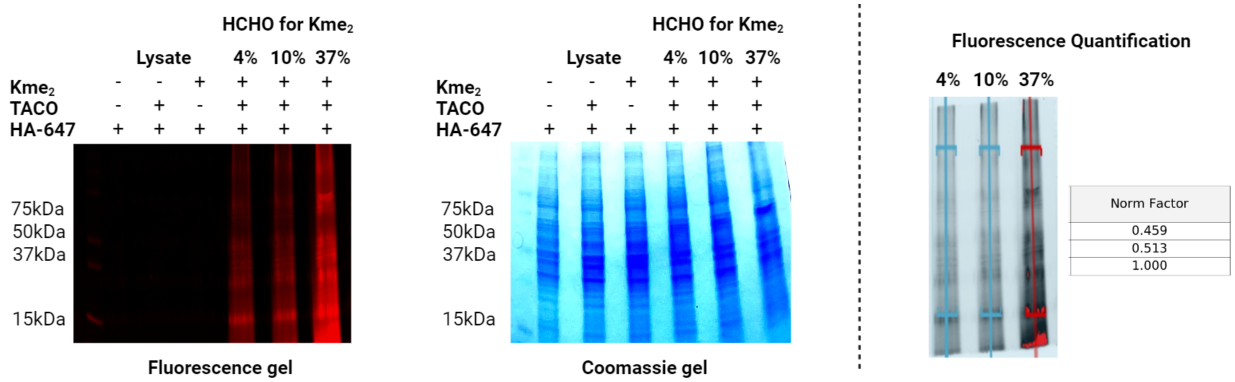

TACO modification of Kme_2 containing lysates and conjugation of Amine-680: To 300 µg of Kme_2-lysate in 500 µL of NaP buffer was added 2 mg of selectfluor and 10 µL of pyridine. The reaction was stirred at room temperature for 30 min. Samples were subjected to acetone precipitation to remove small molecules and obtain pure proteins, dissolved in 200 µL of PBS buffer pH 7.5 and incubated with 200 µL of 15 mM of iodoacetamide in the dark for 30 min. Proteins in cell lysate were acetone precipitated, resuspended in 100 µL PBS buffer. 200 µL of 100 µM solution of amine-680 dye was added and incubated for 2 h. Excess fluorophores were removed by another round of acetone precipitation, followed by analysis of proteins through in gel fluorescence imaging and Coomassie blue staining. Quantification of gel lanes using Invitrogen ibright FL-1500 image processing software clearly showed a dose-dependent response (37% lane = 1.0, 10% lane = 0.672, 4% lane = 0.373).

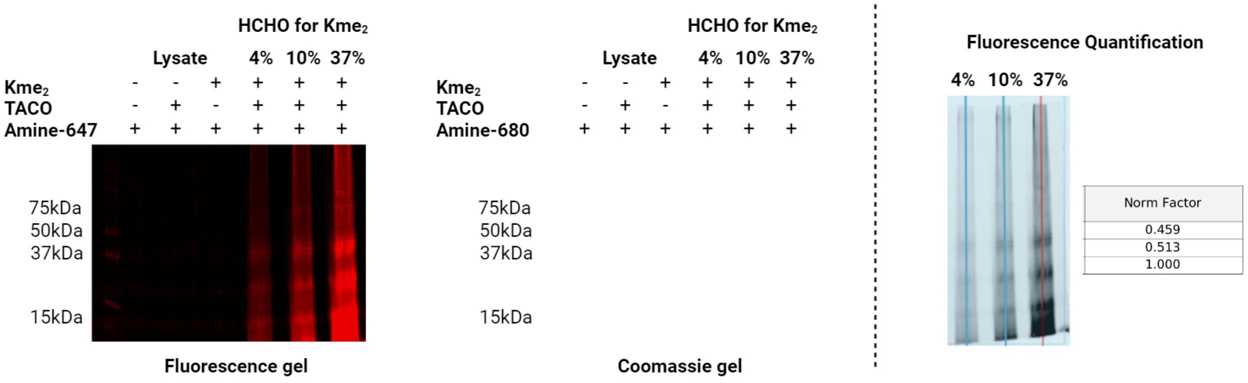

XXVI. Supplementary Figure 20. Solid support mediated enrichment of dimethyllysine containing peptide aldehyde OAcFKme$_2$(CHO)V **2b** by using hydroxylamine solid-support.

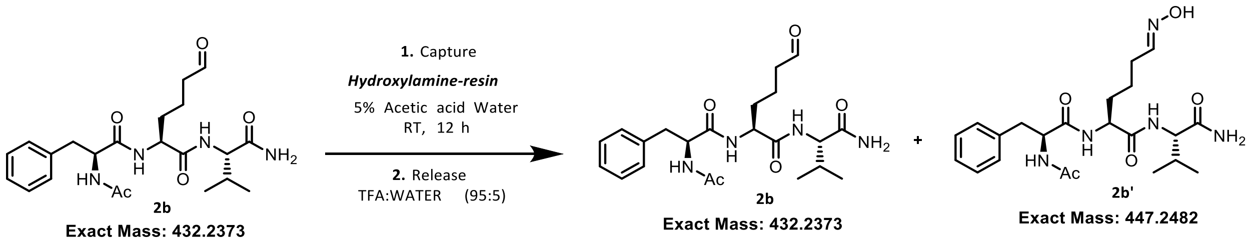

For optimizing the enrichment of aldehyde peptides, we carried out enrichment with a model peptide. To 5 mg of peptide aldehyde OAcFKme$_2$(CHO)V **2b** in 200 µL of NaP buffer pH 7.0 was added hydroxylamine resin. 5% acetic acid in water (500 µL) was added to the resin and reaction was incubated for 12 h. The resin was filtered and washed with methanol (3x), and DMF (3x). Resin was dried and cleaved using 95:5 (TFA:Water) for 2 h at room temperature to release the enriched peptides **2b** and oxime-modified **2b'**. Eluate was concentrated and analyzed using LC-MS.

MS-Trace after the release of peptide aldehyde 2b from resin

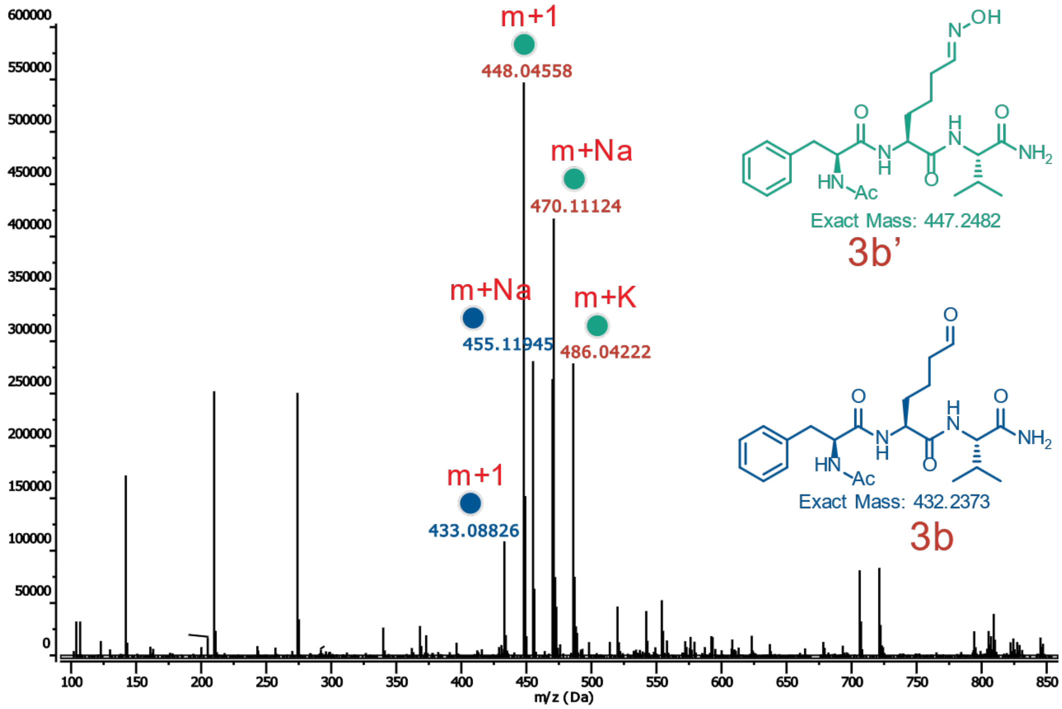

XXVII. Supplementary Figure 21. Solid support mediated enrichment of dimethyllysine containing proteins from nucleosomes obtained from prostate cancer cell lysate.

Cell culture and drugs. Cells were maintained at 37 °C and 5% CO_2. LNCaP cells were cultured in RPMI supplemented with 10% (V/V) fetal bovine serum (FBS) and 1% (V/V) penicillin/streptomycin (100 µg/mL).

Cell lysis and western blotting. Whole cell lysate was generated by lysing cells on ice in RIPA buffer (50 mM TrisHCl [pH 8], 150 mM NaCl, 1% NP-40, 0.5% sodium deoxycholate, 0.1% SDS) supplemented with protease and phosphatase inhibitors. Lysates were centrifuged 6,500 x g, 10 m at 4°C, and soluble lysate was collected. Whole cell lysate proteins were separated using 16% SDS-PAGE. SDS-PAGE gels were stained with coomassie brilliant blue dye and visualized (Odyssey, Li-Cor). Primary antibodies used were as follows: H3 (Abcam).

Nucleosome isolation. Purified nucleosomes were generated by resuspending pelleted cells in lysis buffer (20 mM HEPES [pH 7.5], 0.25 M sucrose, 3 mM $MgCl_2$, 0.5% V/V NP-40, supplemented with protease inhibitors) and Dounce homogenization prior to centrifugation at 3,000 x g, 15 m. Nuclei were subsequently washed twice with lysis buffer, once with Buffer B (20 mM HEPES [pH 7.5], 3 mM $MgCl_2$, 0.2 mM EDTA), once with 0.6 M KCl + 10% glycerol in Buffer B, and pelleted 5,000 x g. Nuclei were then washed in MSB (20 mM HEPES [pH 7.5], 0.4 M NaCl, 1 mM EDTA, 5% V/V glycerol), centrifuged 10,000 x g, 10 m, and resuspended in HSB (20 mM HEPES [pH 7.5], 0.65 M NaCl, 1 mM EDTA, 0.34 M sucrose, supplemented with protease inhibitors) and Dounce homogenized to release oligonucleosome fragments.

Oligonucleosomes were then centrifuged 10,000 x g, 20 m and supernatant dialyzed o/n against LSB (20 mM HEPES [pH 7.5], 0.1 M NaCl, 1 mM EDTA,) with 6-8 kDa MWCO membranes. The next day, dialysate was collected and $CaCl_2$ was added to a final concentration of 3 mM and incubated at 37 ^{o}C for 5 m, after which micrococcal nuclease (Sigma) was added to a final concentration of 10 U/mL and incubated for 10 m at 37 ^{o}C. Digestion was stopped with 0.1 volumes of 0.5 M EDTA on ice, and NaCl was added to a final concentration of 0.6 M. Mononucleosomes were separated via ultracentrifugation by layering 1 mL of material onto equilibrated linear gradients in 1 x 3.5-inch polyallomer centrifuge tubes (Beckman) with 1 mL each of HSB + 10% V/V glycerol, HSB + 20% V/V glycerol, HSB + 30% V/V glycerol, HSB + 40% V/V glycerol, from top to bottom. Samples were centrifuged 100,000 x g, 16 h, in an ultracentrifuge equipped with a SW-55Ti rotor (Beckman). 500 µL fractions were harvested from top to bottom and nucleosomes were assessed via 16% SDS PAGE followed by coomassie stain. Fractions containing nucleosomes were pooled for subsequent experimentation.

TACO modification and enrichment of modified nucleosome. To 100 µg of prostate cancer cell nucleosome extract in 200 µL NaP buffer (pH 7), was added 2 mg of selectfluor and 10 µL of pyridine. The reaction mixture was stirred for 1 h. Reaction mixture was filtered using a 3 kDa molecular weight cut off to remove small molecules. To rest of the solution was added hydroxylamine solid support resin, 5% acetic acid in water (500 µL) and reaction incubated for 12 h. The resin was filtered and washed with methanol (3x), and DMF (3x). Resin was dried and cleaved using 95:5 (TFA: Water) to release the enriched nucleosomes. Eluate was concentrated followed by acetone precipitation. The eluate and filtrate were analyzed using SDS PAGE. This reaction was performed in duplicates.

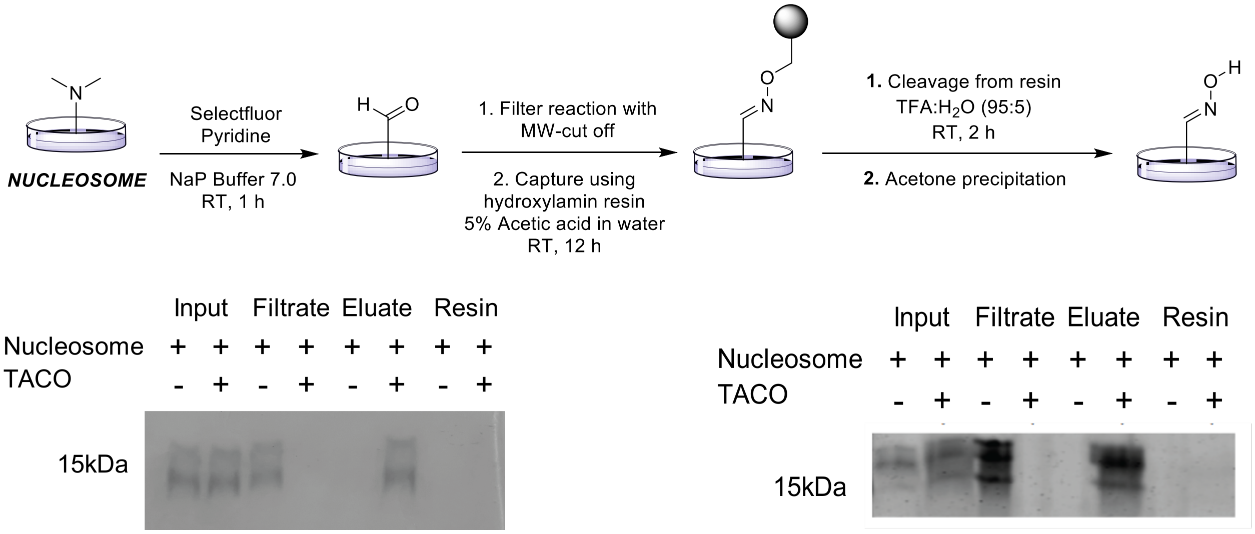

XXVIII. Supplementary Figure 22. TACO modification of nuclear extract and proteomics analysis.

Nuclear extract isolation. Nuclear extracts were generated using a modified protocol of Active Motif Nuclear Extract Kit. Pelleted cells were resuspended in hypotonic lysis buffer (20 mM TrisHCl [pH 7.5], 150 mM NaCl, 5 mM $MgCl_2$, 1% V/V NP-40, supplemented with protease inhibitors) and incubated on ice for 15 min. 50 µL of detergent was added and vortexed for 10 seconds. Samples were dounce homogenized prior to centrifugation at 14,000 x g (5415 rotor)

for 1 m. Cytoplasmic extracts (supernatant) were transferred and nuclear extract (pellet) resuspended in complete lysis buffer and 15 µL of detergent. Sample was dounce homogenized and vortexed for 10 seconds and incubated on ice for 30 m, followed by centrifugation at 14,000 x g (12200 rpm for 5415 rotor) for 10 m to obtain the nuclear extract (supernatant).

TACO modification and propargylation of nuclear extract: To 500 µg of nuclear extract in 500 µL of NaP buffer pH 7 was added 5 mg of selectfluor and 20 µL of pyridine. The reaction was stirred at room temperature for 1 h. Samples were subjected to acetone precipitation to remove small molecules to obtain pure proteins. To proteins resuspended in 500 µL of NaP buffer pH 5.0 was added 5 µL of propargylamine and stirred for 1 h, followed by the addition of 5 mg sodium cyanoborohydride. Reaction was stirred for 5 h followed by acetone precipitation.

Proteomics analysis of proteins: Proteins were digested using Thermo scientific EasyPep Mini MS sample Prep Kit. Digested peptides were analyzed by LC/MS. Samples were analyzed by LC-MS using a Q-Exactive Plus orbitrap mass spectrometer equipped with Dionex UltiMate 3000 LC system (Thermo). Briefly, lyophilized digested peptides were resuspended in 0.1% formic acid in 10% acetonitrile and loaded onto a trap column (PepMap™ NEO 5 µm C18 300 µm X 5 mm Trap Cartridge) and resolved through a custom analytical column packed with ReproSil-Pur 120 C18-AQ 3 µm beads (Dr. Maisch GmbH) at a flow rate of 0.3 µL/min with a gradient solvent A (0.1% formic acid in 2% acetonitrile) and a gradient solvent B (0.1% formic acid in 80% acetonitrile) for 150 minutes. MS analysis was conducted in a data-dependent manner with full scans in the range from 400 to 1800 m/z using an Orbitrap mass analyzer set as follows: MS1: resolution = 70,000, AGC target = 3e6, Max IT = 100ms; MS2: resolution = 17,500, AGC target 1e5, Max IT = 50ms. The top fifteen most intense precursor ions were selected for MS2 with an isolation window of 4 m/z. Isolated precursors were fragmented by high energy collisional dissociation (HCD) with normalized collision energy (NCE) of 27. LC-MS RAW files were searched against the human proteome sequence database from UniProt using the Sequest HT search engine embedded in Proteome Discoverer 3.0 (Thermo) with 10 ppm MS1 precursor mass tolerance, 0.02 Da MS2 fragment mass tolerance, 0.01 false discovery rate. Search included the following modifications: Methionine oxidation (+15.99492 Da), asaparagine and glutamine deamidation (+0.98402 Da) and protein N-terminal acetylation (+42.03670), histidine fluorination (+17.9906), tryptophan fluorination (+33.9855), lysine propargylation (+38.0157), and lysine-to-aldehyde (-1.0316) were variable modifications (up to 3 allowed per peptide); cysteine was assigned a fixed carbamidomethyl modification (+57.021465 Da).

Representative peptide spectra of modified peptides (propargyl and aldehyde sites, +38 on lysine)
+TACO modified peptides: <u>All these peptides were identified after the TACO chemistry on the nuclear extract obtained from LnCap</u>.

A5A3E0 (POTE ankyrin domain family member F)

Sequence: VAPEEHPVLLTEATLNPKANR, K18-Ben_K1 (38.01570 Da) (position: lysine 813)

#1	b⁺	b²⁺	b³⁺	Seq.	y⁺	y²⁺	y³⁺	#2
1	100.07569	50.54148	34.03008	V				21
2	171.11280	86.06004	57.70912	A	2238.187...	1119.597...	746.73410	20
3	268.16557	134.58642	90.06004	P	2167.150...	1084.078...	723.05507	19
4	397.20816	199.10772	133.07424	E	2070.097...	1035.552...	690.70414	18
5	526.25075	263.62902	176.08844	E	1941.055...	971.03128	647.68995	17
6	663.30967	332.15847	221.77474	H	1812.012...	906.50998	604.67575	16
7	760.36243	380.68485	254.12566	P	1674.953...	837.98053	558.98944	15
8	859.43084	430.21906	287.14847	V	1577.901...	789.45415	526.63852	14
9	972.51491	486.76109	324.84315	L	1478.832...	739.91994	493.61572	13
10	1085.598...	543.30312	362.53784	L	1365.748...	683.37791	455.92103	12
11	1186.646...	593.82696	396.22040	T	1252.664...	626.83588	418.22634	11
12	1315.689...	658.34826	439.23460	E	1151.616...	576.31204	384.54378	10
13	1386.726...	693.86682	462.91364	A	1022.574...	511.79074	341.52959	9
14	1487.774...	744.39066	496.59620	T	951.53709	476.27218	317.85055	8
15	1600.858...	800.93269	534.29088	L	850.48941	425.74834	284.16799	7
16	1714.901...	857.95415	572.30519	N	737.40535	369.20631	246.47330	6
17	1811.953...	906.48053	604.65611	P	623.36242	312.18485	208.45899	5
18	1978.064...	989.53586	660.02634	K-Ben...	526.30966	263.65847	176.10807	4
19	2049.101...	1025.054...	683.70537	A	360.19899	180.60314	120.73785	3
20	2163.144...	1082.075...	721.71968	N	289.16188	145.08458	97.05881	2
21				R	175.11895	88.06311	59.04450	1

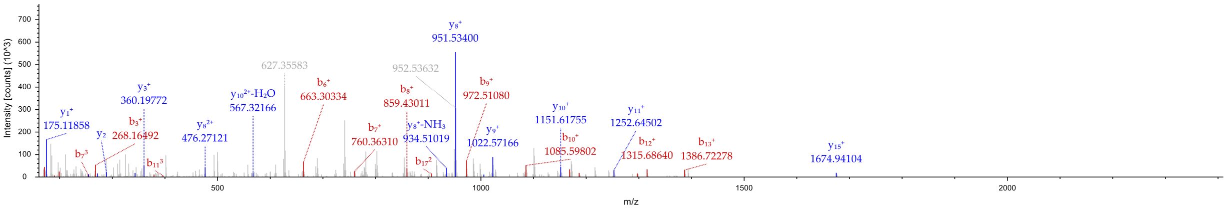

A0A1W2PQ09 (TATA-box-binding protein-associated factor 11-like protein 11)

Sequence: SVSENTAIAMAGIAK, K15-Ben_K1 (38.01570 Da) (position: lysine 147)

#1	b⁺	b²⁺	b³⁺	Seq.	y⁺	y²⁺	y³⁺	#2
1	88.03930	44.52329	30.01795	S				15
2	187.10772	94.05750	63.04076	V	1413.740...	707.37398	471.91841	14
3	274.13975	137.57351	92.05143	S	1314.672...	657.83977	438.89561	13
4	403.18234	202.09481	135.06563	E	1227.640...	614.32376	409.88493	12
5	517.22527	259.11627	173.07994	N	1098.597...	549.80246	366.87073	11
6	618.27295	309.64011	206.76250	T	984.55471	492.78100	328.85642	10
7	689.31006	345.15867	230.44154	A	883.50704	442.25716	295.17386	9
8	802.39412	401.70070	268.13623	I	812.46992	406.73860	271.49482	8
9	873.43124	437.21926	291.81526	A	699.38586	350.19657	233.80014	7
10	1004.471...	502.73950	335.49543	M	628.34874	314.67801	210.12110	6
11	1075.508...	538.25806	359.17446	A	497.30826	249.15777	166.44094	5
12	1132.530...	566.76879	378.18162	G	426.27115	213.63921	142.76190	4
13	1245.614...	623.31082	415.87631	I	369.24968	185.12848	123.75475	3
14	1316.651...	658.82938	439.55534	A	256.16562	128.58645	86.06006	2
15				K-Ben...	185.12850	93.06789	62.38102	1

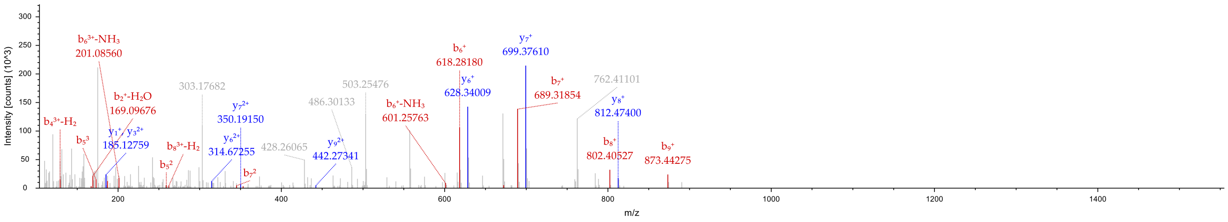

P12259 (Coagulation factor V)

Sequence: WIISSLTPKHLQAGMQAYIDIK, M15-Ben_Met2 (15.99490 Da), K22-Ben_K1 (38.01570 Da) (position: lysine 327)

#1	b⁺	b²⁺	b³⁺	Seq.	y⁺	y²⁺	y³⁺	#2
1	187.08659	94.04693	63.03371	W				22
2	300.17065	150.58897	100.72840	I	2381.289...	1191.148...	794.43477	21
3	413.25472	207.13100	138.42309	I	2268.205...	1134.606...	756.74008	20
4	500.28675	250.64701	167.43377	S	2155.121...	1078.064...	719.04540	19
5	587.31877	294.16303	196.44444	S	2068.089...	1034.548...	690.03472	18
6	700.40284	350.70506	234.13913	L	1981.057...	991.03243	661.02404	17
7	801.45052	401.22890	267.82169	T	1867.973...	934.49040	623.32936	16
8	898.50328	449.75528	300.17261	P	1766.925...	883.96656	589.64680	15
9	1026.598...	513.80276	342.87093	K	1669.873...	835.44017	557.29588	14
10	1163.657...	582.33222	388.55724	H	1541.778...	771.39269	514.59755	13
11	1276.741...	638.87425	426.25192	L	1404.719...	702.86324	468.91125	12
12	1404.799...	702.90354	468.93812	Q	1291.635...	646.32121	431.21656	11
13	1475.836...	738.42209	492.61715	A	1163.576...	582.29192	388.53037	10
14	1532.858...	766.93283	511.62431	G	1092.539...	546.77336	364.85133	9
15	1679.893...	840.45052	560.63610	M-Ben...	1035.517...	518.26263	345.84418	8
16	1807.952...	904.47981	603.32230	Q	888.48259	444.74494	296.83238	7
17	1878.989...	939.99836	627.00133	A	760.42402	380.71565	254.14619	6
18	2042.052...	1021.530...	681.35578	Y	689.38690	345.19709	230.46715	5
19	2155.136...	1078.072...	719.05047	I	526.32358	263.66543	176.11271	4
20	2270.163...	1135.585...	757.39278	D	413.23951	207.12339	138.41802	3
21	2383.247...	1192.127...	795.08747	I	298.21257	149.60992	100.07571	2
22				K-Ben...	185.12850	93.06789	62.38102	1

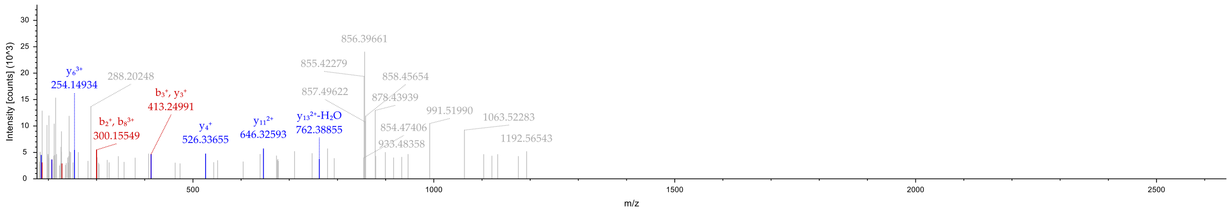

Q09666 (Neuroblast differentiation-associated protein AHNAK)

Sequence: ISMPDVDLHVKGTK, M3-Ben_Met2 (15.99490 Da), K11-Ben_K1 (38.01570 Da) (position: lysine 707)

#1	b⁺	b²⁺	b³⁺	Seq.	y⁺	y²⁺	y³⁺	#2
1	114.09134	57.54931	38.70196	I				14
2	201.12337	101.06532	67.71264	S	1480.746...	740.87688	494.25368	13
3	348.15875	174.58302	116.72444	M-Ben...	1393.714...	697.36086	465.24300	12
4	445.21152	223.10940	149.07536	P	1246.679...	623.84317	416.23121	11
5	560.23846	280.62287	187.41767	D	1149.626...	575.31679	383.88028	10
6	659.30687	330.15708	220.44048	V	1034.599...	517.80332	345.53797	9
7	774.33382	387.67055	258.78279	D	935.53094	468.26911	312.51517	8
8	887.41788	444.21258	296.47748	L	820.50400	410.75564	274.17285	7
9	1024.476...	512.74203	342.16378	H	707.41993	354.21361	236.47816	6
10	1123.545...	562.27624	375.18659	V	570.36102	285.68415	190.79186	5
11	1289.655...	645.33157	430.55681	K-Ben...	471.29261	236.14994	157.76905	4
12	1346.677...	673.84230	449.56396	G	305.18195	153.09461	102.39883	3
13	1447.725...	724.36614	483.24652	T	248.16048	124.58388	83.39168	2
14				K	147.11280	74.06004	49.70912	1

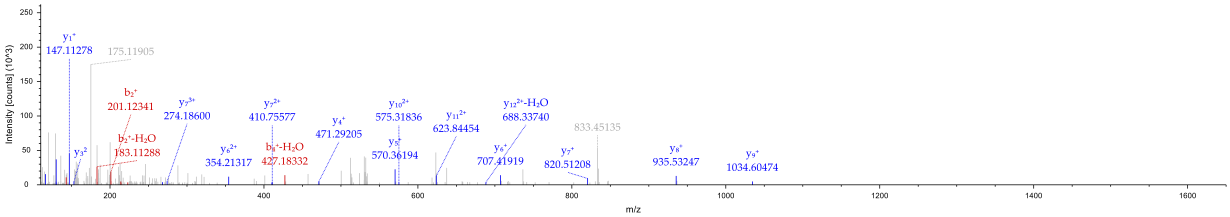

O94760 (N(G),N(G)-dimethylarginine dimethylaminohydrolase 1)

Sequence: SAKGEEVDVAR, K3-Ben_K1 (38.01570 Da) (position: lysine 34)

#1	b+	b2+	Seq.	y+	y2+	#2
1	88.03930	44.52329	S			11
2	159.07642	80.04185	A	1111.574...	556.29077	10
3	325.18708	163.09718	K-Ben...	1040.537...	520.77221	9
4	382.20855	191.60791	G	874.42649	437.71688	8
5	511.25114	256.12921	E	817.40502	409.20615	7
6	640.29373	320.65050	E	688.36243	344.68485	6
7	739.36215	370.18471	V	559.31984	280.16356	5
8	854.38909	427.69818	D	460.25142	230.62935	4
9	953.45750	477.23239	V	345.22448	173.11588	3
10	1024.494...	512.75095	A	246.15607	123.58167	2
11			R	175.11895	88.06311	1

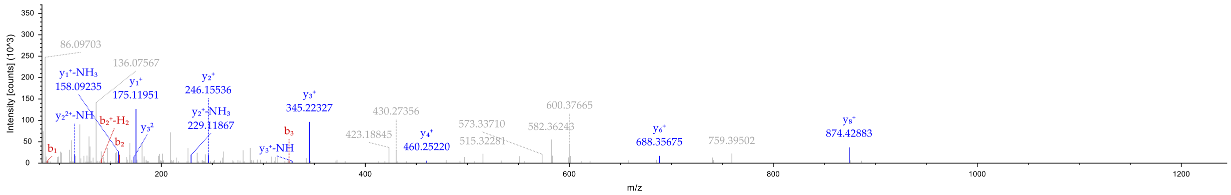

Representative peptide spectra of modified peptides (aldehydes on lysine, -1 on lysine) +TACO modified peptides:

P68431 (Histone H3.1)

Sequence: VTIMPKDIQLAR, M4-Ben_Met2 (15.99490 Da), K6-Ben_K2 (-1.03160 Da) (position: lysine 123)

#1	b⁺	b²⁺	Seq.	y⁺	y²⁺	#2
1	100.07569	50.54148	V			12
2	201.12337	101.06532	T	1300.692...	650.85012	11
3	314.20743	157.60735	I	1199.645...	600.32628	10
4	461.24282	231.12505	M-Ben...	1086.561...	543.78425	9
5	558.29558	279.65143	P	939.52584	470.26656	8
6	685.35894	343.18311	K-Ben...	842.47308	421.74018	7
7	800.38589	400.69658	D	715.40971	358.20850	6
8	913.46995	457.23861	I	600.38277	300.69502	5
9	1041.528...	521.26790	Q	487.29871	244.15299	4
10	1154.612...	577.80993	L	359.24013	180.12370	3
11	1225.649...	613.32849	A	246.15607	123.58167	2
12			R	175.11895	88.06311	1

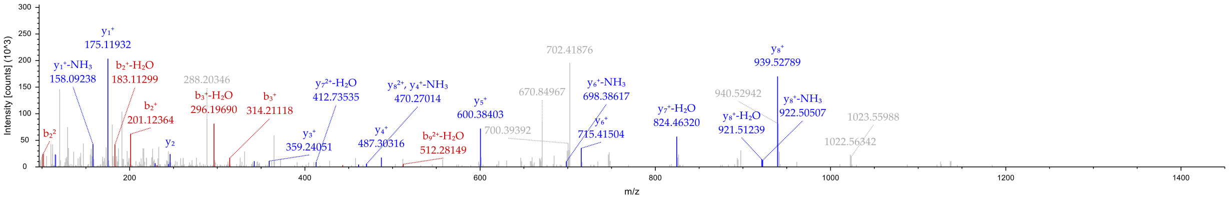

P68431 (Histone H3.1)

Sequence: EIAQDFKTDLR, K7-Ben_K2 (-1.03160 Da) (position: lysine 80)

#1	b⁺	b²⁺	Seq.	y⁺	y²⁺	#2
1	130.04987	65.52857	E			11
2	243.13393	122.07061	I	1205.616...	603.31169	10
3	314.17105	157.58916	A	1092.532...	546.76966	9
4	442.22962	221.61845	Q	1021.494...	511.25111	8
5	557.25657	279.13192	D	893.43636	447.22182	7
6	704.32498	352.66613	F	778.40941	389.70835	6
7	831.38834	416.19781	K-Ben...	631.34100	316.17414	5
8	932.43602	466.72165	T	504.27764	252.64246	4
9	1047.462...	524.23512	D	403.22996	202.11862	3
10	1160.547...	580.77715	L	288.20302	144.60515	2
11			R	175.11895	88.06311	1

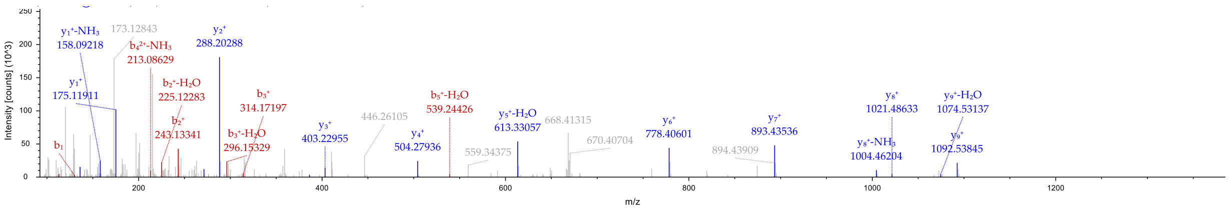

Q71DI3 (Histone 3.2)

Sequence: EIAQDFKTDLR, K7-Ben_K2 (-1.03160 Da) (position: lysine 80)

#1	b+	b2+	Seq.	y+	y2+	#2
1	130.04987	65.52857	E			11
2	243.13393	122.07061	I	1205.616...	603.31169	10
3	314.17105	157.58916	A	1092.532...	546.76966	9
4	442.22962	221.61845	Q	1021.494...	511.25111	8
5	557.25657	279.13192	D	893.43636	447.22182	7
6	704.32498	352.66613	F	778.40941	389.70835	6
7	831.38834	416.19781	K-Ben...	631.34100	316.17414	5
8	932.43602	466.72165	T	504.27764	252.64246	4
9	1047.462...	524.23512	D	403.22996	202.11862	3
10	1160.547...	580.77715	L	288.20302	144.60515	2
11			R	175.11895	88.06311	1

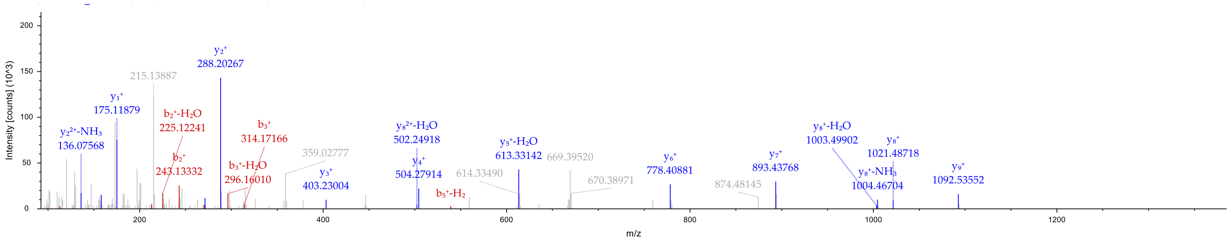

P62805 (Histone H4)

Sequence: DNIQGITKPAIR, K8-Ben_K2 (-1.03160 Da) (position: lysine 32)

#1	b+	b2+	Seq.	y+	y2+	#2
1	116.03422	58.52075	D			12
2	230.07715	115.54221	N	1209.695...	605.35115	11
3	343.16121	172.08424	I	1095.652...	548.32969	10
4	471.21979	236.11353	Q	982.56804	491.78766	9
5	528.24125	264.62426	G	854.50946	427.75837	8
6	641.32532	321.16630	I	797.48800	399.24764	7
7	742.37299	371.69014	T	684.40394	342.70561	6
8	869.43636	435.22182	K-Ben...	583.35626	292.18177	5
9	966.48912	483.74820	P	456.29289	228.65009	4
10	1037.526...	519.26676	A	359.24013	180.12370	3
11	1150.610...	575.80879	I	288.20302	144.60515	2
12			R	175.11895	88.06311	1

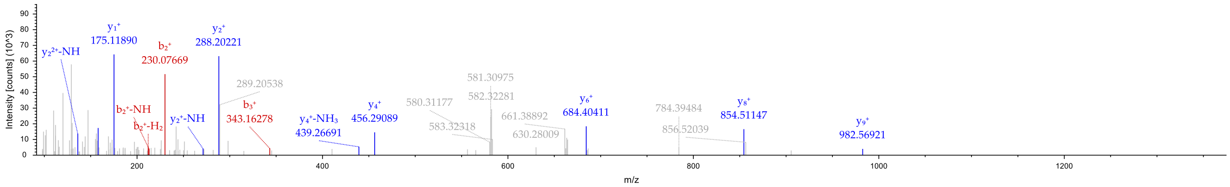

P68431 (Histone H2B type 1-K)

Sequence: LLLPGELAKHAVSEGTK, K9-Ben_K2 (-1.03160 Da) (position: lysine 109)

#1	b^{+}	b^{2+}	b^{3+}	Seq.	y^{+}	y^{2+}	y^{3+}	#2
1	114.09134	57.54931	38.70196	L				17
2	227.17540	114.09134	76.39665	L	1648.890...	824.94889	550.30168	16
3	340.25947	170.63337	114.09134	L	1535.806...	768.40685	512.60700	15
4	437.31223	219.15975	146.44226	P	1422.722...	711.86482	474.91231	14
5	494.33370	247.67049	165.44942	G	1325.669...	663.33844	442.56139	13
6	623.37629	312.19178	208.46361	E	1268.648...	634.82771	423.55423	12
7	736.46035	368.73381	246.15830	L	1139.605...	570.30641	380.54003	11
8	807.49747	404.25237	269.83734	A	1026.521...	513.76438	342.84535	10
9	934.56083	467.78405	312.19179	K-Ben...	955.48437	478.24582	319.16631	9
10	1071.619...	536.31351	357.87810	H	828.42101	414.71414	276.81185	8
11	1142.656...	571.83207	381.55714	A	691.36210	346.18469	231.12555	7
12	1241.725...	621.36627	414.57994	V	620.32498	310.66613	207.44651	6
13	1328.757...	664.88229	443.59062	S	521.25657	261.13192	174.42371	5
14	1457.799...	729.40358	486.60481	E	434.22454	217.61591	145.41303	4
15	1514.821...	757.91432	505.61197	G	305.18195	153.09461	102.39883	3
16	1615.869...	808.43815	539.29453	T	248.16048	124.58388	83.39168	2
17				K	147.11280	74.06004	49.70912	1

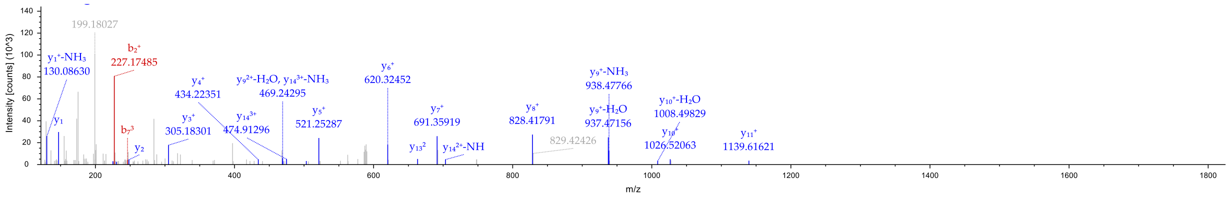

P07910 (Heterogeneous nuclear ribonucleoproteins C1/C2)

Sequence: MIAGQVLDINLAAEPKVNR, M1-Ben_Met2 (15.99490 Da), K16-Ben_K2 (-1.03160 Da) (position: lysine 89)

#1	b^{+}	b^{2+}	Seq.	y^{+}	y^{2+}	#2
1	148.04266	74.52497	M-Ben...			19
2	261.12673	131.06700	I	1920.054...	960.53111	18
3	332.16384	166.58556	A	1806.970...	903.98907	17
4	389.18530	195.09629	G	1735.933...	868.47052	16
5	517.24388	259.12558	Q	1678.912...	839.95979	15
6	616.31229	308.65979	V	1550.853...	775.93050	14
7	729.39636	365.20182	L	1451.785...	726.39629	13
8	844.42330	422.71529	D	1338.701...	669.85426	12
9	957.50736	479.25732	I	1223.674...	612.34079	11
10	1071.550...	536.27878	N	1110.590...	555.79875	10
11	1184.634...	592.82082	L	996.54730	498.77729	9
12	1255.671...	628.33937	A	883.46324	442.23526	8
13	1326.708...	663.85793	A	812.42613	406.71670	7
14	1455.751...	728.37923	E	741.38901	371.19814	6
15	1552.803...	776.90561	P	612.34642	306.67685	5
16	1679.867...	840.43729	K-Ben...	515.29366	258.15047	4
17	1778.935...	889.97150	V	388.23029	194.61879	3
18	1892.978...	946.99296	N	289.16188	145.08458	2
19			R	175.11895	88.06311	1

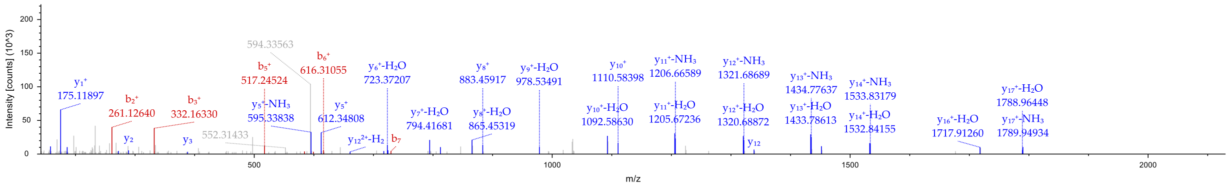

P63261 (Actin cytoplasmic 2)

Sequence: VAPEEHPVLLTEAPLNPKANR, K18-Ben_K2 (-1.03160 Da) (position: lysine 113)

#1	b⁺	b²⁺	b³⁺	Seq.	y⁺	y²⁺	y³⁺	#2
1	100.07569	50.54148	34.03008	V				21
2	171.11280	86.06004	57.70912	A	2195.145...	1098.076...	732.38670	20
3	268.16557	134.58642	90.06004	P	2124.108...	1062.557...	708.70766	19
4	397.20816	199.10772	133.07424	E	2027.055...	1014.031...	676.35674	18
5	526.25075	263.62902	176.08844	E	1898.013...	949.51017	633.34254	17
6	663.30967	332.15847	221.77474	H	1768.970...	884.98888	590.32834	16
7	760.36243	380.68485	254.12566	P	1631.911...	816.45942	544.64204	15
8	859.43084	430.21906	287.14847	V	1534.858...	767.93304	512.29112	14
9	972.51491	486.76109	324.84315	L	1435.790...	718.39883	479.26831	13
10	1085.598...	543.30312	362.53784	L	1322.706...	661.85680	441.57363	12
11	1186.646...	593.82696	396.22040	T	1209.622...	605.31477	403.87894	11
12	1315.689...	658.34826	439.23460	E	1108.574...	554.79093	370.19638	10
13	1386.726...	693.86682	462.91364	A	979.53199	490.26963	327.18218	9
14	1483.779...	742.39320	495.26456	P	908.49488	454.75108	303.50314	8
15	1596.863...	798.93523	532.95925	L	811.44211	406.22469	271.15222	7
16	1710.906...	855.95669	570.97355	N	698.35805	349.68266	233.45753	6
17	1807.958...	904.48308	603.32448	P	584.31512	292.66120	195.44322	5
18	1935.022...	968.01476	645.67893	K-Ben...	487.26236	244.13482	163.09230	4
19	2006.059...	1003.533...	669.35797	A	360.19899	180.60314	120.73785	3
20	2120.102...	1060.554...	707.37228	N	289.16188	145.08458	97.05881	2
21				R	175.11895	88.06311	59.04450	1

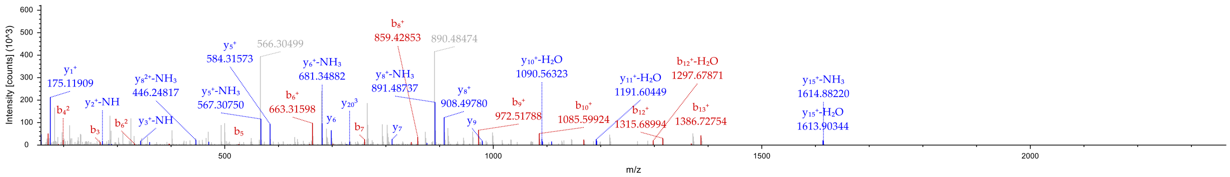

P62979 (Ubiquitin-ribosomal protein eS31 fusion protein)

Sequence: LIFAGKQLEDGR, K6-Ben_K2 (-1.03160 Da) (position: lysine 48)

#1	b⁺	b²⁺	Seq.	y⁺	y²⁺	#2
1	114.09134	57.54931	L			12
2	227.17540	114.09134	I	1232.627...	616.81714	11
3	374.24382	187.62555	F	1119.542...	560.27511	10
4	445.28093	223.14410	A	972.47453	486.74091	9
5	502.30240	251.65484	G	901.43742	451.22235	8
6	629.36576	315.18652	K-Ben...	844.41596	422.71162	7
7	757.42434	379.21581	Q	717.35259	359.17993	6
8	870.50840	435.75784	L	589.29402	295.15065	5
9	999.55099	500.27913	E	476.20995	238.60861	4
10	1114.577...	557.79261	D	347.16736	174.08732	3
11	1171.599...	586.30334	G	232.14042	116.57385	2
12			R	175.11895	88.06311	1

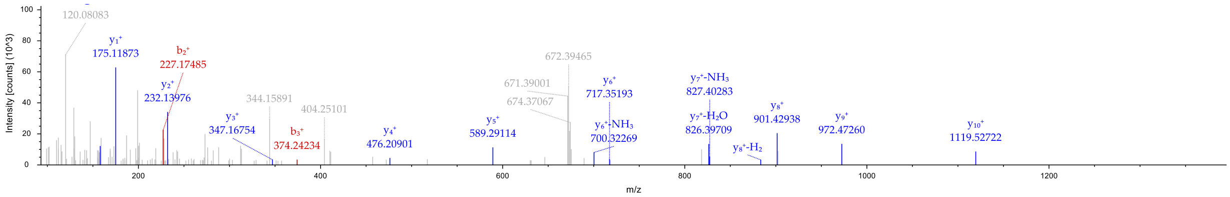

Q15149 (Plectin)

Sequence: LQLEETDHQKNLLDEELQR, K10-Ben_K2 (-1.03160 Da) (position: lysine 2339)

#1	b⁺	b²⁺	b³⁺	Seq.	y⁺	y²⁺	y³⁺	#2
1	114.09134	57.54931	38.70196	L				19
2	242.14992	121.57860	81.38816	Q	2237.068...	1119.037...	746.36088	18
3	355.23398	178.12063	119.08285	L	2109.009...	1055.008...	703.67468	17
4	484.27657	242.64193	162.09704	E	1995.925...	998.46636	665.98000	16
5	613.31917	307.16322	205.11124	E	1866.882...	933.94506	622.96580	15
6	714.36685	357.68706	238.79380	T	1737.840...	869.42376	579.95160	14
7	829.39379	415.20053	277.13611	D	1636.792...	818.89992	546.26904	13
8	966.45270	483.72999	322.82242	H	1521.765...	761.38645	507.92673	12
9	1094.511...	547.75928	365.50861	Q	1384.706...	692.85700	462.24042	11
10	1221.574...	611.29096	407.86306	K-Ben...	1256.648...	628.82771	419.55423	10
11	1335.617...	668.31242	445.87737	N	1129.584...	565.29603	377.19978	9
12	1448.701...	724.85445	483.57206	L	1015.541...	508.27456	339.18547	8
13	1561.785...	781.39649	521.26675	L	902.45779	451.73253	301.49078	7
14	1676.812...	838.90996	559.60906	D	789.37372	395.19050	263.79609	6
15	1805.855...	903.43125	602.62326	E	674.34678	337.67703	225.45378	5
16	1934.897...	967.95255	645.63746	E	545.30419	273.15573	182.43958	4
17	2047.981...	1024.494...	683.33215	L	416.26159	208.63444	139.42538	3
18	2176.040...	1088.523...	726.01834	Q	303.17753	152.09240	101.73069	2
19				R	175.11895	88.06311	59.04450	1

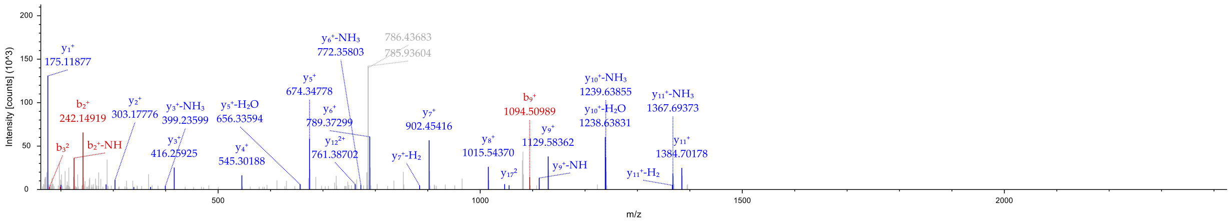

Q562R1 (Beta-actin-like protein 2)

Sequence: VAPDEHPILLTEAPLNPKINR, K18-Ben_K2 (-1.03160 Da) (position: lysine 114)

#1	b⁺	b²⁺	b³⁺	Seq.	y⁺	y²⁺	y³⁺	#2
1	100.07569	50.54148	34.03008	V				21
2	171.11280	86.06004	57.70912	A	2237.192...	1119.099...	746.40235	20
3	268.16557	134.58642	90.06004	P	2166.155...	1083.581...	722.72331	19
4	383.19251	192.09989	128.40235	D	2069.102...	1035.054...	690.37239	18
5	512.23510	256.62119	171.41655	E	1954.075...	977.54147	652.03007	17
6	649.29402	325.15065	217.10286	H	1825.033...	913.02018	609.01588	16
7	746.34678	373.67703	249.45378	P	1687.974...	844.49072	563.32957	15
8	859.43084	430.21906	287.14847	I	1590.921...	795.96434	530.97865	14
9	972.51491	486.76109	324.84315	L	1477.837...	739.42231	493.28396	13
10	1085.598...	543.30312	362.53784	L	1364.753...	682.88028	455.58928	12
11	1186.646...	593.82696	396.22040	T	1251.669...	626.33824	417.89459	11
12	1315.689...	658.34826	439.23460	E	1150.621...	575.81440	384.21203	10
13	1386.726...	693.86682	462.91364	A	1021.578...	511.29311	341.19783	9
14	1483.779...	742.39320	495.26456	P	950.54183	475.77455	317.51879	8
15	1596.863...	798.93523	532.95925	L	853.48906	427.24817	285.16787	7
16	1710.906...	855.95669	570.97355	N	740.40500	370.70614	247.47318	6
17	1807.958...	904.48308	603.32448	P	626.36207	313.68467	209.45887	5
18	1935.022...	968.01476	645.67893	K-Ben...	529.30931	265.15829	177.10795	4
19	2048.106...	1024.556...	683.37362	I	402.24594	201.62661	134.75350	3
20	2162.149...	1081.578...	721.38793	N	289.16188	145.08458	97.05881	2
21				R	175.11895	88.06311	59.04450	1

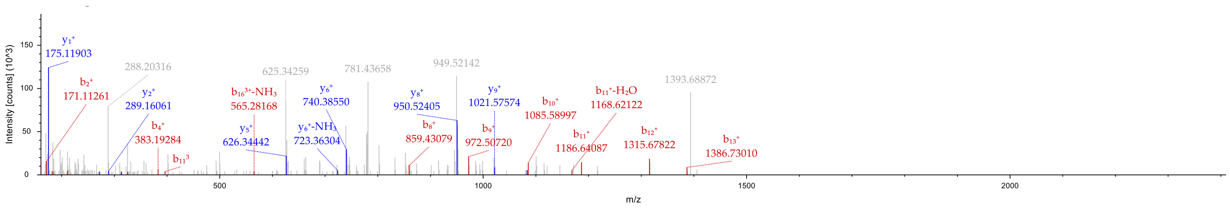

Q9BPW5 (Ras-like protein family member 11B)

Sequence: QALSAKVR, K6-Ben_K2 (-1.03160 Da) (position: lysine 241)

#1	b+	b2+	Seq.	y+	y2+	#2
1	129.06585	65.03657	Q			8
2	200.10297	100.55512	A	743.44105	372.22416	7
3	313.18703	157.09715	L	672.40394	336.70561	6
4	400.21906	200.61317	S	559.31987	280.16357	5
5	471.25617	236.13173	A	472.28784	236.64756	4
6	598.31954	299.66341	K-Ben...	401.25073	201.12900	3
7	697.38795	349.19761	V	274.18737	137.59732	2
8			R	175.11895	88.06311	1

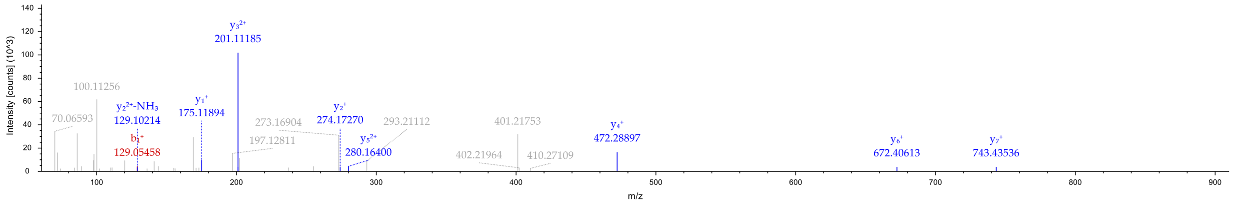

Q15233 (Non-POU domain-containing octamer-binding protein)

Sequence: QQEGFKGTFPDAR, K6-Ben_K2 (-1.03160 Da) (position: lysine 371)

#1	b+	b2+	Seq.	y+	y2+	#2
1	129.06585	65.03657	Q			13
2	257.12443	129.06585	Q	1351.627...	676.31751	12
3	386.16702	193.58715	E	1223.569...	612.28822	11
4	443.18849	222.09788	G	1094.526...	547.76692	10
5	590.25690	295.63209	F	1037.505...	519.25619	9
6	717.32027	359.16377	K-Ben...	890.43669	445.72198	8
7	774.34173	387.67450	G	763.37333	382.19030	7
8	875.38941	438.19834	T	706.35187	353.67957	6
9	1022.457...	511.73255	F	605.30419	303.15573	5
10	1119.510...	560.25893	P	458.23577	229.62152	4
11	1234.537...	617.77240	D	361.18301	181.09514	3
12	1305.574...	653.29096	A	246.15607	123.58167	2
13			R	175.11895	88.06311	1

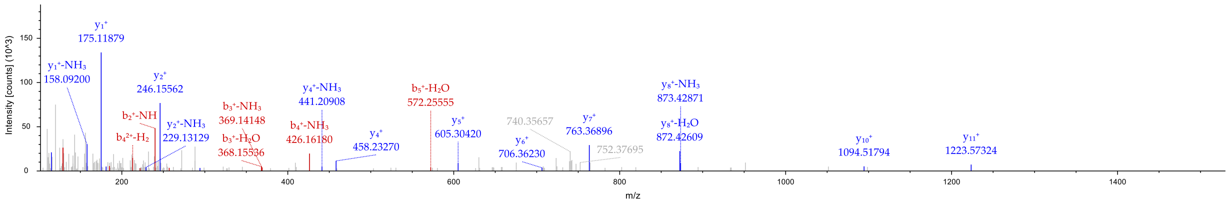

P04908 (Histone H2A type 1-B/E)

Sequence: NDEELNKLLGR, K7-Ben_K2 (-1.03160 Da) (position: lysine 96)

#1	b⁺	b²⁺	Seq.	y⁺	y²⁺	#2
1	115.05020	58.02874	N			11
2	230.07715	115.54221	D	1185.611...	593.30915	10
3	359.11974	180.06351	E	1070.584...	535.79568	9
4	488.16233	244.58480	E	941.54149	471.27438	8
5	601.24640	301.12684	L	812.49890	406.75309	7
6	715.28932	358.14830	N	699.41483	350.21106	6
7	842.35269	421.67998	K-Ben...	585.37191	293.18959	5
8	955.43675	478.22201	L	458.30854	229.65791	4
9	1068.520...	534.76405	L	345.22448	173.11588	3
10	1125.542...	563.27478	G	232.14042	116.57385	2
11			R	175.11895	88.06311	1

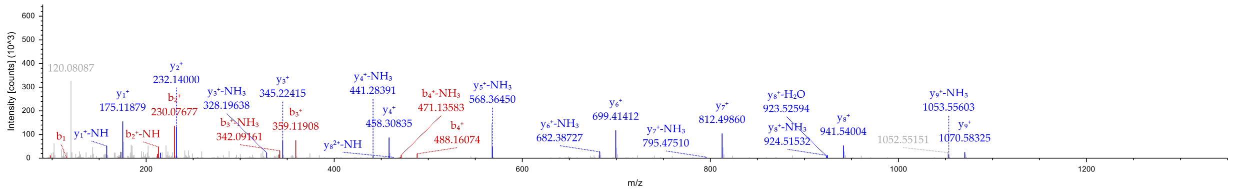

Representative peptide spectra of modified peptides (propargyl sites, +38 on lysine) -*<u>TACO modified peptides: All these peptides are identified from nuclear extract of LnCap without treatment with TACO chemistry.</u>*

Q6ZMV9 (Kinesin-like protein KIF6)

Sequence: LSSAPSQAQDFSILGK, K16-Ben_K1 (38.01570 Da) (position: lysine 538)

#1	b⁺	b²⁺	Seq.	y⁺	y²⁺	#2
1	114.09134	57.54931	L			16
2	201.12337	101.06532	S	1573.785...	787.39649	15
3	288.15540	144.58134	S	1486.753...	743.88048	14
4	359.19251	180.09989	A	1399.721...	700.36447	13
5	456.24527	228.62628	P	1328.684...	664.84591	12
6	543.27730	272.14229	S	1231.631...	616.31953	11
7	671.33588	336.17158	Q	1144.599...	572.80351	10
8	742.37299	371.69014	A	1016.541...	508.77422	9
9	870.43157	435.71942	Q	945.50406	473.25567	8
10	985.45851	493.23290	D	817.44548	409.22638	7
11	1132.526...	566.76710	F	702.41854	351.71291	6
12	1219.558...	610.28312	S	555.35012	278.17870	5
13	1332.643...	666.82515	I	468.31810	234.66269	4
14	1445.727...	723.36718	L	355.23403	178.12065	3
15	1502.748...	751.87791	G	242.14997	121.57862	2
16			K-Ben...	185.12850	93.06789	1

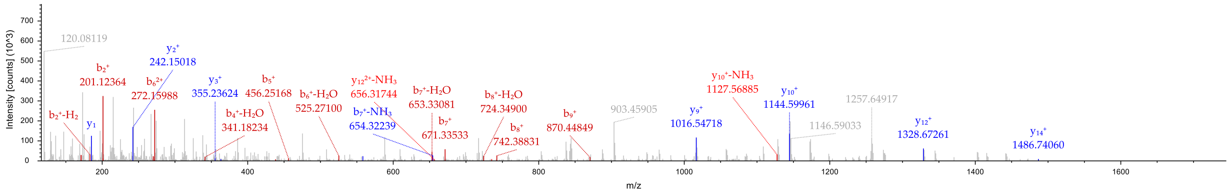

Q12955 (Ankyrin-3)

Sequence: TLPVYVSFVQVGK, K13-Ben_K1 (38.01570 Da) (position: lysine 3102)

#1	b+	b2+	Seq.	y+	y2+	#2
1	102.05496	51.53112	T			13
2	215.13902	108.07315	L	1373.782...	687.39504	12
3	312.19178	156.59953	P	1260.698...	630.85301	11
4	411.26020	206.13374	V	1163.645...	582.32662	10
5	574.32353	287.66540	Y	1064.577...	532.79242	9
6	673.39194	337.19961	V	901.51423	451.26075	8
7	760.42397	380.71562	S	802.44582	401.72655	7
8	907.49238	454.24983	F	715.41379	358.21053	6
9	1006.560...	503.78404	V	568.34537	284.67632	5
10	1134.619...	567.81332	Q	469.27696	235.14212	4
11	1233.687...	617.34753	V	341.21838	171.11283	3
12	1290.709...	645.85826	G	242.14997	121.57862	2
13			K-Ben...	185.12850	93.06789	1

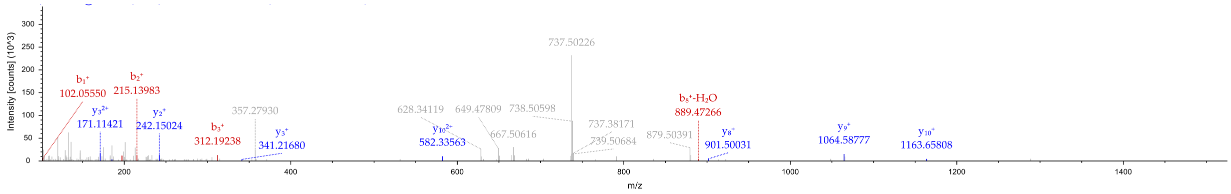

P50454 (Serpin H1)

Sequence: SAGLAFSLYQAMAK, K14-Ben_K1 (38.01570 Da) (position: lysine 60)

#1	b+	b2+	Seq.	y+	y2+	#2
1	88.03930	44.52329	S			14
2	159.07642	80.04185	A	1408.729...	704.86833	13
3	216.09788	108.55258	G	1337.692...	669.34977	12
4	329.18195	165.09461	L	1280.670...	640.83904	11
5	400.21906	200.61317	A	1167.586...	584.29701	10
6	547.28747	274.14738	F	1096.549...	548.77845	9
7	634.31950	317.66339	S	949.48121	475.24425	8
8	747.40357	374.20542	L	862.44919	431.72823	7
9	910.46689	455.73709	Y	749.36512	375.18620	6
10	1038.525...	519.76637	Q	586.30179	293.65454	5
11	1109.562...	555.28493	A	458.24322	229.62525	4
12	1240.603...	620.80517	M	387.20610	194.10669	3
13	1311.640...	656.32373	A	256.16562	128.58645	2
14			K-Ben...	185.12850	93.06789	1

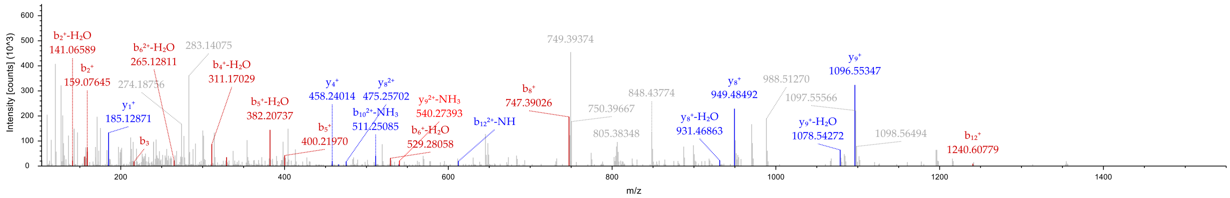

Representative peptide spectra of modified peptides (aldehydes on lysine, -1 on lysine) -*__TACO modified peptides: All these peptides are identified from nuclear extract of LnCap without treatment with TACO chemistry.__*

Q9NYQ6 (Cadherin EGF LAG seven-pass G-type receptor 1)

Sequence: SVVVGGASEDKVSVR, K11-Ben_K2 (-1.03160 Da) (position: lysine 1830)

#1	b⁺	b²⁺	Seq.	y⁺	y²⁺	#2
1	88.03930	44.52329	S			15
2	187.10772	94.05750	V	1400.738...	700.87265	14
3	286.17613	143.59170	V	1301.669...	651.33844	13
4	385.24455	193.12591	V	1202.601...	601.80423	12
5	442.26601	221.63664	G	1103.532...	552.27003	11
6	499.28747	250.14738	G	1046.511...	523.75929	10
7	570.32459	285.66593	A	989.48985	495.24856	9
8	657.35662	329.18195	S	918.45274	459.73001	8
9	786.39921	393.70324	E	831.42071	416.21399	7
10	901.42615	451.21671	D	702.37811	351.69270	6
11	1028.489...	514.74840	K-Ben...	587.35117	294.17922	5
12	1127.557...	564.28260	V	460.28781	230.64754	4
13	1214.589...	607.79862	S	361.21939	181.11334	3
14	1313.658...	657.33282	V	274.18737	137.59732	2
15			R	175.11895	88.06311	1

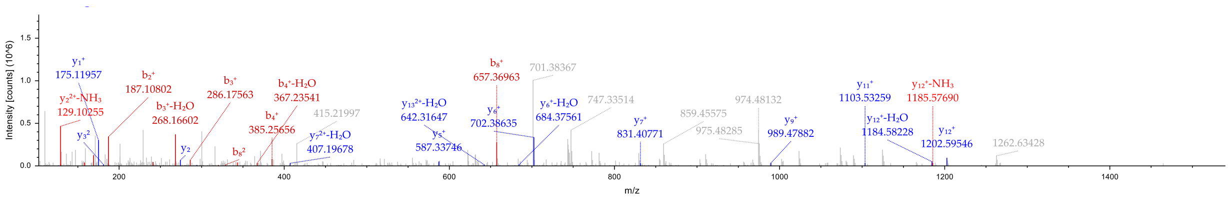

Q9C0D3 (Protein zyg-11 homolog B)

Sequence: TKPEILK, K2-Ben_K2 (-1.03160 Da) (position: lysine 360)

1	102.05496	T		7
2	229.11832	K-Ben...	726.43965	6
3	326.17108	P	599.37629	5
4	455.21367	E	502.32353	4
5	568.29774	I	373.28093	3
6	681.38180	L	260.19687	2
7		K	147.11280	1

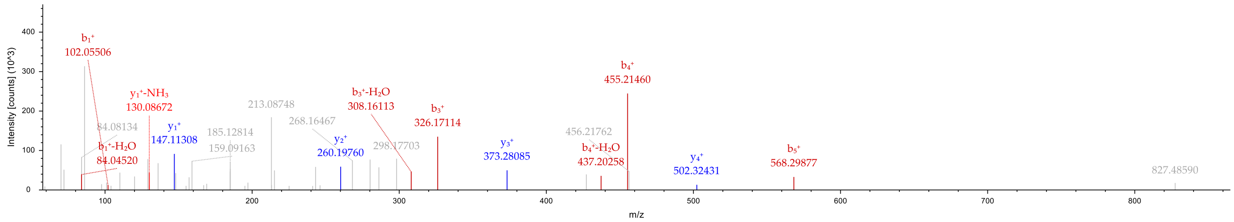

List of MCF-10A Kme$_2$ modification sites and proteins

Representative peptide spectra of modified peptides (propargyl and aldehydes on lysine, +38, -1 on lysine). *All these peptides are identified from cell lysate of MCF-10A after treatment with TACO chemistry.*

Q13162 (Peroxiredoxin-4)

Sequence: VCPAGWkPGSETI, K10-Ben_K2 (-1.03160 Da) (position: lysine 250)

#1	b+	b2+	b3+	b4+	Seq.	y+	y2+	y3+	y4+	#2
1	138.06619	69.53673	46.69358	35.27200	H					23
2	195.08765	98.04746	65.70074	49.52737	G	2265.085...	1133.046...	755.70007	567.02687	22
3	324.13025	162.56876	108.71493	81.78802	E	2208.064...	1104.535...	736.69291	552.77150	21
4	423.19866	212.10297	141.73774	106.55512	V	2079.021...	1040.014...	693.67871	520.51085	20
5	583.22931	292.11829	195.08129	146.56278	C-Car...	1979.953...	990.48022	660.65591	495.74375	19
6	680.28207	340.64467	227.43221	170.82598	P	1819.922...	910.46490	607.31236	455.73609	18
7	751.31919	376.16323	251.11125	188.58525	A	1722.869...	861.93852	574.96144	431.47290	17
8	808.34065	404.67396	270.11840	202.84062	G	1651.832...	826.41996	551.28240	413.71362	16
9	994.41996	497.71362	332.14484	249.36045	W	1594.811...	797.90923	532.27525	399.45825	15
10	1121.483...	561.24530	374.49929	281.12629	K-Ben...	1408.731...	704.86957	470.24881	352.93843	14
11	1218.536...	609.77168	406.85021	305.38948	P	1281.668...	641.33789	427.89435	321.17258	13
12	1275.557...	638.28241	425.85737	319.64485	G	1184.615...	592.81151	395.54343	296.90939	12
13	1362.589...	681.79843	454.86804	341.40285	S	1127.594...	564.30078	376.53628	282.65403	11
14	1491.632...	746.31973	497.88224	373.66350	E	1040.562...	520.78476	347.52560	260.89602	10
15	1592.679...	796.84356	531.56480	398.92542	T	911.51966	456.26347	304.51140	228.63537	9
16	1705.763...	853.38560	569.25949	427.19644	I	810.47198	405.73963	270.82884	203.37345	8
17	1818.847...	909.92763	606.95418	455.46745	I	697.38792	349.19760	233.13416	175.10244	7
18	1915.900...	958.45401	639.30510	479.73064	P	584.30385	292.65556	195.43947	146.83142	6
19	2030.927...	1015.967...	677.64741	508.48738	D	487.25109	244.12918	163.08855	122.56823	5
20	2127.980...	1064.493...	709.99833	532.75057	P	372.22415	186.61571	124.74623	93.81149	4
21	2199.017...	1100.012...	733.67737	550.50985	A	275.17138	138.08933	92.39531	69.54830	3
22	2256.039...	1128.523...	752.68453	564.76521	G	204.13427	102.57077	68.71627	51.78902	2
23					K	147.11280	74.06004	49.70912	37.53366	1

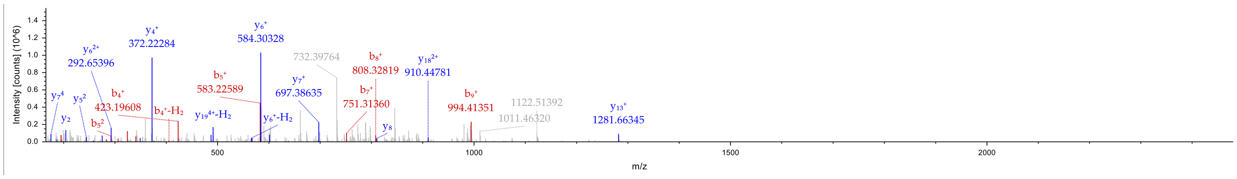

P05556 (Integrin beta-1)

Sequence: GTAEKLkPEDITQ, K2-Ben_K2 (-1.03160 Da) (position: lysine 107)

#1	b+	b2+	b3+	Seq.	y+	y2+	y3+	#2
1	114.09134	57.54931	38.70196	L				17
2	241.15470	121.08099	81.05642	K-Ben...	1905.044...	953.02566	635.68620	16
3	338.20747	169.60737	113.40734	P	1777.980...	889.49398	593.33174	15
4	467.25006	234.12867	156.42154	E	1680.927...	840.96759	560.98082	14
5	582.27700	291.64214	194.76385	D	1551.885...	776.44630	517.96662	13
6	695.36107	348.18417	232.45854	I	1436.858...	718.93283	479.62431	12
7	796.40875	398.70801	266.14110	T	1323.774...	662.39079	441.92962	11
8	924.46732	462.73730	308.82729	Q	1222.726...	611.86695	408.24706	10
9	1037.551...	519.27933	346.52198	I	1094.668...	547.83767	365.56087	9
10	1165.609...	583.30862	389.20817	Q	981.58399	491.29563	327.86618	8
11	1262.662...	631.83500	421.55909	P	853.52541	427.26634	285.17999	7
12	1390.721...	695.86429	464.24529	Q	756.47265	378.73996	252.82907	6
13	1518.779...	759.89358	506.93148	Q	628.41407	314.71067	210.14287	5
14	1631.863...	816.43561	544.62617	L	500.35549	250.68139	167.45668	4
15	1730.932...	865.96982	577.64897	V	387.27143	194.13935	129.76199	3
16	1844.016...	922.51185	615.34366	L	288.20302	144.60515	96.73919	2
17				R	175.11895	88.06311	59.04450	1

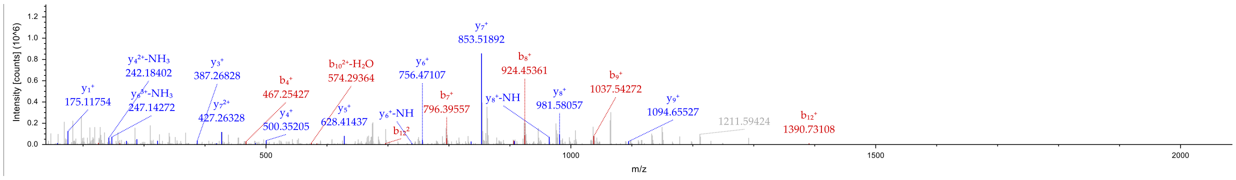

Q9Y2A7 (Nck-associated protein 1)

Sequence: MRTSFDkPDQMAA, K5-Ben_K2 (-1.03160 Da) (position: lysine 886)

#1	b+	b2+	b3+	Seq.	y+	y2+	y3+	#2
1	102.05496	51.53112	34.68984	T				14
2	189.08698	95.04713	63.70051	S	1496.709...	748.85815	499.57453	13
3	336.15540	168.58134	112.72332	F	1409.677...	705.34214	470.56385	12
4	451.18234	226.09481	151.06563	D	1262.608...	631.80793	421.54105	11
5	578.24570	289.62649	193.42009	K-Ben...	1147.581...	574.29446	383.19873	10
6	675.29847	338.15287	225.77101	P	1020.518...	510.76278	340.84428	9
7	790.32541	395.66634	264.11332	D	923.46551	462.23640	308.49336	8
8	918.38399	459.69563	306.79951	Q	808.43857	404.72292	270.15104	7
9	1049.424...	525.21587	350.47968	M	680.37999	340.69364	227.46485	6
10	1120.461...	560.73443	374.15871	A	549.33951	275.17339	183.78469	5
11	1191.498...	596.25299	397.83775	A	478.30240	239.65484	160.10565	4
12	1304.582...	652.79502	435.53244	L	407.26528	204.13628	136.42661	3
13	1451.651...	726.32923	484.55524	F	294.18122	147.59425	98.73192	2
14				K	147.11280	74.06004	49.70912	1

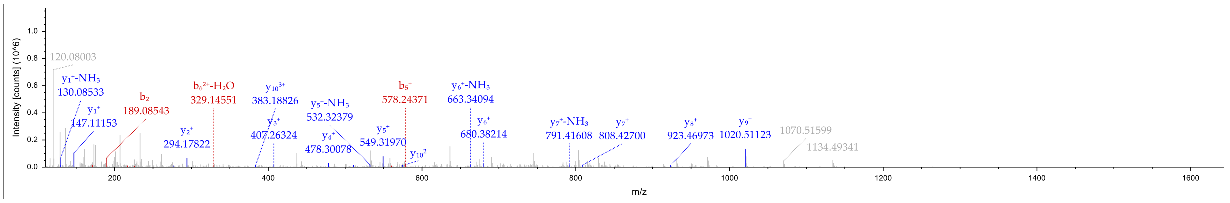

Q562R1 (Beta-actin-like protein 2)

Sequence: IADRMQkEIITLA, K3-Ben_K2 (-1.03160 Da) (position: lysine 316)

#1	b+	b2+	Seq.	y+	y2+	#2
1	148.04268	74.52498	M-Oxi...			14
2	276.10125	138.55427	Q	1458.787...	729.89728	13
3	403.16462	202.08595	K-Ben...	1330.728...	665.86799	12
4	532.20721	266.60724	E	1203.665...	602.33631	11
5	645.29127	323.14928	I	1074.622...	537.81501	10
6	758.37534	379.69131	I	961.53868	481.27298	9
7	859.42302	430.21515	T	848.45462	424.73095	8
8	972.50708	486.75718	L	747.40694	374.20711	7
9	1043.544...	522.27574	A	634.32287	317.66507	6
10	1140.596...	570.80212	P	563.28576	282.14652	5
11	1227.628...	614.31813	S	466.23300	233.62014	4
12	1328.676...	664.84197	T	379.20097	190.10412	3
13	1459.717...	730.36221	M	278.15329	139.58028	2
14			K	147.11280	74.06004	1

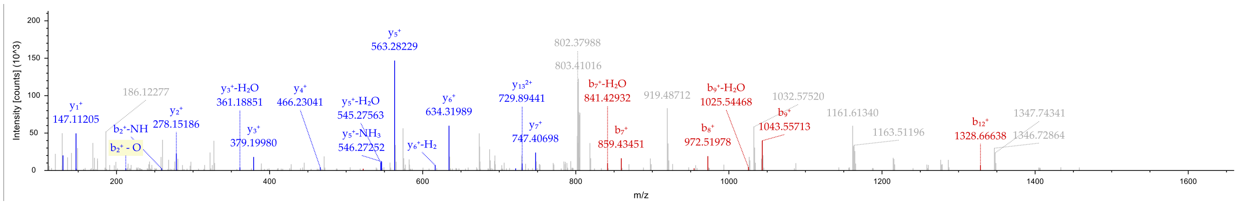

P17540 (Creatine kinase S-type, mitochondrial)

Sequence: KDPRFSkILENLR, K3-Ben_K2 (-1.03160 Da) (position: lysine 344)

#1	b+	b2+	Seq.	y+	y2+	#2
1	148.07569	74.54148	F			9
2	235.10772	118.05750	S	971.55206	486.27967	8
3	362.17108	181.58918	K-Ben...	884.52003	442.76365	7
4	475.25515	238.13121	I	757.45666	379.23197	6
5	588.33921	294.67324	L	644.37260	322.68994	5
6	717.38180	359.19454	E	531.28854	266.14791	4
7	831.42473	416.21600	N	402.24594	201.62661	3
8	944.50879	472.75804	L	288.20302	144.60515	2
9			R	175.11895	88.06311	1

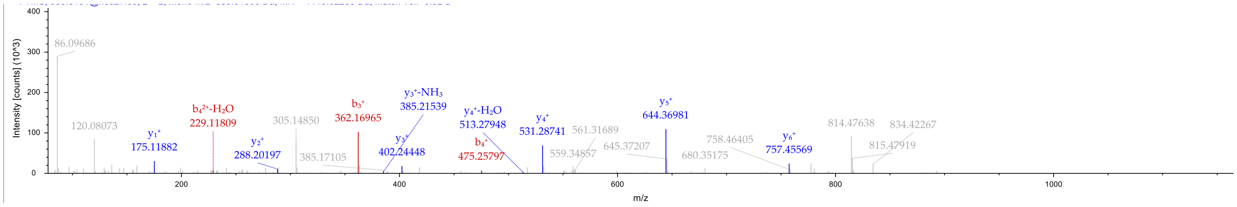

Q15154 (Pericentriolar material 1 protein)

Sequence: QLSENRkPFNFLP, K1-Ben_K2 (-1.03160 Da) (position: lysine 139)

#1	b⁺	b²⁺	Seq.	y⁺	y²⁺	#2
1	128.07064	64.53896	K-Ben...			14
2	225.12340	113.06534	P	1579.793...	790.40050	13
3	372.19182	186.59955	F	1482.740...	741.87412	12
4	486.23474	243.62101	N	1335.672...	668.33991	11
5	633.30316	317.15522	F	1221.629...	611.31845	10
6	746.38722	373.69725	L	1074.561...	537.78424	9
7	843.43999	422.22363	P	961.47714	481.24221	8
8	990.47539	495.74133	M-Oxi...	864.42438	432.71583	7
9	1118.533...	559.77062	Q	717.38898	359.19813	6
10	1231.618...	616.31265	I	589.33040	295.16884	5
11	1345.660...	673.33412	N	476.24634	238.62681	4
12	1446.708...	723.85795	T	362.20341	181.60534	3
13	1560.751...	780.87942	N	261.15573	131.08150	2
14			K	147.11280	74.06004	1

Q15276 (Rab GTPase-binding effector protein 1)

Sequence: TRDQVKkLQLMLR, K1-Ben_K2 (-1.03160 Da) (position: lysine 559)

#1	b⁺	b²⁺	Seq.	y⁺	y²⁺	#2
1	128.07064	64.53896	K-Ben...			7
2	241.15470	121.08099	L	773.47021	387.23874	6
3	369.21328	185.11028	Q	660.38614	330.69671	5
4	482.29734	241.65231	L	532.32756	266.66742	4
5	613.33783	307.17255	M	419.24350	210.12539	3
6	726.42189	363.71459	L	288.20302	144.60515	2
7			R	175.11895	88.06311	1

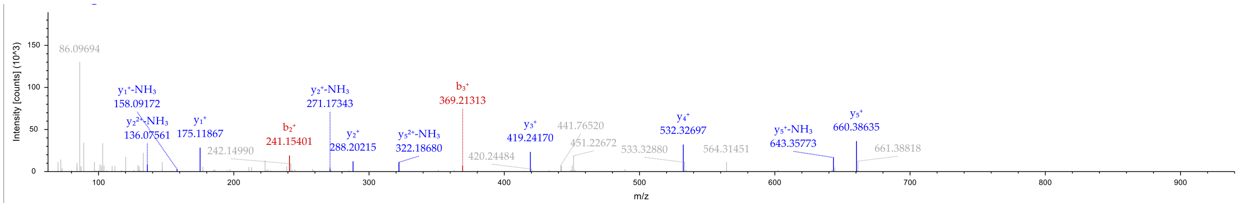

P68032 (Actin, alpha cardiac muscle 1)

Sequence: LCYVALDFENEMATAASSSSLEK, K23-Ben_K2 (-1.03160 Da) (position: lysine 240)

#1	b+	b2+	Seq.	y+	y2+	#2
1	114.09134	57.54931	L			23
2	274.12199	137.56463	C-Car...	2438.037...	1219.522...	22
3	437.18532	219.09630	Y	2278.006...	1139.507...	21
4	536.25373	268.63050	V	2114.943...	1057.975...	20
5	607.29085	304.14906	A	2015.875...	1008.441...	19
6	720.37491	360.69109	L	1944.837...	972.92260	18
7	835.40185	418.20456	D	1831.753...	916.38057	17
8	982.47027	491.73877	F	1716.726...	858.86710	16
9	1111.512...	556.26007	E	1569.658...	785.33289	15
10	1225.555...	613.28153	N	1440.615...	720.81160	14
11	1354.598...	677.80283	E	1326.572...	663.79013	13
12	1501.633...	751.32053	M-Oxi...	1197.530...	599.26884	12
13	1572.670...	786.83908	A	1050.494...	525.75114	11
14	1673.718...	837.36292	T	979.45788	490.23258	10
15	1744.755...	872.88148	A	878.41020	439.70874	9
16	1815.792...	908.40004	A	807.37309	404.19018	8
17	1902.824...	951.91605	S	736.33597	368.67163	7
18	1989.856...	995.43207	S	649.30395	325.15561	6
19	2076.888...	1038.948...	S	562.27192	281.63960	5
20	2163.920...	1082.464...	S	475.23989	238.12358	4
21	2277.004...	1139.006...	L	388.20786	194.60757	3
22	2406.047...	1203.527...	E	275.12380	138.06554	2
23			K-Ben...	146.08120	73.54424	1

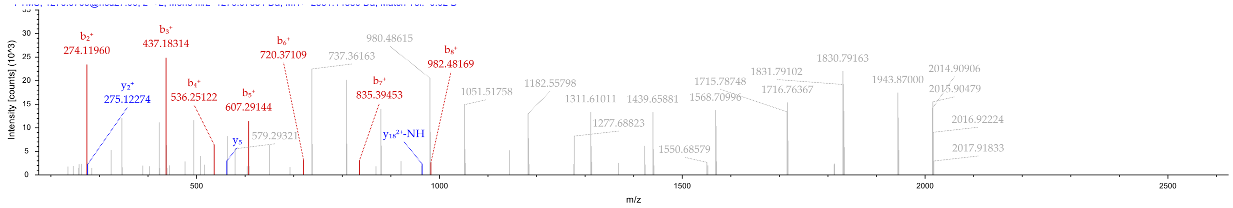

Q9H165 (B-cell lymphoma/leukemia 11A)

Sequence: LSAKGATDAGAKPPR, K4-Ben_K2 (+38.0157 Da) (position: lysine 559)

#1	b+	b2+	b3+	Seq.	y+	y2+	y3+	#2
1	114.09134	57.54931	38.70196	L				15
2	201.12337	101.06532	67.71264	S	1325.680...	663.34406	442.56513	14
3	272.16048	136.58388	91.39168	A	1238.648...	619.82804	413.55445	13
4	399.22385	200.11556	133.74613	K-Ben...	1167.611...	584.30949	389.87542	12
5	456.24531	228.62629	152.75329	G	1040.548...	520.77780	347.52096	11
6	527.28242	264.14485	176.43233	A	983.52687	492.26707	328.51381	10
7	628.33010	314.66869	210.11488	T	912.48976	456.74852	304.83477	9
8	743.35704	372.18216	248.45720	D	811.44208	406.22468	271.15221	8
9	814.39416	407.70072	272.13624	A	696.41513	348.71121	232.80990	7
10	871.41562	436.21145	291.14339	G	625.37802	313.19265	209.13086	6
11	942.45274	471.73001	314.82243	A	568.35656	284.68192	190.12370	5
12	1070.547...	535.77749	357.52075	K	497.31944	249.16336	166.44467	4
13	1167.600...	584.30387	389.87167	P	369.22448	185.11588	123.74634	3
14	1264.653...	632.83025	422.22259	P	272.17172	136.58950	91.39542	2
15				R	175.11895	88.06311	59.04450	1

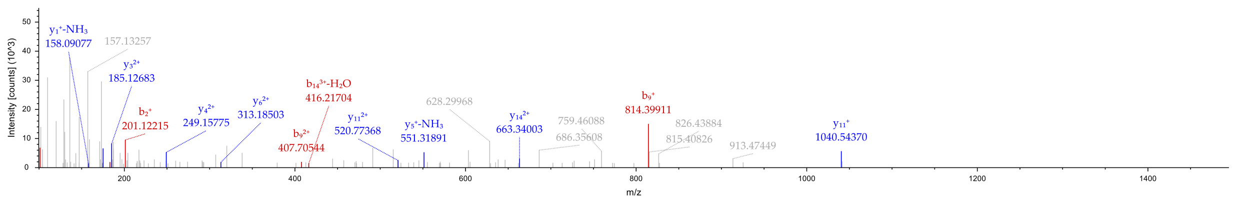

XXIX. Supplementary Figure 23. TACO modification of nuclear extract, biotin enrichment of modified Kme_2 proteins from nuclear extract and identification of Kme_2 proteins and Kme_2 sites by proteomic analysis.

TACO modification and enrichment of Kme_2 proteins from nuclear extract: To 2 mg of nuclear extract in 2 mL of NaP buffer pH 7 was added 20 mg of selectfluor and 80 µL of pyridine. The reaction was stirred at room temperature for 1 h. Samples were subjected to acetone precipitation to remove small molecules. To proteins resuspended in 2 mL of NaP buffer pH 5.0 was added a solution of 0.5 mg of Biotin-dPEG®3-oxyamine hydrochloride in DMSO and stirred for 6 h, followed by acetone precipitation. TACO modified nuclear lysate was resuspended in 2 mL binding buffer (Tris-buffered saline, pH 7.5). 100 µL of prepped magnetic streptavidin beads were added to biotinylated nuclear lysate and incubated at 4 ^{O}C for 1 h. Beads were washed 3x with washing buffer (TBS, 2M urea, pH 7.5) and finally with PBS pH 7. After washing, captured proteins were eluted by adding 1X SDS sample buffer (200 uL) and heated at 98 ^{O}C for 10 m. Eluted proteins were passed through a 3 kDa MW-cut off filter, followed by digestion with Thermo scientific EasyPep Mini MS sample Prep Kit.

Proteomics analysis of enriched proteins: Proteins were digested using Thermo scientific EasyPep Mini MS sample Prep Kit. Digested peptides were analyzed by LC/MS. Samples were analyzed by LC-MS using a Q-Exactive Plus orbitrap mass spectrometer equipped with Dionex UltiMate 3000 LC system (Thermo). Briefly, lyophilized digested peptides were resuspended in 0.1% formic acid in 10% acetonitrile and loaded onto a trap column (PepMap™ NEO 5 µm C18 300 µm X 5 mm Trap Cartridge) and resolved through a custom analytical column packed with ReproSil-Pur 120 C18-AQ 3 µm beads (Dr. Maisch GmbH) at a flow rate of 0.3 µL/min with a gradient solvent A (0.1% formic acid in 2% acetonitrile) and a gradient solvent B (0.1% formic acid in 80% acetonitrile) for 150 minutes. MS analysis was conducted in a data-dependent manner with full scans in the range from 400 to 1800 m/z using an Orbitrap mass analyzer set as follows: MS1: resolution = 70,000, AGC target = 3e6, Max IT = 100ms; MS2: resolution = 17,500, AGC target 1e5, Max IT = 50ms. The top fifteen most intense precursor ions were selected for MS2 with an isolation window of 4 m/z. Isolated precursors were fragmented by high energy collisional dissociation (HCD) with normalized collision energy (NCE) of 27. LC-MS RAW files were searched against the human proteome sequence database from UniProt using the Sequest HT search engine embedded in Proteome Discoverer 3.0 (Thermo) with 10 ppm MS1 precursor mass tolerance, 0.02 Da MS2 fragment mass tolerance, 0.01 false discovery rate. Search included the following modifications: Methionine oxidation (+15.99492 Da), asaparagine and glutamine deamidation (+0.98402 Da) and protein N-terminal acetylation (+42.03670), histidine fluorination (+17.9906), tryptophan fluorination (+33.9855), and lysine biotin-oxime (+415.1777) were variable modifications (up to 3 allowed per peptide); cysteine was assigned a fixed carbamidomethyl modification (+57.021465 Da).

Representative peptide spectra of modified peptides (Biotin-oxime sites, +415 on lysine)
All these peptides are identified from nuclear extract of LnCap after treatment with TACO chemistry.

Q6R327 (Rapamycin-insensitive companion of mTOR)
Sequence: LSSESKTSNR, K6-BenKPlus415 (415.17770 Da) (position: lysine 1125)

#1	b⁺	b²⁺	Seq.	y⁺	y²⁺	#2
1	114.09134	57.54931	L			10
2	201.12337	101.06532	S	1410.652...	705.83010	9
3	288.15540	144.58134	S	1323.620...	662.31409	8
4	417.19799	209.10263	E	1236.588...	618.79807	7
5	504.23002	252.61865	S	1107.546...	554.27678	6
6	1047.502...	524.25498	K-Ben...	1020.514...	510.76076	5
7	1148.550...	574.77882	T	477.24159	239.12443	4
8	1235.582...	618.29483	S	376.19391	188.60059	3
9	1349.625...	675.31630	N	289.16188	145.08458	2
10			R	175.11895	88.06311	1

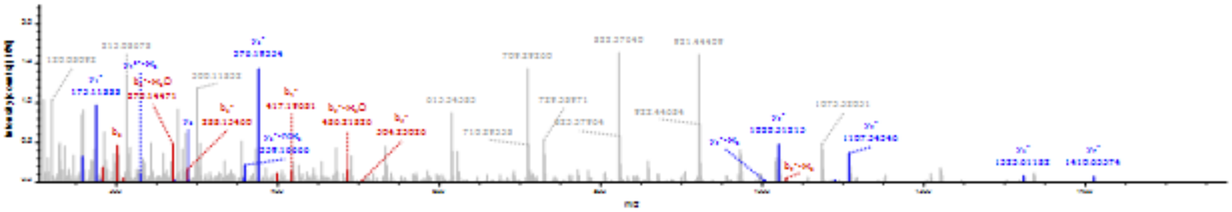

P17098 (Zinc finger protein 8)
Sequence: LIFEQTPALTK, K11-BenKPlus415 (415.17770 Da) (position: lysine 441)

#1	b⁺	b²⁺	Seq.	y⁺	y²⁺	#2
1	114.09134	57.54931	L			11
2	227.17540	114.09134	I	1562.813...	781.91036	10
3	374.24382	187.62555	F	1449.729...	725.36833	9
4	503.28641	252.14684	E	1302.660...	651.83412	8
5	631.34499	316.17613	Q	1173.618...	587.31283	7
6	732.39267	366.69997	T	1045.559...	523.28354	6
7	829.44543	415.22635	P	944.51212	472.75970	5
8	900.48254	450.74491	A	847.45936	424.23332	4
9	1013.566...	507.28694	L	776.42225	388.71476	3
10	1114.614...	557.81078	T	663.33818	332.17273	2
11			K-Ben...	562.29050	281.64889	1

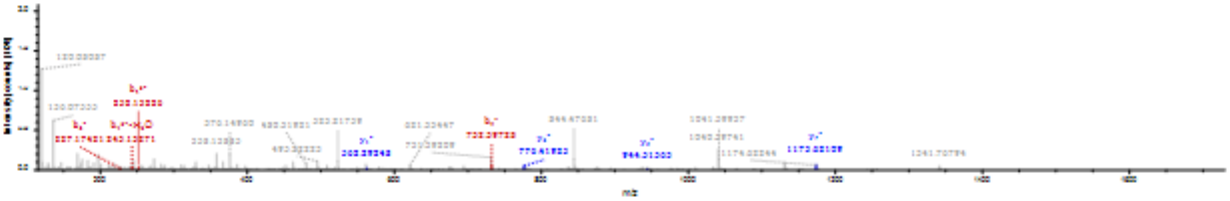

Q9H270 (Vacuolar protein sorting-associated protein 11 homolog)
Sequence: TIGKLEPSYVIR, K4-BenKPlus415 (415.17770 Da) (position: lysine 417)

#1	b+	b2+	Seq.	y+	y2+	#2
1	102.05496	51.53112	T			12
2	215.13902	108.07315	I	1689.924...	845.46584	11
3	272.16048	136.58388	G	1576.840...	788.92381	10
4	815.43315	408.22021	K-Ben...	1519.818...	760.41307	9
5	928.51721	464.76224	L	976.54621	488.77674	8
6	1057.559...	529.28354	E	863.46214	432.23471	7
7	1154.612...	577.80992	P	734.41955	367.71341	6
8	1241.644...	621.32594	S	637.36679	319.18703	5
9	1404.707...	702.85760	Y	550.33476	275.67102	4
10	1503.776...	752.39181	V	387.27143	194.13935	3
11	1616.860...	808.93384	I	288.20302	144.60515	2
12			R	175.11895	88.06311	1

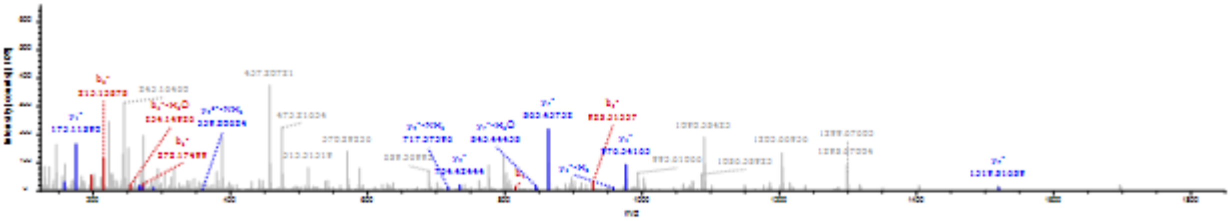

Q99741 (Cell division control protein 6 homolog)
Sequence: KAGSLYLSGAPGTGK, K15-BenKPlus415 (415.17770 Da) (position: lysine 208)

#1	b+	b2+	b3+	Seq.	y+	y2+	y3+	#2
1	129.10224	65.05476	43.70560	K				15
2	200.13935	100.57331	67.38464	A	1693.846...	847.42691	565.28703	14
3	257.16082	129.08405	86.39179	G	1622.809...	811.90835	541.60799	13
4	344.19285	172.60006	115.40247	S	1565.787...	783.39762	522.60084	12
5	457.27691	229.14209	153.09715	L	1478.755...	739.88161	493.59016	11
6	620.34024	310.67376	207.45160	Y	1365.671...	683.33957	455.89548	10
7	733.42430	367.21579	245.14628	L	1202.608...	601.80791	401.54103	9
8	820.45633	410.73180	274.15696	S	1089.524...	545.26588	363.84634	8
9	877.47779	439.24254	293.16412	G	1002.492...	501.74986	334.83567	7
10	948.51491	474.76109	316.84315	A	945.47099	473.23913	315.82851	6
11	1045.567...	523.28747	349.19407	P	874.43387	437.72058	292.14948	5
12	1102.589...	551.79821	368.20123	G	777.38111	389.19419	259.79855	4
13	1203.636...	602.32205	401.88379	T	720.35965	360.68346	240.79140	3
14	1260.658...	630.83278	420.89094	G	619.31197	310.15962	207.10884	2
15				K-Ben...	562.29050	281.64889	188.10169	1

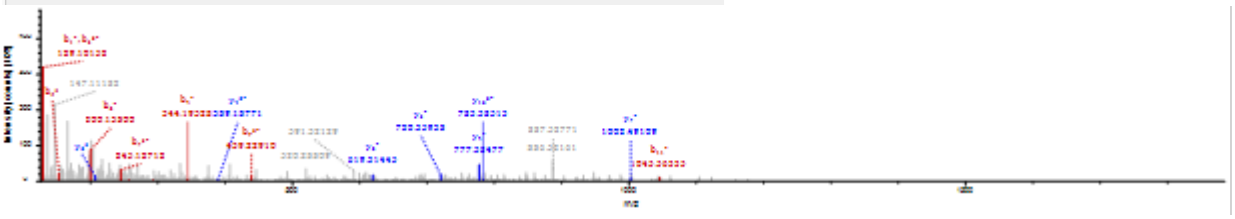

Q6ZSZ5 (Rho guanine nucleotide exchange factor 18)
Sequence: KFQNLIK, K1-BenKPlus415 (415.17770 Da) (position: lysine 573)

#1	b⁺	Seq.	y⁺	#2
1	544.27994	K-Ben...		7
2	691.34835	F	762.45085	6
3	819.40693	Q	615.38244	5
4	933.44986	N	487.32386	4
5	1046.533...	L	373.28093	3
6	1159.617...	I	260.19687	2
7		K	147.11280	1

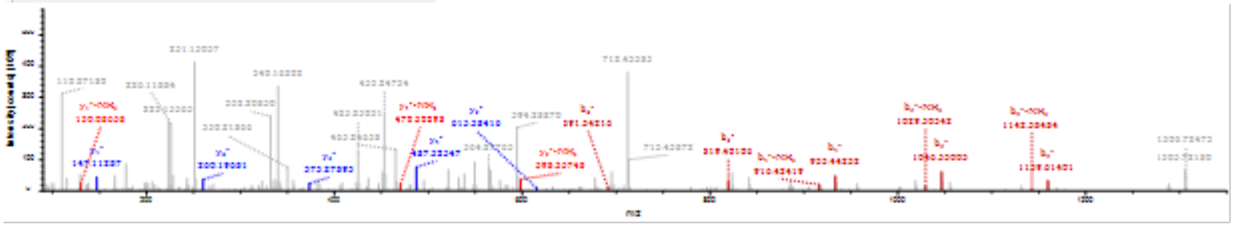

P62979 (Ubiquitin-ribosomal protein eS31 fusion protein)
Sequence: MQIFVKTLTGK, M1-Oxidation (15.99492 Da), K6-BenKPlus415 (415.17770 Da) (position: lysine 6)

#1	b⁺	b²⁺	Seq.	y⁺	y²⁺	#2
1	148.04268	74.52498	M-Oxi...			11
2	276.10125	138.55427	Q	1549.865...	775.43655	10
3	389.18532	195.09630	I	1421.807...	711.40726	9
4	536.25373	268.63050	F	1308.723...	654.86523	8
5	635.32215	318.16471	V	1161.654...	581.33102	7
6	1178.594...	589.80104	K-Ben...	1062.586...	531.79681	6
7	1279.642...	640.32488	T	519.31369	260.16048	5
8	1392.726...	696.86691	L	418.26601	209.63664	4
9	1493.774...	747.39075	T	305.18195	153.09461	3
10	1550.795...	775.90148	G	204.13427	102.57077	2
11			K	147.11280	74.06004	1

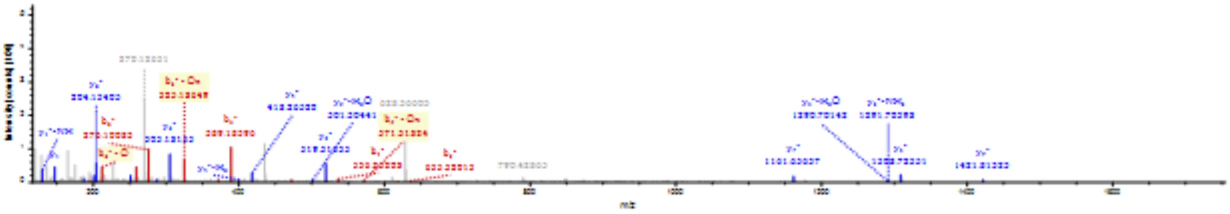

Q99832 (T-complex protein 1 subunit)
Sequence: AFFAKMVVDAVMMLDDLLQLK, M12-Oxidation (15.99492 Da), M13-Oxidation (15.99492 Da), K5-BenKPlus415 (415.17770 Da) (position: lysine 177)

#1	b+	b2+	b3+	Seq.	y+	y2+	y3+	#2
1	72.04439	36.52583	24.68631	A				21
2	219.11280	110.06004	73.70912	F	2774.388...	1387.697...	925.46775	20
3	366.18122	183.59425	122.73192	F	2627.320...	1314.163...	876.44495	19
4	437.21833	219.11280	146.41096	A	2480.251...	1240.629...	827.42215	18
5	980.49099	490.74914	327.50185	K-Ben...	2409.214...	1205.111...	803.74311	17
6	1111.531...	556.26938	371.18201	M	1865.942...	933.47469	622.65222	16
7	1210.599...	605.80358	404.20482	V	1734.901...	867.95445	578.97206	15
8	1309.668...	655.33779	437.22762	V	1635.833...	818.42024	545.94925	14
9	1424.695...	712.85126	475.56993	D	1536.764...	768.88603	512.92645	13
10	1495.732...	748.36982	499.24897	A	1421.737...	711.37256	474.58413	12
11	1594.800...	797.90403	532.27178	V	1350.700...	675.85401	450.90510	11
12	1741.836...	871.42173	581.28358	M-Oxi...	1251.632...	626.31980	417.88229	10
13	1888.871...	944.93943	630.29538	M-Oxi...	1104.596...	552.80210	368.87049	9
14	2001.955...	1001.481...	667.99006	L	957.56152	479.28440	319.85869	8
15	2116.982...	1058.994...	706.33238	D	844.47746	422.74237	282.16400	7
16	2232.009...	1116.508...	744.67469	D	729.45052	365.22890	243.82169	6
17	2345.093...	1173.050...	782.36938	L	614.42357	307.71543	205.47938	5
18	2458.177...	1229.592...	820.06407	L	501.33951	251.17339	167.78469	4
19	2586.236...	1293.621...	862.75026	Q	388.25545	194.63136	130.09000	3
20	2699.320...	1350.163...	900.44495	L	260.19687	130.60207	87.40381	2
21				K	147.11280	74.06004	49.70912	1

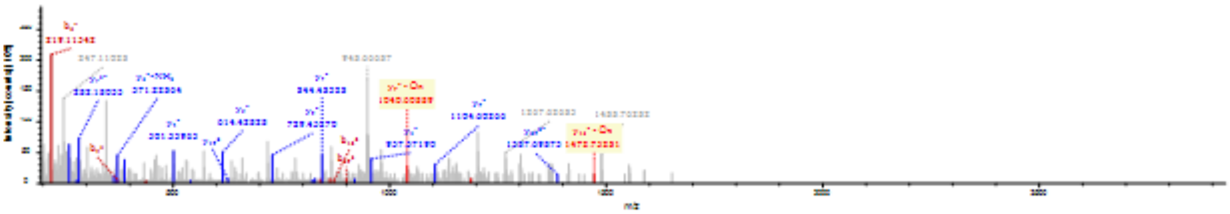

Q8TDJ6 (DmX-like protein 2)

Sequence: TLATGYEVDGGK, K12-BenKPlus415 (415.17770 Da) (position: lysine 2068)

#1	b+	b2+	Seq.	y+	y2+	#2
1	102.05496	51.53112	T			12
2	215.13902	108.07315	L	1524.725...	762.86615	11
3	286.17613	143.59170	A	1411.640...	706.32412	10
4	387.22381	194.11554	T	1340.603...	670.80556	9
5	444.24527	222.62628	G	1239.556...	620.28173	8
6	607.30860	304.15794	Y	1182.534...	591.77099	7
7	736.35120	368.67924	E	1019.471...	510.23933	6
8	835.41961	418.21344	V	890.42879	445.71803	5
9	950.44655	475.72691	D	791.36037	396.18383	4
10	1007.468...	504.23765	G	676.33343	338.67035	3
11	1064.489...	532.74838	G	619.31197	310.15962	2
12			K-Ben...	562.29050	281.64889	1

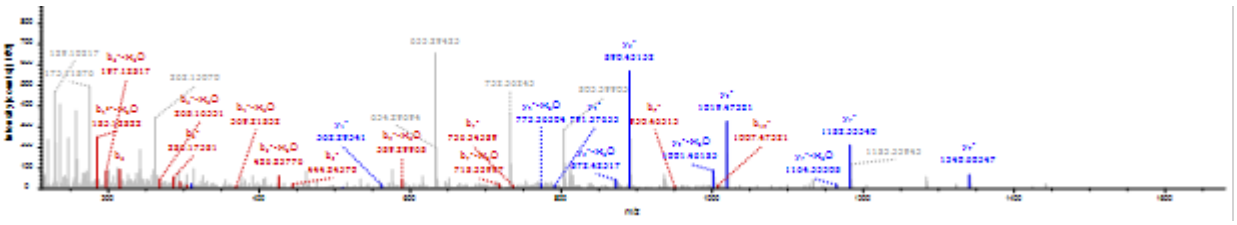

O14686 (Histone-lysine N-methyltransferase 2D)

Sequence: KGEAEGPGGK, K1-BenKPlus415 (415.17770 Da) (position: lysine 4832)

#1	b⁺	Seq.	y⁺	#2
1	544.27994	K-Ben...		10
2	601.30140	G	801.37372	9
3	730.34400	E	744.35226	8
4	801.38111	A	615.30967	7
5	930.42370	E	544.27255	6
6	987.44517	G	415.22996	5
7	1084.497...	P	358.20850	4
8	1141.519...	G	261.15573	3
9	1198.540...	G	204.13427	2
10		K	147.11280	1

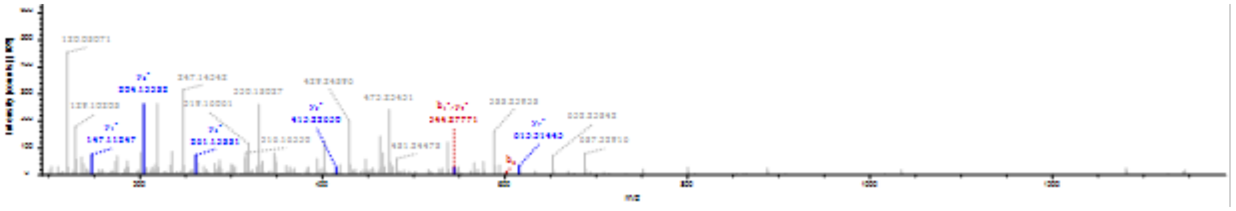

Representative peptide spectra of modified peptides (Biotin-oxime sites, +415 on lysine)
<u>All these peptides are identified from nuclear extract of LnCap without treatment with TACO chemistry.</u>

-TACO modified peptides:

Q86YW9 (Mediator of RNA polymerase II transcription subunit 12-like protein)

Sequence: LDPAGSFVPTNTK, K13-BenKPlus415 (415.17770 Da) (position: lysine 1851)

#1	b^+	b^{2+}	Seq.	y^+	y^{2+}	#2
1	114.09134	57.54931	L			13
2	229.11828	115.06278	D	1648.788…	824.89798	12
3	326.17105	163.58916	P	1533.761…	767.38451	11
4	397.20816	199.10772	A	1436.708…	718.85813	10
5	454.22962	227.61845	G	1365.671…	683.33957	9
6	541.26165	271.13446	S	1308.650…	654.82884	8
7	688.33007	344.66867	F	1221.618…	611.31283	7
8	787.39848	394.20288	V	1074.549…	537.77862	6
9	884.45124	442.72926	P	975.48155	488.24441	5
10	985.49892	493.25310	T	878.42879	439.71803	4
11	1099.541…	550.27456	N	777.38111	389.19419	3
12	1200.589…	600.79840	T	663.33818	332.17273	2
13			K-Ben…	562.29050	281.64889	1

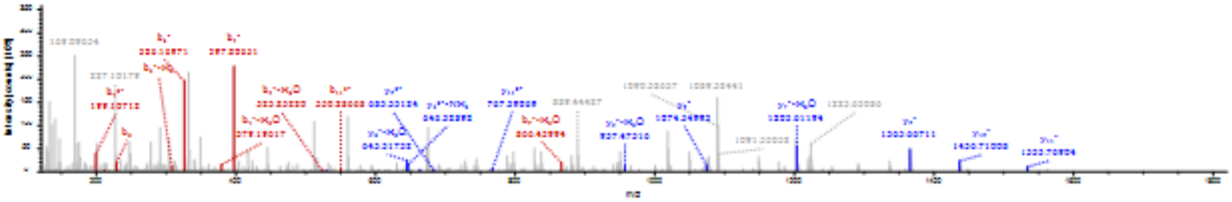

References

1. Chan, W. C.; White, P. D. Fmoc solid phase peptide synthesis: A practical approach (Oxford Univ. Press, New York, 2000).
2. Swaminathan, J.; Boulgakov, A.A.; Hernandez, E. T.; Bardo, A. M.; Bachman, J. L.; Marotta, J.; *et al.* Highly parallel single-molecule identification of proteins in zeptomole-scale mixtures. *Nat. Biotechnol.* **36**, 1076–1082 (2018).
